# Ground State Generation and Cyclization of Aminium Radicals in the Formation of Tetrahydroquinolines

**DOI:** 10.1021/acs.orglett.4c00179

**Published:** 2024-02-02

**Authors:** Cassie Pratley, Sabine Fenner, John A. Murphy

**Affiliations:** † Department of Pure and Applied Chemistry, University of Strathclyde, 295 Cathedral Street, Glasgow G1 1XL, United Kingdom; ‡ GSK Medicines Research Centre, Gunnels Wood Road, Stevenage, Hertfordshire SG1 2NY, United Kingdom

## Abstract

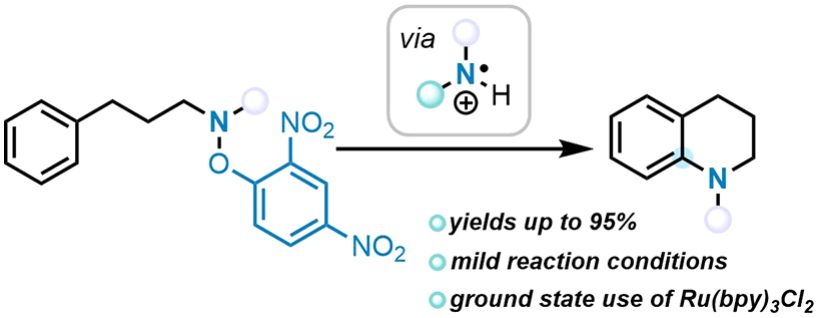

This paper reports the first examples of ground state radical-mediated intramolecular C–H amination to afford 1-methyl-1,2,3,4-tetrahydroquinolines from *N*-2,4-dinitrophenoxy derivatives of arylpropylamines. Whereas the photoactivation of *N*-2,4-dinitrophenoxyamines for intermolecular reactions has been established, ground state chemistry provides the desired cyclization products in moderate to excellent yields using Ru(bpy)_3_Cl_2_ (42–95% yields) under acidic conditions under an air atmosphere.

Nitrogen-centered radicals (NCRs) have become popular reactive intermediates for C–H amination reactions.^1^ Aminium radicals (**1**) have been of particular interest because of their efficiency in forming C–N bonds (Scheme 1a).^2−20^ These radicals can be generated by reduction of precursors RR′N–X under acidic conditions with appropriate single-electron reducing agents^2−15^ or by oxidation of RR′N–H with single-electron oxidizing agents.^16−20^ The aminium radicals can functionalize alkenes and arenes. In recent years, photoredox methods have become popular for both approaches.

**Scheme 1:**
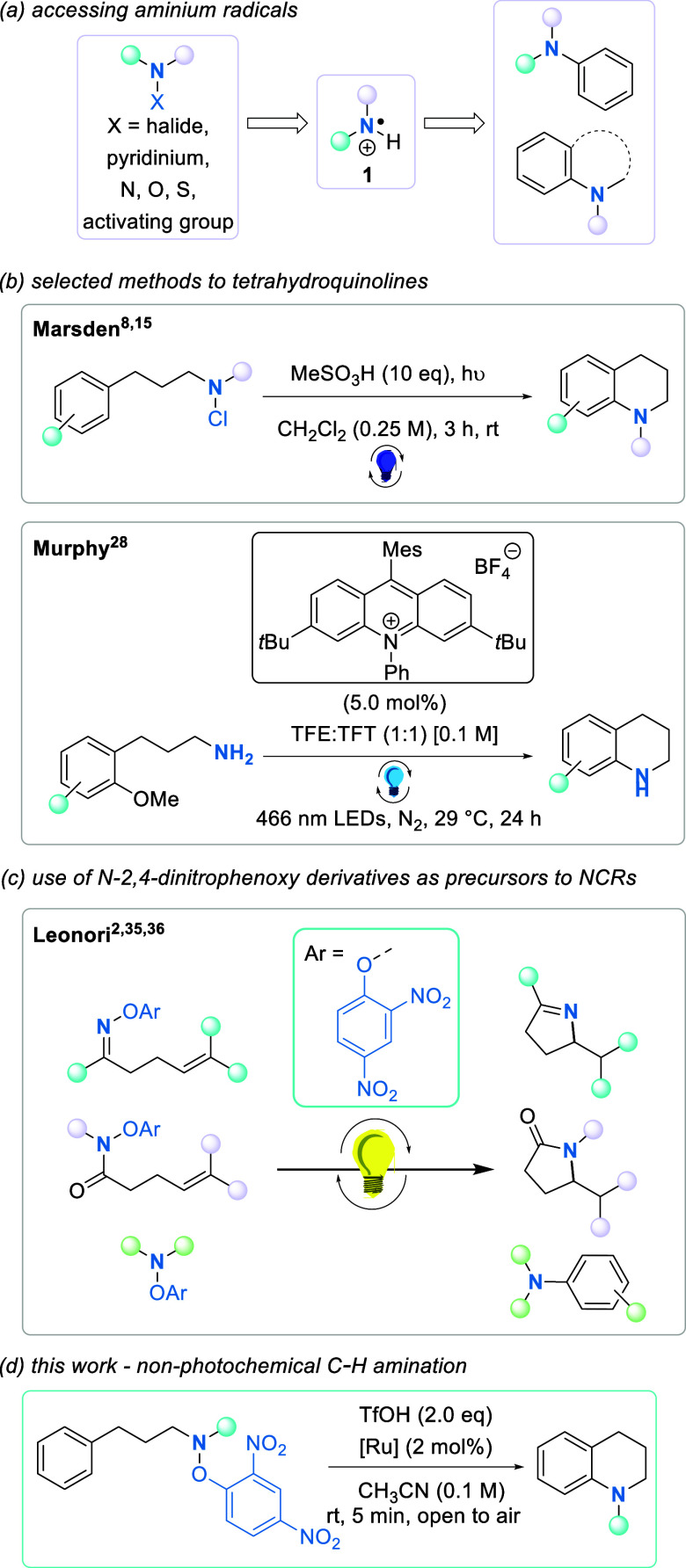
(a) General Methods for Generating Aminium Radicals (**1**), (b) Selected Routes to Tetrahydroquinolines, (c) Examples of the Leonori Group Using the *N*-2,4-Dinitrophenoxy Activating Group, and (d) This Work (intramolecular nonphotochemical C–H amination)

A special case in the amination of arenes relates to the formation of tetrahydroquinolines, which are key scaffolds in medicinal chemistry programs^21−26^ and are the focus of our interest. There are many approaches to tetrahydroquinolines, but methods particularly relevant to our studies involve the formation of the Ar–N bond. A wide variety of strategies have been used in this regard starting from arylpropanamine derivatives. Reductive approaches with iron(II) have been developed by Morandi;^11^ it is not yet clear whether these reactions are mediated by radicals or by organoiron intermediates.^12,13^ Marsden’s route^8,15^ certainly involves radicals that are formed from *N*-chloroamine derivatives under ultraviolet (UV) activation. UV activation was also used to activate *N*-iodosulfonamides to form sulfonamidyl radicals^27^ that cyclized to yield *N*-sulfonyltetrahydroquinolines. In an oxidative approach, *o*-alkoxyarylpropanamines were converted into their radical cations, leading to cyclization with displacement of the alkoxy group.^28^ Electrophilic aromatic substitution of N-bonded leaving groups, assisted by Brønsted or Lewis acids, is a popular approach to tetrahydroquinolines,^29−31^ while alternative rhodium–nitrene electrophiles were developed by Falck et al. for this purpose.^32^ Palladium-mediated C–N cross-coupling reactions also play a prominent role;^33^ the wide variety of approaches shows the high level of interest in tetrahydroquinolines.

Among the recent methods for forming Ar–N bonds through aminium radicals, the use of *N*-2,4-dinitrophenoxy derivatives is attractive. *N*-2,4-Dinitrophenoxyimines were reported as precursors to NCRs by Narasaka et al.,^34^ leading to iminyl radicals that cyclized to pyrrolenines under UV irradiation. More recently, this type of precursor has been deployed under photoredox conditions using visible light. Extensive progress in this area has been made by the Leonori group, who published the cyclization of iminyl radicals to pyrrolenines,^35^ the cyclization of amidyl radicals to γ-lactams,^36^ and the intermolecular C–H amination of (hetero)arenes with aminium radicals^2^ (Scheme 1c).

In Leonori’s paper,^2^ aminium radicals were generated by the protonation of *O*-2,4-dinitrophenoxyamines under strongly acidic conditions (HClO_4_) followed by single-electron transfer (SET) reduction by photoredox catalysis. This led to fragmentation to the highly electrophilic aminium radical species (**1**) that then participated in radical additions to aromatic compounds with high regioselectivity. Our work focuses on the use of these precursors for the synthesis of 1-methyl-1,2,3,4-tetrahydroquinolines (Scheme 1d).^37,38,41^

Our interest in the report by Leonori^1^ was in the example shown in Scheme 2i, which looked at the nonphotochemical amination of *tert*-butylbenzene (**2**) with NCR precursor **3** in the presence of ruthenium catalyst Ru(bpy)_3_Cl_2_ to afford **4** in 51% yield. Intrigued by this example, we envisaged that this protocol might work better in our intramolecular setting. Aminium radicals would be generated on secondary amines under nonphotochemical conditions and cyclize rapidly to afford 1-methyl-1,2,3,4-tetrahydroquinolines.

**Scheme 2:**
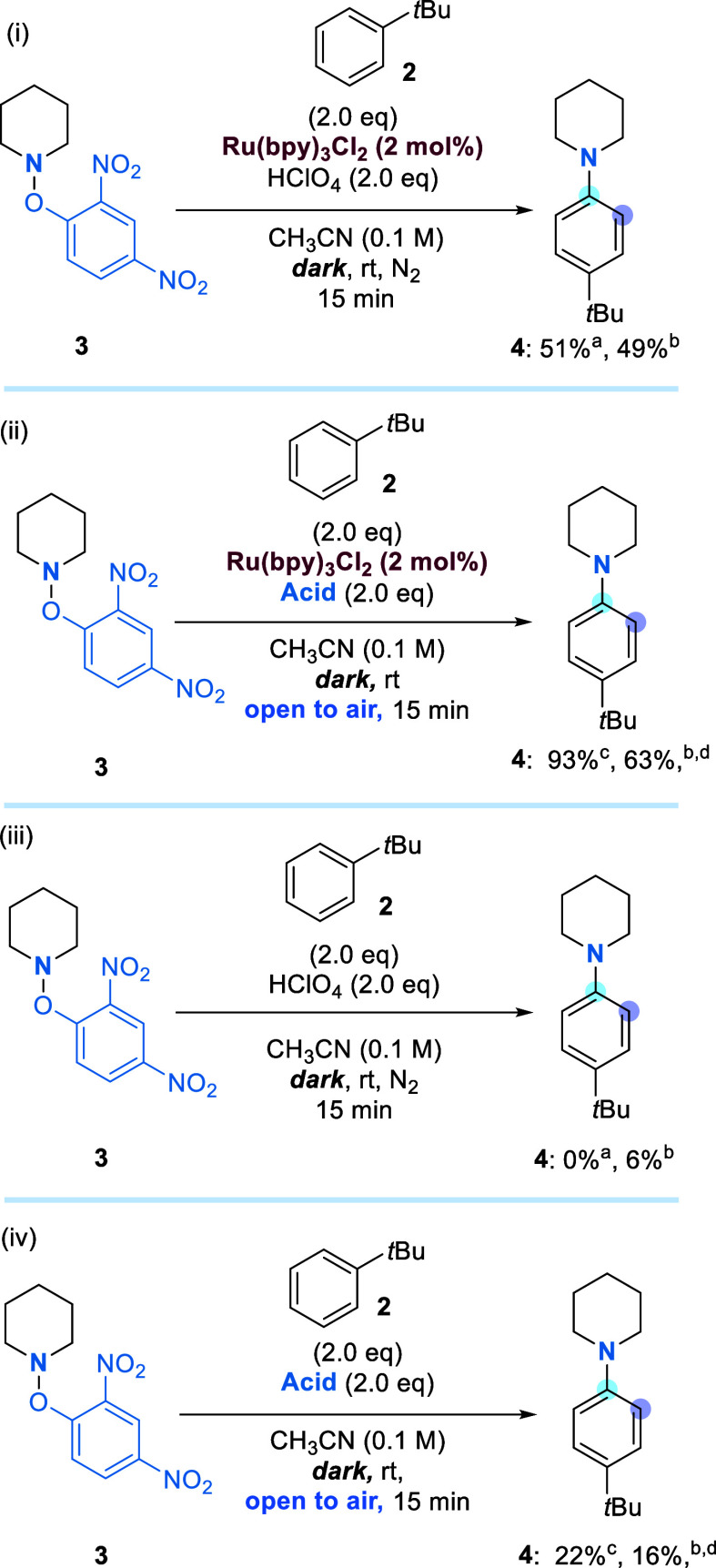
Comparison of Control Reactions Using *tert*-Butylbenzene (**2**) as the Arene Source ^1^H NMR yield reported by Leonori and co-workers.^2^ ^1^H NMR yield. Using TfOH and isolated yield. Using perchloric acid.

Our explorations of the chemistry in Scheme 2 revealed that an inert atmosphere was not required for the reaction to proceed well, and in fact, the presence of air enhanced the reaction [93% (Scheme 2ii)]. The beneficial use of oxygen as an easily accessible oxidant in other amination reactions has been observed by Nicewicz^18^ and Hashmi.^39^ The Leonori publication^2^ did indeed report product formation in the absence of light; however, this was not elaborated due to the superior yields that were achieved with the photoactivation method.

In our hands, changing the acid from perchloric acid (63%) to triflic acid (93%) also had a beneficial effect on the yield of the reaction (Scheme 2ii), and thus, this was the acid used in future reactions. Perchloric acid has known hazards, and its corresponding salts can be explosive in nature;^40^ therefore, a change in acid to triflic acid was desirable for the sake of safety. Next, the reaction was tested in the absence of Ru(bpy)_3_Cl_2_. In contrast to the results of Leonori [0% (Scheme 2iii)], the reaction was found to take place to some extent in the absence of the [Ru] catalyst [22% (Scheme 2iv)].

With these observations in mind, we probed the reaction parameters to gain a better understanding of the key components of the reaction. Anisole was adopted as the arene, leading to piperidine **5** as a mixture of *ortho* and *para* isomers. The standard reaction conditions are shown in Table 1, and the variations are shown in the table with a focus on finding better conditions without a catalyst.

**Table 1:**
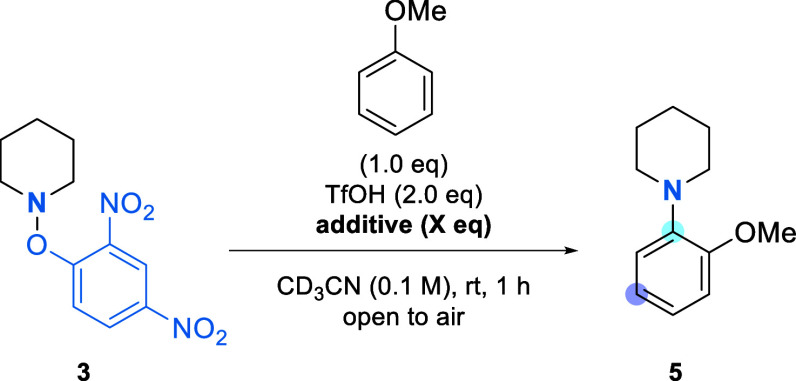
Optimizing the Reaction Conditions

entry	variations^a^	dark reaction	^1^H NMR yield (%) of **5** (*o*:*p*)
1	–	–	56 (1:2)
2	no acid	–	0
3	under N_2_	–	22 (1:1)
4^b^	–	yes	51 (1:2)
5^c^	Ru(bpy)_3_Cl_2_·6H_2_O	–	81 (1.2:1)
6^c^	Eosin Y	–	39 (1:2)
7^c^	[Ir(dtbbpy)(ppy)_2_]PF_6_	–	32 (1:2)
8^c^	Ru(bpy)_3_Cl_2_·6H_2_O under N_2_	–	9 (1.5:1)
9^c^	Ru(bpy)_3_Cl_2_·6H_2_O under N_2_	yes	11 (1.4:1)
10^c^	Ru(bpy)_3_Cl_2_·6H_2_O without acid	yes	0

a All reactions were conducted on a 0.0613 mmol scale in *d*^3^-acetonitrile, at room temperature, open to air, and at a concentration of 0.1 M, unless stated otherwise.

b The reaction was carried out in an amber HPLC vial in a blacked out box (including the addition of acid).

c In cases in which an additive was used, the additive is listed under “variations” and was used at a 2 mol % level.

Entry 1 shows the parent conditions that afforded product **5** in 56% yield. In the absence of an acid (entry 2), no reactivity was observed. Entry 3 shows the results obtained when the experiment was carried out under an inert atmosphere (N_2_). Surprisingly, the remaining starting material **3** was detected (40%), showing that the reaction did not reach completion. This can be contrasted with the exposure to air in entry 1 that resulted in a better yield for the reaction relative to entry 3 as well as the full consumption of **3**. To determine whether light was required for the transformation, we performed a reaction in the dark (entry 4). A comparable ^1^H NMR yield was obtained (51%) when the reaction mixture was exposed to general laboratory light as reported in entry 1, thus confirming that light was not assisting the reaction. For the sake of completeness, the reaction was performed in ambient light with a [Ru] photocatalyst present (entry 5). Interestingly, the yield of the reaction increased dramatically, and the desired product was obtained in excellent yield (81%). This result was comparable with the works of Leonori, who obtained **5** in 77% yield; however, this previous result used light irradiation and an inert atmosphere as compared with our conditions of exposure to air.^2^ In our case, an interesting observation was also made regarding the regioselectivity of the reaction; a switch in selectivity was observed from favoring *para* (1:2 *o*:*p*) (with no catalyst present) to an *ortho*-directed transformation (1.2:1 *o*:*p*). Entries 6 and 7 show that other redox agents can also achieve this chemistry, albeit in lower yields. The final three entries (8–10) look into the control reaction with the [Ru] catalyst as an additive. Entries 8 and 9 show the impact of an inert atmosphere, and both fail to achieve good yields. However, upon comparison of the two, performing the reaction in the dark has a negligible effect. Entry 10 demonstrates that in the presence of the [Ru] catalyst, acid still plays a key role in the reaction. The reaction conditions worked well for a number of substrates in an intermolecular fashion (Scheme 3).

**Scheme 3:**
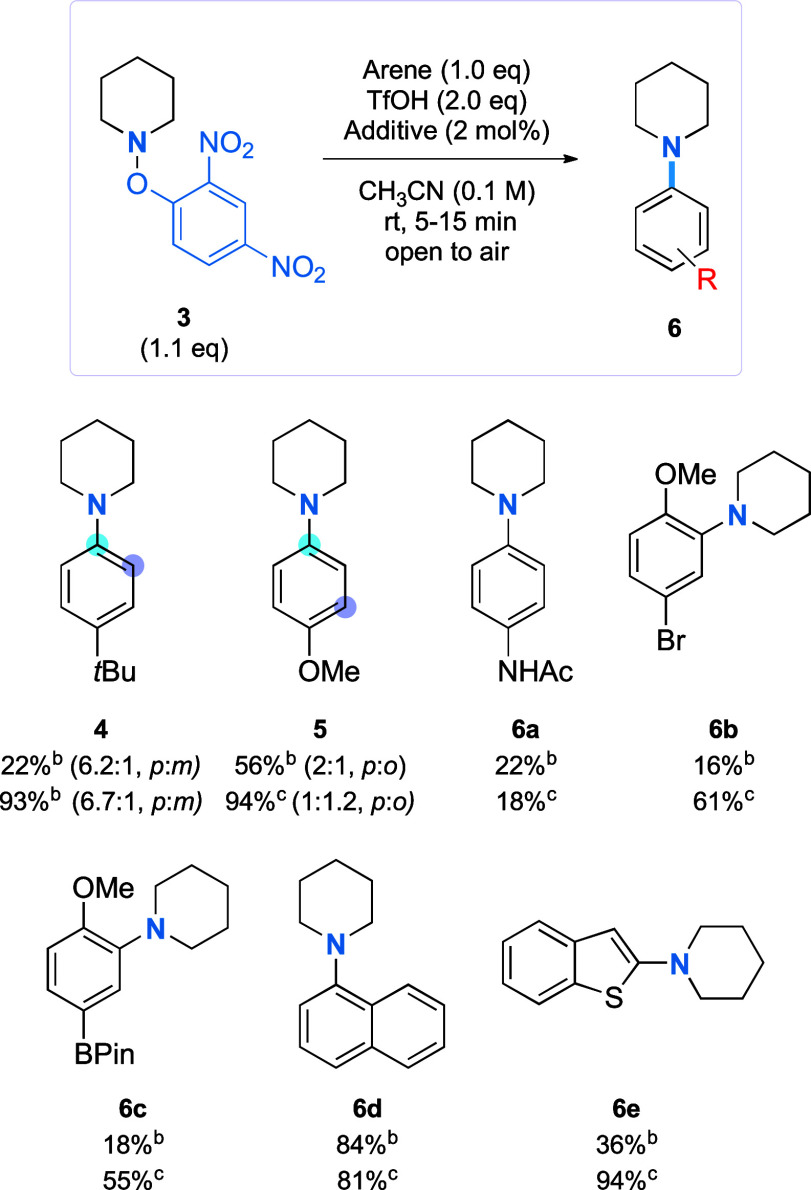
Radical-Mediated Intermolecular C–H Amination of Arenes with Piperidine No additive was used. Ru(bpy)_3_Cl_2_·6H_2_O was used (2 mol %) as the additive. Yields are reported as isolated yields, following column chromatography. The major isomer is shown; in the minor isomer, the site of substitution by the amine is indicated by a purple-colored atom.

The effect of the [Ru] additive on the intermolecular reactions was assessed by performing reactions with and without the additive. Overall, the yields obtained in the presence of the [Ru] catalyst were consistently higher. Electron-rich arenes performed best, generating the corresponding aniline products in good to excellent yields in the presence of [Ru] (**4**, **5**, and **6b–6e**). In the case of the acetanilide example (**6a**), a decreased yield was observed, which possibly arises from protonation of the oxygen of the acyl group, which could retard the amination.

The ground state transformation was adapted to an intramolecular setting for the synthesis of 1,2,3,4-tetrahydroquinoline derivatives (Scheme 4). The presence of alkyl and phenyl groups at position 4 of the substrates afforded the cyclized products in very good yields (**8b** and **8c**). Interestingly, two products (**8d** and **8d′**) were formed in the example in which a phenyl substituent was used. The second product (**8d′**) arose by initial 5-*exo* cyclization at the *ipso* position, which could be followed directly by a C–C bond fragmentation, *ortho* cyclization, and rearomatization or the intermediate spirocyclic radical could undergo electron transfer to form a cation that could then form the product following bond migration and deprotonation. Methyl ether and halide functionalities at the same position were examined and successfully cyclized to the desired products (**8e–8g**). Next, substitution at position 3 was explored and gave rise to the corresponding methyl ether and bromo products (**8h** and **8i**, respectively). Regioisomers of **8h** were isolated, favoring the *para* position. This aligns with the case of intermolecular functionalization of anisole, in which the *para* position was the most activated.^1,42^ Intriguingly, only a single regioisomer was isolated in the 3-bromo example (**8i**).^11^ For *ortho*-substituted substrates, products with alkyl (**8j**) and halo (**8l** and **8k**) functionalities were isolated, although lower yields were obtained. No *ipso* substitution was observed in any of these cases. The precursors to tetrahydroquinolines (**8m** and **8n**) were cyclized to the products in very good yields. Product benzomorpholine (**8o**) demonstrated that successful substrates were not confined to arylpropylamine derivatives.

**Scheme 4:**
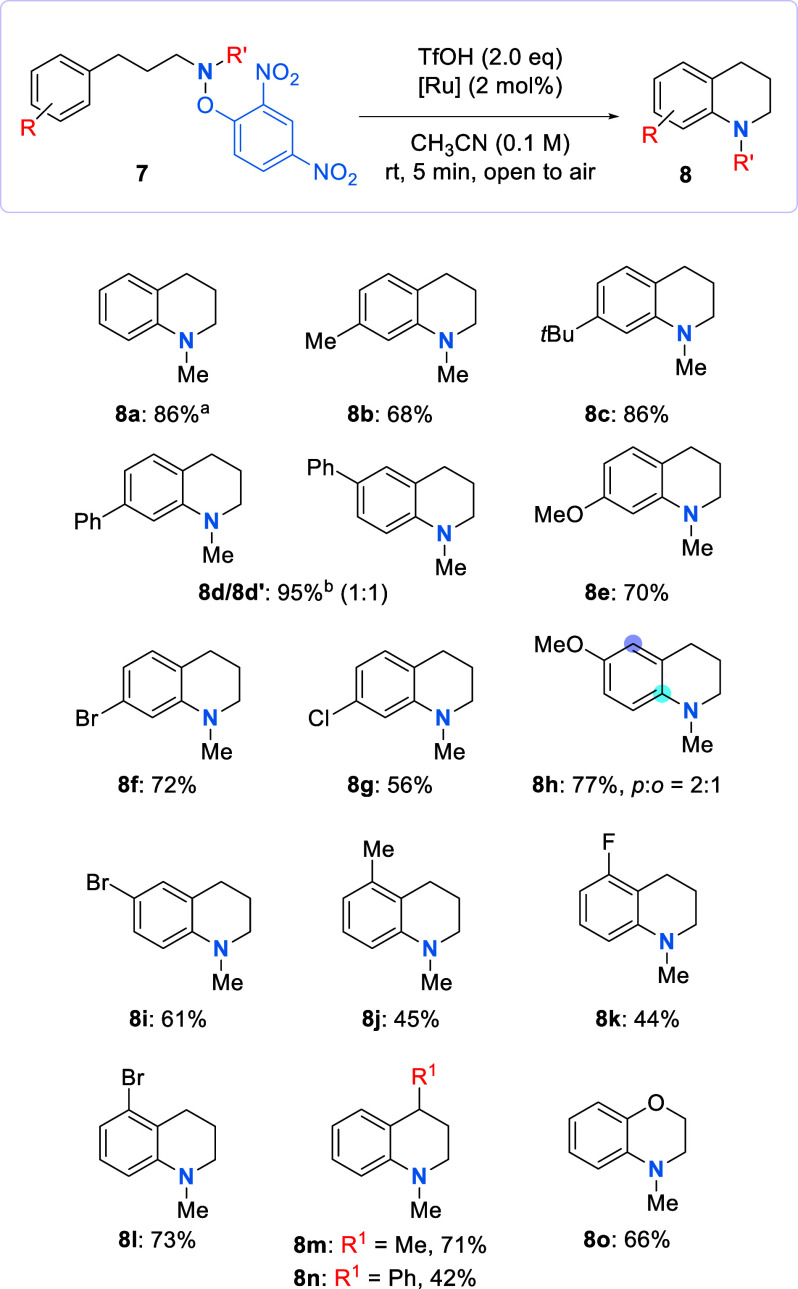
Radical-Mediated Intramolecular C–H Amination of Secondary Amines On a 3.36 mmol scale. A mixture of the two isomers was formed. Yields are reported as isolated yields following column chromatography. The major isomer is shown; the site of C–N bond formation in the minor isomer is indicated at the site of a purple-colored atom.

The mechanism for the formation of these C–N bonds likely features a radical chain mechanism (Scheme 5). When the [Ru] catalyst is present, it is beneficial in the initial step of reductive SET to the protonated NCR precursor. In the case without the [Ru] catalyst, under super acid conditions, spontaneous N–O bond homolysis can occur after protonation of the precursor (**7**).

**Scheme 5:**
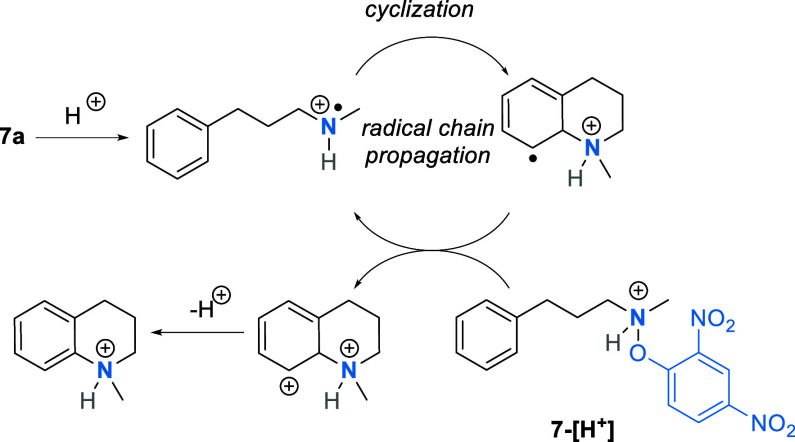
Radical Chain Mechanism of the Intramolecular C–H Amination of Arenes

In conclusion, we report the first examples of ground state radical-mediated intramolecular C–H amination to afford 1-methyl-1,2,3,4-tetrahydroquinolines from *N*-2,4-dinitrophenoxy derivatives of arylpropylamines. In these cases, the ruthenium complex that is normally deployed as a photocatalyst is found to be useful in the ground state. We are now investigating further applications of these protocols.

## Data Availability

The data underlying this study are available in the published article and its Supporting Information.

## Supporting Information Available

Experimental details and spectroscopic information that supports the structural assignments of the products (PDF)
