# Supplementary material for: Ground State Generation and Cyclization of Aminium Radicals in the Formation of Tetrahydroquinolines
Source: Org Lett. 2024 Feb 2;26(6):1287–92. doi: 10.1021/acs.orglett.4c00179 (PMC10877599; doi:10.1021/acs.orglett.4c00179)

Ground state generation and cyclization of aminium radicals in the formation of tetrahydroquinolines.

Cassie Pratley[a,b], Sabine Fenner[b] and John A. Murphy[a]

[a]Department of Pure and Applied Chemistry, University of Strathclyde, 295 Cathedral Street, Glasgow G1 1XL, United Kingdom; [b]GSK Medicines Research Centre, Gunnels Wood Road, Stevenage, Herts SG1 2NY, United Kingdom.

Contents

1. General Experimental Details 2
2. Synthesis of 1-(2,4-dinitrophenoxy)piperidine (**3**) 4
3. Control Reactions 4
4. Intermolecular Parameter Experiments (Table 1) 7
5. Intermolecular Substrates 10
6. Intramolecular Amination 16
7. References 47
8. NMR Spectra 49

1. General Experimental Details

All commercially obtained chemicals were used as received.

Thin layer chromatography (TLC) was carried out using POLYGRAM® SIL G/UV_{254} plates. Visualisation of the results was obtained using UV light or developed using a potassium permanganate dip.

LCMS data were obtained using a Waters® Acquity UPLC™ CSH C18 column (2.1 mm x 50 mm i.d. 1.7 µm packing diameter) at 40 °C and a flow rate of 1 mL/min. Samples were eluted with either modified 10 mM ammonium bicarbonate adjusted to pH 10 or 0.1% formic acid, water (solvent A) to acetonitrile (solvent B) over a 2-minute run. The gradient of elution is as stated:

Time / min	% A	% B
0.00	97	3
0.05	97	3
1.50	5	95
1.90	5	95
2.00	97	3

UV detection occurred in the range of 210nm to 350 nm. Mass spectra were recorded on a Waters ZQ mass spectrometer using alternating positive and negative electrospray ionisation (ES+ and ES-). All LCMS values stated were run in a 0.1% formic acid, water to acetonitrile solvent system, unless stated otherwise.

NMR spectra were obtained on a Bruker AV-400 instrument (^{1}H = 400 MHz, ^{13}C = 101 MHz) with samples in $CDCl_3$. Chemical shifts (δ) are reported in ppm relative to tetramethylsilane (TMS) (δ = 0.00). Coupling constants are reported in Hertz (Hz) and signal multiplicity is denoted as singlet (s), doublet (d), triplet (t), quartet (q), quintet (quin), multiplet (m), doublet of doublets (dd), quartet of triplets (qt) and broad (br). Structural assignments were made with additional information from gCOSY, gROESY, and gHMBC experiments.

Normal phase purification was carried out by Flash Column Chromatography using an Isolera 4 purification system. UV detection was monitored at 254 nm and 220 nm unless stated otherwise. Pre-packed Biotage® SNAP Ultra or Biotage® SNAP KP-NH cartridges were used eluting with the following solvent systems: EtOAc:heptane or MeOH: 1% Et_3N modified CH_2Cl_2, where modified solvents were prepared in-house.

Reverse phase purification was carried out on the EZ prep and was conducted on an XSelect® CSH™ prep C18 (100 mm x 30 mm, 5µm OBD™) column, eluting with modified 0.1% formic acid modified H_2O:MeCN gradient. UV detection was monitored at 254 nm and 220 nm. Larger scale purifications

were carried out on a Teledyne Isco Combi Flash Companion machine using Biotage® SNAP Ultra C18 cartridges eluting with modified 0.1% formic acid modified H_2O:MeCN gradient. UV detection was monitored at 254 nm and 220 nm.

Infrared Spectroscopy (IR) was carried out on a Perkin Elmer Spectrum One spectrometer. Absorption frequencies (υ_{max}) are reported in wavenumbers (cm^{-1}). Only selected absorbances are reported.

Melting points were measured on a Büchi Melting Point M-565.

High Resolution Mass Spectrometry (HRMS) was conducted using one of two methods. Conditions for the 20 min high pH method were as follows: Acquity UPLC BEH C18 column (100mm x 2.1mm i.d. 1.7µm packing diameter) at 50 °C. The solvents employed were: A = 10 mM ammonium bicarbonate in water adjusted to pH 10 with ammonia solution. B = acetonitrile. The gradient employed was:

Time (min)	Flow Rate (mL/min)	% A	% B
0.0	0.8	99	1
0.5	0.8	99	1
17.0	0.8	10	90
18.5	0.8	10	90
19.0	0.8	99	1
20.0	0.8	99	1

The UV detection was a summed signal from wavelengths between 210 nm and 500 nm.

Conditions for the 10 min formic acid method are as follows: Acquity UPLC CSH C18 column (100mm x 2.1mm i.d. 1.7µm packing diameter) at 50 °C. The solvents employed were: A = 0.1% v/v solution of formic acid in water. B = 0.1% v/v solution of formic acid in acetonitrile. The gradient employed was:

Time (min)	Flow Rate (mL/min)	% A	% B
0.0	0.8	95	5
8.5	0.8	7	93
9.0	0.8	7	93
9.5	0.8	95	5
10.0	0.8	95	5

The UV detection was a summed signal from wavelengths between 210 nm and 500 nm.

The MS conditions used for both methods were: Waters XEVO G2-XS QTOF with a positive electrospray ionisation mode and a scan range of 100 to 1200 AMU.

2. Synthesis of 1-(2,4-dinitrophenoxy)piperidine (**3**)

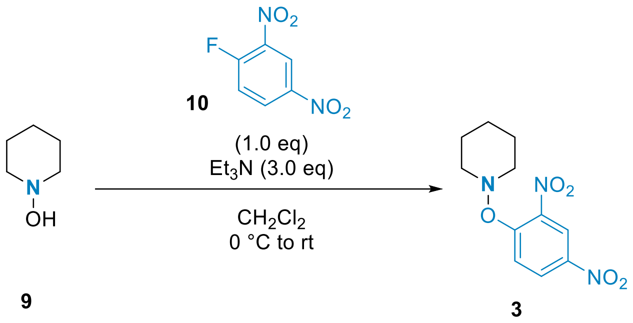

Piperidin-1-ol (**9**, 1.1 eq, 1.0 g, 9.89 mmol) was dissolved in CH_2Cl_2 (40 mL) and cooled to 0 °C before being treated with triethylamine (3.0 eq, 2.77 g, 3.82 mL, 27.4 mmol). 1-Fluoro-2,4-dinitrobenzene (**10**, 1.0 eq, 1.7 g, 9.13 mmol) was dissolved in CH_2Cl_2 (5 mL) and added dropwise to the reaction mixture. The mixture was warmed to rt overnight and stirred for 16 h. The reaction was diluted with water (15 mL) and the layers were separated. The aqueous layer was extracted with CH_2Cl_2 (3 x 20 mL), the organics were combined, dried through a phase separator, and concentrated *in vacuo* to give a dark red oil. The crude product was loaded onto a 100 g Biotage Sfar column and eluted with 0-40% EtOAc:heptane (12 column volumes). The relevant fractions were combined and concentrated *in vacuo* to give **3** as a yellow solid (2.055 g, 7.69 mmol, 84 % yield). **^{1}H NMR** (400 MHz, $CDCl_3$) δ = 8.79 (d, *J* = 2.7 Hz, 1 H), 8.39 (dd, *J* = 9.4, 2.7 Hz, 1 H), 7.89 (d, *J* = 9.4 Hz, 1 H), 3.42 - 3.35 (m, 2 H), 2.94 - 2.86 (m, 2 H), 1.95 - 1.88 (m, 2 H), 1.77 - 1.67 (m, 3 H), 1.40 - 1.27 (m, 1 H). **^{13}C NMR** (101 MHz, $CDCl_3$) δ = 157.9, 140.1, 136.4, 129.3, 122.0, 116.9, 57.0, 25.2, 23.0. Data were in accordance with previously reported literature.[1]

3. Control Reactions

3.1 Running the Amination Reactions under N_2 Atmosphere with No Light Irradiation:

(i) In the presence of $Ru(bpy)_3Cl_2$ with perchloric acid: : [Scheme 2, (i)]

To a dry 0.5-2 mL microwave vial was added 1-(2,4-dinitrophenoxy)piperidine (**3**, 1.0 eq, 27 mg, 0.101 mmol), $Ru(bpy)_3Cl_2.H_2O$ (2 mol%, 2.0 mg, 2.00 µmol) and the stirrer bar. The vial was capped with a crimp seal and was purged with N_2 (x 3). To the vial was added *tert*-butylbenzene (2.0 eq, 34 µL, 0.202 mmol) and CH_3CN (1 mL, 0.1 M) and the vial was degassed with N_2 for 15 min. To the vial was added perchloric acid (2.0 eq, 17 µL, 0.202 mmol) and the mixture was stirred in the absence of light for 15 min at rt. An immediate colour change to a green coloured solution was observed, indicating the formation of [Ru^{3+}].[2,3] The reaction mixture was quenched with aqueous 1 M KOH solution (1 mL) which was stirred for 15 min before being diluted with water (10 mL) and EtOAc (15 mL). The aqueous layer was extracted with EtOAc (3 x 15 mL). The organic layers were combined and filtered through a hydrophobic frit and concentrated *in vacuo*. Then 1,3,5-trimethoxybenzene (0.235 eq, 4 mg, 0.024

mmol) was added as an NMR calibrant and the sample was taken up in $CDCl_3$ (0.6 mL) and analysed by ^{1}H NMR. A ^{1}H NMR yield of 49% was calculated (2:1, *para*:*meta*). The characterisation for the products is stated in Section 5: Intermolecular Substrates.

(ii) In the absence of $Ru(bpy)_3Cl_2$ with perchloric acid: : [Scheme 2, (iii)]

To a dry 0.5-2 mL microwave vial was added 1-(2,4-dinitrophenoxy)piperidine (**3**, 1.0 eq, 27 mg, 0.101 mmol) and the stirrer bar. The vial was capped with a crimp seal and was purged with N_2 (x 3). To the vial was added *tert*-butylbenzene (2.0 eq, 34 µL, 0.202 mmol) and CH_3CN (1 mL, 0.1 M) and the vial was degassed with N_2 for 15 min. To the vial was added perchloric acid (2.0 eq, 17 µL, 0.202 mmol) and the mixture was stirred in the absence of light for 15 min at rt. The reaction mixture was quenched with aqueous 1 M KOH solution (1 mL) which was stirred for 15 min before being diluted with water (10 mL) and EtOAc (15 mL). The aqueous layer was extracted with EtOAc (3 x 15 mL). The organic layers were combined and filtered through a hydrophobic frit and concentrated *in vacuo*. Then 1,3,5-trimethoxybenzene (0.235 eq, 4 mg, 0.024 mmol) was added as an NMR calibrant and the sample was taken up in $CDCl_3$ (0.6 mL) and analysed by ^{1}H NMR. A ^{1}H NMR yield of 6% was calculated (2:1, *para*:*meta*).

3.2 Running the Amination Reactions Open to Air with No Light Irradiation: : [Scheme 2, (iv)]

(i) In the presence of $Ru(bpy)_3Cl_2$:

A vial was charged with a stirrer bar, $Ru(bpy)_3Cl_2.H_2O$ (6.77 mg, 9.04 µmol), 1-(2,4-dinitrophenoxy)piperidine (**3**, 133 mg, 0.497 mmol), *tert*-butylbenzene (70 µL, 0.452 mmol) and CH_3CN (0.1 M, 3.75 mL) which was left to stir for 1 min. To the vial was added TfOH (80 µL, 0.904 mmol) and the vial was stirred for 10 min. The reaction mixture was quenched with 1 M aqueous KOH solution (5 mL) before being diluted with water (5 mL) and CH_2Cl_2 (20 mL) and the layers were separated. The organic layer was collected and extracted with 1 M aqueous KOH solution (10 mL). The organic layer was filtered through a hydrophobic frit and concentrated *in vacuo* to give a red gum. The sample was purified on a 5 g Sfar Biotage column, eluting with 0-40% EtOAc:heptane (10 column volumes). The relevant fractions were combined and concentrated *in vacuo* to give a yellow oil as a mixture of 1-(4-(*tert*-butyl)phenyl)piperidine and 1-(3-(*tert*-butyl)phenyl)piperidine (**4'**:**4"** [6.7:1], 91.3 mg, 93%).

This procedure was repeated with perchloric acid and a yellow oil was isolated as a mixture of 1-(4-(*tert*-butyl)phenyl)piperidine and 1-(3-(*tert*-butyl)phenyl)piperidine (**4'**:**4"** [6.5:1], 61.9 mg, 63%).

(ii) In the absence of $Ru(bpy)_3Cl_2$:

A vial was charged with a stirrer bar, 1-(2,4-dinitrophenoxy)piperidine (**3**, 1.1 eq, 133 mg, 0.497 mmol), *tert*-butylbenzene (1.0 eq, 63.5 µL, 0.410 mmol) and CH_3CN (0.1 M, 3.75 mL) which was left to stir for 1 min. To the vial was added TfOH (2.0 eq, 80 µL, 0.904 mmol) and the vial was stirred for 10 min. The

reaction mixture was quenched with 1 M aqueous KOH solution (5 mL) before being diluted with water (5 mL) and CH_2Cl_2 (20 mL) and the layers were separated. The organic layer was collected and extracted with 1 M aqueous KOH solution (10 mL). The organic layer was filtered through a hydrophobic frit and concentrated *in vacuo* to give a red gum. The sample was purified on a 5 g Sfar Biotage column, eluting with 0-40% EtOAc:heptane (10 column volumes). The relevant fractions were combined and concentrated *in vacuo* to give a yellow oil as a mixture of 1-(4-(*tert*-butyl)phenyl)piperidine and 1-(3-(*tert*-butyl)phenyl)piperidine (**4'**:**4"** [6.2:1], 20 mg, 22%). The ratio of regioisomers was analysed by ^{1}H NMR and the relative integrations of the *tert*-butyl protons.

This procedure was repeated with perchloric acid and a yellow oil was isolated as a mixture of 1-(4-(*tert*-butyl)phenyl)piperidine and 1-(3-(*tert*-butyl)phenyl)piperidine (**4'**:**4"** [6.3:1], 15.7 mg, 16%).

3.3 Testing the Oxidation of the [Ru] Catalyst with Acid Only:

(i) Perchloric Acid

To a vial containing CH_3CN (1.2 mL) and $Ru(bpy)_3Cl_2·6H_2O$ (2 mol%, 2 mg, 2 µmoL) was added perchloric acid (5 eq, 36.4 µL, 0.604 mmol) and the reaction was stirred for 5 min. There was no observation of a green coloured solution, therefore the acid is unlikely to be oxidising the [Ru] catalyst alone.

(ii) Triflic Acid

To a vial containing CH_3CN (1.2 mL) and $Ru(bpy)_3Cl_2·6H_2O$ (2 mol%, 2 mg, 2 µmoL) was added triflic acid (5 eq, 53.6 µL, 0.604 mmol) and the reaction was stirred for 5 min. There was no observation of a green coloured solution, therefore the acid is unlikely to be oxidising the [Ru] catalyst alone.

3.4 Acid Optimisation

For ^{1}H NMR yields, the OMe was used as the reference peak. For 1-(4-methoxyphenyl)piperidine (**5'**): δ 3.78, 3H; for 1-(2-methoxyphenyl)piperidine (**5"**): δ 3.88, 3H.

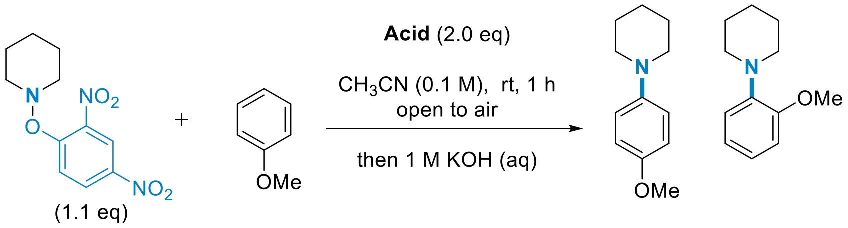

3 **11** **5'** **5"**

(i) Sulfuric Acid

A 20 mL vial was charged with a stirrer bar 1-(2,4-dinitrophenoxy)piperidine (**3,** 1.1 eq, 110 mg, 0.412 mmol), anisole (**11**) (1.0 eq, 40 µL, 0.374 mmol) and CH_3CN (3750 µL) and left to stir for 5 min. To the vial was added H_2SO_4 (2.0 eq, 42 µL, 0.749 mmol) and the mixture was stirred for 1 h. The reaction mixture was quenched with 1 M aqueous KOH solution (5 mL) before being diluted with water (5 mL)

and CH_2Cl_2 (20 mL) and the layers were separated. The organic layer was collected and extracted with 1 M aqueous KOH solution (10 mL). The organic layer was collected, filtered through a hydrophobic frit and terephthalonitrile (0.54 eq, 26 mg, 0.203 mmol) was added as an internal standard. The sample was concentrated *in vacuo* and a quantitative ^{1}H NMR was taken which gave a ^{1}H NMR yield of **5'**:**5"** as 10% (2:1, *para:ortho*).

(ii) Triflic Acid

A 20 mL vial was charged with a stirrer bar 1-(2,4-dinitrophenoxy)piperidine (**3**) (1.1 eq, 120 mg, 0.449 mmol), anisole (**11**) (1.0 eq, 45 µL, 0.414 mmol) and CH_3CN (3.75 mL) and left to stir for 5 min. To the vial was added triflic acid (2.0 eq, 73.5 µL, 0.828 mmol) and the mixture was stirred for 1 h. The reaction mixture was quenched with 1 M aqueous KOH solution (5 mL) before being diluted with water (5 mL) and CH_2Cl_2 (20 mL) and the layers were separated. The organic layer was collected and extracted with 1 M aqueous KOH solution (10 mL). The organic layer was collected, filtered through a hydrophobic frit and terephthalonitrile (0.25 eq, 13 mg, 0.101 mmol) was added as an internal standard. The sample was concentrated *in vacuo* and a quantitative ^{1}H NMR was taken which gave a ^{1}H NMR yield of 78% (1:2.2 *ortho:para*). The ^{1}H NMR sample was concentrated *in vacuo* and purified on a 5 g Sfar Biotage column, eluting with Et_2O:pentane, 0-40% (10 column volumes). The relevant fractions were combined and concentrated *in vacuo* to give 1-(2-methoxyphenyl)piperidine (**5"**) and 1-(4-methoxyphenyl)piperidine (**5'**) as a yellow oil (56%, 1:2, *ortho:para*).

4. Intermolecular Parameter Experiments (Table 1)

For ^{1}H NMR yields, the OMe was used as the reference peak. For 1-(4-methoxyphenyl)piperidine: δ 3.78, 3H, for 1-(2-methoxyphenyl)piperidine: δ 3.88, 3H.

A variety of reaction parameters were trialled in these experiments. For ease of analysis by ^{1}H NMR, the reactions were run in CD_3CN. The stock solution of starting materials was freshly prepared, a total of 6 x 0.6 mL reactions at one time containing the NCR precursor and the aromatic coupling partner. The internal standard (terephthalonitrile) for the ^{1}H NMR analysis was added after the reaction had been left for 1 h, to avoid any interference of the internal standard with the reaction. A stock solution was freshly prepared for the addition of the internal standard to the ^{1}H NMR tubes (6 x 0.1 mL samples).

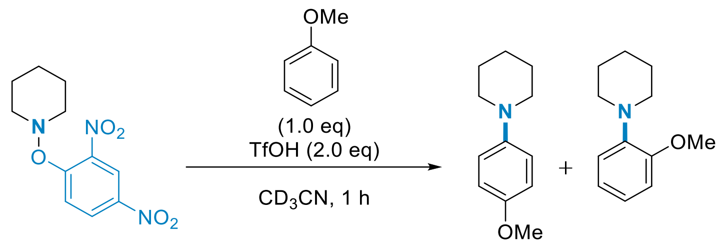

3 **5'** **5"**

*Preparation of 0.1 M Stock Solution **A** (NCR precursor **3** and anisole)*

A 20 mL glass vial was charged with a magnetic stirrer bar. To the vial were added anisole (anhydrous) (1.0 eq, 40 µL, 0.368 mmol), 1-(2,4-dinitrophenoxy)piperidine (**3**, 1.1 eq, 108 mg, 0.404 mmol) and acetonitrile-d^3 (3680 µL), which were left to stir for 5 min.

*Preparation of Internal Standard Stock Solution **B** (terephthalonitrile)*

A 2 mL HPLC vial was charged with a magnetic stirrer bar. To the vial were added terephthalonitrile (0.1 eq, 13 mg, 0.101 mmol) and acetonitrile-d^3 (600 µL) which were left to stir for 5 min. For each reaction, the ^{1}H NMR tube was dosed with 100 µL of stock solution (0.01683 mmol per reaction).

4.1 General Procedure for Reactions Run Open to Air (GENERAL PROCEDURE 1)

With a Catalyst (General Procedure 1.1):

A 2 mL glass HPLC vial was equipped with a small stirrer bar and ***catalyst*** (2 mol%) was added. The vial was dosed with 600 µL stock solution **A**. To the vial was added triflic acid (2.0 eq, 11 µL, 0.123 mmol) which was left to stir for 1 h.

Without a Catalyst (General Procedure 1.2):

A 2 mL glass HPLC vial was equipped with a small stirrer bar and was dosed with 600 µL stock solution **A**. To the vial was added triflic acid (2.0 eq, 11 µL, 0.123 mmol) which was left to stir for 1 h.

4.2 General Procedure for Reactions Run under N_2 (GENERAL PROCEDURE 2)

With a Catalyst (General Procedure 2.1):

A 2 mL glass HPLC vial was equipped with a small stirrer bar and ***catalyst*** (2 mol%) was added. The vial was dosed with 600 µL stock solution **A** before being sealed with a crimp top. The reaction mixture was purged (x 3) with N_2, then the vessel was sparged with N_2 for 5 min. To the vial was added triflic acid (2.0 eq, 11 µL, 0.123 mmol) which was left to stir for 1 h.

Without a Catalyst (General Procedure 2.2):

A 2 mL glass HPLC vial was equipped with a small stirrer bar and was dosed with 600 µL stock solution **A**. The vial was sealed with a crimp top and the reaction mixture was purged (x 3) with N_2, then the

vessel was sparged with N_2 for 5 min. To the vial was added triflic acid (2.0 eq, 11 µL, 0.123 mmol) which was left to stir for 1 h.

4.3 General Procedure for Reactions Run in the Dark (GENERAL PROCEDURE 3)

With a Catalyst (GENERAL PROCEDURE 3.1):

All dark reactions were prepared in a fume hood with no light on. A 2 mL glass amber vial was equipped with a small stirrer bar and ***catalyst*** (2 mol%) was added. The vial was dosed with 600 µL stock solution **A** and was placed in a blacked out TLC viewing box in a fume hood, with no light turned on, and left to stir for 10 min. To the vial was added (in the blacked out box) triflic acid (2.0 eq, 11 µL, 0.123 mmol) which was left to stir for 1 h.

Without a Catalyst (GENERAL PROCEDURE 3.2):

All dark reactions were prepared in a fume hood with no light on. A 2 mL glass amber vial was equipped with a small stirrer bar and was dosed with 600 µL stock solution **A**. The vial was placed in a blacked out TLC viewing box in a fume hood with no light turned on and left to stir for 10 min. To the vial was added (in the blacked out box) triflic acid (2.0 eq, 11 µL, 0.123 mmol) which was left to stir for 1 h.

5. Intermolecular Substrates

General Procedure 4.1 for C–H amination (with no additive)

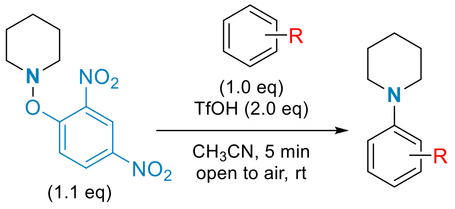

A 20 mL vial was charged with a stirrer bar, 1-(2,4-dinitrophenoxy)piperidine (**3**, 1.1 eq), **(hetero)arene** (1.0 eq) and CH_3CN (0.1 M), which was left to stir for 5 min at rt. To the vial was added triflic acid (2.0 eq) and the vial was stirred at rt for 5 min/until all of **3** had reacted. The reaction mixture was quenched with 1 M aqueous KOH solution (5 mL) before being diluted with water (5 mL) and CH_2Cl_2 (20 mL) and the layers were separated. The organic layer was collected and the aqueous layer was washed with further CH_2Cl_2 (20 mL). The organics were combined and extracted with 1 M aqueous KOH solution (10 mL) and water (10 mL). The organic layer was collected and filtered through a hydrophobic frit and concentrated *in vacuo*. Purification was conducted as defined in the experimental report of the respective product.

General Procedure 4.2 for C–H amination ($Ru(bpy)_3Cl_2$ as an additive)

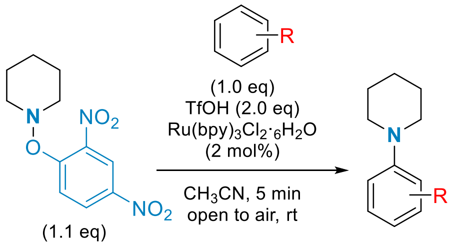

A 20 mL D-vial was charged with a stirrer bar, 1-(2,4-dinitrophenoxy)piperidine (**3**, 1.1 eq), $Ru(bpy)_3Cl_2·6H_2O$ (2 mol%), **(hetero)arene** (1.0 eq) and CH_3CN (0.1 M), which was left to stir for 5 min at rt. To the vial was added, triflic acid (2.0 eq) and the vial was stirred at rt for 5 min/until all of the **3** had reacted. The reaction mixture was quenched with 1 M aqueous KOH solution (5 mL) before being diluted with water (5 mL) and CH_2Cl_2 (20 mL) and the layers were separated. The organic layer was collected and the aqueous layer was washed with further CH_2Cl_2 (20 mL). The organics were combined and extracted with 1 M aqueous KOH solution (10 mL) and water (10 mL). The organic layer was collected and filtered through a hydrophobic frit and concentrated *in vacuo*. Purification was conducted as defined in the experimental report of the respective product.

1-(4-(*tert*-Butyl)phenyl)piperidine and 1-(3-(*tert*-butyl)phenyl)piperidine (**4' + 4"**)

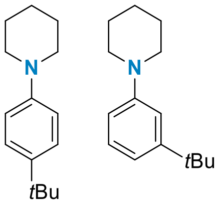

4' **4"**

This experiment followed General Procedure 4.1, using using *tert*-butylbenzene (63.5 µL, 0.410 mmol) and purification by a 5 g Biotage Sfar column, eluting with 0-40% EtOAc:heptane (10 column volumes). The relevant fractions were combined and concentrated *in vacuo* to yield the desired products as a yellow oil (20 mg, 22%). 1-(4-(*tert*-Butyl)phenyl)piperidine (**4'**) and 1-(3-(*tert*-butyl)phenyl)piperidine (**4"**) were isolated as a mixture of regioisomers (6.2:1, *para*:*meta*).

This experiment followed General Procedure 4.2, using *tert*-butylbenzene (70 µL, 0.452 mmol) and purification by a 5 g Biotage Sfar column, eluting with 0-40% EtOAc:heptane (10 column volumes). The relevant fractions were combined and concentrated *in vacuo* to yield the desired products as a mixture of regioisomers (**4'**:**4"** [6.7:1], 91.3 mg, 93%). The mixture of regioisomers was submitted for specialist purification eluting with 80% EtOH (+ 0.2% isopropylamine): 20% IPA(+ 0.2% isopropylamine) for 15 min. 1-(4-(*tert*-Butyl)phenyl)piperidine (**4'**) was isolated as a white solid (33 mg, 34%).

^{1}H NMR (400 MHz, $CDCl_3$) δ (ppm) = 7.31 - 7.27 (m, 2H), 6.93 - 6.88 (m, 2H), 3.18 - 3.09 (m, 4H), 1.73 (m, 4H), 1.62 - 1.56 (m, 2H), 1.30 (s, 9H); **^{13}C NMR** (101 MHz, $CDCl_3$) δ (ppm) = 150.0, 141.9, 125.8, 116.2, 50.9, 33.9, 31.5, 26.0, 24.4; **LCMS (ES+):** t_R = 0.68 min, $[M+H]^+$ 218.37; **HRMS (ESI) m/z**: $[M+H]^+$ Calcd for $C_{15}H_{24}N$ 218.1903, found 218.1900. Data were in accordance with previously reported literature.[4]

1-(3-(*tert*-Butyl)phenyl)piperidine (**4"**) was isolated as a yellow oil (13 mg, 13%).

^{1}H NMR (400 MHz, $CDCl_3$) δ (ppm) = 7.21 (t, *J* = 7.9 Hz, 1H), 7.03 (t, *J* = 2.2 Hz, 1H), 6.91 (ddd, *J* = 1.0, 2.0, 7.8 Hz, 1H), 6.79 (ddd, *J* = 1.0, 2.3, 7.9 Hz, 1H), 3.21 - 3.13 (m, 4H), 1.75 (quin, *J* = 5.8, 4H), 1.63 - 1.57 (m, 2H), 1.35 (s, 9H); **^{13}C NMR** (101 MHz, $CDCl_3$) δ (ppm) = 152.3, 151.9, 128.5, 116.8, 114.5, 113.7, 51.2, 34.8, 31.4, 26.1, 24.4; **LCMS** (ES+): t_R = 0.68 min, $[M+H]^+$ 218.38; **ν_{max} (neat)**: 3073, 2933, 2856, 2792, 1598, 1578, 1490, 1466, 1381, 1229; **HRMS (ESI) m/z**: $[M+H]^+$ Calcd for $C_{15}H_{24}N$ 218.1903, found 218.1900.

1-(4-Methoxyphenyl)piperidine and 1-(2-methoxyphenyl)piperidine (5′ + 5″)

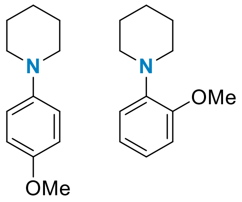

5' **5"**

This experiment followed General Procedure 4.1, using using anisole (45 µL, 0.414 mmol) and purification by a 5 g Biotage Sfar column, eluting with 0-40% EtOAc:heptane (10 column volumes). The relevant fractions were combined and concentrated *in vacuo* to yield the desired products as a yellow oil (50 mg, 56%). 1-(4-Methoxyphenyl)piperidine (**5′**) and 1-(2-methoxyphenyl)piperidine (**5″**) were isolated as a mixture of regioisomers (2:1, *para*:*ortho*).

This experiment followed General Procedure 4.2, using using anisole (45 µL, 0.414 mmol) and purification by a 5 g Biotage Sfar column, eluting with 0-40% EtOAc:heptane (10 column volumes). The relevant fractions were combined and concentrated *in vacuo* to yield the desired products **5′** and **5″** as a yellow oil (78 mg, 94%). 1-(4-Methoxyphenyl)piperidine (**5′**) and 1-(2-methoxyphenyl)piperidine (**5″**) were isolated as a mixture of regioisomers (1:1.2, *para*:*ortho*).

^{1}H NMR (400 MHz, $CDCl_3$): 7.02 - 6.81 (m, 8H, **5′** + **5″**: Ar**H**), 3.88 (s, 3H, **5″**: OC**H**$_3$), 3.78 (s, 3H, **5′**: OC**H**$_3$), 3.02 - 2.98 (m, 8H, **5′** + **5″**: 2 x NC**H**$_2$), 1.62 - 1.52 (m, 12H, **5′** + **5″**: 2 x NCH_2C**H**$_2$, 1 x NCH_2CH_2C**H**$_2$); **^{13}C NMR** (101 MHz, $CDCl_3$) δ (ppm) 153.6 (**5′**), 152.4 (**5″**), 146.9 (**5′**), 142.8 (**5″**), 122.5 (**5″**), 120.9 (**5″**), 118.8 (**5″**), 118.4 (**5″**), 114.4 (**5′**), 111.2 (**5″**), 55.6 (**5′**), 55.4 (**5″**), 52.4 (**5″**), 52.3 (**5′**), 26.3 (**5″**), 26.1 (**5′**), 24.5 (**5″**), 24.2 (**5′**). **LCMS (ES+):** t_R = 0.38 min, $[M+H]^+$ 192.36. Data were in accordance with previously reported literature.[1, 5]

N-(4-(Piperidin-1-yl)phenyl)acetamide (6a)

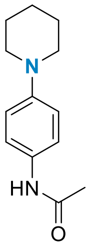

6a

A 20 mL vial was charged with a stirrer bar, 1-(2,4-dinitrophenoxy)piperidine (**3**) (1.0 eq, 100 mg, 0.374 mmol), acetanilide (1.1 eq, 55 mg, 0.412 mmol) and CH_3CN (0.1 M), which was left to stir for 5 min at rt. To the vial was added perchloric acid (2.0 eq, 64 µL, 0.748 mmol) and the mixture was stirred at rt

for 5 min/until all of **2** had reacted. The reaction mixture was quenched with 1 M aqueous KOH solution (5 mL) before being diluted with water (5 mL) and CH_2Cl_2 (20 mL) and the layers were separated. The organic layer was collected, and the aqueous layer was washed with further CH_2Cl_2 (20 mL). The organics were combined and extracted with 1 M aqueous KOH solution (10 mL) and water (10 mL). The organic layer was collected and filtered through a hydrophobic frit and concentrated *in vacuo*. The crude material was purified on a 5 g Biotage Sfar column, eluting with 0-100% EtOAc:heptane (10 column volumes). The relevant fractions were combined and concentrated *in vacuo* to yield *N*-(4-(piperidin-1-yl)phenyl)acetamide (**6a**) as an off-white solid (18 mg, 22%).

This experiment followed General Procedure 4.2, using acetanilide (54 mg, 0.400 mmol) and purification by a 5 g Biotage Sfar column, eluting with 0-100% EtOAc:heptane (10 column volumes). The relevant fractions were combined and concentrated *in vacuo* to yield *N*-(4-(piperidin-1-yl)phenyl)acetamide (**6a**) as an off-white solid (16 mg, 18%).

^{1}H NMR (400 MHz, $CDCl_3$) δ (ppm) 7.34 (d, *J* = 9.3 Hz, 2H), 7.18 (br s, 1H), 6.89 (d, *J* = 9.3 Hz, 2H), 3.12 - 3.07 (m, 4H), 2.14 (s, 3H), 1.71 (quin, *J* = 5.6 Hz, 4H), 1.60 - 1.53 (m, 2H); **^{13}C NMR** (101 MHz, $CDCl_3$) δ (ppm) 168.1, 149.4, 129.8, 121.5, 117.0, 51.1, 25.9, 24.3, 24.2. Data were in accordance with previously reported literature.[6]

1-(5-Bromo-2-methoxyphenyl)piperidine (**6b**)

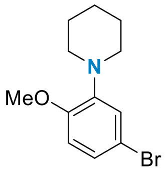

6b

This experiment followed General Procedure 4.1, using 1-bromo-4-methoxybenzene (50.1 µL, 0.400 mmol) and purification by a 5 g Biotage Sfar column, eluting with 0-40% EtOAc:heptane (10 column volumes). The relevant fractions were combined and concentrated *in vacuo* to yield 1-(5-bromo-2-methoxyphenyl)piperidine (**6b**) as a colourless oil (17.7 mg, 16%).

This experiment followed General Procedure 4.2, using 1-bromo-4-methoxybenzene (50.1 µL, 0.400 mmol) and purification by a 5 g Biotage Sfar column, eluting with 0-40% EtOAc:heptane (10 column volumes). The relevant fractions were combined and concentrated *in vacuo* to yield 1-(5-bromo-2-methoxyphenyl)piperidine (**6b**) as a colourless oil (65 mg, 61%).

^{1}H NMR (400 MHz, $CDCl_3$) δ (ppm) = 7.06 (dd, *J* = 2.4, 8.8 Hz, 1H), 7.02 (d, *J* = 2.4 Hz, 1H), 6.70 (d, *J* = 8.3 Hz, 1H), 3.84 (s, 3H), 3.00 - 2.93 (m, 4H), 1.79 - 1.71 (m, 4H), 1.61 - 1.53 (m, 2H); **^{13}C NMR** (101

MHz, $CDCl_3$) δ (ppm) = 151.6, 144.2, 124.8, 121.7, 113.2, 112.6, 55.6, 52.1, 26.2, 24.3.; **LCMS (ES+):** t_R = 0.76 min, $[M+H]^+$ 270.17; **HRMS (ESI) m/z**: $[M+H]^+$ Calcd for $C_{12}H_{17}{}^{79}BrNO$ 270.0488, found 270.0489. Data were in accordance with previously reported literature.[1]

1-(2-Methoxy-5-(4,4,5,5-tetramethyl-1,3,2-dioxaborolan-2-yl)phenyl)piperidine (6c)

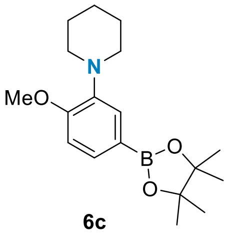

6c

This experiment followed General Procedure 4.1, using 2-(4-methoxyphenyl)-4,4,5,5-tetramethyl-1,3,2-dioxaborolane (94 mg, 0.400 mmol) and purification by a 5 g Biotage Sfar column, eluting with 0-40% EtOAc:heptane (10 column volumes). The relevant fractions were combined and concentrated *in vacuo* to yield 1-(5-bromo-2-methoxyphenyl)piperidine (**6c**) as a pale yellow oil (23.3 mg, 18%).

This experiment followed General Procedure 4.2, using 2-(4-methoxyphenyl)-4,4,5,5-tetramethyl-1,3,2-dioxaborolane (94 mg, 0.400 mmol) and purification by a 5 g Biotage Sfar column, eluting with 0-40% EtOAc:heptane (10 column volumes). The relevant fractions were combined and concentrated *in vacuo* to yield 1-(5-bromo-2-methoxyphenyl)piperidine (**6c**) as a pale yellow oil (70 mg, 55%).

^{1}H NMR (400 MHz, $CDCl_3$) δ (ppm) = 7.48 (dd, *J* = 1.5, 8.0 Hz, 1H), 7.39 (d, *J* = 1.5 Hz, 1H), 6.85 (d, *J* = 7.8 Hz, 1H), 3.89 (s, 3H), 3.04 - 2.98 (m, 4H), 1.76 (quin, *J* = 5.7, 4H), 1.60 - 1.54 (m, 2H), 1.34 (s, 12H); **^{13}C NMR** (101 MHz, $CDCl_3$) δ (ppm) = 155.2, 142.1, 130.3, 124.6, 110.4, 83.5, 55.4, 52.4, 26.9, 26.4, 24.9, 24.5; **LCMS (ES+):** t_R = 0.67 min, $[M+H]^+$ 318.32; **v_{max} (neat)**: 2976, 2931, 2852, 2799, 1594, 1510, 1411, 1351; **HRMS (ESI) m/z**: $[M+H]^+$ Calcd for $C_{18}H_{29}BNO_3$ 318.2235, found 318.2235.

1-(Naphthalen-1-yl)piperidine (6d)

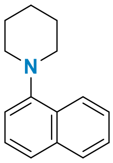

6d

This experiment followed General Procedure 4.1, using naphthalene (50 mg, 0.390 mmol) and purification by a 5 g Biotage Sfar column, eluting with 0-40% EtOAc:heptane (10 column volumes). The relevant fractions were combined and concentrated *in vacuo* to yield 1-(naphthalen-1-yl)piperidine (**6d**) as a yellow oil (76 mg, 84%).

This experiment followed General Procedure 4.2, using naphthalene (51.3 mg, 0.400 mmol) and purification by a 5 g Biotage Sfar column, eluting with 0-40% EtOAc:heptane (10 column volumes UMES). The relevant fractions were combined and concentrated *in vacuo* to yield 1-(naphthalen-1-yl)piperidine (**6d**) as a yellow oil (72 mg, 81%).

^{1}H NMR (400 MHz, $CDCl_3$) δ (ppm) = 8.22 (dd, *J* = 1.5, 8.3 Hz, 1H), 7.86 - 7.79 (m, 1H), 7.53 (d, *J* = 8.3 Hz, 1H), 7.51 - 7.44 (m, 2H), 7.43 - 7.37 (m, 1H), 7.08 (dd, *J* = 1.0, 7.3 Hz, 1H), 3.20 - 2.91 (m, 4H), 1.87 (quin, *J* = 5.6 Hz, 4H), 1.74 - 1.60 (m, 2H); **^{13}C NMR** (101 MHz, $CDCl_3$) δ (ppm) = 151.1, 134.7, 129.1, 128.3, 125.9, 125.6, 125.1, 123.8, 122.9, 114.4, 54.6, 26.7, 24.6; **LCMS (ES+):** t_R = 1.38 min, $[M+H]^+$ 212.37; **HRMS (ESI) m/z**: $[M+H]^+$ Calcd for $C_{15}H_{18}N$ 212.1434, found 212.1430. Data were in accordance with previously reported literature.[1]

1-(Benzo[*b*]thiophen-2-yl)piperidine (**6e**)

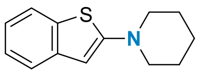

6e

This experiment followed General Procedure 4.1, using benzo[*b*]thiophene (57 mg, 0.400 mmol) and purification by a 5 g Biotage Sfar column, eluting with 0-40% EtOAc:heptane (10 column volumes). The relevant fractions were combined and concentrated *in vacuo* to yield 1-(benzo[b]thiophen-2-yl)piperidine (**6e**) as a pale yellow solid (31 mg, 36%).

This experiment followed General Procedure 4.2, using benzo[*b*]thiophene (57 mg, 0.400 mmol) and purification by a 5 g Biotage Sfar column, eluting with 0-40% EtOAc:heptane (10 column volumes). The relevant fractions were combined and concentrated *in vacuo* to yield 1-(benzo[b]thiophen-2-yl)piperidine (**6e**) as a pale yellow solid (80 mg, 94%).

^{1}H NMR (400 MHz, $CDCl_3$) δ (ppm) = 7.58 (dd, *J* = 1.0, 7.8 Hz, 1H), 7.43 (d, *J* = 7.8 Hz, 1H), 7.22 (dt, *J* = 1.0, 7.6 Hz, 1H), 7.06 (ddd, *J* = 1.5, 7.1, 8.1 Hz, 1H), 6.19 - 6.14 (m, 1H), 3.29 - 3.23 (m, 4H), 1.79 - 1.71 (m, 4H), 1.66 - 1.57 (m, 2H); **^{13}C NMR** (101 MHz, $CDCl_3$) δ (ppm) = 158.5, 140.9, 132.6, 124.3, 121.4, 120.9, 120.5, 98.4, 52.0, 25.2, 23.9; **LCMS (ES+):** t_R = 1.42 min, $[M+H]^+$ 218.25; **HRMS (ESI) m/z**: $[M+H]^+$ Calcd for $C_{13}H_{16}NS$ 218.0998, found 218.0996. Data were in accordance with previously reported literature.[7]

6. Intramolecular Amination

6.1 Synthesis of Precursors:

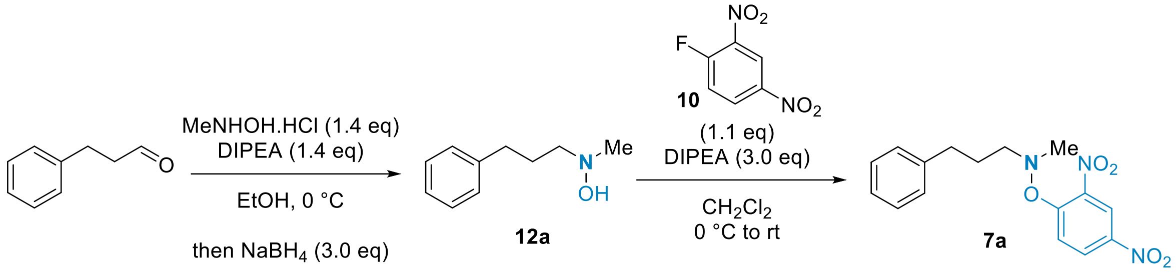

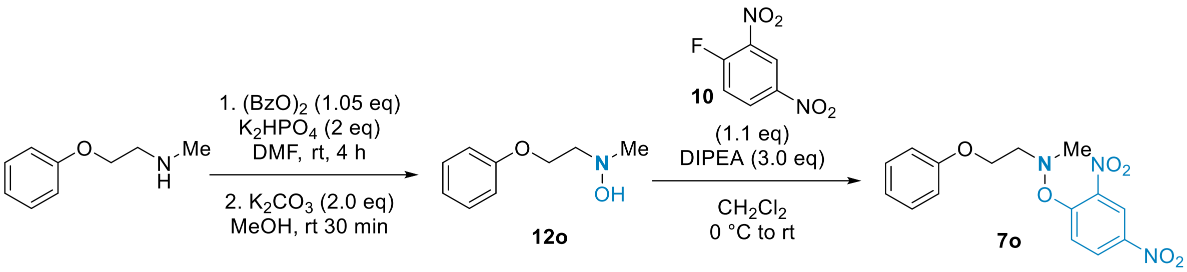

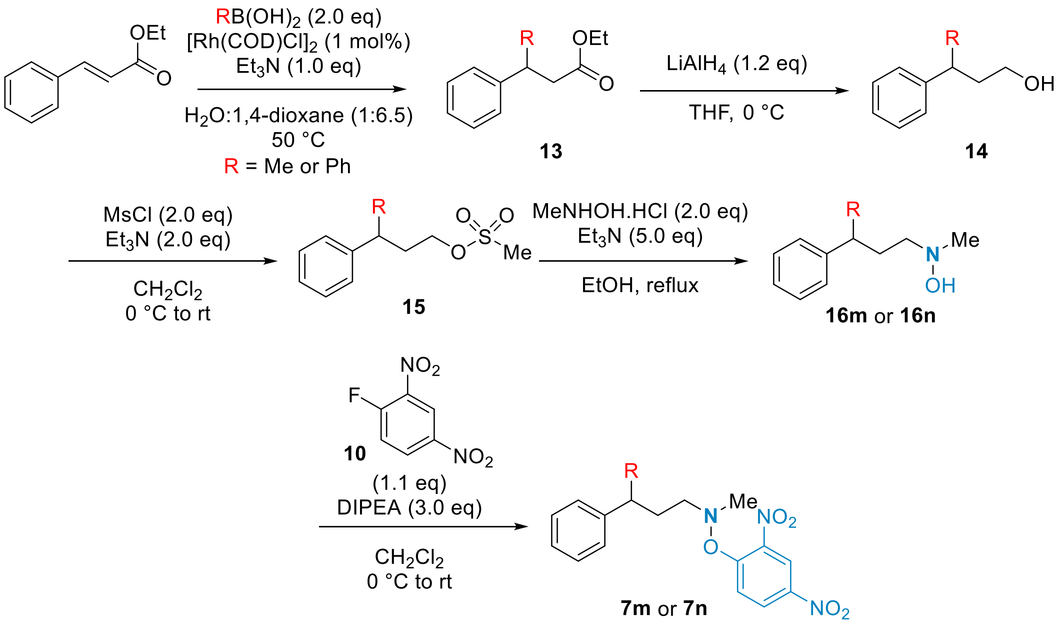

6.2 General Procedure 5 for the optimisation

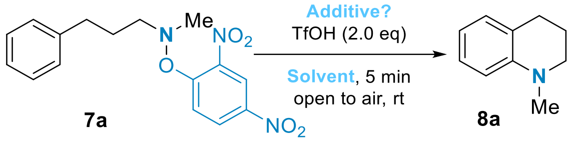

To a vial containing **7a** (1.0 eq), $Ru(bpy)_3Cl_2 \cdot 6H_2O$ (2 mol%) and **solvent** (0.1 M) was added TfOH (2.0 eq) at room temperature. The reaction mixture was left to stir for 5 min.

For isolated yields, the reaction was quenched with 1 M aqueous KOH solution and extracted with CH_2Cl_2 (x3). The organics were combined, filtered through a hydrophobic frit and concentrated *in vacuo* to give a red oil. The crude sample was loaded onto a 5 g Sfar Biotage column, eluting with 0-30% EtOAc:heptane (10 column volumes). The relevant fractions were combined and concentrated *in vacuo* to give **8a** as a yellow oil.

Entry	[$Ru(bpy)_3Cl_2$]	Solvent	8a[a] (%)
**1**	×	HFIP	64%
**2**	✓	**CH_3CN**	**63%**
**3**	✓	HFIP	60%
**4**	✓	EtOAc	51%

[a] Isolated yields

Entry	[$Ru(bpy)_3Cl_2$] (2 mol%)	N_2 atmosphere	Addition of Acid	8a[a] (%)
**1**	×	×	✓	65%
**2**	✓	×	✓	**77%**
**3**	✓	✓	✓	45%
**4**	×	✓	✓	37%
**5**	×	×	×	0%
**6**	✓	×	×	0%

[a] ^1H NMR yields were calculated in reference to 1,3,5-trimethoxybenzene as an internal standard.

6.3 Hydroxylamines

General Procedure 6

A round-bottomed flask was charged with *N*-methylhydroxylamine hydrochloride (1.4 eq), EtOH (15 mL) and DIPEA (1.4 eq) before being cooled to 0 °C. To the vial was added aldehyde (1.0 eq) in a solution of EtOH (5 mL) at 0 °C. Once formation of the imine was observed, sodium borohydride (3 eq) was added portionwise at 0 °C and the mixture was left to stir until the reaction had reached completion by LCMS. The reaction mixture was concentrated *in vacuo* before being diluted with brine and EtOAc. The aqueous layer was washed with EtOAc (x3). The organics were combined, filtered through a hydrophobic frit and concentrated *in vacuo* to give a the crude hydroxylamine. Purification was conducted as defined in the experimental report of the respective product.

N-(3-Phenylpropyl)hydroxylamine (**12a**)

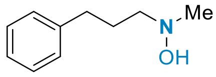

12a

This experiment followed General Procedure 6, using *N*-methylhydroxylamine hydrochloride (1.77 g, 21.2 mmol), EtOH (10 mL) and DIPEA (3.71 mL, 21.2 mmol) with 3-phenylpropanal (2.0 g, 15.2 mmol) in EtOH (10 mL) and sodium borohydride (1.15 g, 30.4 mmol). The crude material was purified on a 50 g Sfar Biotage column, eluting with 0-50% EtOAc:heptane for 10 column volumes. The relevant fractions were combined and concentrated *in vacuo* to give a *N*-methyl-*N*-(3-phenylpropyl)hydroxylamine (**12a**) as a colourless oil (1.88 g, 75%).

^{1}H NMR (400 MHz, $CDCl_3$, 303 K) δ (ppm) = 7.33 - 7.28 (m, 2H), 7.24 - 7.19 (m, 3H), 2.76 - 2.67 (m, 7H), 1.98 (quin, *J* = 7.7 Hz, 2H); **^{13}C NMR** (101 MHz, $CDCl_3$, 303 K) δ (ppm) = 141.9, 128.4, 128.3, 125.8, 61.6, 48.7, 33.4, 28.8; **LCMS (ES+)**: t_R = 0.44 min, $[M+H]^+$ 166.17; **ν_{max} (neat)**: 3203, 3025, 2951, 2851, 1602, 1495, 1453 cm^{-1}; **HRMS (ESI) m/z**: $[M+H]^+$ Calcd for $C_{10}H_{16}NO$ 166.1226, found 166.1219.

N-Methyl-*N*-(3-(*p*-tolyl)propyl)hydroxylamine (**12b**)

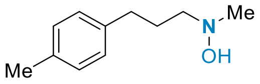

12b

This experiment followed General Procedure 6, using *N*-methylhydroxylamine hydrochloride (789 mg, 9.45 mmol), EtOH (15 mL) and DIPEA (1.65 mL, 9.45 mmol) with 3-(*p*-tolyl)propanal (1 g, 6.75 mmol) in EtOH (5 mL) and sodium borohydride (766 mg, 15.8 mmol). The crude material was purified on a 50 g Sfar Biotage column, eluting with 0-50% EtOAc:heptane for 10 column volumes. The relevant

fractions were combined and concentrated *in vacuo* to give a *N*-methyl-*N*-(3-(*p*-tolyl)propyl)hydroxylamine (**12b**) as a white solid (982 mg, 81%).

Mp: 71-72 °C; **^{1}H NMR** (400 MHz, $CDCl_3$, 303 K) δ (ppm) = 7.14 - 7.07 (m, 4H), 2.71 - 2.61 (m, 7H), 2.34 (s, 3H), 1.92 (quin, *J* = 7.5 Hz, 2H); **^{13}C NMR** (101 MHz, $CDCl_3$, 303 K) δ (ppm) = 138.9, 135.2, 129.0, 128.3, 61.7, 48.7, 33.0, 29.0, 21.0; **LCMS (ES+)**: t_R = 0.56 min, $[M+H]^+$ 180.30; **ν_{max} (neat)**: 3177, 2931, 2838, 1906, 1515, 1437 cm^{-1}; **HRMS (ESI) m/z**: $[M+H]^+$ Calcd for $C_{11}H_{18}NO$ 180.1383, found 180.1379.

N-(3-(4-(*tert*-Butyl)phenyl)propyl)-*N*-methylhydroxylamine (**12c**)

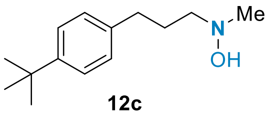

12c

This experiment followed General Procedure 6, using *N*-methylhydroxylamine hydrochloride (614 mg, 7.36 mmol), EtOH (15 mL) and DIPEA (1.29 mL, 7.36 mmol) with 3-(4-(*tert*-butyl)phenyl)propanal (1 g, 5.26 mmol) in EtOH (5 mL) and sodium borohydride (597 mg, 15.8 mmol). The crude material was purified on a 50 g Sfar Biotage column, eluting with 0-40% EtOAc:heptane for 10 column volumes. The relevant fractions were combined and concentrated *in vacuo* to give *N*-(3-(4-(*tert*-butyl)phenyl)propyl)-*N*-methylhydroxylamine (**12c**) as a colourless oil (769 mg, 66%).

^{1}H NMR (400 MHz, $CDCl_3$, 303 K) δ (ppm) = 7.33 (d, *J* = 8.3 Hz, 2H), 7.15 (d, *J* = 8.8 Hz, 2H), 2.72 - 2.64 (m, 7H), 1.94 (quin, *J* = 7.6 Hz, 2H), 1.34 (s, 9H); **^{13}C NMR** (101 MHz, $CDCl_3$, 300 K) δ (ppm) = 148.6, 138.8, 128.0, 125.2, 61.7, 48.7, 34.4, 32.8, 31.4, 28.8; **LCMS (ES+):** t_R = 0.73 min, $[M+H]^+$ 222.30; **ν_{max} (neat)**: 3202, 2955, 2865, 2908, 1510, 1461, 1362; **HRMS (ESI) m/z**: $[M+H]^+$ Calcd for $C_{14}H_{24}NO$ 222.1852, found 222.1848.

N-(3-([1,1'-Biphenyl]-4-yl)propyl)-*N*-methylhydroxylamine (**12d**)

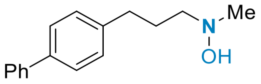

12d

This experiment followed General Procedure 6, using *N*-methylhydroxylamine hydrochloride (139 mg, 1.66 mmol), EtOH (5 mL) and DIPEA (0.29 mL, 1.66 mmol) with 3-([1,1'-biphenyl]-4-yl)propanal (250 mg, 1.19 mmol) in EtOH (2 mL) and sodium borohydride (135 mg, 3.57 mmol). The crude material was purified on a 10 g Sfar Biotage column, eluting with 30-50% EtOAc:heptane for 10 column volumes. The relevant fractions were combined and concentrated *in vacuo* to give *N*-(3-([1,1'-biphenyl]-4-yl)propyl)-*N*-methylhydroxylamine (**12d**) as a colourless oil (280 mg, 98%).

^{1}H NMR (400 MHz, $CDCl_3$, 303 K) δ (ppm) = 7.64 - 7.59 (m, 2H), 7.57 - 7.52 (m, 2H), 7.49 - 7.43 (m, 2H), 7.39 - 7.33 (m, 1H), 7.31 - 7.27 (m, 2H), 2.78 - 2.71 (m, 4H), 2.69 (s, 3H), 2.01 (quin, *J* = 7.5 Hz, 2H); **^{13}C**

NMR (101 MHz, $CDCl_3$, 303 K) δ (ppm) = 141.1, 141.0, 138.8, 128.8, 128.7, 127.1, 127.0, 127.0, 61.6, 48.8, 33.1, 28.8; **LCMS (ES+):** t_R = 0.70 min, $[M+H]^+$ 242.37; **ν_{max} (neat)**: 3170, 3034, 2986, 2868, 1599, 1468 cm^{-1}; **HRMS (ESI) m/z**: $[M+H]^+$ Calcd for $C_{16}H_{20}NO$ 242.1539, found 242.1536.

N-(3-(4-Methoxyphenyl)propyl)-*N*-methylhydroxylamine (**12e**)

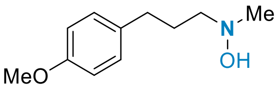

12e

This experiment followed General Procedure 6, using *N*-methylhydroxylamine hydrochloride (712 mg, 8.53 mmol), EtOH (10 mL) and DIPEA (1.49 mL, 8.53 mmol) with 3-(4-methoxyphenyl)propanal (1 g, 6.09 mmol) in EtOH (5 mL) and sodium borohydride (691 mg, 18.27 mmol). The crude material was purified on a 10 g Sfar Biotage column, eluting with 30-50% EtOAc:heptane for 10 column volumes. The relevant fractions were combined and concentrated *in vacuo* to give *N*-(3-(4-methoxyphenyl)propyl)-*N*-methylhydroxylamine (**12e**) as a white solid (780 mg, 66%).

Mp: 57-60 °C; **^{1}H NMR** (400 MHz, $CDCl_3$, 303 K) δ (ppm) = 7.12 (d, *J* = 8.8 Hz, 2H), 6.85 (d, *J* = 8.8 Hz, 2H), 3.81 (s, 3H), 2.70 - 2.58 (m, 7H), 1.91 (quin, *J* = 7.6 Hz, 2H); **^{13}C NMR** (101 MHz, $CDCl_3$, 303 K) δ (ppm) = 157.8, 134.0, 129.2, 113.8, 61.6, 55.3, 48.7, 32.5, 29.1; **LCMS (ES+):** t_R = 0.45 min, $[M+H]^+$ 196.33; **ν_{max} (neat)**: 3197, 3000, 2946, 2846, 1611, 1510, 1241; **HRMS (ESI) m/z**: $[M+H]^+$ Calcd for $C_{11}H_{18}NO_2$ 196.1332, found 196.1329.

N-(3-(4-Bromophenyl)propyl)-*N*-methylhydroxylamine (**12f**)

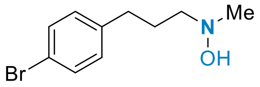

12f

This experiment followed General Procedure 6, using *N*-methylhydroxylamine hydrochloride (274 mg, 3.29 mmol), EtOH (5 mL) and DIPEA (0.57 mL, 3.29 mmol) with 3-(4-bromophenyl)propanal (500 mg, 2.35 mmol) in EtOH (5 mL) and sodium borohydride (266 mg, 7.04 mmol). The crude material was purified on a 10 g Sfar Biotage column, eluting with 25-45% EtOAc:heptane for 10 column volumes. The relevant fractions were combined and concentrated *in vacuo* to give *N*-(3-(4-bromophenyl)propyl)-*N*-methylhydroxylamine (**12f**) as a colourless gum (475 mg, 83%).

^{1}H NMR (400 MHz, $CDCl_3$, 303 K) δ (ppm) = 7.45 - 7.37 (m, 2H), 7.11 - 7.04 (m, 2H), 2.73 - 2.60 (m, 7H), 1.90 (quin, *J* = 7.5 Hz, 2H). **^{13}C NMR** (101 MHz, $CDCl_3$, 303 K) δ (ppm) = 140.6, 131.5, 130.1, 119.7, 61.0, 48.3, 32.7, 28.1; **LCMS (ES+):** t_R = 0.57 min, $[M+H]^+$ 244.19; **ν_{max} (neat)**: 3332, 2928, 2861, 1898, 1590, 1486; cm^{-1}; **HRMS (ESI) m/z**: $[M+H]^+$ Calcd for $C_{10}H_{15}{}^{79}BrNO$ 244.0332, found 244.0329.

N-(3-(4-Chlorophenyl)propyl)-N-methylhydroxylamine (**12g**)

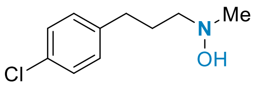

12g

This experiment followed General Procedure 6, using *N*-methylhydroxylamine hydrochloride (173 mg, 2.01 mmol), EtOH (5 mL) and DIPEA (0.36 mL, 2.01 mmol) with 3-(4-chlorophenyl)propanal (250 mg, 1.48 mmol) in EtOH (2 mL) and sodium borohydride (168 mg, 4.45 mmol). The crude material was purified on a 10 g Sfar Biotage column, eluting with 35-55% EtOAc:heptane for 10 column volumes. The relevant fractions were combined and concentrated *in vacuo* to give *N*-(3-(4-chlorophenyl)propyl)-*N*-methylhydroxylamine (**12g**) as a white solid (148 mg, 50%).

Mp: 71-74 °C; **^{1}H NMR** (400 MHz, $CDCl_3$, 303 K) δ (ppm) = 7.26 (d, *J* = 8.3 Hz, 2H), 7.13 (d, *J* = 8.3 Hz, 2H), 5.90 (br s, 1H), 2.72 - 2.60 (m, 7H), 1.97 - 1.84 (m, 2H); **^{13}C NMR** (101 MHz, $CDCl_3$, 303 K) δ (ppm) = 140.4, 131.5, 129.7, 128.4, 61.4, 48.9, 32.7, 28.8; **LCMS (ES+):** t_R = 0.56 min, $[M+H]^+$ 200.22; **ν_{max} (neat)**: 3145, 2920, 2862, 1898, 1596, 1492 cm^{-1}; **HRMS (ESI) m/z**: $[M+H]^+$ Calcd for $C_{10}H_{15}{}^{35}ClNO$ 200.0837, found 200.0838.

N-(3-(3-Methoxyphenyl)propyl)-N-methylhydroxylamine (**12h**)

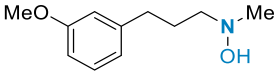

12h

This experiment followed General Procedure 6, using *N*-methylhydroxylamine hydrochloride (184 mg, 2.20 mmol), EtOH (10 mL) and DIPEA (0.38 mL, 2.20 mmol) with 3-(3-methoxyphenyl)propanal (258 mg, 1.57 mmol) in EtOH (5 mL) and sodium borohydride (119 mg, 3.14 mmol). The crude material was purified on a 10 g Sfar Biotage column, eluting with 25-45% EtOAc:heptane for 10 column volumes. The relevant fractions were combined and concentrated *in vacuo* to give *N*-(3-(3-methoxyphenyl)propyl)-*N*-methylhydroxylamine (**12h**) as a colourless oil (179 mg, 58%).

^{1}H NMR (400 MHz, $CDCl_3$, 303 K) δ (ppm) = 7.25 - 7.17 (m, 1H), 6.85 - 6.73 (m, 3H), 3.82 (s, 3H), 2.73 - 2.62 (m, 7H), 1.94 (quin, *J* = 7.6 Hz, 2H); **^{13}C NMR** (101 MHz, $CDCl_3$, 303 K) δ (ppm) = 159.7, 143.6, 129.3, 120.8, 114.2, 111.1, 61.6, 55.1, 48.7, 33.5, 28.7; **LCMS (ES+):** t_R = 0.45 min, $[M+H]^+$ 196.18; **ν_{max} (neat)**: 3202, 2951, 2834, 1600, 1583, 1453, 1259 cm^{-1}; **HRMS (ESI) m/z**: $[M+H]^+$ Calcd for $C_{11}H_{18}NO_2$ 196.1332, found 196.1326.

N-(3-(3-Bromophenyl)propyl)-*N*-methylhydroxylamine (**12i**)

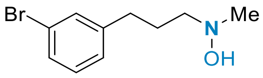

12i

This experiment followed General Procedure 6, using *N*-methylhydroxylamine hydrochloride (137 mg, 1.64 mmol), EtOH (5 mL) and DIPEA (0.287 mL, 1.64 mmol) with 3-(3-bromophenyl)propanal (250 mg, 1.17 mmol) in EtOH (2 mL) and sodium borohydride (133 mg, 3.52 mmol). The reaction mixture was worked up and the organics were concentrated *in vacuo* to give *N*-(3-(3-bromophenyl)propyl)-*N*-methylhydroxylamine (**12i**) as a colourless oil (217 mg, 76%). No further purification was carried out.

^{1}H NMR (400 MHz, $CDCl_3$, 303 K) δ (ppm) = 7.39 - 7.31 (m, 2H), 7.20 - 7.08 (m, 2H), 2.72 - 2.60 (m, 7H), 1.92 (quin, *J* = 7.7 Hz, 2H); ^{13}C NMR (101 MHz, $CDCl_3$, 300 K) δ (ppm) = 144.3, 131.5, 129.9, 129.0, 127.0, 122.4, 61.3, 48.8, 33.1, 28.6; **LCMS (ES+)**: t_R = 0.59 min, $[M+H]^+$ 244.20; **ν_{max} (neat)**: 3245, 2949, 2861, 1594, 1566, 1472 cm^{-1}; **HRMS (ESI) m/z**: $[M+H]^+$ Calcd for $C_{10}H_{15}{}^{79}BrNO$ 244.0332, found 244.0345.

N-Methyl-*N*-(3-(*o*-tolyl)propyl)hydroxylamine (**12j**)

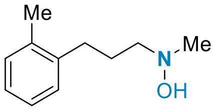

12j

This experiment followed General Procedure 6, using *N*-methylhydroxylamine hydrochloride (789 mg, 9.45 mmol), EtOH (15 mL) and DIPEA (1.65 mL, 9.45 mmol) with 3-(*o*-tolyl)propanal (1 g, 3.29 mmol) in EtOH (5 mL) and sodium borohydride (766 mg, 20.24 mmol). The crude material was purified on a 50 g Sfar Biotage column, eluting with 0-50% EtOAc:heptane for 10 column volumes. The relevant fractions were combined and concentrated *in vacuo* to give *N*-methyl-*N*-(3-(*o*-tolyl)propyl)hydroxylamine (**12j**) as a colourless oil (972 mg, 80%).

^{1}H NMR (400 MHz, $CDCl_3$, 303 K) δ (ppm) = 7.19 - 7.10 (m, 4H), 2.75 - 2.65 (m, 7H), 2.33 (s, 3H), 1.90 (quin, *J* = 7.6 Hz, 2H); **^{13}C NMR** (101 MHz, $CDCl_3$, 303 K) δ (ppm) = 140.1, 135.9, 130.2, 128.7, 125.9, 125.9, 61.9, 48.7, 30.7, 27.7, 19.3; **LCMS (ES+)**: t_R = 0.51 min, $[M+H]^+$ 180.27; **ν_{max} (neat)**: 3203, 3060, 2952, 2866, 1604, 1492, 1458 cm^{-1}; **HRMS (ESI) m/z**: $[M+H]^+$ Calcd for $C_{11}H_{18}NO$ 180.1383, found 180.1380.

N-(3-(2-Fluorophenyl)propyl)-*N*-methylhydroxylamine (**12k**)

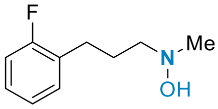

12k

This experiment followed General Procedure 6, using *N*-methylhydroxylamine hydrochloride (384 mg, 4.60 mmol), EtOH (15 mL) and DIPEA (0.80 mL, 4.60 mmol) with 3-(2-fluorophenyl)propanal (1 g, 3.29 mmol) in EtOH (5 mL) and sodium borohydride (373 mg, 9.86 mmol). The crude material was purified on a 50 g Sfar Biotage column, eluting with 0-50% EtOAc:heptane for 10 column volumes. The relevant fractions were combined and concentrated *in vacuo* to give *N*-(3-(2-fluorophenyl)propyl)-*N*-methylhydroxylamine (**12k**) as a colourless oil (547 mg, 91%).

^{1}H NMR (400 MHz, $CDCl_3$, 303 K) δ (ppm) = 7.24 - 7.14 (m, 2H), 7.12 - 6.98 (m, 2H), 2.76 - 2.64 (m, 7H), 1.93 (quin, *J* = 7.5 Hz, 2H); **^{19}F NMR** (376 MHz, $CDCl_3$, 303 K) δ (ppm) = −118.79 (s, 1F); **^{13}C NMR** (101 MHz, $CDCl_3$, 303 K) δ = 161.2 (d, *J* = 244.3 Hz), 130.6 (d, *J* = 5.1 Hz), 128.8 (d, *J* = 16.1 Hz), 127.5 (d, *J* = 8.1 Hz), 123.9 (d, *J* = 3.7 Hz), 115.2 (d, *J* = 22.0 Hz), 61.5, 48.7, 27.7, 26.6 (d, *J* = 2.2 Hz); **LCMS (ES+)**: t_R = 0.46 min, $[M+H]^+$ 184.21; **ν_{max} (neat)**: 3206, 2955, 2866, 1583, 1490, 1455, 1227; **HRMS (ESI) m/z**: $[M+H]^+$ Calcd for $C_{10}H_{15}FNO$ 184.1132, found 184.1129.

N-(3-(2-Bromophenyl)propyl)-*N*-methylhydroxylamine (**12l**)

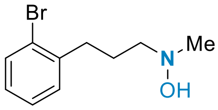

12l

This experiment followed General Procedure 6, using *N*-methylhydroxylamine hydrochloride (277 mg, 3.32 mmol), EtOH (8 mL) and DIPEA (0.58 mL, 3.32 mmol) with 3-(2-bromophenyl)propanal (532 mg, 2.37 mmol) in EtOH (3 mL) and sodium borohydride (269 mg, 7.12 mmol). The crude material was purified on a 50 g Sfar Biotage column, eluting with 0-50% EtOAc:heptane for 10 column volumes. The relevant fractions were combined and concentrated *in vacuo* to give *N*-(3-(4-bromophenyl)propyl)-*N*-methylhydroxylamine (**12l**) as a colourless oil (546 mg, 94%).

^{1}H NMR (400 MHz, $CDCl_3$, 303 K) δ (ppm) = 7.55 (d, *J* = 7.8 Hz, 1H), 7.26 - 7.22 (m, 2H), 7.12 - 7.03 (m, 1H), 6.05 (br s, 1H), 2.85 - 2.79 (m, 2H), 2.71 (t, *J* = 7.1 Hz, 2H), 2.68 (s, 3H), 1.98 - 1.88 (m, 2H); **^{13}C NMR** (101 MHz, $CDCl_3$, 303 K) δ (ppm) = 141.3, 132.8, 130.3, 127.6, 127.4, 124.5, 61.5, 48.8, 33.7, 27.5; **LCMS (ES+)**: t_R = 0.55 min, $[M+H]^+$ 244.22; **ν_{max} (neat)**: 3110, 2956, 2849, 2800, 1565, 1470; **HRMS (ESI) m/z**: $[M+H]^+$ Calcd for $C_{10}H_{15}{}^{79}BrNO$ 244.0332, found 244.0327.

Oxidation to the Hydroxylamine[1]

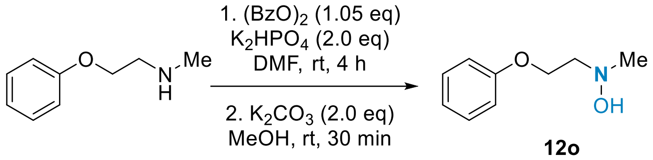

12o

N-Methyl-*N*-(2-phenoxyethyl)hydroxylamine (**12o**)

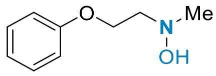

12o

To a solution of *N*-methyl-2-phenoxyethan-1-amine (1.0 eq, 200 mg, 1.22 mmol) in DMF (5 mL) were added benzoyl peroxide (1.05 eq, 413 mg, 1.28 mmol) and K_2HPO_4 (2.0 eq, 424 mg, 2.43 mmol) and the reaction was stirred at rt for 4 h. The reaction mixture was diluted with aqueous saturated sodium sulfite solution (10 mL) and extracted with EtOAc (3 x 20 mL). The organic layers were combined and washed with aqueous saturated NH_4Cl solution (3 x 40 mL). The organic layer was filtered through a hydrophobic frit and concentrated *in vacuo* to give the crude *O*-benzoyl hydroxylamine. The crude product was purified on a 25 g Sfar Biotage column, eluting with 0-40% EtOAc:heptane for 10 column volumes. The relevant fractions were combined and concentrated *in vacuo* to give a colourless oil. The *O*-benzoyl hydroxylamine was diluted with MeOH (4 mL) and K_2CO_3 (2.0 eq, 336 mg, 2.43 mmol) was added and the reaction mixture was stirred for 30 min at rt. The reaction mixture was concentrated *in vacuo* and diluted with water (15 mL) and EtOAc (20 mL). The aqueous layer was washed with EtOAc (3 x 20 mL). The organics were combined, filtered through a hydrophobic frit and concentrated *in vacuo* to give *N*-methyl-*N*-(2-phenoxyethyl)hydroxylamine (**12o**) as a white solid (140 mg, 69%).

Mp: 52-54 °C; **^{1}H NMR** (400 MHz, $CDCl_3$, 303 K) δ (ppm) = 7.32 - 7.25 (m, 2H), 6.97 (tt, *J* = 1.0, 7.3 Hz, 1H), 6.93 - 6.88 (m, 2H), 4.16 (br t, *J* = 5.1 Hz, 2H), 3.09 (t, *J* = 5.4 Hz, 2H), 2.77 (s, 3H); **^{13}C NMR** (101 MHz, $CDCl_3$, 303 K) δ (ppm) = 158.5, 129.5, 120.9, 114.6, 64.4, 60.5, 48.8; **LCMS (ES+)**: t_R = 0.38 min, $[M+H]^+$ 168.16; **ν_{max} (neat)**: 3208, 3065, 2962, 2867, 1937, 1599, 1500, 1242 cm^{-1}; **HRMS (ESI) m/z**: $[M+H]^+$ Calcd for $C_9H_{14}NO_2$ 168.1019, found 168.1029.

1,4-Hayashi Couplings[8]

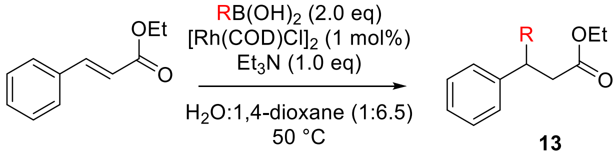

13

Ethyl 3-phenylbutanoate (**13m**)

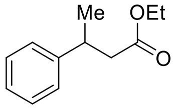

13m

To a microwave vial containing phenylboronic acid (2.0 eq, 0.732 g, 6 mmol) and $[Rh(COD)Cl]_2$ (1 mol%, 14.5 mg, 0.029 mmol) were added degassed 1,4-dioxane (7.8 mL) and water (1.2 mL). To the vial were added ethyl crotonate (1.0 eq, 0.389 mL, 3 mmol) and degassed triethylamine (1.0 eq, 0.422 mL, 3 mmol). The reaction mixture was heated to 50 °C and stirred for 1 h. The reaction mixture was concentrated *in vacuo* and purified on a 10 g Sfar Biotage column, eluting with 0-20% EtOAc:heptane for 10 column volumes. The relevant fractions were combined and concentrated *in vacuo* to give ethyl 3-phenylbutanoate (**13m**) as a yellow oil (434 mg, 75%).

^{1}H NMR (400 MHz, $CDCl_3$, 303 K) δ (ppm) = 7.36 - 7.29 (m, 2H), 7.27 - 7.19 (m, 3H), 4.10 (q, *J* = 7.3 Hz, 2H), 3.31 (sxt, *J* = 7.2 Hz, 1H), 2.64 (dd, *J* = 6.8, 15.0 Hz, 1H), 2.56 (dd, *J* = 8.0, 15.0 Hz, 1H), 1.33 (d, *J* = 6.8 Hz, 3H), 1.21 (t, *J* = 7.1 Hz, 3H); **^{13}C NMR** (101 MHz, $CDCl_3$, 303 K) δ (ppm) = 172.4, 145.8, 128.5, 126.8, 126.4, 60.2, 43.0, 36.5, 21.8, 14.2; **LCMS (ES+):** t_R = 1.19 min, $[M+H]^+$ 193.28. Data were in accordance with previously reported literature.[9]

Ethyl 3,3-diphenylpropanoate (**13n**)

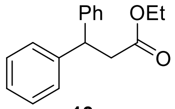

13n

To a microwave vial containing phenylboronic acid (2.0 eq, 0.503 g, 4.1 mmol) and $[Rh(COD)Cl]_2$ (1 mol%, 10 mg, 0.02 mmol) were added degassed 1,4-dioxane (5.2 mL) and water (0.8 mL). To the vial were added ethyl cinnamate (1.0 eq, 0.347 mL, 2.1 mmol) and degassed triethylamine (1.0 eq, 0.29 mL, 2 mmol). The reaction mixture was heated to 50 °C and stirred for 4.5 h. The reaction mixture was concentrated *in vacuo* and purified on a 10 g Sfar Biotage column, eluting with 0-20% EtOAc:heptane for 10 column volumes. The relevant fractions were combined and concentrated *in vacuo* to give ethyl 3,3-diphenylpropanoate (**13n**) as a yellow oil (434 mg, 75%).

^{1}H NMR (400 MHz, $CDCl_3$, 303 K) δ (ppm) = 7.33 - 7.25 (m, 8H), 7.24 - 7.18 (m, 2H), 4.58 (t, *J* = 7.8 Hz, 1H), 4.06 (q, *J* = 6.9 Hz, 2H), 3.08 (d, *J* = 7.8 Hz, 2H), 1.13 (t, *J* = 7.2 Hz, 3H); **^{13}C NMR** (101 MHz, $CDCl_3$, 303 K) δ (ppm) = 171.8, 143.5, 128.5, 127.7, 126.5, 60.4, 47.1, 40.9, 14.1; **LCMS (ES+):** t_R = 1.29 min, $[M+H]^+$ 255.21. Data were in accordance with previously reported literature.[9]

Reduction of Esters

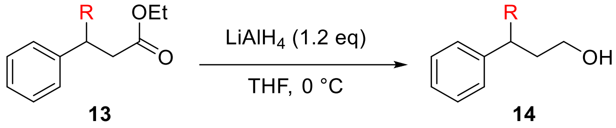

3-Phenylbutan-1-ol (**14m**)

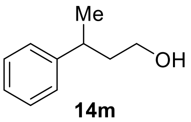

14m

To a round-bottomed flask was added ethyl 3-phenylbutanoate (**13m**, 1.0 eq, 430 mg, 1.77 mmol) and the vessel was purged with N_2 (x 3) and THF (3 mL, 0.59 M) was added. The mixture was cooled to 0 °C in an ice-bath then, $LiAlH_4$ [2 M in THF] (1.2 eq, 1.06 mL, 2.12 mmol) was added and the reaction mixture was stirred for 10 min. The reaction mixture was quenched with the slow addition of water (1 mL) at 0 °C and was concentrated *in vacuo* to give a black oil. The crude was diluted with water (10 mL), saturated aqueous solution of Rochelle salt (20 mL) and EtOAc (40 mL). The aqueous layer was extracted with EtOAc (3 x 40 mL). The organics were combined, filtered through a hydrophobic frit and concentrated *in vacuo* to give 3-phenylbutan-1-ol (**14m**) as a colourless oil (271 mg, >99%).

^{1}H NMR (400 MHz, $CDCl_3$, 303 K) δ (ppm) = 7.36 - 7.30 (m, 2H), 7.27 - 7.18 (m, 3H), 3.66 - 3.52 (m, 2H), 2.92 (sxt, *J* = 7.1 Hz, 1H), 1.92 - 1.86 (m, 2H), 1.31 (d, *J* = 7.3 Hz, 3H), 1.15 (t, *J* = 5.4 Hz, 1H); **^{13}C NMR** (101 MHz, $CDCl_3$, 303 K) δ (ppm) = 146.8, 128.5, 127.0, 126.1, 61.3, 41.0, 36.5, 22.4; **LCMS (ES+):** t_R = 0.88 min, does not ionise well. Data were in accordance with previously reported literature.[10]

3,3-Diphenylpropan-1-ol (**21n**)

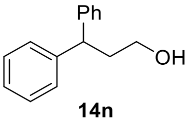

14n

To a round-bottomed flask was added ethyl 3,3-diphenylpropanoate (**13n**, 1.0 eq, 181 mg, 0.71 mmol) and the vessel was purged with N_2 (x 3) and THF (3 mL, 0.24 M) was added. The mixture was cooled to 0 °C in an ice-bath then, $LiAlH_4$ [2 M in THF] (1.2 eq, 0.8 mL, 1.6 mmol) was added and the reaction mixture was stirred for 30 min. The reaction mixture was quenched with the slow addition of water (1 mL) at 0 °C and was concentrated *in vacuo* to give a black oil. The crude was diluted with water (10 mL), saturated aqueous solution of Rochelle salt (20 mL) and EtOAc (40 mL). The aqueous layer was extracted with EtOAc (3 x 40 mL). The organics were combined, filtered through a hydrophobic frit and concentrated *in vacuo* to give 3,3-diphenylpropan-1-ol (**14n**) as a colourless oil (151 mg, 96%).

^{1}**H NMR** (400 MHz, $CDCl_3$, 303 K) δ (ppm) = 7.35 - 7.26 (m, 8H), 7.24 - 7.17 (m, 2H), 4.17 (t, *J* = 7.8 Hz, 1H), 3.68 - 3.61 (m, 2H), 2.35 (td, *J* = 6.5, 7.9 Hz, 2H); ^{13}**C NMR** (101 MHz, $CDCl_3$, 303 K) δ (ppm) = 144.5, 128.5, 127.9, 126.3, 61.1, 47.4, 38.3; **LCMS (ES+):** t_R = 1.04 min, does not ionise well. Data were in accordance with previously reported literature.[11]

Mesylation

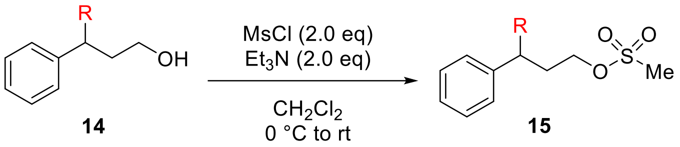

3-Phenylbutyl methanesulfonate (15m)

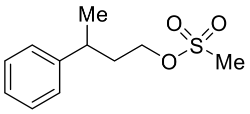

15m

A solution of 3-phenylbutan-1-ol (**14m**, 1.0 eq, 265 mg, 1.76 mmol) and Et_3N (2.0 eq, 0.496 mL, 3.53 mmol) in CH_2Cl_2 (5 mL, 0.35 M) was cooled to 0 °C and place under an N_2 atmosphere. To the solution was added methanesulfonyl chloride (2.0 eq, 0.273 mL, 3.53 mmol) dropwise. The reaction was allowed to warm to rt and stirred for 40 min. The reaction mixture was diluted with CH_2Cl_2 (10 mL) and washed with water (10 mL) and saturated aqueous NH_4Cl solution (2 x 10 mL). The organic layer was filtered through a hydrophobic frit and concentrated *in vacuo* to give a pale yellow oil. The crude was purified on a 5 g Sfar Biotage column, eluting with 0-40% EtOAc:heptane for 10 column volumes. The relevant fractions were combined and concentrated *in vacuo* to give 3-phenylbutyl methanesulfonate (**15m**) as a colourless oil (303 mg, 75%).

^{1}**H NMR** (400 MHz, $CDCl_3$, 303 K) δ (ppm) = 7.36 - 7.29 (m, 2H), 7.26 - 7.17 (m, 3H), 4.16 (td, *J* = 6.2, 9.5 Hz, 1H), 4.11 - 3.99 (m, 1H), 2.99 – 2.86 (m, 4H), 2.13 - 1.93 (m, 2H), 1.32 (d, *J* = 6.8 Hz, 3H); ^{13}**C NMR** (101 MHz, $CDCl_3$, 303 K) δ (ppm) = 145.3, 128.7, 126.9, 126.6, 68.5, 37.2, 37.2, 36.2, 22.3; **LCMS (ES−):** t_R = 1.04 min, $[M-H]^-$ 227.02. Data were in accordance with previously reported literature.[12]

3,3-Diphenylpropyl methanesulfonate (15n)

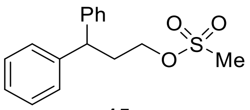

15n

A solution of 3,3-diphenylpropan-1-ol (**14n**, 1.0 eq, 139 mg, 0.655 mmol) and Et_3N (2.0 eq, 0.184 mL, 1.31 mmol) in CH_2Cl_2 (2 mL, 0.32 M) was cooled to 0 °C and place under an N_2 atmosphere. To the solution was added methanesulfonyl chloride (2.0 eq, 0.101 mL, 1.31 mmol) dropwise. The reaction

was allowed to warm to rt and stirred for 1 h 50 min. The reaction mixture was diluted with CH_2Cl_2 (10 mL) and washed with water (10 mL) and saturated aqueous NH_4Cl solution (2 x 10 mL). The organic layer was filtered through a hydrophobic frit and concentrated *in vacuo* to give a pale yellow oil. The crude was purified on a 5 g Sfar Biotage column, eluting with 0-40% EtOAc:heptane for 10 column volumes. The relevant fractions were combined and concentrated *in vacuo* to give 3,3-diphenylpropyl methanesulfonate (**15n**) as a colourless gum (159 mg, 83%).

^{1}H NMR (400 MHz, $CDCl_3$, 303 K) δ (ppm) = 7.36 - 7.30 (m, 4H), 7.28 - 7.20 (m, 6H), 4.24 - 4.12 (m, 3H), 2.92 (s, 3H), 2.53 (td, *J* = 6.4, 7.8 Hz, 2H); **^{13}C NMR** (101 MHz, $CDCl_3$, 303 K) δ (ppm) = 143.2, 128.7, 127.8, 126.7, 68.3, 47.0, 37.2, 34.8; **LCMS (ES+):** t_R = 1.15 min, $[M+H]^+$ 291.12. Data were in accordance with previously reported literature.[13]

S_N2 Reactions

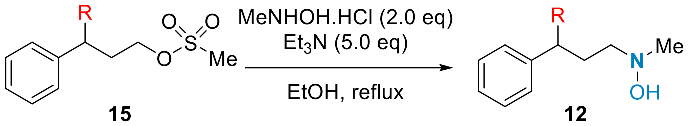

15 **12**

N-Methyl-*N*-(3-phenylbutyl)hydroxylamine (**12m**)

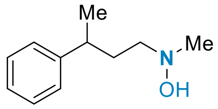

12m

3-Phenylbutyl methanesulfonate (**15m**, 1.0 eq, 282 mg, 1.23 mmol), *N*-methylhydroxylamine hydrochloride (2.0 eq, 206 mg, 2.47 mmol), Et_3N (5.0 eq, 0.868 mL, 6.18 mmol) and EtOH (10 mL, 0.12 M) were placed in a flask and heated to reflux for 3 h. The reaction mixture was concentrated *in vacuo* to give a white solid which was diluted with water (15 mL) and CH_2Cl_2 (20 mL) and the layers were separated. The aqueous layer was extracted with CH_2Cl_2 (3 x 20 mL). The organics were combined, filtered through a hydrophobic frit and concentrated *in vacuo* to give a colourless oil. The crude material was purified on a 10 g Sfar Biotage column, eluting with 0-45% EtOAc:heptane for 10 column volumes. The relevant fractions were combined and concentrated *in vacuo* to give *N*-methyl-*N*-(3-phenylbutyl)hydroxylamine (**12m**) as a colourless oil (150 mg, 68%).

^{1}H NMR (400 MHz, $CDCl_3$, 303 K) δ (ppm) = 7.36 - 7.27 (m, 2H), 7.25 - 7.18 (m, 3H), 6.69 (br s, 1H), 2.80 (sxt, *J* = 7.1 Hz, 1H), 2.67 - 2.45 (m, 5H), 1.98 - 1.83 (m, 2H), 1.29 (d, *J* = 6.8 Hz, 3H); **^{13}C NMR** (101 MHz, $CDCl_3$, 303 K) δ (ppm) = 147.0, 128.4, 126.9, 126.0, 60.6, 48.8, 37.9, 35.7, 22.7; **LCMS (ES+):** t_R = 0.49 min, $[M+H]^+$ 180.12; **ν_{max} (neat)**: 3249, 2958, 2869, 1602, 1493, 1451 cm^{-1}; **HRMS (ESI) m/z**: $[M+H]^+$ Calcd for $C_{11}H_{18}NO$ 180.1383, found 180.1374.

N-(3,3-Diphenylpropyl)-*N*-methylhydroxylamine (**12n**)

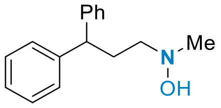

12n

3,3-Diphenylpropyl methanesulfonate (**15n** 1.0 eq, 150 mg, 0.517 mmol), *N*-methylhydroxylamine hydrochloride (4.0 eq, 200 mg, 1.20 mmol), Et_3N (5.0 eq, 0.363 mL, 2.58 mmol) and EtOH (5 mL, 0.1 M) were placed in a ROUND-BOTTOMED FLASKand heated to reflux for 8.5 h. The reaction mixture was concentrated *in vacuo* to give a white solid which was diluted with water (15 mL) and CH_2Cl_2 (20 mL) and the layers were separated. The aqueous layer was extracted with CH_2Cl_2 (3 x 20 mL). The organics were combined, filtered through a hydrophobic frit and concentrated *in vacuo* to give a colourless oil. The crude material was purified on a 10 g Sfar Biotage column, eluting with 0-45% EtOAc:heptane for 10 column volumes. The relevant fractions were combined and concentrated *in vacuo* to give *N*-(3,3-diphenylpropyl)-*N*-methylhydroxylamine (**12n**) as a white solid (80 mg, 64%).

Mp: 86-89 °C; **^{1}H NMR** (400 MHz, $CDCl_3$, 303 K) δ (ppm) = 7.34 - 7.25 (m, 8H), 7.22 - 7.15 (m, 2H), 4.05 (t, *J* = 7.8 Hz, 1H), 2.64 - 2.57 (m, 5H), 2.41 - 2.31 (m, 2H); **^{13}C NMR** (101 MHz, $CDCl_3$, 303 K) δ (ppm) = 144.7, 128.5, 127.8, 126.2, 60.6, 48.9, 48.8, 33.1; **LCMS (ES+):** t_R = 0.66 min, $[M+H]^+$ 242.21; **ν_{max} (neat)**: 3206, 3021, 2873, 1597, 1494, 1448 cm^{-1}; **HRMS (ESI) m/z**: $[M+H]^+$ Calcd for $C_{16}H_{20}NO$ 242.1539, found 242.1531.

General Procedure 7 – Synthesis of NCR Precursors via an S_NAr reaction

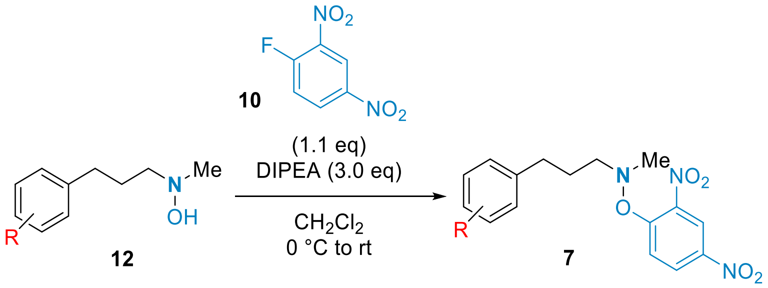

A vial was charged with **hydroxylamine 12** (1.0 eq), DIPEA (1.4 eq) and CH_2Cl_2 before being cooled to 0 °C. To the vial was added 1-fluoro-2,4-dinitrobenzene (**10**, 1.1 eq) and the reaction was left to stir till the reaction had completed, whilst warming to rt. The reaction mixture was diluted with water and the layers were separated. The aqueous layer was extracted with CH_2Cl_2 (x 3), the organics were combined, filtered through a hydrophobic frit and concentrated *in vacuo* to give the crude NCR precursor. The crude product was purified on a silica Sfar Biotage column, eluting with 0-10% EtOAc:heptane over 10 column volumes, unless stated otherwise.

O-(2,4-Dinitrophenyl)-*N*-methyl-*N*-(3-phenylpropyl)hydroxylamine (**7a**)

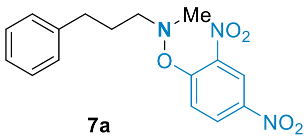

7a

This experiment followed General Procedure 7, using *N*-(3-phenylpropyl)hydroxylamine (**12a**, 1.0 eq, 444 mg, 2.69 mmol), 1-fluoro-2,4-dinitrobenzene **10** (1.1 eq, 550 mg, 2.96 mmol), DIPEA (3.0 eq, 1.41 mL, 8.07 mmol) gave a dark red oil which was purified by a 55 g Biotage KP-NH column, eluting with 0-10% EtOAc:heptane for 20 min. The relevant fractions were combined and concentrated *in vacuo* to yield *O*-(2,4-dinitrophenyl)-*N*-methyl-*N*-(3-phenylpropyl)hydroxylamine (**7a**) as an orange oil (453 mg, 46%).

^{1}H NMR (400 MHz, $CDCl_3$) δ 8.80 (d, *J* = 3.0 Hz, 1 H), 8.40 (dd, *J* = 9.4, 3.0 Hz, 1 H), 7.88 (d, *J* = 9.4 Hz, 1 H), 7.36 - 7.27 (m, 2 H), 7.24 - 7.13 (m, 3 H), 3.26 - 2.94 (br m, 2 H), 2.93 - 2.86 (s, 3 H), 2.71 (t, *J* = 7.1 Hz, 2 H), 1.92 (quin, *J* = 7.1 Hz, 2 H); **^{13}C NMR** (101 MHz, $CDCl_3$) δ 158.3, 141.1, 140.2, 136.3, 129.3, 128.5, 128.3, 126.1, 122.0, 116.8, 60.6, 46.4, 33.3, 28.4; **LCMS (ES+):** t_R = 1.38 min, $[M+H]^+$ 332.14; **ν_{max} (neat)**: 3093, 3062, 3025, 2926, 2850, 1949, 1600, 1522, 1338, 1312 cm^{-1}; **HRMS (ESI) m/z**: $[M+H]^+$ Calcd for $C_{16}H_{18}N_3O_5$ 332.1241, found 332.1241.

O-(2,4-Dinitrophenyl)-*N*-methyl-*N*-(3-(*p*-tolyl)propyl)hydroxylamine (**7b**)

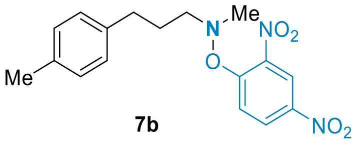

7b

This experiment followed General Procedure 7, using *N*-methyl-*N*-(3-(*p*-tolyl)propyl)hydroxylamine (**12b**, 1.0 eq, 475 mg, 2.65 mmol), 1-fluoro-2,4-dinitrobenzene **10** (1.1 eq, 542 mg, 2.91 mmol) and DIPEA (3.0 eq, 1.4 mL, 8.02 mmol) gave *O*-(2,4-dinitrophenyl)-*N*-methyl-*N*-(3-(*p*-tolyl)propyl)hydroxylamine (**7b**) as a yellow oil (808 mg, 88%).

^{1}H NMR (400 MHz, $CDCl_3$, 303 K) δ (ppm) = 8.80 (d, *J* = 2.9 Hz, 1H), 8.40 (dd, *J* = 2.9, 9.3 Hz, 1H), 7.89 (d, *J* = 9.3 Hz, 1H), 7.11 (d, *J* = 8.0 Hz, 2H), 7.04 (d, *J* = 8.0 Hz, 2H), 3.09 (br s, 2H), 2.89 (s, 3H), 2.66 (t, *J* = 7.6 Hz, 2H), 2.33 (s, 3H), 1.90 (quin, *J* = 7.5 Hz, 2H); **^{13}C NMR** (101 MHz, $CDCl_3$, 303 K) δ (ppm) = 158.3, 140.2, 138.0, 136.3, 135.6, 129.3, 129.2, 128.2, 122.0, 116.9, 60.6, 46.4, 32.8, 28.5, 21.0; **LCMS (ES+)**: t_R = 1.48 min, $[M+H]^+$ 346.34; **ν_{max} (neat)**: 3109, 2930, 2850, 1602, 1517, 1338 cm^{-1}; **HRMS (ESI) m/z**: $[M+H]^+$ Calcd for $C_{17}H_{20}N_3O_5$ 346.1397, found 346.1391.

N-(3-(4-(*tert*-Butyl)phenyl)propyl)-*O*-(2,4-dinitrophenyl)-*N*-methylhydroxylamine (**7c**)

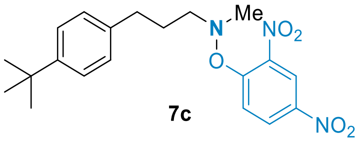

7c

This experiment followed General Procedure 7, using *N*-(3-(4-(*tert*-butyl)phenyl)propyl)-*N*-methylhydroxylamine (**12c**, 1.0 eq, 630 mg, 2.85 mmol), 1-fluoro-2,4-dinitrobenzene **10** (1.1 eq, 583 mg, 3.13 mmol) and DIPEA (3.0 eq, 1.49 mL, 8.54 mmol) gave *N*-(3-(4-(*tert*-butyl)phenyl)propyl)-*O*-(2,4-dinitrophenyl)-*N*-methylhydroxylamine (**7c**) as a yellow oil (540 mg, 49%).

^{1}H NMR (400 MHz, $CDCl_3$, 303 K) δ (ppm) = 8.81 (d, *J* = 2.4 Hz, 1H), 8.41 (dd, *J* = 2.5, 9.3 Hz, 1H), 7.90 (d, *J* = 9.3 Hz, 1H), 7.32 (d, *J* = 8.3 Hz, 2H), 7.09 (d, *J* = 8.3 Hz, 2H), 2.90 (s, 3H), 2.67 (t, *J* = 7.4 Hz, 2H), 1.91 (quin, *J* = 7.3 Hz, 2H), 1.35 - 1.31 (m, 11H); **^{13}C NMR** (101 MHz, $CDCl_3$, 303 K) δ (ppm) = 158.3, 149.0, 140.3, 138.0, 136.3, 129.3, 127.9, 125.4, 122.0, 116.9, 60.7, 46.4, 34.4, 32.7, 31.4, 28.4; **LCMS (ES+):** t_R = 1.60 min, $[M+H]^+$ 388.30; **ν_{max} (neat)**: 2959, 2866, 1682, 1602, 1525, 1469, 1339 cm^{-1}; **HRMS (ESI) m/z**: $[M+H]^+$ Calcd for $C_{20}H_{26}N_3O_5$ 388.1867, found 388.1864.

N-(3-([1,1'-Biphenyl]-4-yl)propyl)-*O*-(2,4-dinitrophenyl)-*N*-methylhydroxylamine (**7d**)

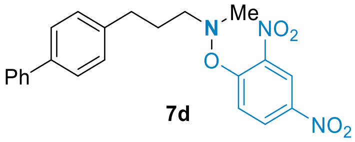

7d

This experiment followed General Procedure 7, using *N*-(3-([1,1'-biphenyl]-4-yl)propyl)-*N*-methylhydroxylamine (**12d**, 1.0 eq, 186 mg, 0.771 mmol), 1-fluoro-2,4-dinitrobenzene **10** (1.1 eq, 158 mg, 0.848 mmol) and DIPEA (3.0 eq, 0.40 mL, 2.31 mmol) gave *N*-(3-([1,1'-biphenyl]-4-yl)propyl)-*O*-(2,4-dinitrophenyl)-*N*-methylhydroxylamine (**7d**) as a yellow oil (254 mg, 73%).

^{1}H NMR (400 MHz, $CDCl_3$, 303 K) δ (ppm) = 8.81 (d, *J* = 2.4 Hz, 1H), 8.41 (dd, *J* = 2.5, 9.7 Hz, 1H), 7.90 (d, *J* = 9.8 Hz, 1H), 7.62 - 7.56 (m, 2H), 7.53 (d, *J* = 7.8 Hz, 2H), 7.45 (t, *J* = 7.6 Hz, 2H), 7.35 (tt, *J* = 1.5, 7.4 Hz, 1H), 7.23 (d, *J* = 8.3 Hz, 2H), 3.23 - 2.97 (m, 2H), 2.90 (s, 3H), 2.74 (t, *J* = 7.6 Hz, 2H), 1.96 (quin, *J* = 7.3 Hz, 2H); **^{13}C NMR** (101 MHz, $CDCl_3$, 300 K) δ (ppm) = 158.3, 140.8, 140.2, 139.1, 136.3, 129.3, 128.8, 127.21, 127.16, 126.9, 122.0, 116.9, 60.6, 46.4, 32.9, 28.4; **LCMS (ES+):** t_R = 1.54 min, $[M+H]^+$ 408.21; **ν_{max} (neat)**: 3115, 3027, 2926, 2860, 1602, 1522, 1338 cm^{-1}; **HRMS (ESI) m/z**: $[M+H]^+$ Calcd for $C_{22}H_{22}N_3O_5$ 408.1554, found 408.1557.

O-(2,4-Dinitrophenyl)-*N*-(3-(4-methoxyphenyl)propyl)-*N*-methylhydroxylamine (**7e**)

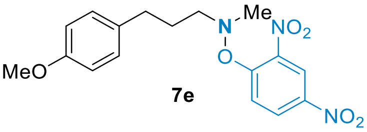

This experiment followed General Procedure 7, using *N*-(3-(4-methoxyphenyl)propyl)-*N*-methylhydroxylamine (**12e**, 1.0 eq, 167 mg, 0.855 mmol), 1-fluoro-2,4-dinitrobenzene **10** (1.1 eq, 175 mg, 0.941 mmol), DIPEA (3.0 eq, 0.44 mL, 2.56 mmol) gave a dark red oil which was purified on a 28 g Biotage KP-NH column, eluting with 0-10% EtOAc:heptane for 20 min. The relevant fractions were combined and concentrated *in vacuo* to yield *O*-(2,4-dinitrophenyl)-*N*-(3-(4-methoxyphenyl)propyl)-*N*-methylhydroxylamine (**7e**) as a yellow oil (214 mg, 92%).

^{1}H NMR (400 MHz, $CDCl_3$) δ 8.79 (d, *J* = 2.7 Hz, 1 H) 8.39 (dd, *J* = 9.4, 2.7 Hz, 1 H) 7.87 (d, *J* = 9.4 Hz, 1 H) 7.05 (d, *J* = 8.6 Hz, 2 H) 6.82 (d, *J* = 8.6 Hz, 2 H) 3.78 (s, 3 H) 3.18 - 2.92 (br m, 2 H) 2.87 (s, 3 H) 2.62 (t, *J* = 7.6 Hz, 2 H) 1.86 (quin, *J* = 7.4 Hz, 2 H); **^{13}C NMR** (101 MHz, $CDCl_3$) δ 158.3, 158.0, 140.2, 136.4, 133.1, 129.2, 129.2, 122.0, 116.9, 113.9, 60.6, 55.3, 46.4, 32.4, 28.6; **LCMS (ES+)**: t_R = 1.36 min, $[M+H]^+$ 362.15; **ν_{max} (neat)**: 3109, 3003, 2934, 2839, 1601, 1510, 1338 cm^{-1}; **HRMS (ESI) m/z**: $[M+H]^+$ Calcd for $C_{17}H_{20}N_3O_6$ 362.1347, found 362.1360.

N-(3-(4-Bromophenyl)propyl)-*O*-(2,4-dinitrophenyl)-*N*-methylhydroxylamine (**7f**)

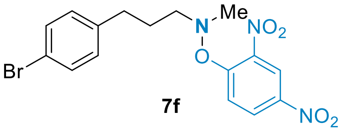

This experiment followed General Procedure 7, using *N*-(3-(4-bromophenyl)propyl)-*N*-methylhydroxylamine (**12f**, 1.0 eq, 455 mg, 1.864 mmol), 1-fluoro-2,4-dinitrobenzene **10** (1.1 eq, 382 mg, 2.050mmol) and DIPEA (3.0 eq, 0.78 mL, 5.60 mmol) gave *N*-(3-(4-bromophenyl)propyl)-*O*-(2,4-dinitrophenyl)-*N*-methylhydroxylamine (**7f**) as a yellow oil (219 mg, 29%).

^{1}H NMR (400 MHz, $CDCl_3$, 300 K) δ (ppm) = 8.78 (d, *J* = 2.7 Hz, 1H), 8.40 (dd, *J* = 2.7, 9.4 Hz, 1H), 7.88 (d, *J* = 9.4 Hz, 1H), 7.38 (d, *J* = 8.4 Hz, 2H), 7.02 (d, *J* = 8.4 Hz, 2H), 3.06 (br s, 2H), 2.89 (s, 3H), 2.65 (t, *J* = 7.6 Hz, 2H), 1.89 (quin, *J* = 7.4 Hz, 2H); **^{13}C NMR** (101 MHz, $CDCl_3$, 300 K) δ (ppm) = 158.2, 140.2, 140.1, 136.2, 131.5, 130.1, 129.4, 122.0, 119.8, 116.8, 60.4, 46.4, 32.7, 28.2; **LCMS (ES+):** t_R = 1.49 min, $[M+H]^+$ 410.16; **ν_{max} (neat)**: 3097, 2924, 2859, 2782, 1681, 1601, 1521, 1486 cm^{-1}; **HRMS (ESI) m/z**: $[M+H]^+$ Calcd for $C_{16}H_{17}{}^{79}BrN_3O_5$ 410.0346, found 410.0352.

N-(3-(4-Chlorophenyl)propyl)-*O*-(2,4-dinitrophenyl)-*N*-methylhydroxylamine (**7g**)

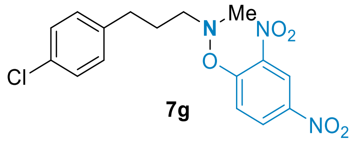

7g

This experiment followed General Procedure 7, using *N*-(3-(4-chlorophenyl)propyl)-*N*-methylhydroxylamine (**12g**, 1.0 eq, 120 mg, 0.601 mmol), 1-fluoro-2,4-dinitrobenzene **10** (1.1 eq, 123 mg, 0.661 mmol) and DIPEA (3.0 eq, 0.35 mL, 1.80 mmol) gave *N*-(3-(4-chlorophenyl)propyl)-*O*-(2,4-dinitrophenyl)-*N*-methylhydroxylamine (**7g**) as a yellow oil (181 mg, 82%).

^{1}H NMR (400 MHz, $CDCl_3$, 303 K) δ (ppm) = 8.80 (d, *J* = 2.9 Hz, 1H), 8.41 (dd, *J* = 2.6, 9.4 Hz, 1H), 7.86 (d, *J* = 9.3 Hz, 1H), 7.26 (d, *J* = 8.3 Hz, 2H), 7.08 (d, *J* = 8.3 Hz, 2H), 3.07 (br s, 2H), 2.88 (s, 3H), 2.66 (t, *J* = 7.6 Hz, 2H), 1.89 (quin, *J* = 7.5 Hz, 2H); **^{13}C NMR** (101 MHz, $CDCl_3$, 303 K) δ (ppm) = 158.1, 140.3, 139.5, 136.3, 131.9, 129.6, 129.3, 128.6, 122.0, 116.7, 60.4, 46.4, 32.6, 28.3; **LCMS (ES+):** t_R = 1.47 min, $[M+H]^+$ 366.20; **ν_{max} (neat)**: 3112, 2924, 2860, 1602, 1523, 1339 cm^{-1}; **HRMS (ESI) m/z**: $[M+H]^+$ Calcd for $C_{16}H_{17}{}^{35}ClN_3O_5$ 366.0851, found 366.0851.

O-(2,4-Dinitrophenyl)-*N*-(3-(3-methoxyphenyl)propyl)-*N*-methylhydroxylamine (**7h**)

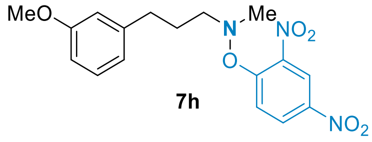

7h

This experiment followed General Procedure 7, using *N*-(3-(3-methoxyphenyl)propyl)-*N*-methylhydroxylamine (**12h**, 1.0 eq, 667 mg, 3.416 mmol), 1-fluoro-2,4-dinitrobenzene **10** (1.1 eq, 699 mg, 3.758 mmol) and DIPEA (3.0 eq, 1.79 mL, 10.248 mmol) gave *O*-(2,4-dinitrophenyl)-*N*-(3-(3-methoxyphenyl)propyl)-*N*-methylhydroxylamine (**7h**) as a yellow oil (992 mg, 80%).

^{1}H NMR (400 MHz, $CDCl_3$, 303 K) δ (ppm) = 8.80 (d, *J* = 2.4 Hz, 1H), 8.40 (dd, *J* = 2.5, 9.4 Hz, 1H), 7.89 (d, *J* = 9.3 Hz, 1H), 7.21 (t, *J* = 7.8 Hz, 1H), 6.79 - 6.68 (m, 3H), 3.80 (s, 3H), 3.07 (br s, 2H), 2.89 (s, 3H), 2.67 (t, *J* = 7.6 Hz, 2H), 1.91 (quin, *J* = 7.5 Hz, 2H); **^{13}C NMR** (101 MHz, $CDCl_3$, 303 K) δ (ppm) = 159.7, 158.3, 142.7, 140.3, 136.3, 129.5, 129.3, 122.0, 120.7, 116.9, 114.3, 111.1, 60.6, 55.1, 46.4, 33.0, 28.3; **LCMS (ES+)**: t_R = 1.35 min, $[M+H]^+$ 362.26; **ν_{max} (neat)**: 3112, 2938, 2842, 1602, 1523, 1339 cm^{-1}; **HRMS (ESI) m/z**: $[M+H]^+$ Calcd for $C_{17}H_{20}N_3O_6$ 362.1347, found 362.1350.

N-(3-(3-Bromophenyl)propyl)-*O*-(2,4-dinitrophenyl)-*N*-methylhydroxylamine (**7i**)

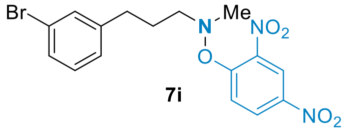

7i

This experiment followed General Procedure 7, using *N*-(3-(3-bromophenyl)propyl)-*N*-methylhydroxylamine (**12i**, 1.0 eq, 198 mg, 0.811 mmol), 1-fluoro-2,4-dinitrobenzene **10** (1.1 eq, 166 mg, 0.892 mmol) and DIPEA (3.0 eq, 0.425 mL, 2.433 mmol) gave *N*-(3-(4-bromophenyl)propyl)-*O*-(2,4-dinitrophenyl)-*N*-methylhydroxylamine (**7i**) as a yellow oil (166 mg, 50%).

^{1}H NMR (400 MHz, $CDCl_3$, 300 K) δ (ppm) = 8.81 (d, *J* = 2.7 Hz, 1H), 8.42 (dd, *J* = 2.7, 9.4 Hz, 1H), 7.87 (d, *J* = 9.4 Hz, 1H), 7.34 (td, *J* = 1.6, 7.9 Hz, 1H), 7.30 - 7.29 (m, 1H), 7.16 (t, *J* = 7.6 Hz, 1H), 7.10 - 7.05 (m, 1H), 3.05 (br s, 2H), 2.89 (s, 3H), 2.67 (t, *J* = 7.6 Hz, 2H), 1.90 (quin, *J* = 7.3 Hz, 2H); **^{13}C NMR** (101 MHz, $CDCl_3$, 300 K) δ (ppm) = 158.1, 143.5, 140.3, 136.3, 131.4, 130.1, 129.3, 129.3, 127.0, 122.6, 122.0, 116.8, 60.4, 46.4, 32.9, 28.2; **LCMS (ES+):** t_R = 1.45 min, $[M+H]^+$ 410.05; **ν_{max} (neat)**: 3112, 2919, 2850, 1601, 1522, 1337 cm^{-1}; **HRMS (ESI) m/z**: $[M+H]^+$ Calcd for $C_{16}H_{17}{}^{79}BrN_3O_5$ 410.0346, found 410.0342.

O-(2,4-Dinitrophenyl)-*N*-methyl-*N*-(3-(*p*-tolyl)propyl)hydroxylamine (**7j**)

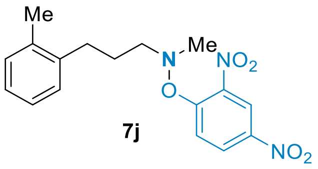

7j

This experiment followed General Procedure 7, using *N*-methyl-*N*-(3-(*o*-tolyl)propyl)-*N*-methylhydroxylamine (**12j**, 1.0 eq, 500 mg, 2.79 mmol), 1-fluoro-2,4-dinitrobenzene (1.1 eq, 571 mg, 3.07 mmol) and DIPEA (3.0 eq, 1.46 mL, 8.37 mmol) gave *O*-(2,4-dinitrophenyl)-*N*-methyl-*N*-(3-(*p*-tolyl)propyl)hydroxylamine (**7j**) as a yellow oil (819 mg, 85%).

^{1}H NMR (400 MHz, $CDCl_3$, 300 K) δ (ppm) = 8.79 (d, *J* = 2.7 Hz, 1H), 8.42 (dd, *J* = 2.7, 9.4 Hz, 1H), 7.93 (d, *J* = 9.4 Hz, 1H), 7.18 - 7.07 (m, 4H), 3.14 (br s, 2H), 2.94 (s, 3H), 2.74 - 2.67 (m, 2H), 2.29 (s, 3H), 1.96 - 1.85 (m, 2H); **^{13}C NMR** (101 MHz, $CDCl_3$, 300 K) δ (ppm) = 158.3, 140.2, 139.4, 136.3, 135.8, 130.3, 129.4, 128.8, 126.2, 126.1, 122.0, 116.9, 60.9, 46.5, 30.7, 27.3, 19.2; **LCMS (ES+)**: t_R = 1.46 min, $[M+H]^+$ 346.35; **ν_{max} (neat)**: 3098, 3066, 2934, 2864, 1600, 1519, 1338 cm^{-1}; **HRMS (ESI) m/z**: $[M+H]^+$ Calcd for $C_{17}H_{20}N_3O_5$ 346.1397, found 346.1393.

N-(3-(2-Fluorophenyl)propyl)-*O*-(2,4-dinitrophenyl)-*N*-methylhydroxylamine (**7k**)

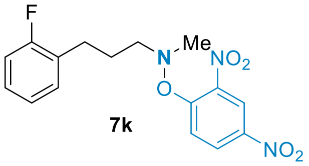

7k

This experiment followed General Procedure 7, using *N*-(3-(2-fluorophenyl)propyl-*N*-methylhydroxylamine (**12k**, 1.0 eq, 414 mg, 2.259 mmol), 1-fluoro-2,4-dinitrobenzene **10** (1.1 eq, 463

mg, 2.485 mmol) and DIPEA (3.0 eq, 1.2 mL, 6.87 mmol) gave *N*-(3-(2-fluorophenyl)propyl)-*O*-(2,4-dinitrophenyl)-*N*-methylhydroxylamine (**7k**) as a yellow oil (570 mg, 74%).

^{1}H NMR (400 MHz, $CDCl_3$, 300 K) δ (ppm) = 8.80 (d, *J* = 2.7 Hz, 1H), 8.41 (dd, *J* = 2.7, 9.4 Hz, 1H), 7.90 (d, *J* = 9.4 Hz, 1H), 7.23 - 7.12 (m, 2H), 7.09 - 6.96 (m, 2H), 3.10 (br s, 2H), 2.90 (s, 3H), 2.73 (t, *J* = 7.6 Hz, 2H), 1.90 (quin, *J* = 7.3 Hz, 2H); **^{19}F NMR** (376 MHz, $CDCl_3$, 300 K) δ (ppm) = −118.85 (s, 1F); **^{13}C NMR** (151 MHz, $CDCl_3$, 300 K) δ (ppm) = 161.0 (d, *J* = 245 Hz), 158.2, 140.2, 136.2, 130.4 (d, *J* = 5 Hz), 129.3, 128.0 (d, *J* = 16 Hz), 127.8 (d, *J* = 8 Hz), 124.0 (d, *J* = 4 Hz), 121.9, 116.8, 115.2 (d, *J* = 22 Hz), 60.5, 46.4, 27.2 (d, *J* = 1 Hz), 26.5 (d, *J* = 2 Hz); **LCMS (ES+)**: t_R = 1.40 min, $[M+H]^+$ 350.24; **ν_{max} (neat)**: 3115, 2936, 2865, 1601, 1520, 1489, 1339 cm^{-1}; **HRMS (ESI) m/z**: $[M+H]^+$ Calcd for $C_{16}H_{17}FN_3O_5$ 350.1147, found 350.1147.

N-(3-(2-Bromophenyl)propyl)-*O*-(2,4-dinitrophenyl)-*N*-methylhydroxylamine (**7l**)

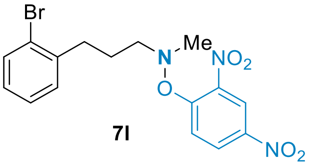

7l

This experiment followed General Procedure 7, using *N*-(3-(2-bromophenyl)propyl)-*N*-methylhydroxylamine (**12l**, 1.0 eq, 400 mg, 1.638 mmol), 1-fluoro-2,4-dinitrobenzene (1.1 eq, 335 mg, 1.802 mmol) and DIPEA (3.0 eq, 0.86 mL, 4.92 mmol) gave *N*-(3-(2-bromophenyl)propyl)-*O*-(2,4-dinitrophenyl)-*N*-methylhydroxylamine (**7l**) as a yellow oil (498 mg, 74%).

^{1}H NMR (400 MHz, $CDCl_3$, 303 K) δ (ppm) = 8.80 (d, *J* = 2.9 Hz, 1H), 8.41 (dd, *J* = 2.8, 9.3 Hz, 1H), 7.91 (d, *J* = 9.3 Hz, 1H), 7.53 (dd, *J* = 1.2, 8.1 Hz, 1H), 7.24 (dt, *J* = 1.2, 7.5 Hz, 1H), 7.17 (dd, *J* = 2.0, 7.7 Hz, 1H), 7.07 (dt, *J* = 2.0, 7.6 Hz, 1H), 3.12 (br s, 2H), 2.91 (s, 3H), 2.82 (t, *J* = 7.8 Hz, 2H), 1.92 (quin, *J* = 7.4 Hz, 2H); **^{13}C NMR** (101 MHz, $CDCl_3$, 303 K) δ (ppm) = 158.2, 140.5, 140.3, 136.3, 132.9, 130.2, 129.3, 127.9, 127.6, 124.4, 122.0, 116.9, 60.6, 46.4, 33.7, 27.1; **LCMS (ES+)**: t_R = 1.47 min, $[M+H]^+$ 410.09; **ν_{max} (neat)**: 3111, 3060, 2926, 2864, 1601, 1552, 1469, 1337 cm^{-1}; **HRMS (ESI) m/z**: $[M+H]^+$ Calcd for $C_{16}H_{17}{}^{79}BrN_3O_5$ 410.0346, found 410.0348.

O-(2,4-Dinitrophenyl)-*N*-methyl-*N*-(3-phenylbutyl)hydroxylamine (**7m**)

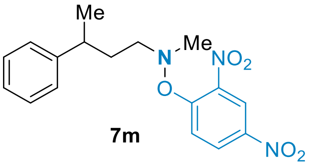

7m

This experiment followed General Procedure 7, using *N*-methyl-*N*-(3-phenylbutyl)hydroxylamine (**12m**, 1.0 eq, 142 mg, 0.792 mmol), 1-fluoro-2,4-dinitrobenzene **10** (1.1 eq, 162 mg, 0.871 mmol) and

DIPEA (3.0 eq, 0.33 mL, 2.38 mmol) gave *O*-(2,4-dinitrophenyl)-*N*-methyl-*N*-(3-phenylbutyl)hydroxylamine (**7m**) as a yellow oil (242 mg, 88%).

^{1}H NMR (400 MHz, $CDCl_3$, 300 K) δ (ppm) = 8.80 (d, *J* = 2.7 Hz, 1H), 8.40 (br dd, *J* = 2.5, 9.4 Hz, 1H), 7.83 (br d, *J* = 9.6 Hz, 1H), 7.36 - 7.29 (m, 2H), 7.25 - 7.14 (m, 3H), 2.98 - 2.75 (m, 6H), 1.86 (q, *J* = 7.4 Hz, 2H), 1.26 (br d, *J* = 6.9 Hz, 3H); **^{13}C NMR** (101 MHz, $CDCl_3$, 300 K) δ (ppm) = 158.3, 146.1, 140.2, 129.3, 128.6, 126.9, 126.3, 122.0, 116.8, 110.0, 59.6, 46.4, 37.9, 35.0, 22.7; **LCMS (ES+)**: t_R = 1.43 min, $[M+H]^+$ 346.20; **ν_{max} (neat)**: 3112, 3026, 2960, 2870, 1602, 1523, 1340 cm^{-1}; **HRMS (ESI) m/z**: $[M+H]^+$ Calcd for $C_{17}H_{20}N_3O_5$ 346.1397, found 346.1403.

O-(2,4-Dinitrophenyl)-*N*-(3,3-diphenylpropyl)-*N*-methylhydroxylamine (7n)

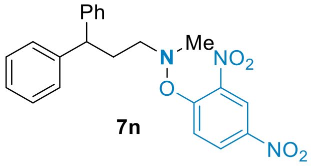

7n

This experiment followed General Procedure 7, using *N*-(3,3-diphenylpropyl)-*N*-methylhydroxylamine (**12n**, 1.0 eq, 78 mg, 0.323 mmol), 1-fluoro-2,4-dinitrobenzene (1.1 eq, 66 mg, 0.356 mmol) and Et_3N (3.0 eq, 0.136 mL, 0.97 mmol) gave *O*-(2,4-dinitrophenyl)-*N*-(3,3-diphenylpropyl)-*N*-methylhydroxylamine (**7n**) as a yellow oil (114 mg, 79%).

^{1}H NMR (400 MHz, $CDCl_3$, 303 K) δ (ppm) = 8.78 (d, *J* = 2.9 Hz, 1H), 8.38 (dd, *J* = 2.9, 9.3 Hz, 1H), 7.84 (d, *J* = 9.3 Hz, 1H), 7.32 - 7.16 (m, 10H), 4.02 (t, *J* = 7.8 Hz, 1H), 3.03 (br s, 2H), 2.83 (s, 3H), 2.33 (q, *J* = 7.3 Hz, 2H); **^{13}C NMR** (101 MHz, $CDCl_3$, 303 K) δ (ppm) = 158.2, 143.9, 140.3, 136.3, 129.3, 128.7, 127.7, 126.5, 122.0, 116.8, 59.7, 48.9, 46.4, 32.6; **LCMS (ES+)**: t_R = 1.47 min, $[M+H]^+$ 408.22; **ν_{max} (neat)**: 3115, 3082, 3026, 2930, 2876, 1601, 1522, 1338 cm^{-1}; **HRMS (ESI) m/z**: $[M+H]^+$ Calcd for $C_{22}H_{22}N_3O_5$ 408.1554, found 408.1556.

O-(2,4-Dinitrophenyl)-*N*-methyl-*N*-(2-phenoxyethyl)hydroxylamine (7o)

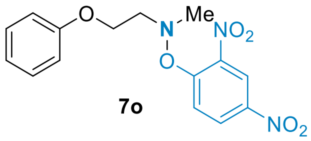

7o

This experiment followed General Procedure 7, using *N*-methyl-*N*-(2-phenyoxyethyl)hydroxylamine (**12o**, 1.0 eq, 100 mg, 0.598 mmol), 1-fluoro-2,4-dinitrobenzene **10** (1.1 eq, 122 mg, 0.658 mmol) and DIPEA (3.0 eq, 0.313 mL, 1.79 mmol) gave *O*-(2,4-dinitrophenyl)-*N*-methyl-*N*-(2-phenoxyethyl)hydroxylamine (**7o**) as a yellow solid (118 mg, 59%).

Mp: 91-93 °C; **^{1}H NMR** (400 MHz, $CDCl_3$, 303 K) δ (ppm) = 8.76 (s, 1H), 8.35 - 8.26 (m, 1H), 7.97 (d, *J* = 9.3 Hz, 1H), 7.29 - 7.22 (m, 2H), 6.95 (tt, *J* = 1.0, 7.3 Hz, 1H), 6.86 - 6.81 (m, 2H), 4.14 (t, *J* = 5.1 Hz, 2H), 3.46 (br m, 2H), 3.01 (s, 3H); **^{13}C NMR** (101 MHz, $CDCl_3$, 303 K) δ (ppm) = 158.2, 158.0, 140.5, 136.3, 129.6, 129.2, 121.8, 121.3, 117.3, 114.4, 64.1, 60.1, 46.9; **LCMS (ES+):** t_R = 1.28 min, $[M+H]^+$ 334.18; **ν_{max} (neat)**: 3117, 2977, 2934, 2880, 1605, 1517, 1335 cm^{-1}; **HRMS (ESI) m/z**: $[M+H]^+$ Calcd for $C_{15}H_{16}N_3O_6$ 334.1034, found 334.1035.

General Procedure 8 – C-H Amination to Synthesise Tetrahydroquinolines

To a vial containing a stirrer bar, the NCR precursor (1.0 eq) and $Ru(bpy)_3Cl_2.6H_2O$ (2 mol%) in CH_3CN (0.1 M) was added triflic acid (2.0 eq) and the reaction was left to stir at room temperature for 5 min. The reaction was diluted with 1 M KOH aqueous solution (5 mL), water (5 mL) and CH_2Cl_2 (15 mL) and the layers were separated. The aqueous layer was extracted with CH_2Cl_2 (3 x 15 mL). The organics were combined, filtered through a hydrophobic frit and concentrated *in vacuo* to give the crude product. The crude product was purified on a Biotage Sfar column, and the relevant fractions were combined and concentrated *in vacuo* to yield the desired product.

1-Methyl-1,2,3,4-tetrahydroquinoline (**8a**)

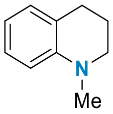

8a

This experiment followed General Procedure 8, using *O*-(2,4-dinitrophenyl)-*N*-methyl-*N*-(3-phenylpropyl)hydroxylamine (**7a**, 1.0 eq, 1.31 g, 3.36 mmol), triflic acid (2.0 eq, 0.6 mL, 6.72 mmol) and $Ru(bpy)_3Cl_2.6H_2O$ (2 mol%, 50 mg, 0.067 mmmol). The crude product was purified on a 10 g Sfar Biotage column, eluting with 0-30% EtOAc:heptane (10 column volumes) to give 1-methyl-1,2,3,4-tetrahydroquinoline (**8a**) as a yellow oil (427 mg, 86%).

^{1}H NMR (400 MHz, $CDCl_3$, 303 K) δ (ppm) = 7.10 (dt, *J* = 1.5, 7.8 Hz, 1H), 7.01 - 6.96 (m, 1H), 6.66 - 6.60 (m, 2H), 3.25 (t, *J* = 5.8 Hz, 2H), 2.91 (s, 3H), 2.80 (t, *J* = 6.6 Hz, 2H), 2.06 - 1.97 (m, 2H). **^{13}C NMR** (101 MHz, $CDCl_3$, 303 K) δ (ppm) = 146.8, 128.8, 127.0, 122.9, 116.2, 111.0, 51.3, 39.1, 27.8, 22.5; **LCMS (ES+):** t_R = 0.71 min, $[M+H]^+$ 148.08; **ν_{max} (neat)**: 3064, 3019, 2928, 2814, 1601, 1505, 1320 cm^{-1}; **HRMS (ESI) m/z**: $[M+H]^+$ Calcd for $C_{10}H_{14}N$ 148.1121, found 148.1118.

1,7-Dimethyl-1,2,3,4-tetrahydroquinoline (**8b**)

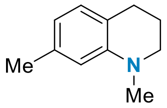

8b

This experiment followed General Procedure 8, using *O*-(2,4-dinitrophenyl)-*N*-methyl-*N*-(3-(*p*-tolyl)propyl)hydroxylamine (**7b**, 1.0 eq, 235 mg, 0.680 mmol), triflic acid (2.0 eq, 121 µL, 1.361 mmol) and $Ru(bpy)_3Cl_2.6H_2O$ (2 mol%, 10 mg, 0.014 mmmol). The crude product was purified on a 10 g Sfar Biotage column, eluting with 0-30% EtOAc:heptane (10 column volumes) to give 1,7-dimethyl-1,2,3,4-tetrahydroquinoline (**8b**) as a red oil (78.4 mg, 68%).

^{1}H NMR (400 MHz, $CDCl_3$, 303 K) δ (ppm) = 6.88 (d, *J* = 7.3 Hz, 1H), 6.50 - 6.44 (m, 2H), 3.27 - 3.20 (m, 2H), 2.92 (s, 3H), 2.77 (t, *J* = 6.4 Hz, 2H), 2.32 (s, 3H), 2.05 - 1.96 (m, 2H); **^{13}C NMR** (101 MHz, $CDCl_3$, 303 K) δ (ppm) = 146.6, 136.6, 128.7, 120.0, 117.0, 111.8, 51.4, 39.2, 27.5, 22.7, 21.6; **LCMS (ES+):** t_R = 0.77 min, $[M+H]^+$ 162.21; **ν_{max} (neat)**: 3012, 2923, 2811, 2838, 1714, 1611, 1576, 1509 cm^{-1}; **HRMS (ESI) m/z**: $[M+H]^+$ Calcd for $C_{11}H_{16}N$ 162.1277, found 162.1273.

7-(*tert*-Butyl)-1-methyl-1,2,3,4-tetrahydroquinoline (8c)

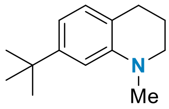

8c

This experiment followed General Procedure 8, using gave *N*-(3-(4-(*tert*-butyl)phenyl)propyl)-*O*-(2,4-dinitrophenyl)-*N*-methylhydroxylamine (**7c**, 1.0 eq, 169 mg, 0.384 mmol), triflic acid (2.0 eq, 68 µL, 0.768 mmol) and $Ru(bpy)_3Cl_2.6H_2O$ (2 mol%, 5.7 mg, 7.68 µmol). The crude product was purified on a 5 g Sfar Biotage column, eluting with 0-30% EtOAc:heptane (10 column volumes) to give 7-(*tert*-butyl)-1-methyl-1,2,3,4-tetrahydroquinoline (**8c**) as a pale yellow oil (67 mg, 86%).

^{1}H NMR (400 MHz, $CDCl_3$, 303 K) δ (ppm) = 6.93 (d, *J* = 7.3 Hz, 1H), 6.72 - 6.66 (m, 2H), 3.27 - 3.21 (m, 2H), 2.96 - 2.93 (m, 3H), 2.78 (t, *J* = 6.3 Hz, 2H), 2.07 - 1.95 (m, 2H), 1.34 (s, 9H); **^{13}C NMR** (101 MHz, $CDCl_3$, 303 K) δ (ppm) = 150.0, 146.4, 128.5, 120.1, 113.5, 108.4, 51.6, 39.3, 34.6, 31.5, 27.4, 22.6; **LCMS (ES+):** t_R = 1.18 min, $[M+H]^+$ 204.28; **ν_{max} (neat)**: 2950, 2864, 1686, 1610, 1514, 1313 cm^{-1}; **HRMS (ESI) m/z**: $[M+H]^+$ Calcd for $C_{14}H_{22}N$ 204.1747, found 204.1742.

1-Methyl-7-phenyl-1,2,3,4-tetrahydroquinoline and 1-methyl-6-phenyl-1,2,3,4-tetrahydroquinoline (8d' + 8d")

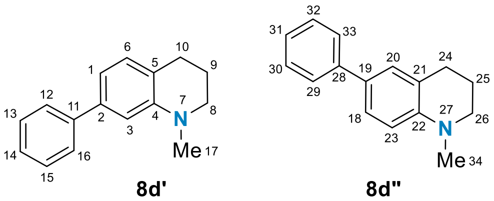

8d' **8d"**

This experiment followed General Procedure 11, using *N*-(3-([1,1'-biphenyl]-4-yl)propyl)-*O*-(2,4-dinitrophenyl)-*N*-methylhydroxylamine (**7d**, 1.0 eq, 105 mg, 0.193 mmol), triflic acid (2.0 eq, 34 µL,

0.387 mmol) and $Ru(bpy)_3Cl_2.6H_2O$ (2 mol%, 2.9 mg, 3.87 µmol). The crude product was purified on a 5 g Sfar Biotage column, eluting with 0-30% EtOAc:heptane (10 column volumes) to give 1-methyl-7-phenyl-1,2,3,4-tetrahydroquinoline and 1-methyl-6-phenyl-1,2,3,4-tetrahydroquinoline (**8d’** + **8d”**) as a yellow oil (41.2 mg, 95%) which comprised an inseparable mixture of isomers (1:1).

^{1}H NMR (600 MHz, $CDCl_3$, 303 K): δ (ppm) = 7.60 (d, *J* = 7.3 Hz, 2H, **8d’**: H-12, 16), 7.56 (d, *J* = 7.3 Hz, 2H, **8d”**: H-29, 33), 7.43 (t, *J* = 7.7 Hz, 2H, **8d’**: H-13, 15), 7.40 (t, *J* = 7.7 Hz, 2H, **8d”**: H-30, 32), 7.34 - 7.37 (m, 1H, **8d”**: H-18), 7.32 - 7.35 (m, 1H, **8d’**: H-14), 7.24 - 7.27 (m, 1H, **8d”**: H-31), 7.24 - 7.26 (m, 1H, **8d”**: H-20), 7.04 (d, *J* = 7.6 Hz, 1H, **8d’**: H-6), 6.85 (dd, *J* = 7.6, 1.5 Hz, 1H, **8d’**: H-1), 6.81 (s, 1H, **8d’**: H-3), 6.68 (d, *J* = 8.5 Hz, 1H, **8d”**: H-23), 3.27 - 3.30 (m, 2H, **8d”**: H-26), 3.27 - 3.30 (m, 2H, **8d’**: H-8), 2.98 (s, 3H, **8d’**: H-17), 2.95 (s, 3H, **8d”**: H-34), 2.84 - 2.87 (m, 2H, **8d”**: H-24), 2.82 - 2.85 (m, 2H, **8d’**: H-10), 2.01 - 2.07 (m, 4H, **8d’**: H-9, **8d”**: 25); **^{13}C NMR** (151 MHz, $CDCl_3$, 303 K): δ (ppm) = 146.9 (s, **8d’**: C-4), 146.2 (s, **8d”**: C-22), 142.3 (s, **8d’**: C-11), 141.4 (s, **8d”**: C-28), 140.4 (s, **8d’**: C-2), 129.1 (s, **8d’**: C-6), 128.9 (s, **8d”**: C-19), 128.5 (s, **8d”**: C-30, 32), 128.5 (s, **8d’**: C-13, 15), 127.5 (s, **8d”**: C-20), 127.1 (s, **8d’**: C-12, 16), 126.8 (s, **8d’**: C-14), 126.2 (s, **8d”**: C-29, 33), 125.8 (s, **8d”**: C-31), 125.6 (s, **8d”**: C-18), 123.0 (s, **8d”**: C-21), 122.0 (s, **8d’**: C-5), 115.2 (s, **8d’**: C-1), 111.2 (s, **8d”**: C-23), 109.8 (s, **8d’**: C-3), 51.3 (s, **8d’**: C-8), 51.3 (s, **8d”**: C-26), 39.2 (s, **8d’**: C-17), 39.1 (s, **8d”**: C-34), 27.9 (s, **8d”**: C-24), 27.5 (s, **8d’**: C-10), 22.4 (s, **8d’**: C-9, **8d”**: 25); **LCMS (ES+):** t_R = 1.38 min, $[M+H]^+$ 224.26; **$ν_{max}$ (neat)**: 3060, 3027, 2925, 2881, 2836, 1604, 1561 cm^{-1}; **HRMS (ESI) m/z**: $[M+H]^+$ Calcd for $C_{16}H_{18}N$ 224.1434, found 224.1427.

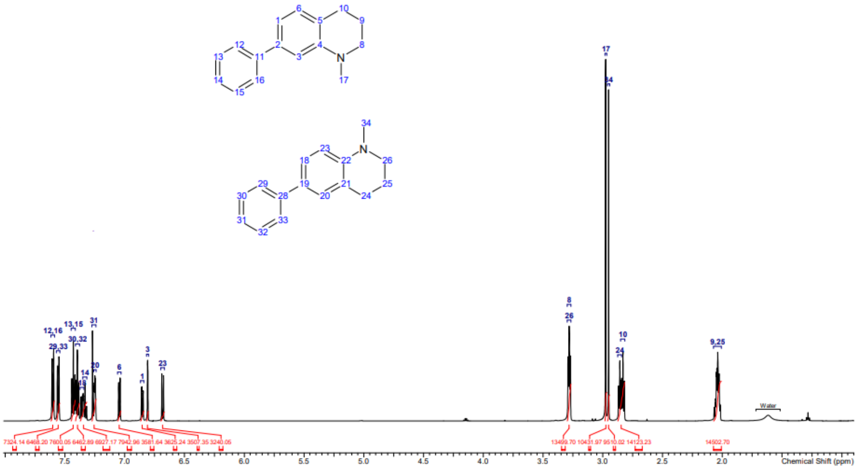

Figure 1. ^{1}H NMR of the regioisomers 1-methyl-7-phenyl-1,2,3,4-tetrahydroquinoline and 1-methyl-6-phenyl-1,2,3,4-tetrahydroquinoline.

COSY

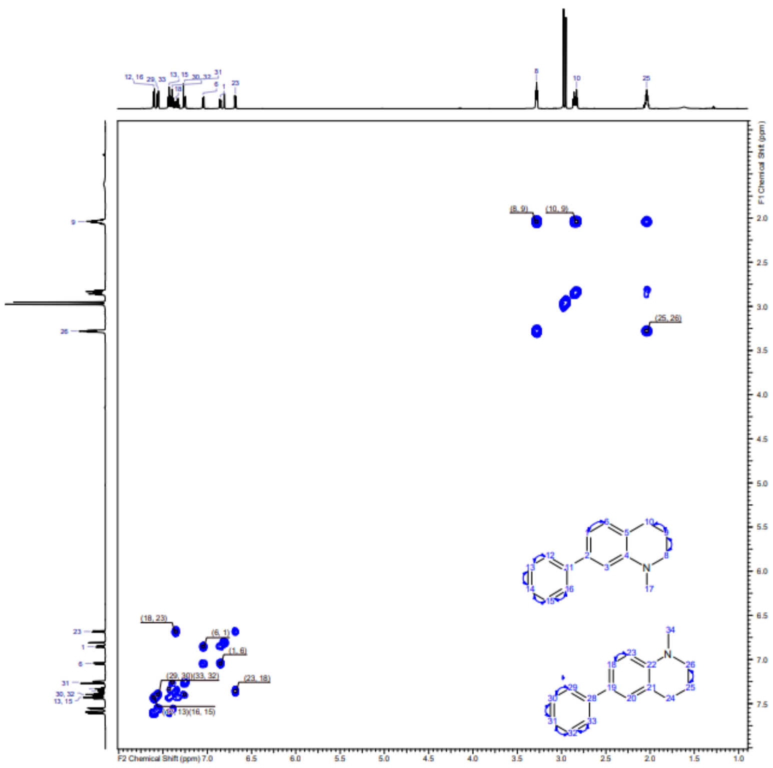

Figure 2. COSY spectra to determine the regioisomers 1-methyl-7-phenyl-1,2,3,4-tetrahydroquinoline and 1-methyl-6-phenyl-1,2,3,4-tetrahydroquinoline.

ROESY

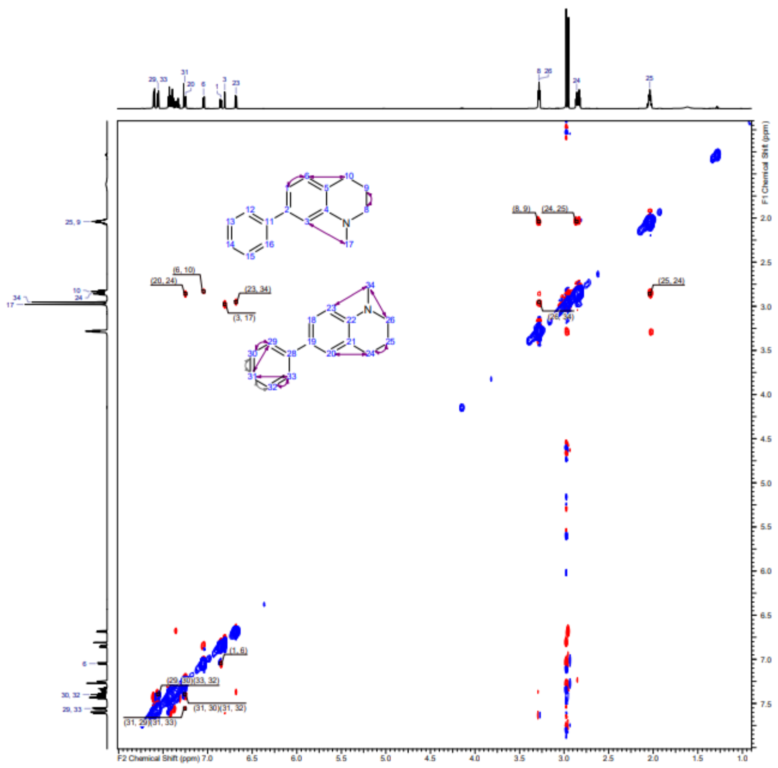

Figure 3. ROESY spectra to determine the regioisomers 1-methyl-7-phenyl-1,2,3,4-tetrahydroquinoline and 1-methyl-6-phenyl-1,2,3,4-tetrahydroquinoline.

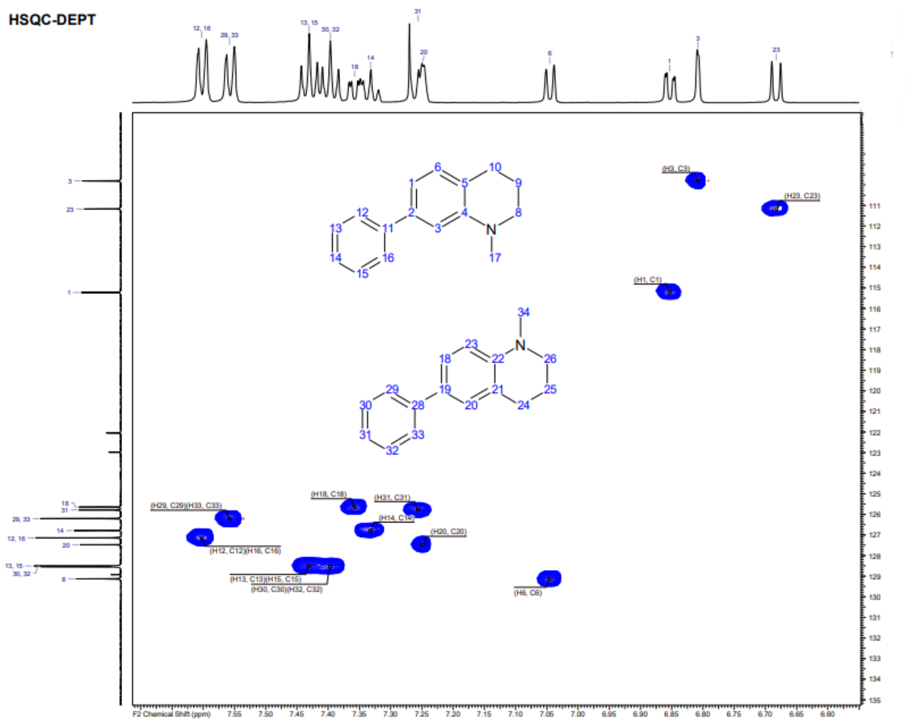

Figure 4. HSQC-DEPT to determine the regioisomers 1-methyl-7-phenyl-1,2,3,4-tetrahydroquinoline and 1-methyl-6-phenyl-1,2,3,4-tetrahydroquinoline.

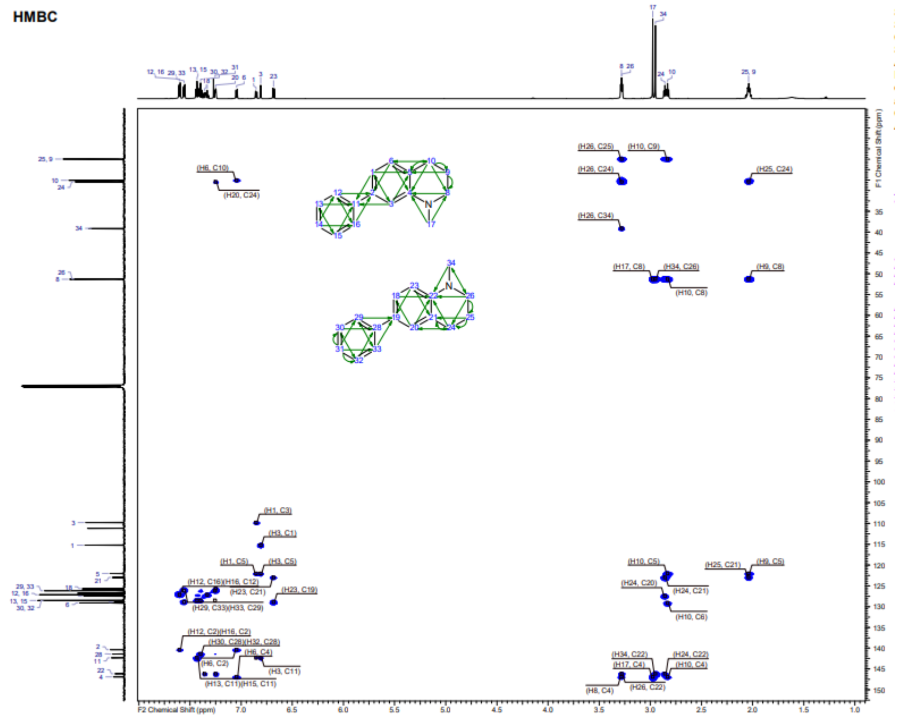

Figure 5. HMBC to determine the regioisomers 1-methyl-7-phenyl-1,2,3,4-tetrahydroquinoline and 1-methyl-6-phenyl-1,2,3,4-tetrahydroquinoline.

7-Methoxy-1-methyl-1,2,3,4-tetrahydroquinoline (**8e**)

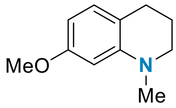

8e

This experiment followed General Procedure 8, using *O*-(2,4-dinitrophenyl)-*N*-(3-(4-methoxyphenyl)propyl)-*N*-methylhydroxylamine (**7e**, 1.0 eq, 163 mg, 0.45 mmol), triflic acid (2.0 eq, 80 µL, 0.90 mmol) and Ru(bpy)$_3$Cl$_2$.6H$_2$O (2 mol%, 6.7 mg, 9.0 µmol). The crude product was purified on a 10 g Sfar Biotage column, eluting with 0-40% Et$_2$O:pentane (10 column volumes) to give 7-methoxy-1-methyl-1,2,3,4-tetrahydroquinoline (**8e**) as an orange oil (56 mg, 70%).

^{1}H NMR (400 MHz, CDCl$_3$, 303 K) δ (ppm) = 6.88 (d, *J* = 8.3 Hz, 1H), 6.25 - 6.17 (m, 2H), 3.80 (s, 3H), 3.23 (t, *J* = 5.8 Hz, 2H), 2.90 (s, 3H), 2.73 (t, *J* = 6.6 Hz, 2H), 2.03 - 1.93 (m, 2H); **^{13}C NMR** (101 MHz, CDCl$_3$) δ 159.2, 147.6, 129.1, 115.7, 100.4, 97.8, 55.2, 51.1, 39.1, 27.1, 22.6; **LCMS (ES+):** t_R = 0.86 min, [M+H]$^+$ 178.20; **ν_{max} (neat)**: 2931, 2834, 1610, 1508 cm^{-1}; **HRMS (ESI) m/z**: [M+H]$^+$ Calcd for $C_{11}H_{16}NO$ 178.1226, found 178.1224.

7-Bromo-1-methyl-1,2,3,4-tetrahydroquinoline (**8f**)

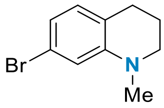

8f

This experiment followed General Procedure 8, using *N*-(3-(4-bromophenyl)propyl)-*O*-(2,4-dinitrophenyl)-*N*-methylhydroxylamine (**7f**, 1.0 eq, 88.2 mg, 0.215 mmol), triflic acid (2.0 eq, 38 µL, 0.430 mmol) and Ru(bpy)$_3$Cl$_2$.6H$_2$O (2 mol%, 3.2 mg, 4.3 µmol). The crude product was purified on a 10 g Sfar Biotage column, eluting with 0-30% EtOAc:heptane (10 column volumes) to give 7-bromo-1-methyl-1,2,3,4-tetrahydroquinoline (**8f**) as a pale yellow oil (35 mg, 72%).

^{1}H NMR (400 MHz, CDCl$_3$, 303 K) δ (ppm) = 6.84 - 6.78 (m, 1H), 6.74 - 6.68 (m, 2H), 3.29 - 3.21 (m, 2H), 2.89 (s, 3H), 2.71 (t, *J* = 6.4 Hz, 2H), 2.02 - 1.93 (m, 2H); **^{13}C NMR** (101 MHz, CDCl$_3$, 303 K) δ (ppm) = 147.7, 129.8, 121.5, 120.6, 118.5, 113.3, 50.9, 38.9, 27.4, 22.1; **LCMS (ES+):** t_R = 1.38 min, [M+H]$^+$ 226.22; **ν_{max} (neat)**: 2928, 2885, 2837, 1594, 1499 cm^{-1}; **HRMS (ESI) m/z**: [M+H]$^+$ Calcd for $C_{10}H_{13}{}^{79}BrN$ 226.0226, found 226.0223.

7-Chloro-1-methyl-1,2,3,4-tetrahydroquinoline (**8g**)

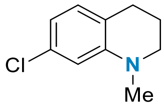

8g

This experiment followed General Procedure 8, using *N*-(3-(4-chlorophenyl)propyl)-*O*-(2,4-dinitrophenyl)-*N*-methylhydroxylamine (**7g**, 1.0 eq, 135 mg, 0.371 mmol), triflic acid (2.0 eq, 70 µL, 0.742 mmol) and Ru(bpy)$_3$Cl$_2$.6H$_2$O (2 mol%, 5.5 mg, 7.4 µmol). The crude product was purified on a 10 g Sfar Biotage column, eluting with 0-30% EtOAc:heptane (10 column volumes) to give 7-chloro-1-methyl-1,2,3,4-tetrahydroquinoline (**8g**) as a pale yellow oil (38 mg, 56%).

^{1}H NMR (400 MHz, CDCl$_3$, 303 K) δ (ppm) = 6.84 (d, *J* = 7.8 Hz, 1H), 6.58 - 6.52 (m, 2H), 3.26 - 3.21 (m, 2H), 2.88 (s, 3H), 2.71 (t, *J* = 6.4 Hz, 2H), 2.00 - 1.92 (m, 2H); **^{13}C NMR** (101 MHz, CDCl$_3$, 303 K) δ (ppm) = 147.5, 132.5, 129.5, 121.0, 115.5, 110.4, 50.9, 38.9, 27.3, 22.2; **LCMS (ES+):** t$_R$ = 1.34 min, [M+H]$_+$ 182.17; **ν_{max} (neat)**: 2928, 2887, 2837, 1599, 1564, 1501 cm^{-1}; **HRMS (ESI) m/z**: [M+H]$^+$ Calcd for C$_{10}$H$_{13}$^{35}ClN 182.0731, found 182.0729.

6-Methoxy-1-methyl-1,2,3,4-tetrahydroquinoline and 8-methoxy-1-methyl-1,2,3,4-tetrahydroquinoline (8h' + 8h'')

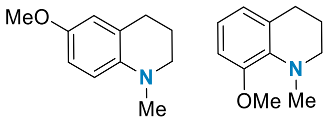

8h' **8h''**

This experiment followed General Procedure 8, using *O*-(2,4-dinitrophenyl)-*N*-(3-(3-methoxyphenyl)propyl)-*N*-methylhydroxylamine (**7h**, 1.0 eq, 128 mg, 0.336 mmol), triflic acid (2.0 eq, 60 µL, 0.672 mmol) and Ru(bpy)$_3$Cl$_2$.6H$_2$O (2 mol%, 5.0 mg, 6.72 µmol). The crude product was purified on a 10 g Sfar Biotage column, eluting with 0-40% EtOAc:heptane (10 column volumes) to give 6-methoxy-1-methyl-1,2,3,4-tetrahydroquinoline (**8h'**) as a light red oil (28 mg, 47%) and 8-methoxy-1-methyl-1,2,3,4-tetrahydroquinoline (**8h''**) as a light yellow oil (18 mg, 30%). The data for the products are listed respectively.

6-Methoxy-1-methyl-1,2,3,4-tetrahydroquinoline (**8h'**): **^{1}H NMR** (400 MHz, CDCl$_3$, 303 K) δ (ppm) = 6.71 (dd, *J* = 2.9, 9.0 Hz, 1H), 6.66 - 6.56 (m, 2H), 3.77 (s, 3H), 3.20 - 3.12 (m, 2H), 2.87 (s, 3H), 2.79 (t, *J* = 6.6 Hz, 2H), 2.07 - 1.96 (m, 2H); **^{13}C NMR** (101 MHz, CDCl$_3$, 303 K) δ (ppm) = 151.4, 141.6, 124.7, 115.1, 112.5, 112.3, 55.8, 51.7, 39.9, 28.0, 22.7; **LCMS (ES+):** t$_R$ = 0.41 min, [M+H]$_+$ 178.18; **ν_{max} (neat)**: 2932, 2827, 2800, 1578, 1505 cm^{-1}; **HRMS (ESI) m/z**: [M+H]$^+$ Calcd for C$_{11}$H$_{16}$NO 178.1226, found 178.1224.

8-Methoxy-1-methyl-1,2,3,4-tetrahydroquinoline (**8h''**): **^{1}H NMR** (400 MHz, CDCl$_3$, 303 K) δ (ppm) = 6.88 (t, *J* = 7.8 Hz, 1H), 6.74 - 6.66 (m, 2H), 3.89 (s, 3H), 3.20 - 3.13 (m, 2H), 2.87 (s, 3H), 2.80 (t, *J* = 6.6 Hz, 2H), 1.91 - 1.82 (m, 2H); **^{13}C** NMR (101 MHz, CDCl$_3$, 303 K) δ (ppm) = 152.2, 137.9, 129.6, 121.9, 121.2, 108.5, 55.3, 52.5, 42.3, 27.9, 17.6; **LCMS (ES+):** t$_R$ = 0.35 min, [M+H]$_+$ 178.19; **ν_{max} (neat)**: 3001,

2932, 2834, 2794, 1575, 1452, 1480 cm^{-1}; **HRMS (ESI) m/z**: $[M+H]^+$ Calcd for $C_{11}H_{16}NO$ 178.1226, found 178.1223.

6-Bromo-1-methyl-1,2,3,4-tetrahydroquinoline (**8i**)

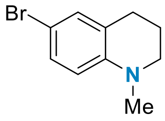

8i

This experiment followed General Procedure 8, using *N*-(3-(4-bromophenyl)propyl)-*O*-(2,4-dinitrophenyl)-*N*-methylhydroxylamine (**7i**, 1.0 eq, 92.5 mg, 0.225 mmol), triflic acid (2.0 eq, 40 µL, 0.451 mmol) and $Ru(bpy)_3Cl_2.6H_2O$ (2 mol%, 3.4 mg, 4.51 µmol). The crude product was purified on a 10 g Sfar Biotage column, eluting with 0-30% EtOAc:heptane (10 column volumes) to give 6-bromo-1-methyl-1,2,3,4-tetrahydroquinoline (**8i**) as a pale yellow oil (31 mg, 61%).

^{1}H NMR (400 MHz, $CDCl_3$, 300 K) δ (ppm) = 7.16 (dd, *J* = 2.5, 8.9 Hz, 1H), 7.08 - 7.05 (m, 1H), 6.46 (d, *J* = 8.6 Hz, 1H), 3.27 - 3.20 (m, 2H), 2.88 (s, 3H), 2.75 (t, *J* = 6.5 Hz, 2H), 2.04 - 1.93 (m, 2H); **^{13}C NMR** (101 MHz, $CDCl_3$, 300 K) δ (ppm) = 145.7, 131.1, 129.6, 124.9, 112.4, 107.8, 51.1, 39.0, 27.7, 22.2; **LCMS (ES+):** t_R = 1.36 min, $[M+H]_+$ 226.2; **$ν_{max}$ (neat)**: 2931, 2874, 2819, 1593, 1498, 1321 cm^{-1}; **HRMS (ESI) m/z**: $[M+H]^+$ Calcd for $C_{10}H_{13}{}^{79}BrN$ 226.0226, found 226.0220.

1,5-Dimethyl-1,2,3,4-tetrahydroquinoline (**8j**)

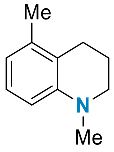

8j

This experiment followed General Procedure 8, using *O*-(2,4-dinitrophenyl)-*N*-methyl-*N*-(3-(*p*-tolyl)propyl)hydroxylamine (**7j**, 1.0 eq, 286 mg, 0.828 mmol), triflic acid (2.0 eq, 147 µL, 1.656 mmol) and $Ru(bpy)_3Cl_2.6H_2O$ (2 mol%, 12.4 mg, 0.017 mmol). The crude product was purified on a 10 g Sfar Biotage column, eluting with 0-30% EtOAc:heptane (10 column volumes) to give 1,5-dimethyl-1,2,3,4-tetrahydroquinoline (**8j**) as a red oil (60.1 mg, 45%).

^{1}H NMR (400 MHz, $CDCl_3$, 303 K) δ (ppm) = 7.03 (t, *J* = 7.8 Hz, 1H), 6.56 (m, 2H), 3.23 - 3.18 (m, 2H), 2.92 (s, 3H), 2.69 (t, *J* = 6.6 Hz, 2H), 2.23 (s, 3H), 2.10 - 2.02 (m, 2H); **^{13}C NMR** (101 MHz, $CDCl_3$, 303 K) δ (ppm) = 147.2, 136.4, 126.3, 121.5, 118.6, 109.4, 51.0, 39.9, 24.7, 22.5, 19.8; **LCMS (ES+):** t_R = 0.64 min, $[M+H]_+$ 162.21; **$ν_{max}$ (neat)**: 3115, 3060, 2934, 2864, 1601, 1522, 1338 cm^{-1}; **HRMS (ESI) m/z**: $[M+H]^+$ Calcd for $C_{11}H_{16}N$ 162.1277, found 162.1274.

5-Fluoro-1-methyl-1,2,3,4-tetrahydroquinoline (**8k**)

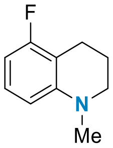

8k

This experiment followed General Procedure 8, using *N*-(3-(2-fluorophenyl)propyl)-*O*-(2,4-dinitrophenyl)-*N*-methylhydroxylamine (**7k**, 1.0 eq, 158 mg, 0.453 mmol), triflic acid (2.0 eq, 80 µL, 0.907 mmol) and Ru(bpy)$_3$Cl$_2$.6H$_2$O (2 mol%, 6.79 mg, 9.07 µmol). The crude product was purified on a 10 g Sfar Biotage column, eluting with 0-30% EtOAc:heptane (10 column volumes) to give 5-fluoro-1-methyl-1,2,3,4-tetrahydroquinoline (**8k**) as a pale yellow oil (33 mg, 44%).

^{1}H NMR (400 MHz, CDCl$_3$, 303 K) δ (ppm) = 7.03 (m, 1H), 6.43 - 6.34 (m, 2H), 3.26 - 3.22 (m, 2H), 2.93 (s, 3H), 2.77 (t, *J* = 6.6 Hz, 2H), 2.05 - 1.96 (m, 2H); **^{13}C NMR** (101 MHz, CDCl$_3$, 303 K) δ = 161.0 (d, *J* = 239.9 Hz), 148.3 (d, *J* = 8.1 Hz), 127.1 (d, *J* = 11.0 Hz), 109.6 (d, *J* = 21.3 Hz), 106.4 (d, *J* = 2.9 Hz), 102.8 (d, *J* = 22.7 Hz), 50.7, 39.5, 21.5, 20.1 (d, *J* = 5.1 Hz); **^{19}F NMR** (376 MHz, CDCl$_3$, 303 K) δ (ppm) = −118.46 (s, 1F); **LCMS (ES+):** t_R = 1.22 min, [M+H]$^+$ 166.22; **ν_{max} (neat)**: 2938, 2843, 2818, 2864, 1618, 1570, 1500 cm^{-1}; **HRMS (ESI) m/z**: [M+H]$^+$ Calcd for $C_{10}H_{13}FN$ 166.1027, found 166.1024.

5-Bromo-1-methyl-1,2,3,4-tetrahydroquinoline (**8l**)

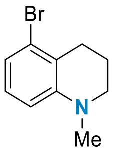

8l

This experiment followed General Procedure 8, using *N*-(3-(2-bromophenyl)propyl)-*O*-(2,4-dinitrophenyl)-*N*-methylhydroxylamine (**7l**, 1.0 eq, 240 mg, 0.585 mmol), triflic acid (2.0 eq, 104 µL, 1.170 mmol) and Ru(bpy)$_3$Cl$_2$.6H$_2$O (2 mol%, 8.76 mg, 0.012 mmol). The crude product was purified on a 10 g Sfar Biotage column, eluting with 0-30% EtOAc:heptane (10 column volumes) to give 5-bromo-1-methyl-1,2,3,4-tetrahydroquinoline (**8l**) as an orange oil (97 mg, 73%).

^{1}H NMR (400 MHz, CDCl$_3$, 303 K) δ (ppm) = 6.97 - 6.88 (m, 2H), 6.55 (dd, *J* = 1.5, 7.8 Hz, 1H), 3.26 - 3.20 (m, 2H), 2.92 (s, 3H), 2.83 (t, *J* = 6.6 Hz, 2H), 2.06 - 1.98 (m, 2H); **^{13}C NMR** (101 MHz, CDCl$_3$, 303 K) δ (ppm) = 148.4, 127.7, 125.5, 122.0, 120.2, 110.0, 50.9, 39.5, 28.5, 22.3; **LCMS (ES+):** t_R = 1.36 min, [M+H]$^+$ 226.14; **ν_{max} (neat)**: 2943, 2859, 2818, 1588, 1557, 1488, 1320 cm^{-1}; **HRMS (ESI) m/z**: [M+H]$^+$ Calcd for $C_{10}H_{13}{}^{79}BrN$ 226.0226, found 226.0223.

1,4-Dimethyl-3,4-dihydro-2*H*-quinoline (8m)

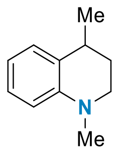

8m

This experiment followed General Procedure 8, using *O*-(2,4-dinitrophenyl)-*N*-methyl-*N*-(3-phenylbutyl)hydroxylamine (**7m**, 1.0 eq, 198 mg, 0.545 mmol), triflic acid (2.0 eq, 96 µL, 1.089 mmol) and Ru(bpy)$_3$Cl$_2$.6H$_2$O (2 mol%, 8.0 mg, 0.011 mmol). The crude product was purified on a 10 g Sfar Biotage column, eluting with 0-30% EtOAc:heptane (10 column volumes) to give 1,4-dimethyl-3,4-dihydro-2*H*-quinoline (**8m**) as a red oil (62 mg, 71%).

^{1}H NMR (400 MHz, CDCl$_3$, 303 K) δ (ppm) = 7.15 - 7.05 (m, 2H), 6.67 (t, *J* = 7.3 Hz, 1H), 6.63 (d, *J* = 8.3 Hz, 1H), 3.34 - 3.17 (m, 2H), 2.98 - 2.88 (m, 4H), 2.07 (tdd, *J* = 4.9, 8.3, 13.2 Hz, 1H), 1.73 (dtd, *J* = 4.2, 6.4, 12.9 Hz, 1H), 1.32 (d, *J* = 6.8 Hz, 3H); **^{13}C NMR** (101 MHz, CDCl$_3$, 303 K) δ (ppm) = 146.1, 128.0, 127.8, 127.0, 116.1, 110.9, 48.3, 39.2, 30.8, 30.0, 22.7; **LCMS (ES+):** t_R =0.95 min, [M+H]+ 162.09; **ν_{max} (neat)**: 3066, 3017, 2935, 2818, 1601, 1502 cm^{-1}; **HRMS (ESI) m/z**: [M+H]$^+$ Calcd for $C_{11}H_{16}N$ 162.1277, found 162.1270.

1-Methyl-4-phenyl-3,4-dihydro-2*H*-quinoline (8n)

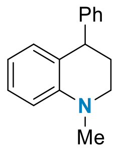

8n

This experiment followed General Procedure 8, using *O*-(2,4-dinitrophenyl)-*N*-(3,3-diphenylpropyl)-*N*-methylhydroxylamine (**7n**, 1.0 eq, 74 mg, 0.16 mmol), triflic acid (2.0 eq, 28 µL, 0.32 mmol) and Ru(bpy)$_3$Cl$_2$.6H$_2$O (2 mol%, 2.0 mg, 3.0 µmol). The crude product was purified on a 10 g Sfar Biotage column, eluting with 0-30% EtOAc:heptane (10 column volumes) to give 1-methyl-4-phenyl-3,4-dihydro-2*H*-quinoline (**8n**) as a colourless oil (17.4 mg, 42%).

^{1}H NMR (400 MHz, CDCl$_3$, 303 K) δ (ppm) = 7.36 - 7.29 (m, 2H), 7.23 (tt, 1H), 7.18 - 7.12 (m, 3H), 6.77 (td, *J* = 1.4, 7.5 Hz, 1H), 6.71 (d, *J* = 7.3 Hz, 1H), 6.60 (dt, *J* = 1.2, 7.5 Hz, 1H), 4.17 (t, *J* = 6.1 Hz, 1H), 3.30 - 3.14 (m, 2H), 2.98 (s, 3H), 2.34 - 2.25 (m, 1H), 2.19 - 2.09 (m, 1H); **^{13}C NMR** (101 MHz, CDCl$_3$, 303 K) δ (ppm) = 146.9, 146.6, 129.9, 128.7, 128.3, 127.6, 126.1, 124.8, 116.2, 111.0, 48.5, 43.4, 39.3, 31.1; **LCMS (ES+):** t_R =1.35 min, [M+H]+ 224.18; **ν_{max} (neat)**: 3064, 3023, 2922, 2858, 1600, 1502 cm^{-1}; **HRMS (ESI) m/z**: [M+H]$^+$ Calcd for $C_{16}H_{18}N$ 224.1434, found 224.1429.

4-Methyl-3,4-dihydro-2*H*-benzo[*b*][1,4]oxazine (**8o**)

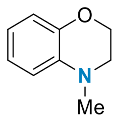

8o

This experiment followed General Procedure 8, using *O*-(2,4-dinitrophenyl)-*N*-methyl-*N*-(2-phenoxyethyl)hydroxylamine (**7o**, 1.0 eq, 78.8 mg, 0.236 mmol), triflic acid (2.0 eq, 42 µL, 0.473 mmol) and $Ru(bpy)_3Cl_2.6H_2O$ (2 mol%, 3.5 mg, 4.73 µmol). The crude product was purified on a 10 g Sfar Biotage column, eluting with 0-30% EtOAc:heptane (10 column volumes) to give 4-methyl-3,4-dihydro-2*H*-benzo[*b*][1,4]oxazine (**8o**) as a yellow oil (23.5 mg, 66%).

^{1}H NMR (400 MHz, $CDCl_3$, 303 K) δ (ppm) = 6.91 - 6.84 (m, 1H), 6.80 (dd, *J* = 1.5, 7.8 Hz, 1H), 6.73 - 6.65 (m, 2H), 4.36 - 4.31 (m, 2H), 3.33 - 3.27 (m, 2H), 2.91 (s, 3H); **^{13}C NMR** (101 MHz, $CDCl_3$, 303 K) δ (ppm) = 144.3, 136.6, 121.4, 118.2, 115.9, 112.6, 64.9, 49.3, 38.8; **LCMS (ES+):** t_R = 0.93 min, $[M+H]_+$ 150.05; **ν_{max} (neat)**: 3039, 2925, 2871, 2817, 1605, 1501, 1307 cm^{-1}; **HRMS (ESI) m/z**: $[M+H]^+$ Calcd for $C_9H_{12}NO$ 150.0913, found 150.0907.

7. References

1. Svejstrup, T. D.; Ruffoni, A.; Juliá, F.; Aubert, V. M.; Leonori, D., Synthesis of Arylamines via Aminium Radicals. *Angew. Chem. Int. Ed.* **2017,** *56*, 14948–14952.
2. Hidalgo-Acosta, J. C.; Méndez, M. A.; Scanlon, M. D.; Vrubel, H.; Amstutz, V.; Adamiak, W.; Opallo, M.; Girault, H. H., Catalysis of water oxidation in acetonitrile by iridium oxide nanoparticles. *Chem. Sci.* **2015,** *6*, 1761-1769.
3. Ruffoni, A.; Juliá, F.; Svejstrup, T. D.; McMillan, A. J.; Douglas, J. J.; Leonori, D., Practical and regioselective amination of arenes using alkyl amines. *Nat. Chem.* **2019,** *11*, 426–433.
4. Girard, S. A.; Hu, X.; Knauber, T.; Zhou, F.; Simon, M.-O.; Deng, G.-J.; Li, C.-J., Pd-Catalyzed Synthesis of Aryl Amines via Oxidative Aromatization of Cyclic Ketones and Amines with Molecular Oxygen. *Org. Lett.* **2012,** *14*, 5606–5609.
5. Takasu, N.; Oisaki, K.; Kanai, M., Iron-Catalyzed Oxidative C(3)–H Functionalization of Amines. *Organic Letters* **2013,** *15*, 1918–1921.
6. Gurjar, J.; Fokin, V. V., Sulfuryl Fluoride Mediated Synthesis of Amides and Amidines from Ketoximes via Beckmann Rearrangement. *Chem. Eur. J.* **2020,** *26*, 10402–10405.
7. Bouarfa, S.; Graßl, S.; Ivanova, M.; Langlais, T.; Bentabed-Ababsa, G.; Lassagne, F.; Erb, W.; Roisnel, T.; Dorcet, V.; Knochel, P.; Mongin, F., Copper- and Cobalt-Catalyzed Syntheses of Thiophene-Based Tertiary Amines. *European Journal of Organic Chemistry* **2019,** 3244–3258.
8. Itooka, R.; Iguchi, Y.; Miyaura, N., Rhodium-Catalyzed 1,4-Addition of Arylboronic Acids to α,β-Unsaturated Carbonyl Compounds: Large Accelerating Effects of Bases and Ligands. *J. Org. Chem.* **2003,** *68*, 6000–6004.
9. Lin, S.; Lu, X., Palladium–bipyridine catalyzed conjugate addition of arylboronic acids to α,β-unsaturated carbonyl compounds in aqueous media. *Tetrahedron Lett.* **2006,** *47*, 7167–7170.
10. Huang, C.; Ma, W.; Zheng, X.; Xu, M.; Qi, X.; Lu, Q., Epoxide Electroreduction. *J. Am. Chem. Soc.* **2022,** *144*, 1389–1395.
11. Kelly, C. B.; Mercadante, M. A.; Wiles, R. J.; Leadbeater, N. E., Oxidative Esterification of Aldehydes Using a Recyclable Oxoammonium Salt. *Org. Lett.* **2013,** *15*, 2222-2225.

12. Groendyke, B. J.; Modak, A.; Cook, S. P., Fenton-Inspired C–H Functionalization: Peroxide-Directed C–H Thioetherification. *J. Org. Chem.* **2019,** *84*, 13073−13091.
13. Pinna, G. A.; Cignarella, G.; Loriga, G.; Murineddu, G.; Mussinu, J.-M.; Ruiu, S.; Fadda, P.; Fratta, W., N-3(9)-Arylpropenyl-N-9(3)-propionyl-3,9-diazabicyclo[3.3.1]nonanes as μ-Opioid receptor agonists. Effects on μ-Affinity of arylalkenyl chain modifications. *Biorg. Med. Chem.* **2002,** *10*, 1929–1937.

8. NMR Spectra

1-(2,4-dinitrophenoxy)piperidine (**3**)

Solvent: $CDCl_3$, ^1H NMR (400 MHz), ^{13}C NMR (101 MHz).

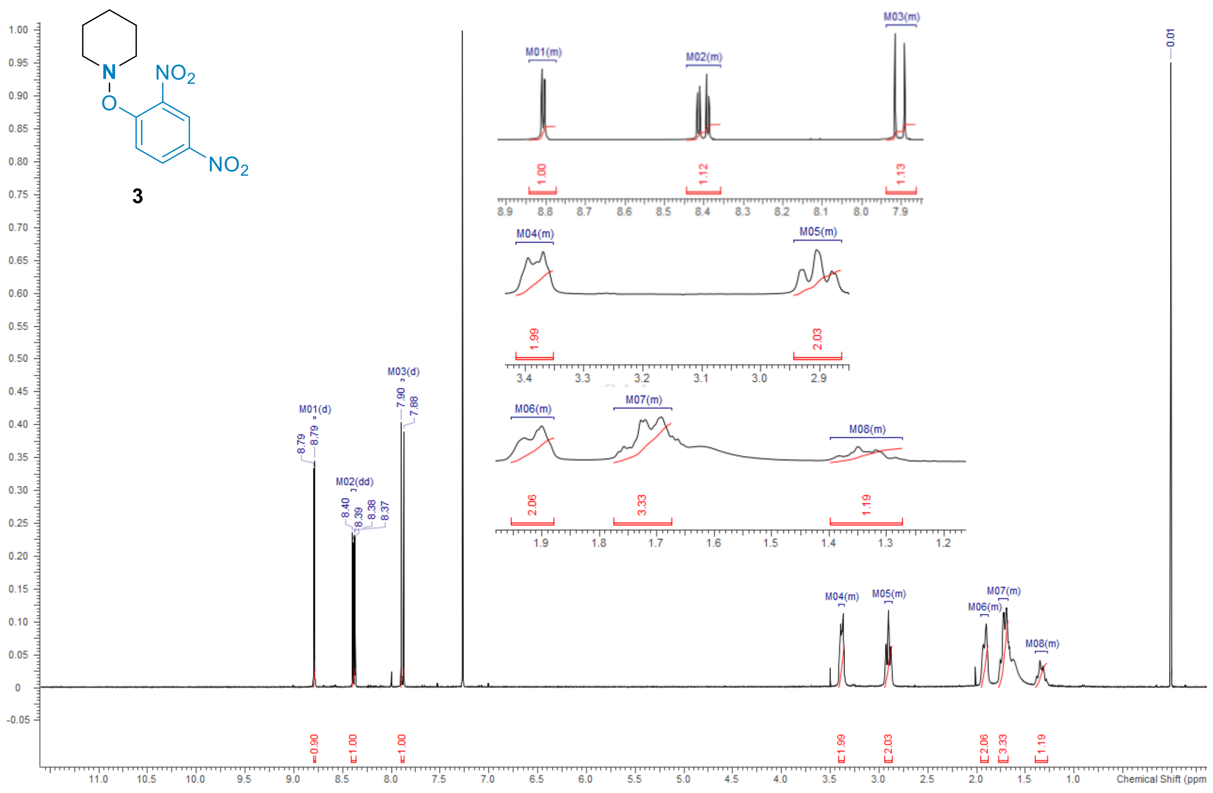

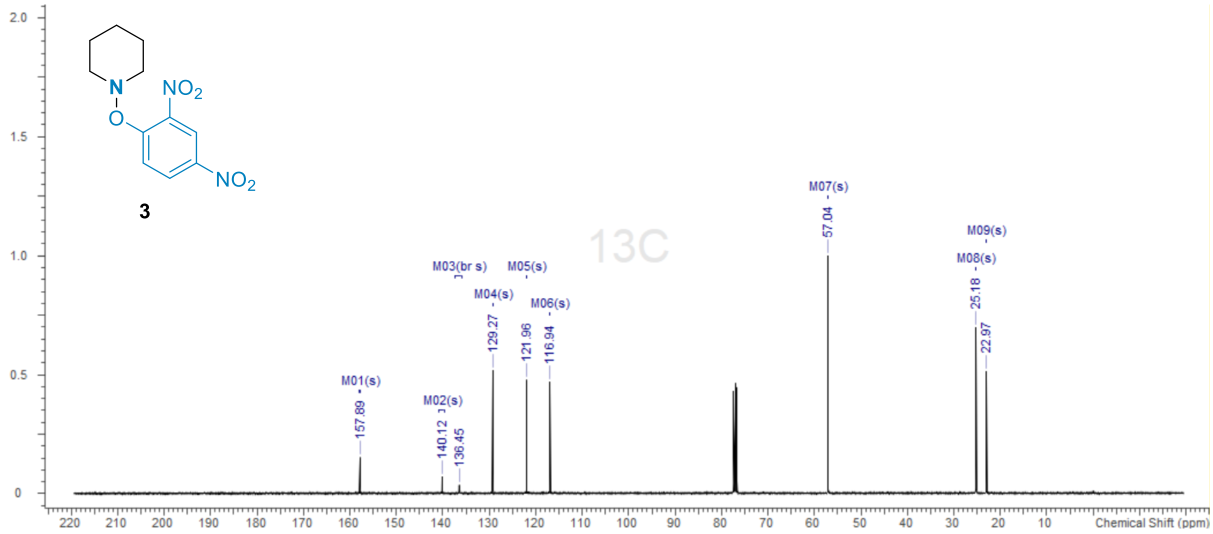

1-(4-(*tert*-Butyl)phenyl)piperidine and (**4’**)

Solvent: $CDCl_3$, ^1H NMR (400 MHz), ^{13}C NMR (101 MHz).

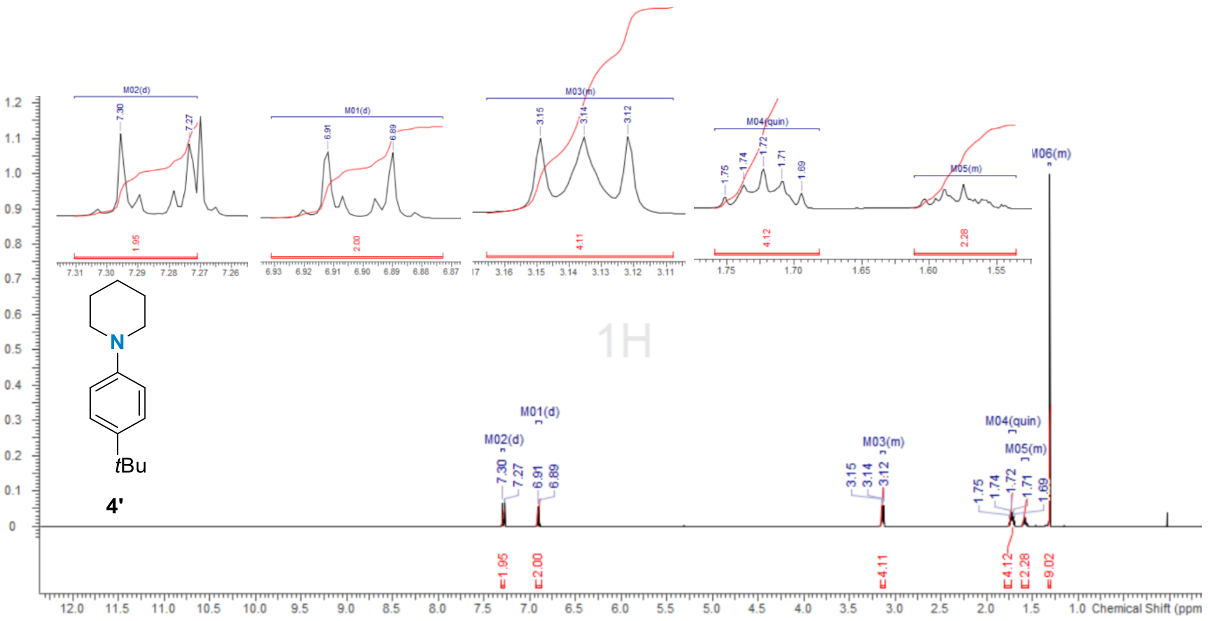

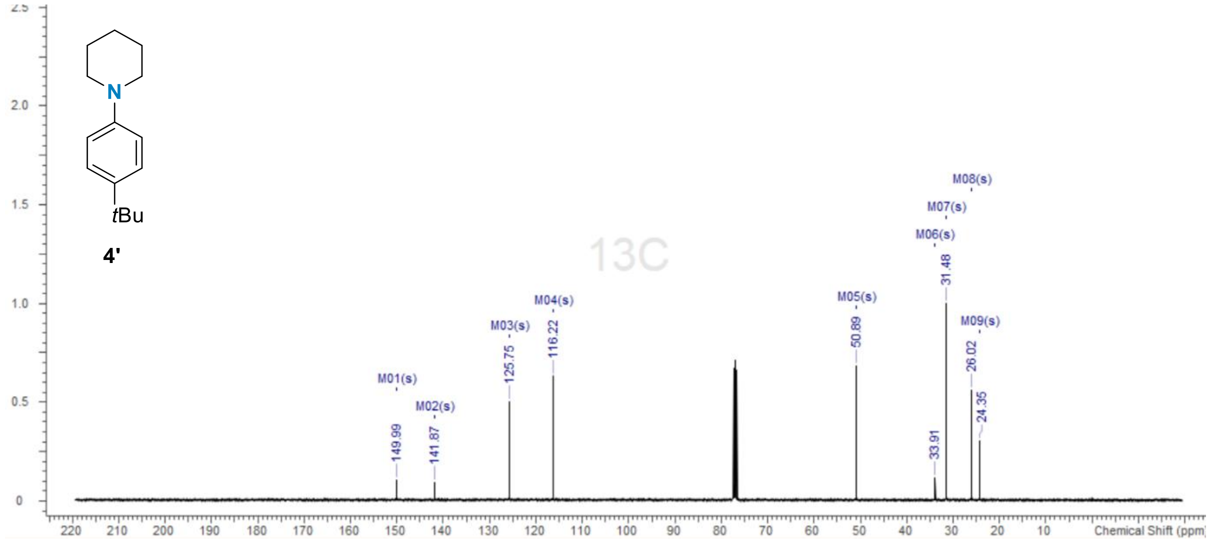

1-(3-(*tert*-Butyl)phenyl)piperidine (**4”**)

Solvent: $CDCl_3$, ^{1}H NMR (400 MHz), ^{13}C NMR (101 MHz).

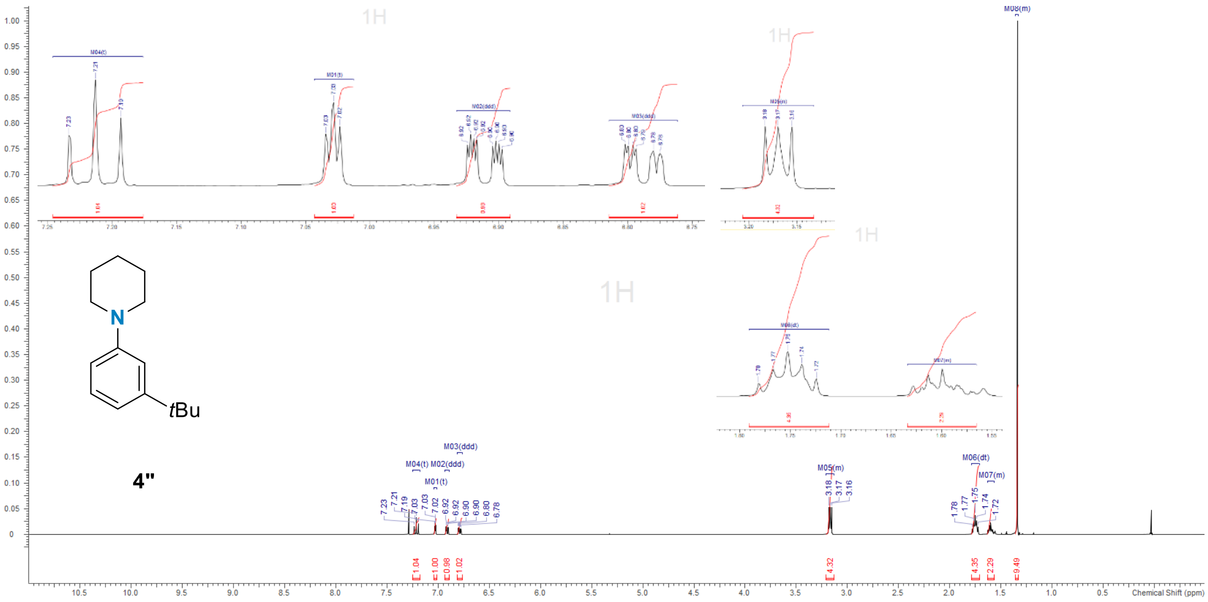

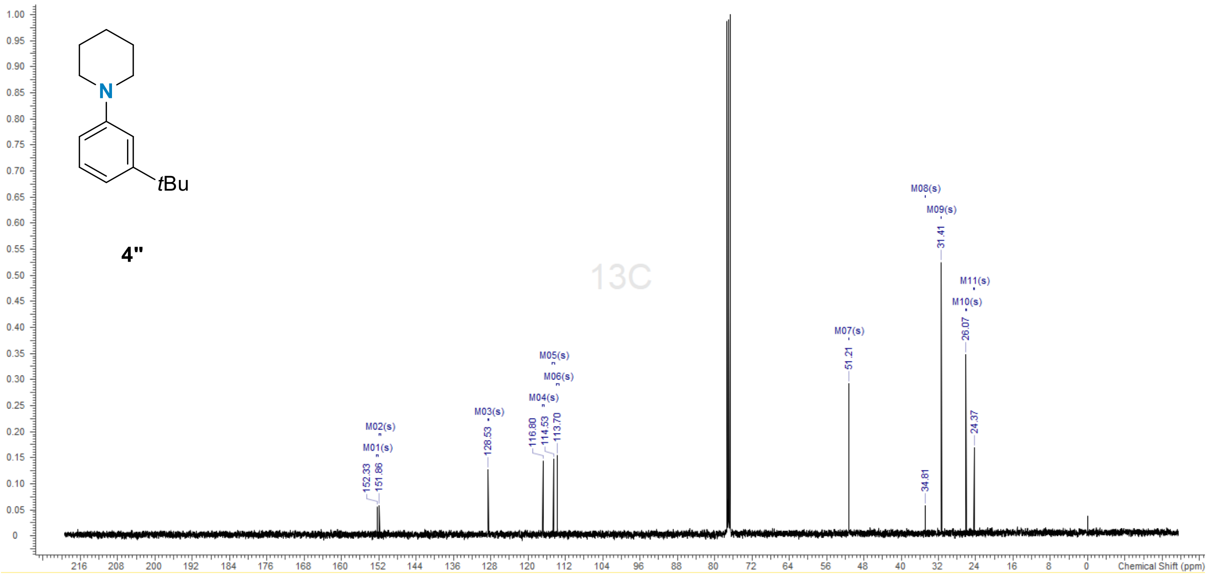

1-(4-Methoxyphenyl)piperidine and 1-(2-methoxyphenyl)piperidine (5’ + 5”)

Solvent: $CDCl_3$, ^1H NMR (400 MHz), ^{13}C NMR (101 MHz).

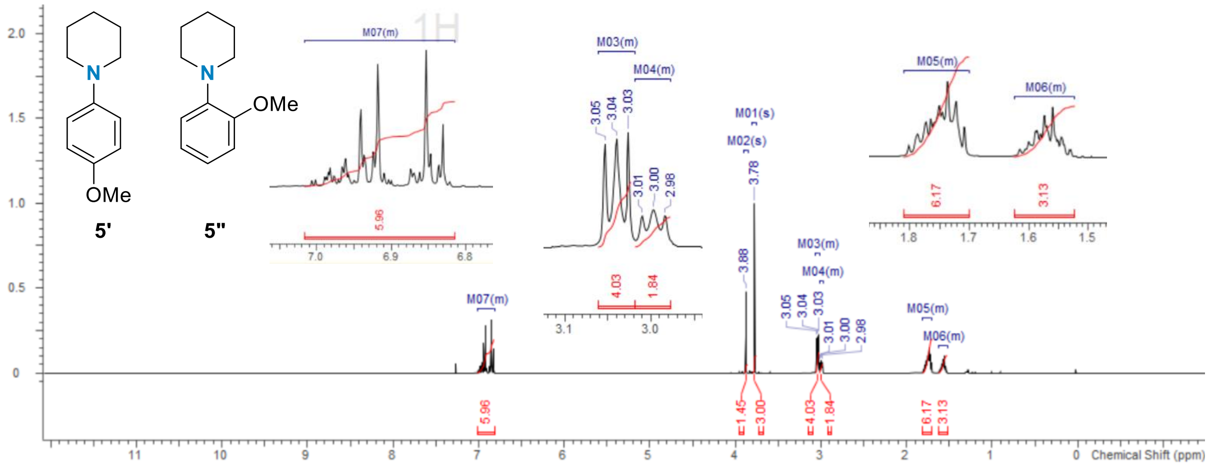

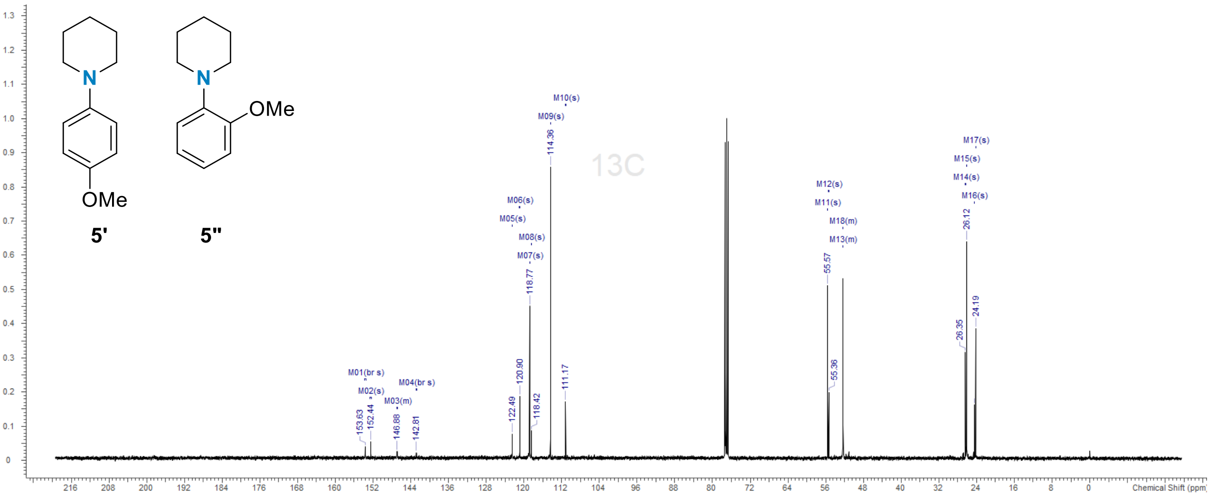

N-(4-(Piperidin-1-yl)phenyl)acetamide (**6a**)

Solvent: $CDCl_3$, ^1H NMR (400 MHz), ^{13}C NMR (101 MHz).

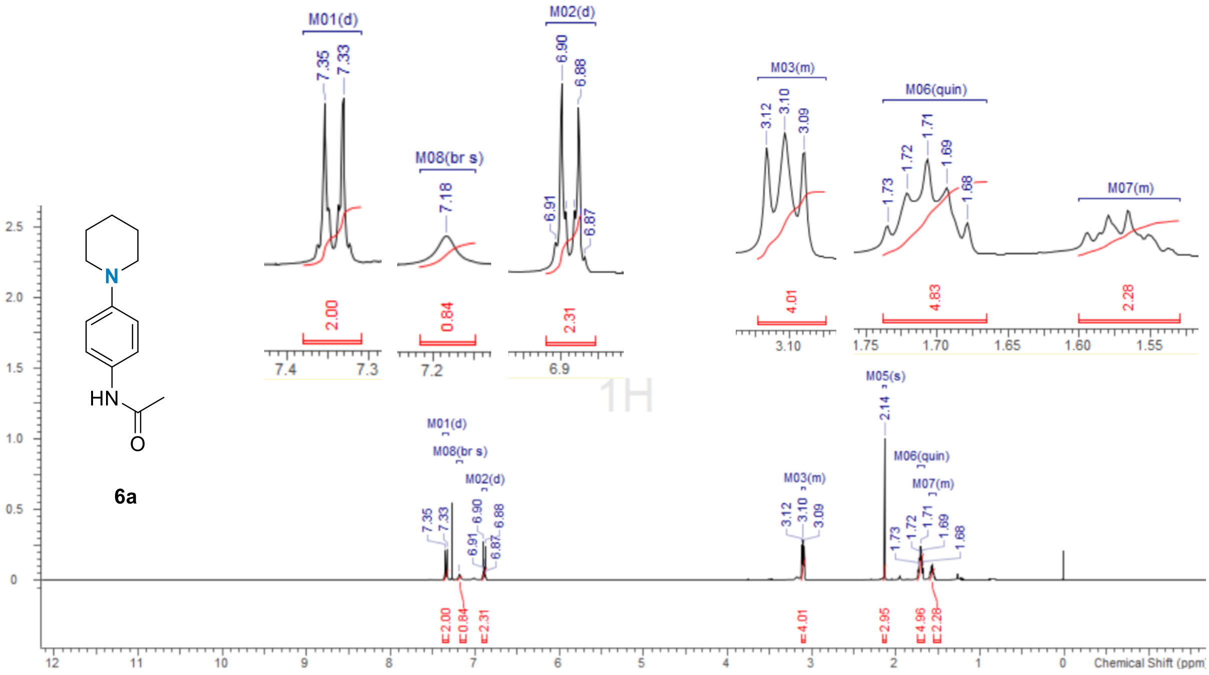

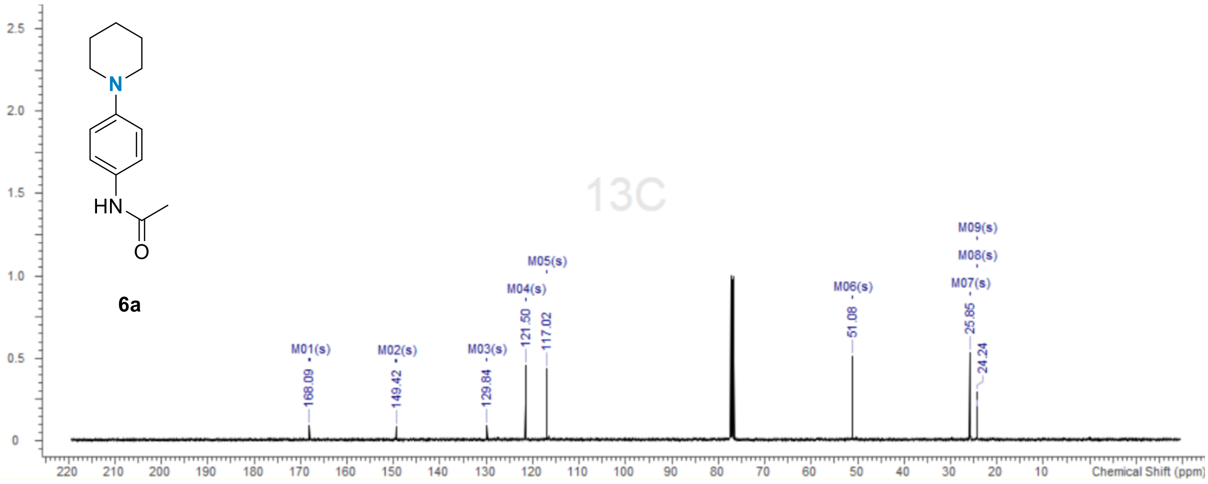

1-(5-Bromo-2-methoxyphenyl)piperidine (**6b**)

Solvent: $CDCl_3$, ^{1}H NMR (400 MHz), ^{13}C NMR (101 MHz).

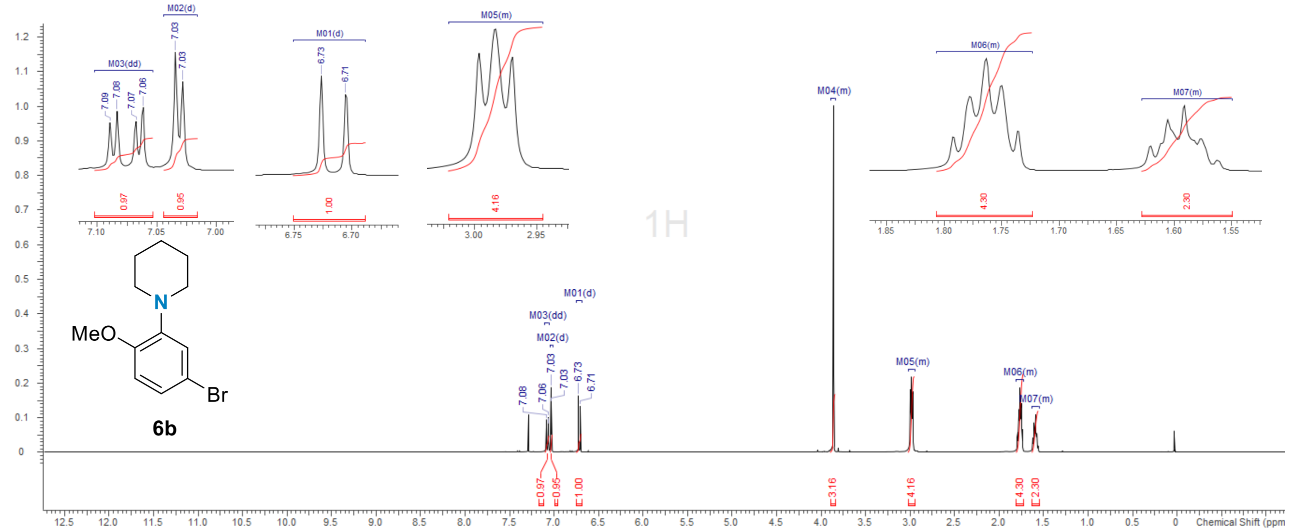

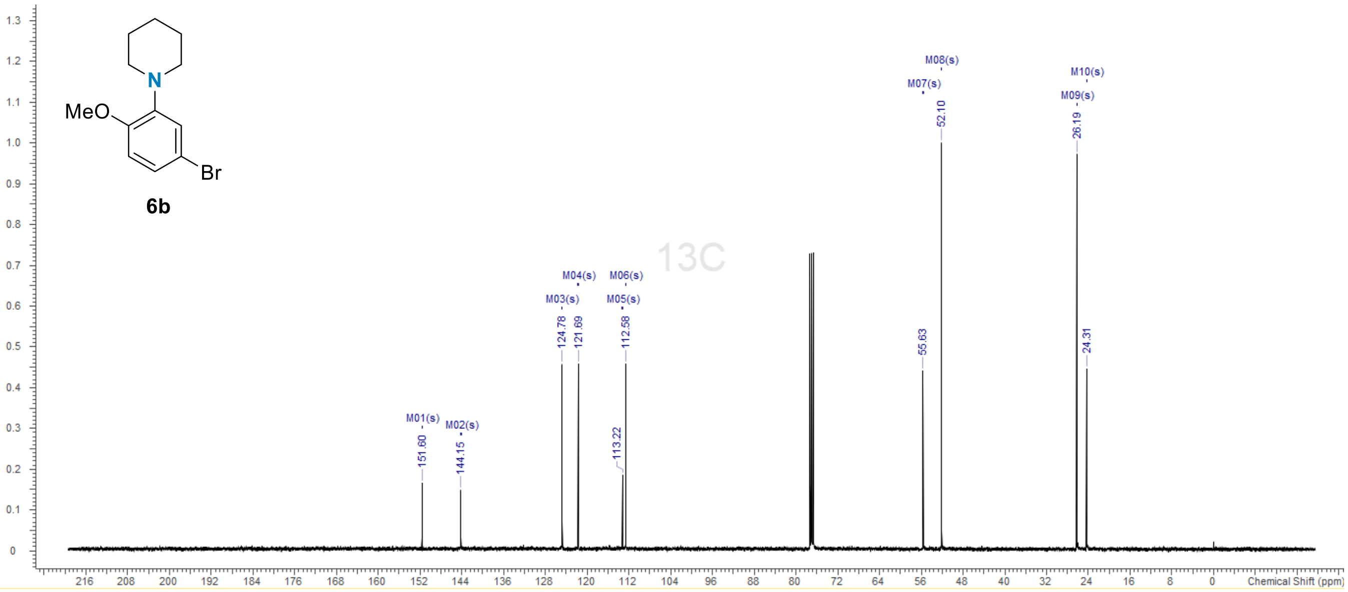

1-(2-Methoxy-5-(4,4,5,5-tetramethyl-1,3,2-dioxaborolan-2-yl)phenyl)piperidine (**6c**)

Solvent: $CDCl_3$, ^1H NMR (400 MHz), ^{13}C NMR (101 MHz).

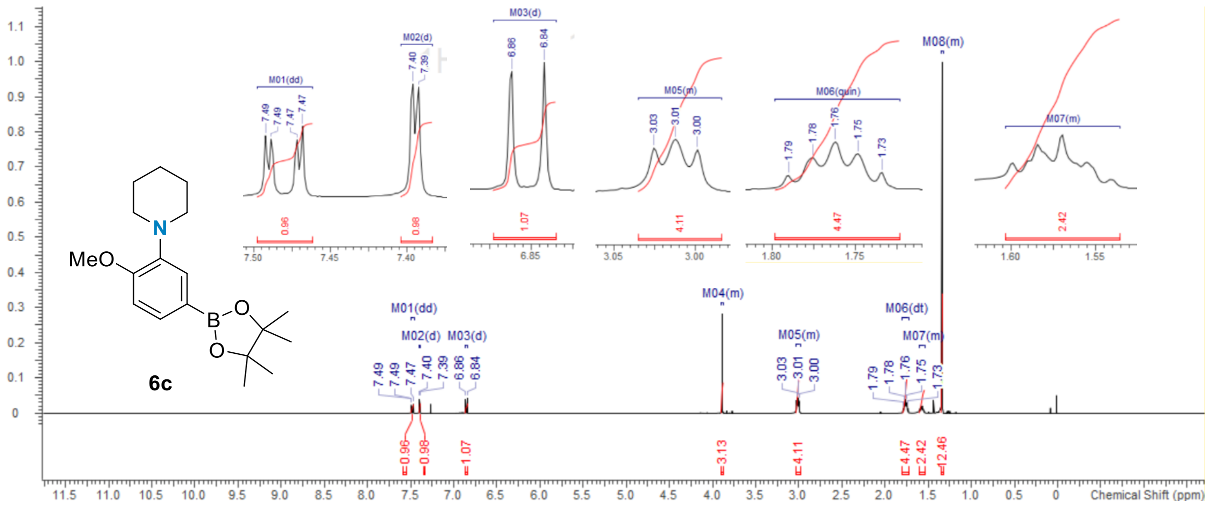

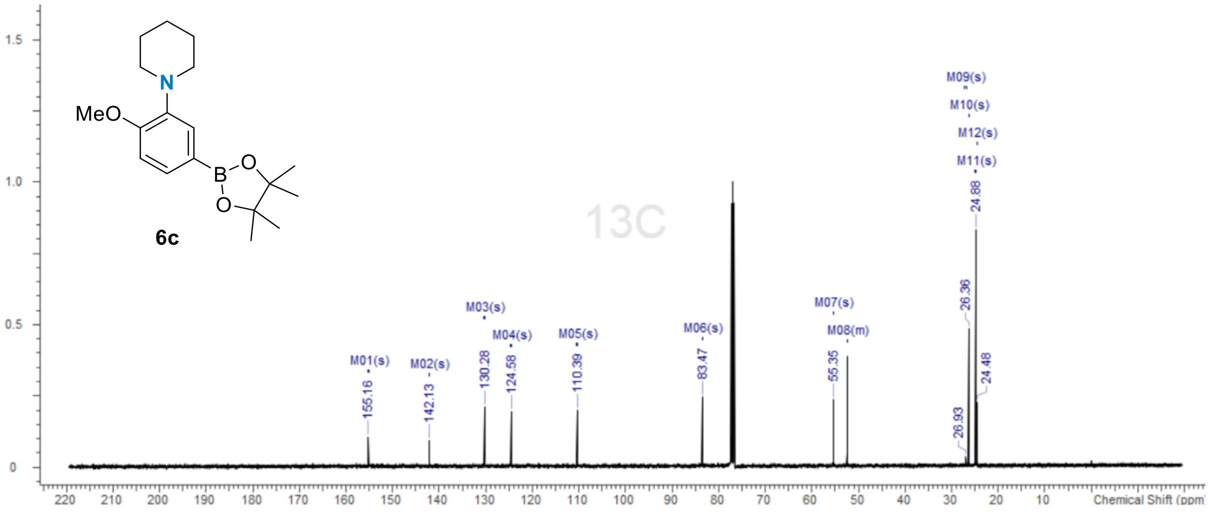

1-(Naphthalen-1-yl)piperidine (**6d**)

Solvent: $CDCl_3$, ^{1}H NMR (400 MHz), ^{13}C NMR (101 MHz).

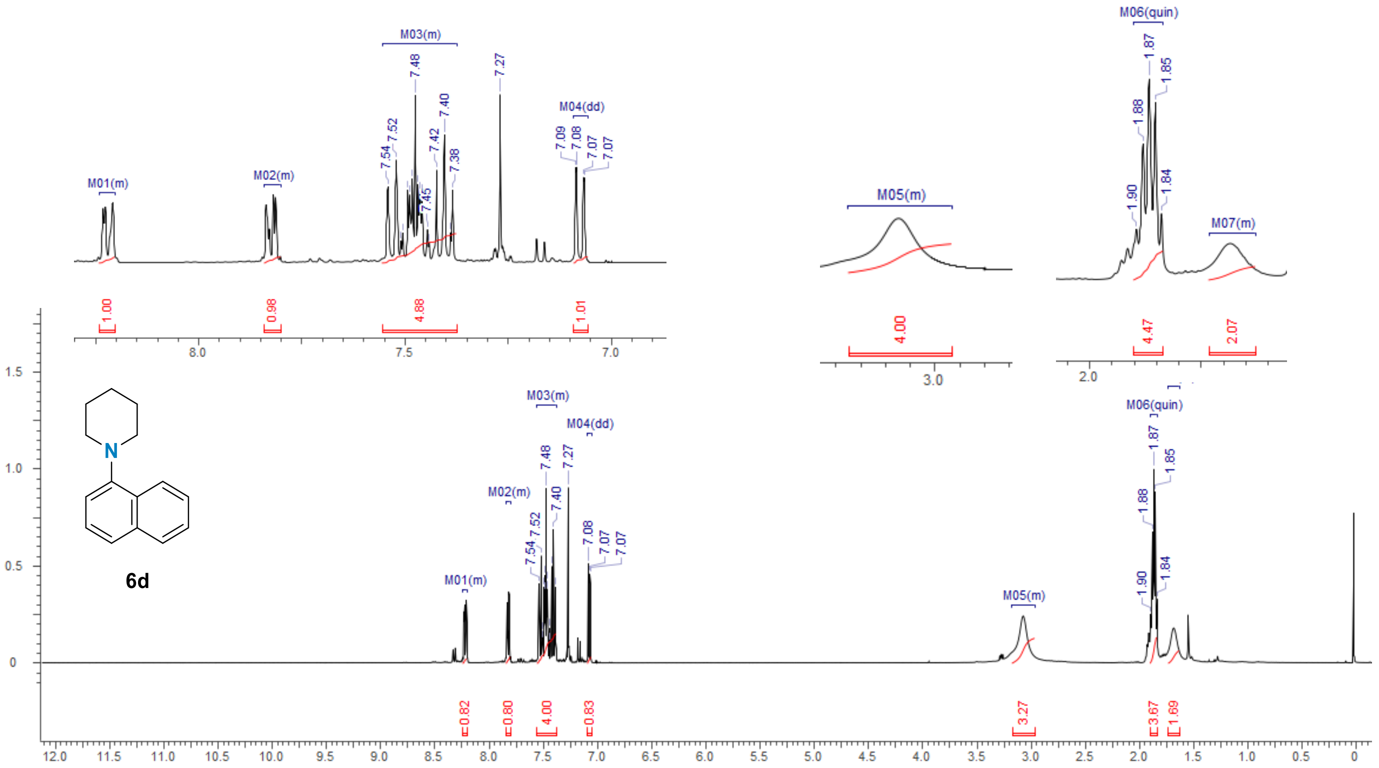

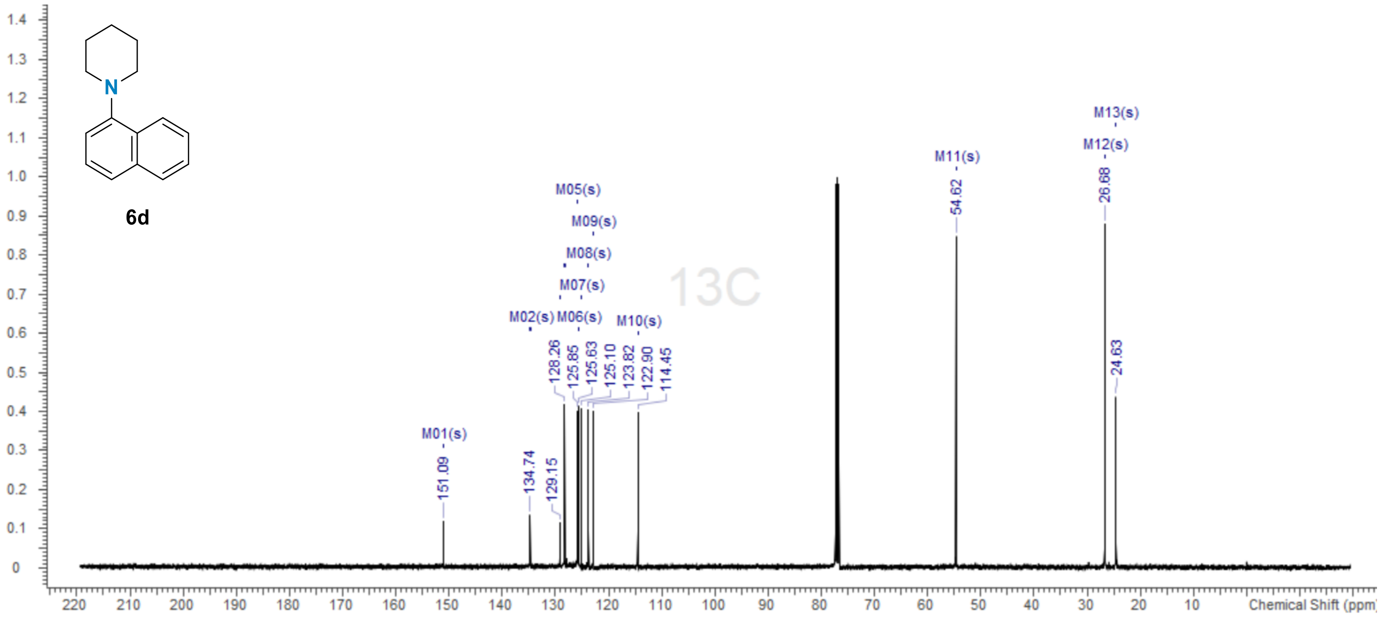

1-(Benzo[*b*]thiophen-2-yl)piperidine (**6e**)

Solvent: $CDCl_3$, ^1H NMR (400 MHz), ^{13}C NMR (101 MHz).

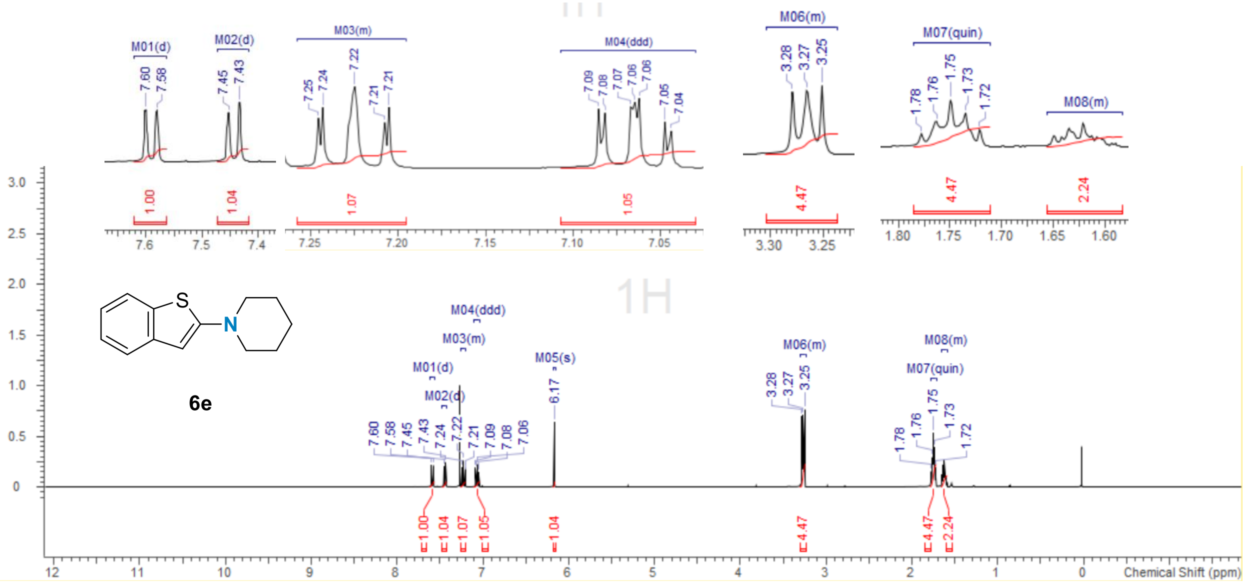

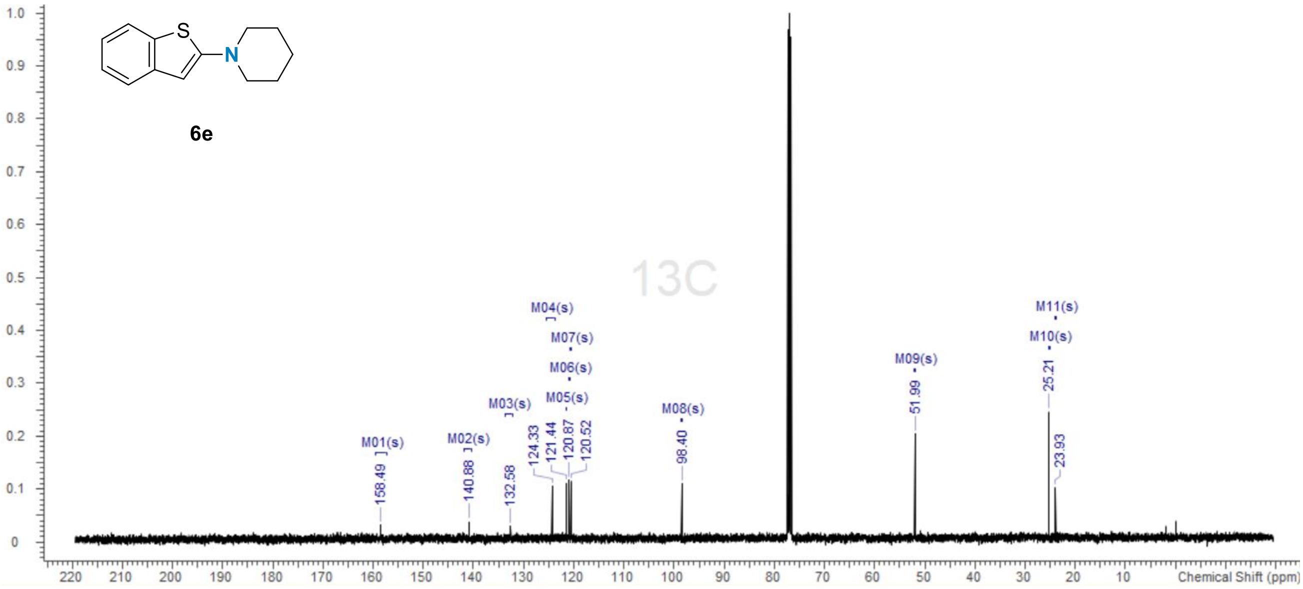

N-(3-Phenylpropyl)hydroxylamine (12a)

Solvent: $CDCl_3$, ^1H NMR (400 MHz), ^{13}C NMR (101 MHz).

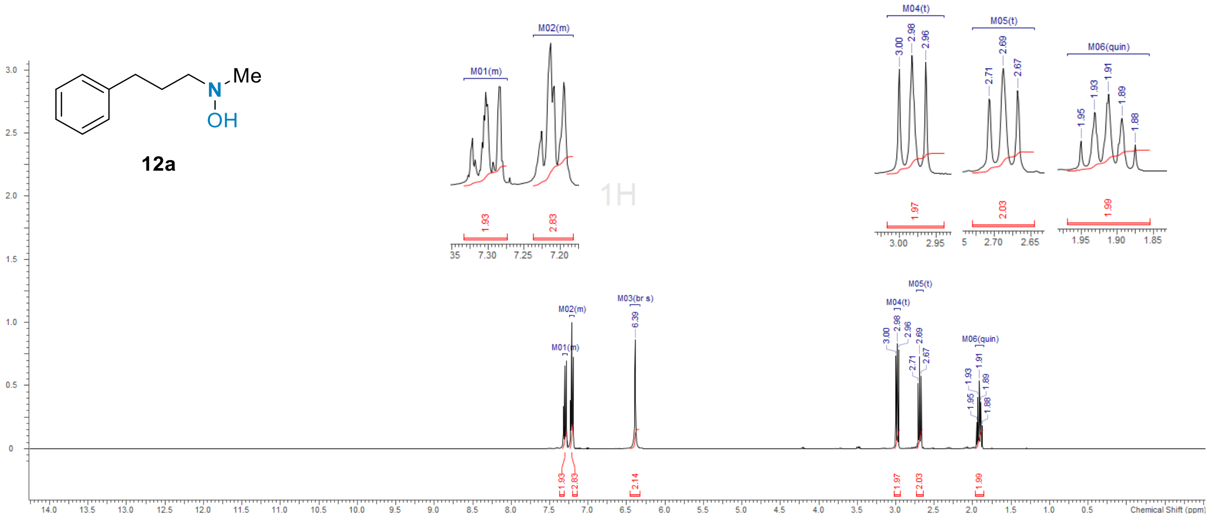

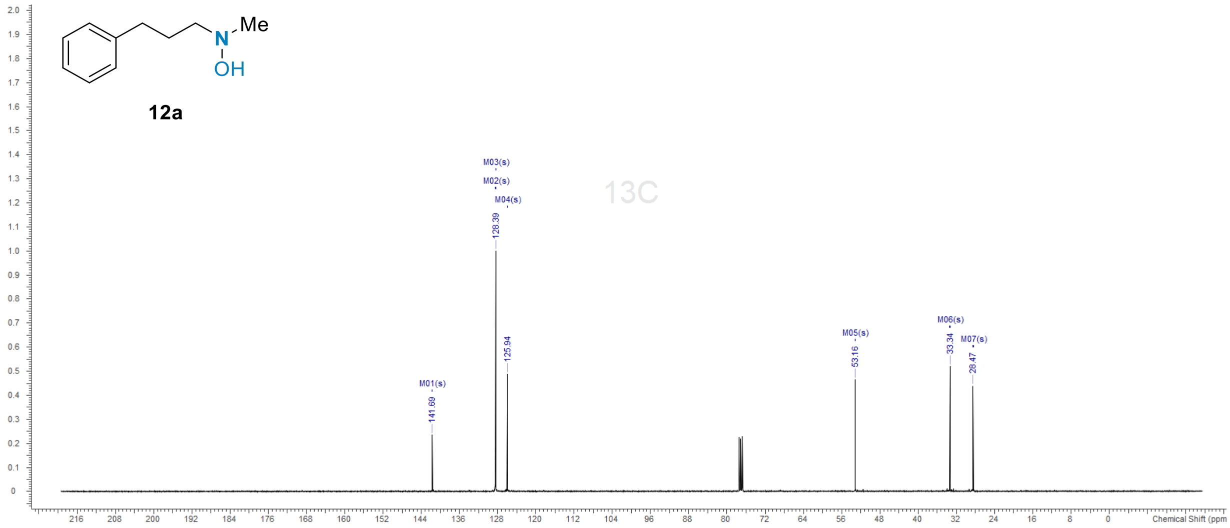

N-Methyl-*N*-(3-(*p*-tolyl)propyl)hydroxylamine (**12b**)

Solvent: $CDCl_3$, ^1H NMR (400 MHz), ^{13}C NMR (101 MHz).

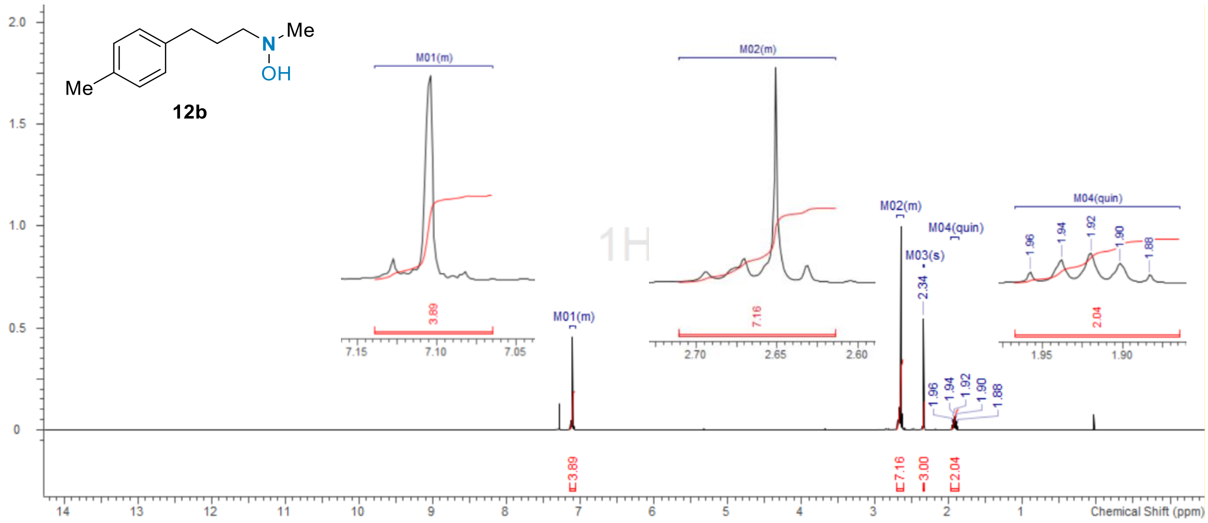

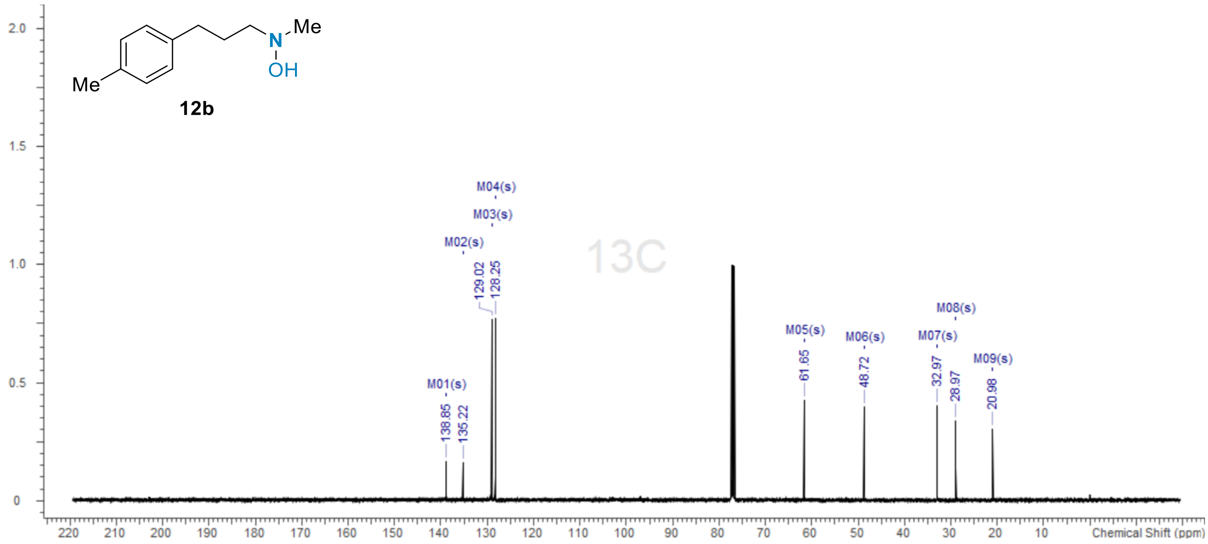

N-(3-(4-(*tert*-Butyl)phenyl)propyl)-*N*-methylhydroxylamine (**12c**)

Solvent: $CDCl_3$, ^{1}H NMR (400 MHz), ^{13}C NMR (101 MHz).

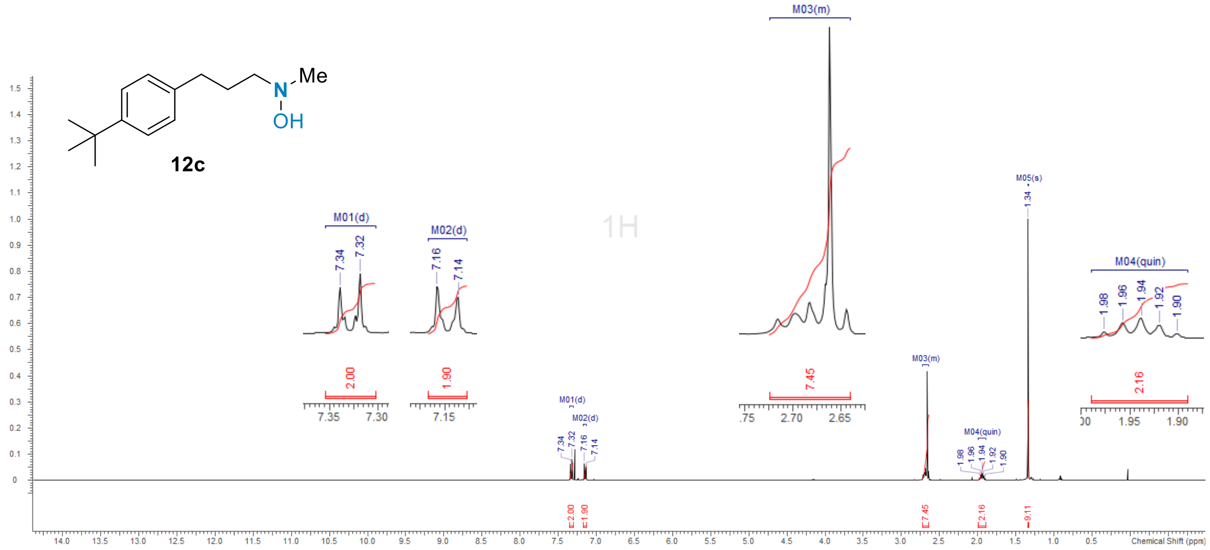

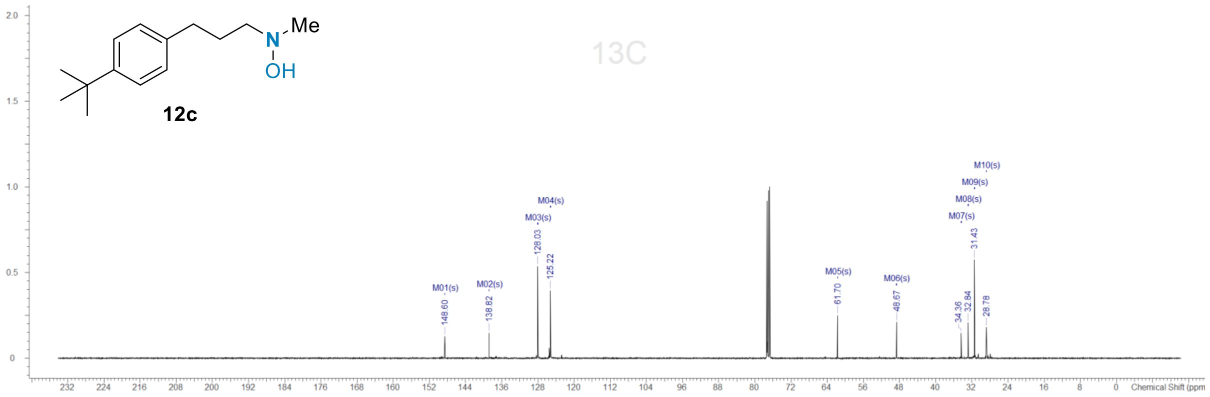

N-(3-([1,1'-Biphenyl]-4-yl)propyl)-*N*-methylhydroxylamine (**12d**)

Solvent: $CDCl_3$, ^1H NMR (400 MHz), ^{13}C NMR (101 MHz).

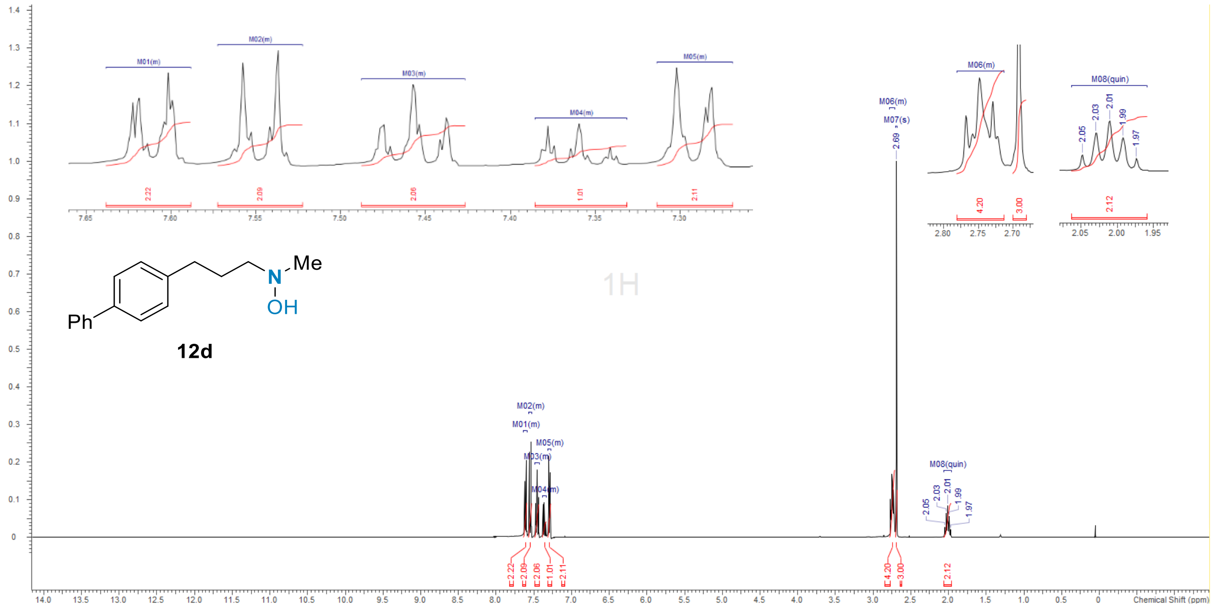

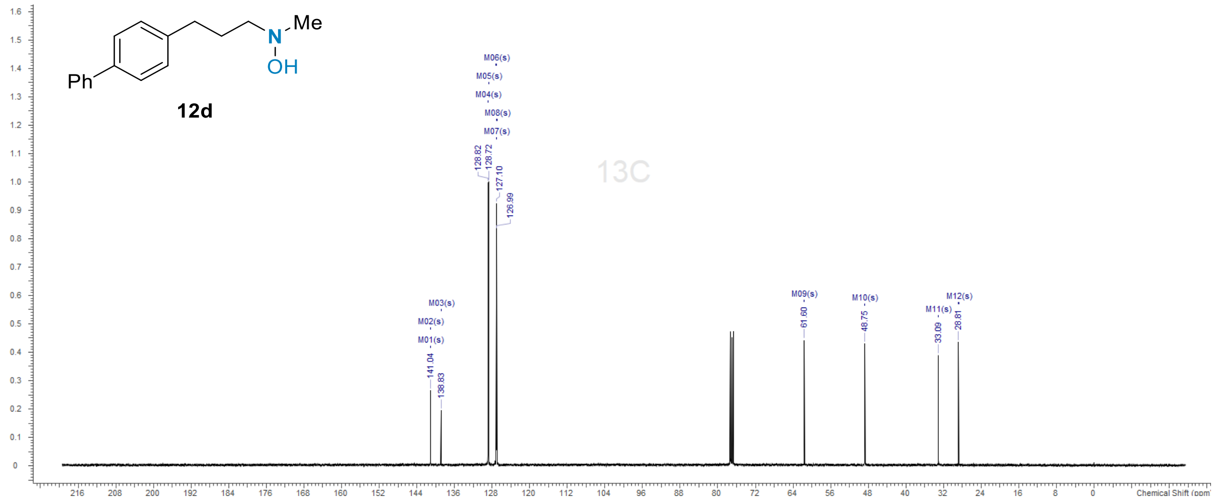

N-(3-(4-Methoxyphenyl)propyl)-*N*-methylhydroxylamine (**12e**)

Solvent: $CDCl_3$, ^1H NMR (400 MHz), ^{13}C NMR (101 MHz).

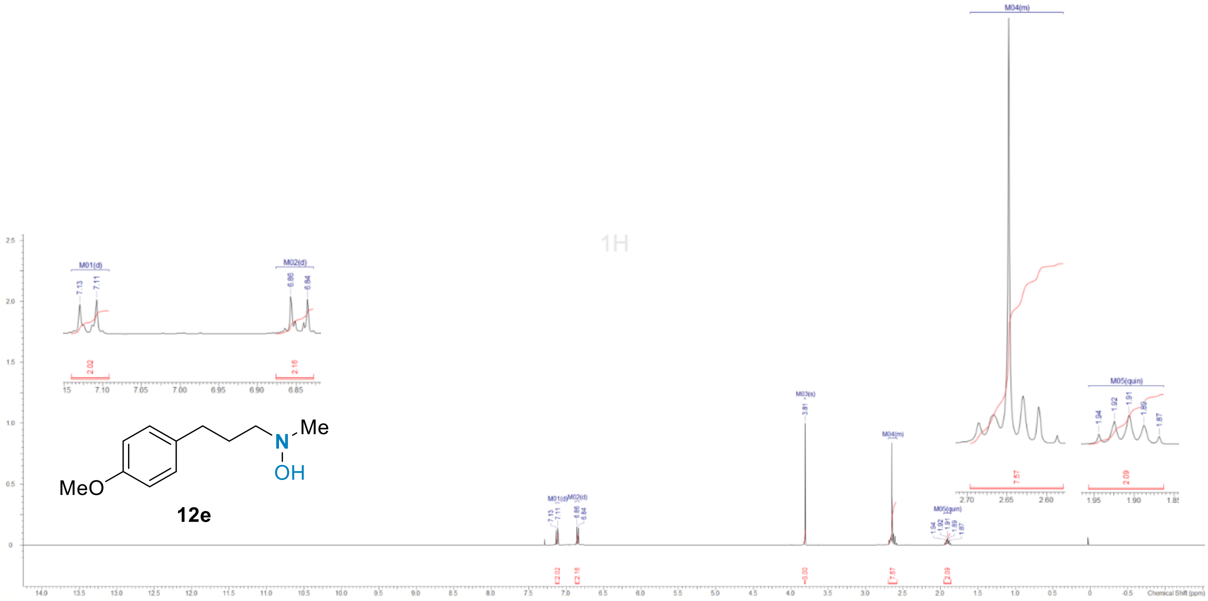

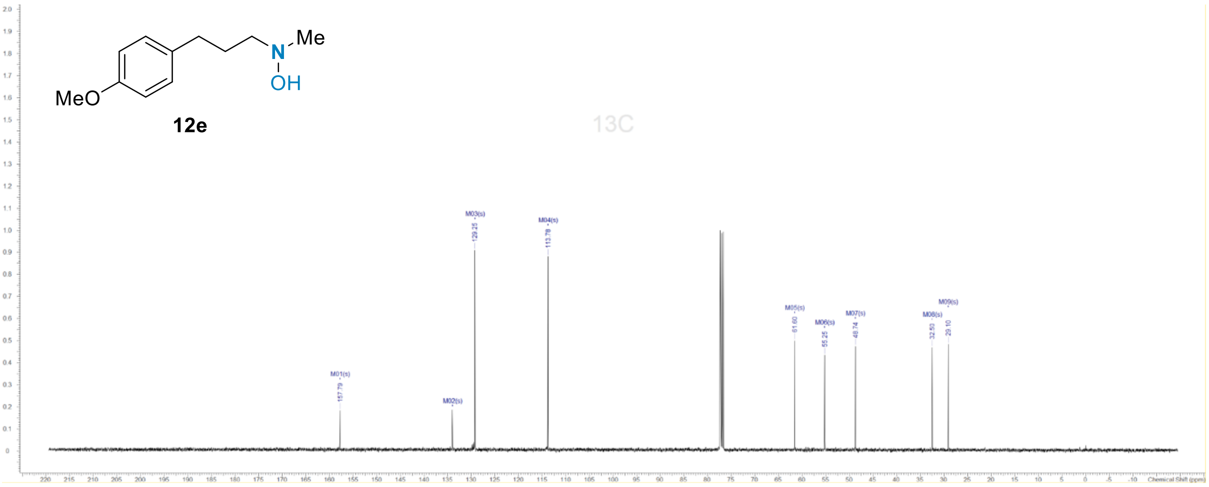

N-(3-(4-Bromophenyl)propyl)-*N*-methylhydroxylamine (**12f**)

Solvent: $CDCl_3$, ^{1}H NMR (400 MHz), ^{13}C NMR (101 MHz).

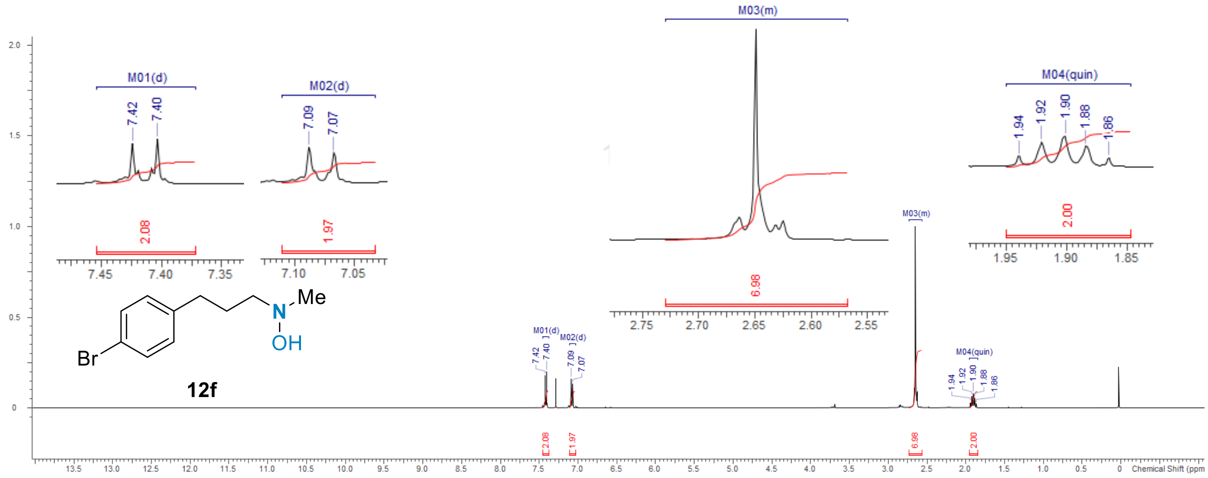

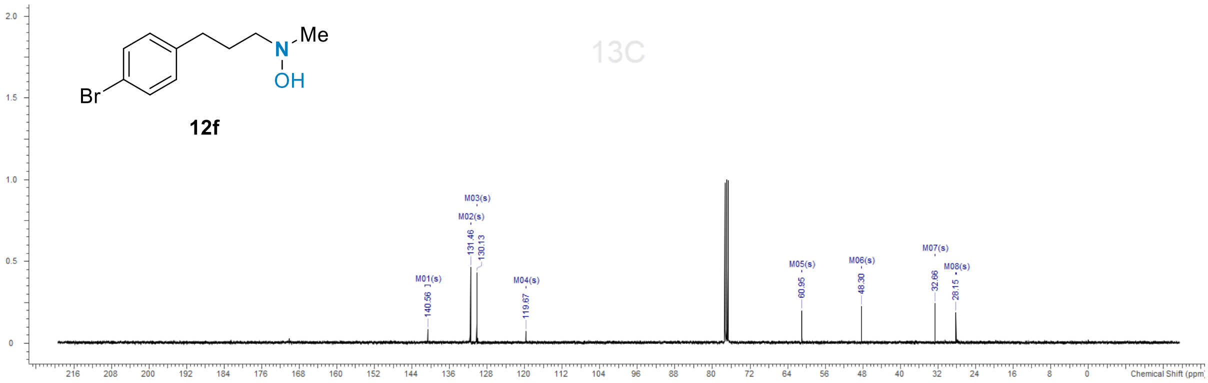

N-(3-(4-Chlorophenyl)propyl)-*N*-methylhydroxylamine (**12g**)

Solvent: $CDCl_3$, ^1H NMR (400 MHz), ^{13}C NMR (101 MHz).

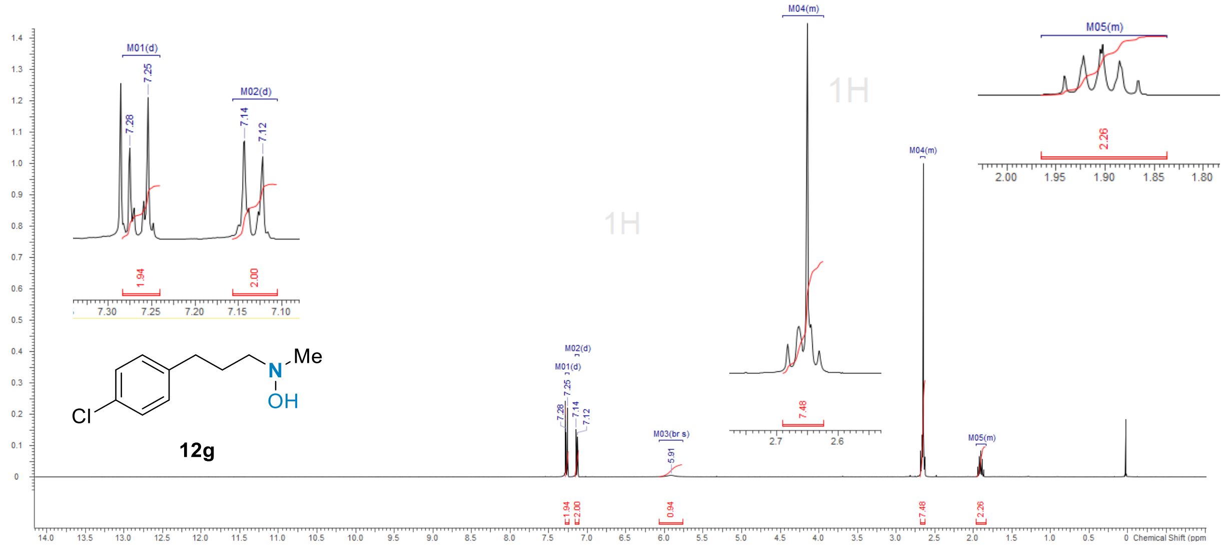

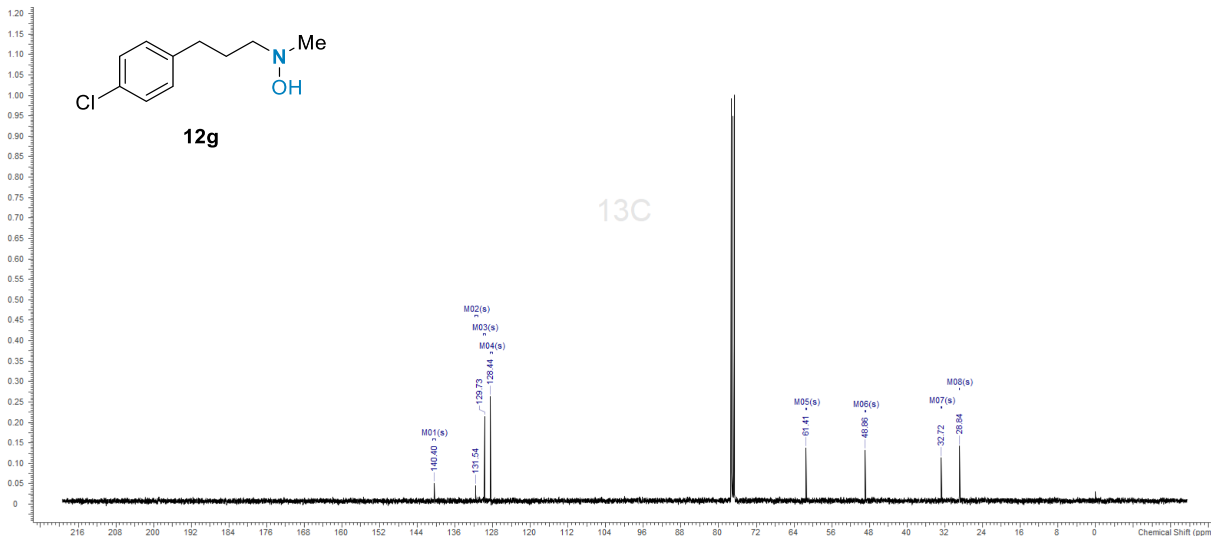

N-(3-(3-Methoxyphenyl)propyl)-*N*-methylhydroxylamine (**12h**)

Solvent: $CDCl_3$, ^1H NMR (400 MHz), ^{13}C NMR (101 MHz).

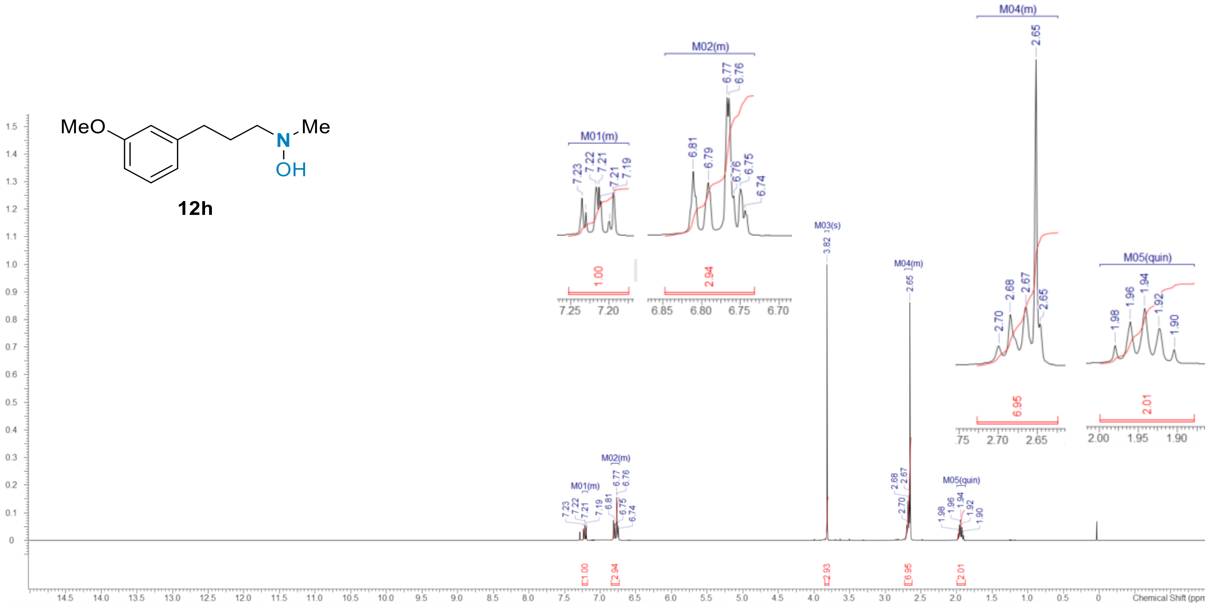

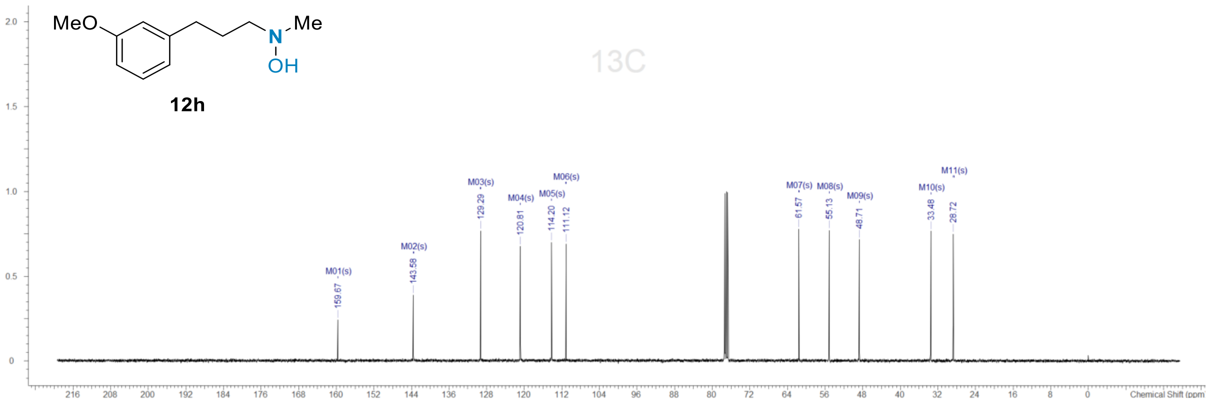

N-(3-(3-Bromophenyl)propyl)-*N*-methylhydroxylamine (**12i**)

Solvent: $CDCl_3$, ^1H NMR (400 MHz), ^{13}C NMR (101 MHz).

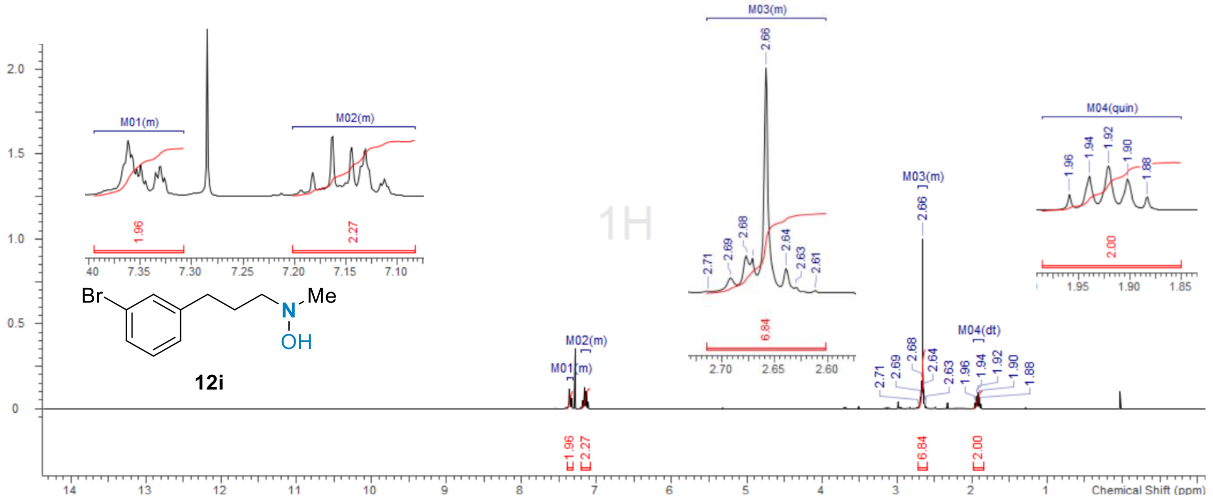

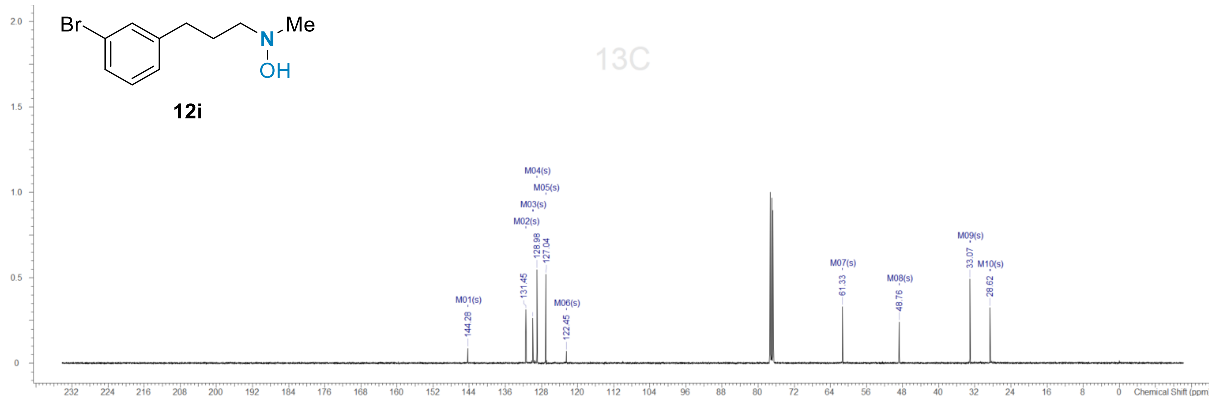

N-Methyl-*N*-(3-(*o*-tolyl)propyl)hydroxylamine (**12j**)

Solvent: $CDCl_3$, ^1H NMR (400 MHz), ^{13}C NMR (101 MHz).

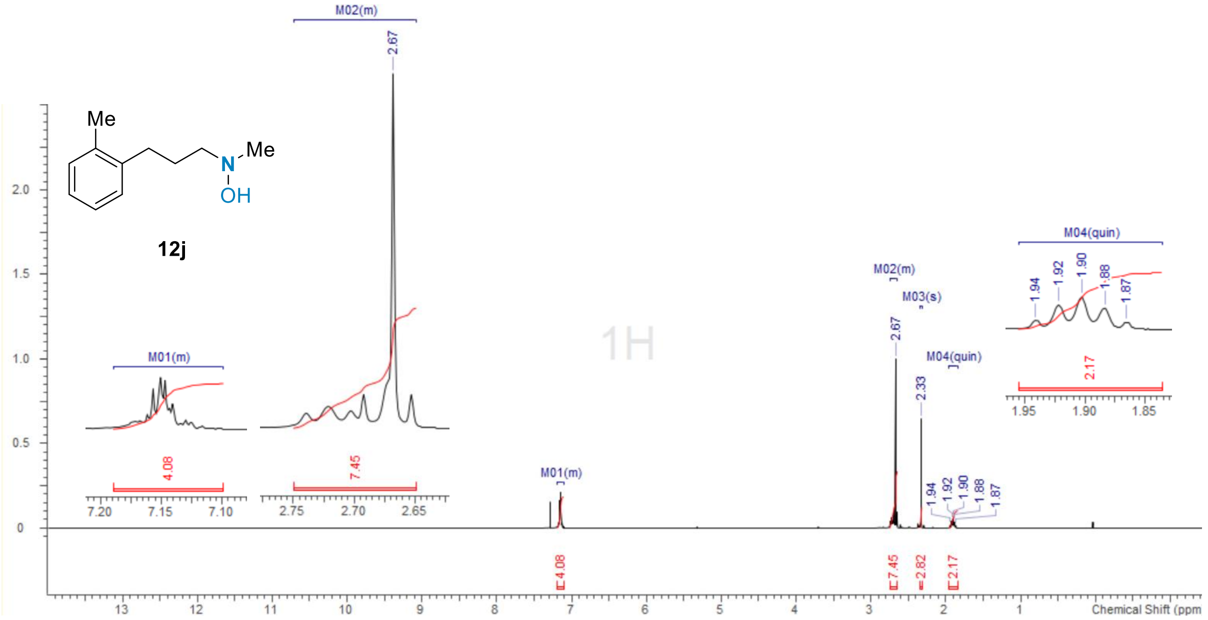

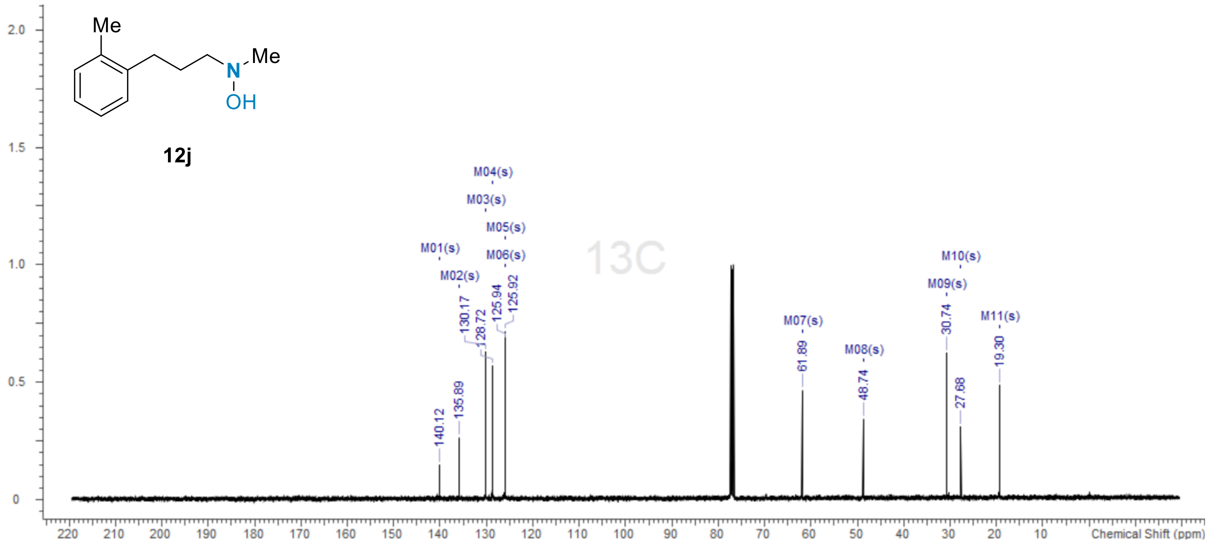

N-(3-(2-Fluorophenyl)propyl)-*N*-methylhydroxylamine (**12k**)

Solvent: $CDCl_3$, ^{1}H NMR (400 MHz), ^{19}F NMR (376 MHz), ^{13}C NMR (101 MHz).

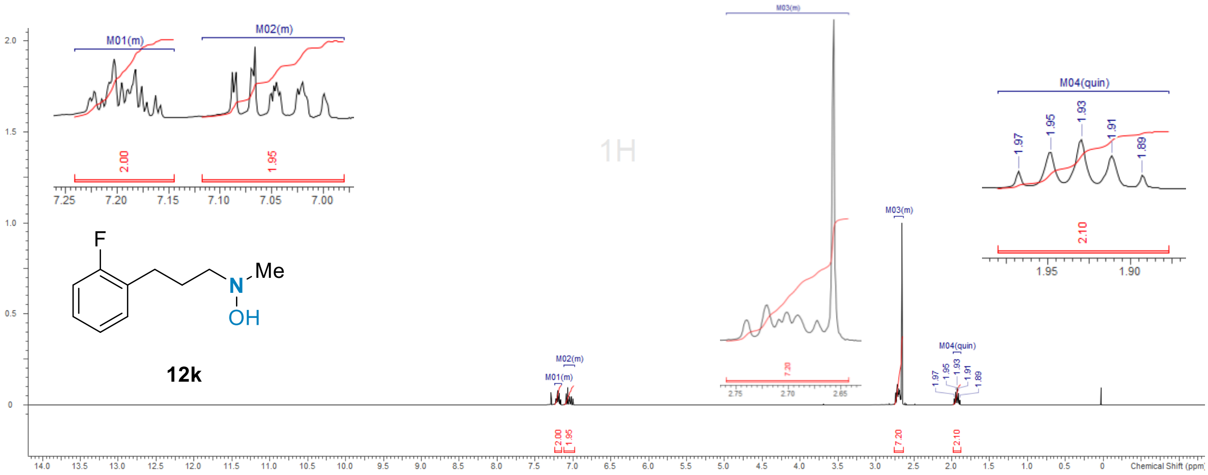

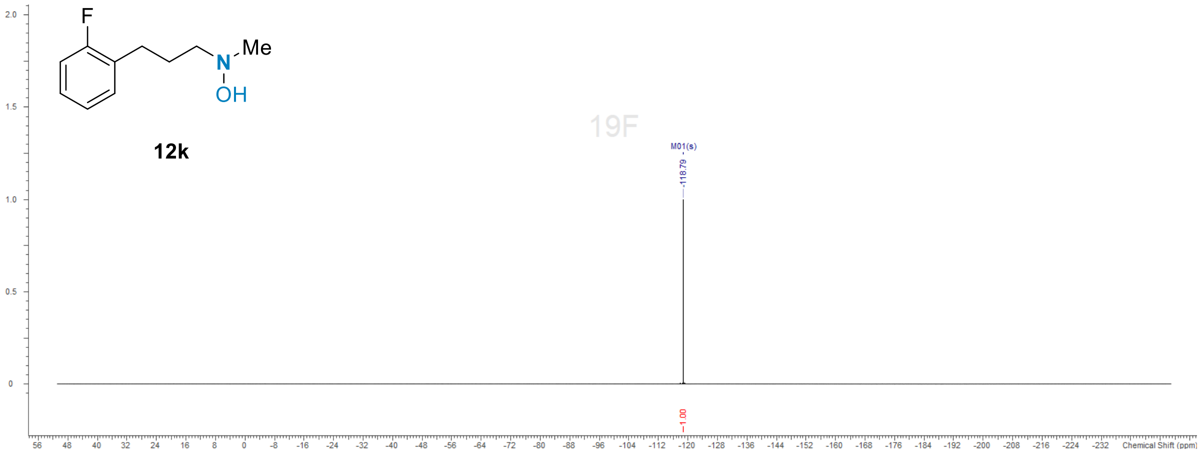

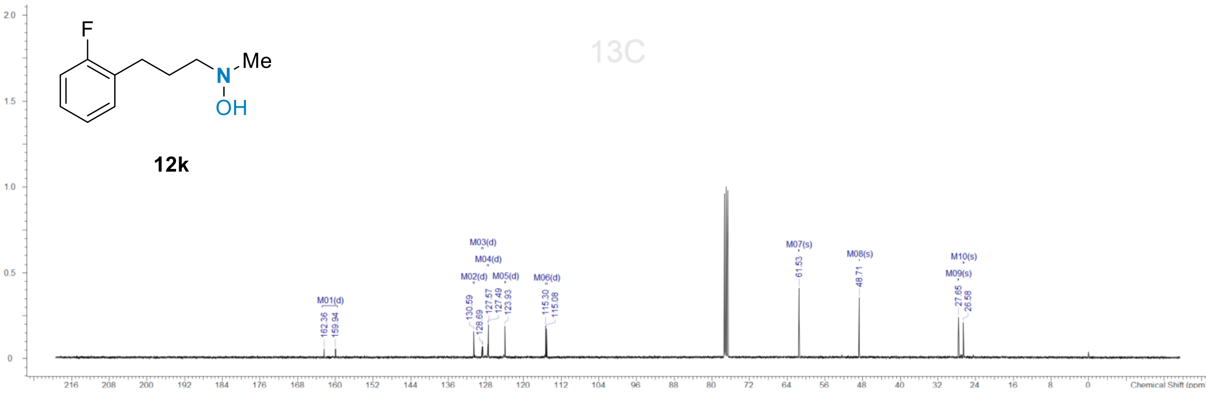

N-(3-(2-Bromophenyl)propyl)-*N*-methylhydroxylamine (**12l**)

Solvent: $CDCl_3$, ^1H NMR (400 MHz), ^{13}C NMR (101 MHz).

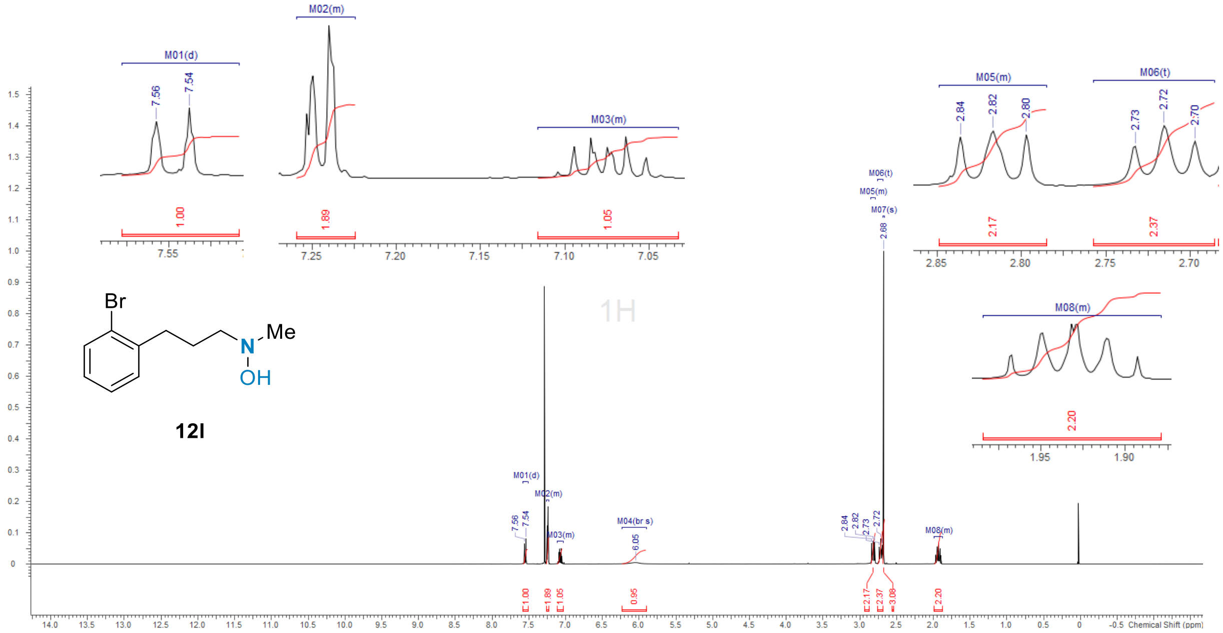

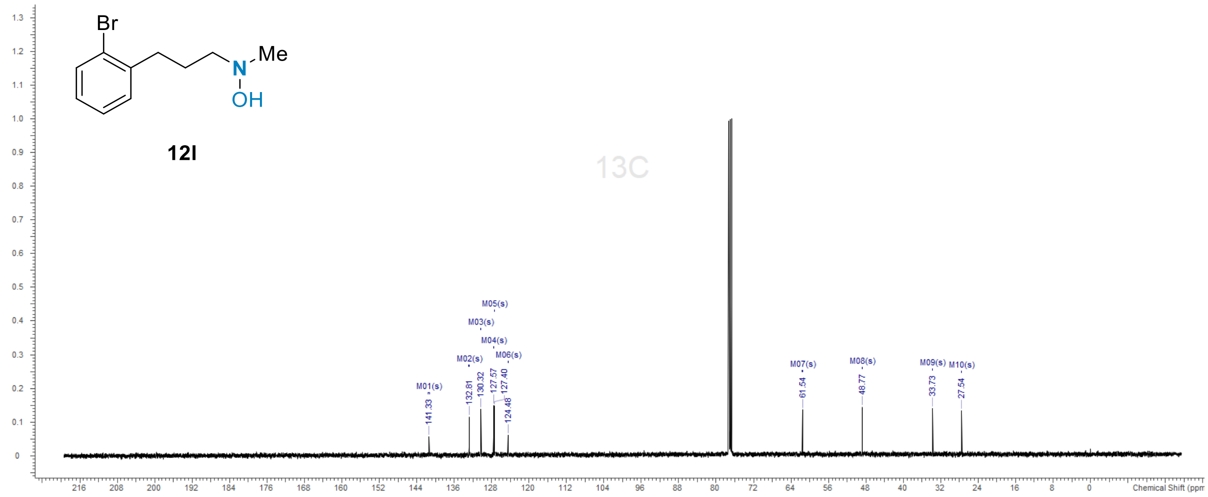

N-Methyl-*N*-(2-phenoxyethyl)hydroxylamine (**12o**)

Solvent: $CDCl_3$, ^1H NMR (400 MHz), ^{13}C NMR (101 MHz).

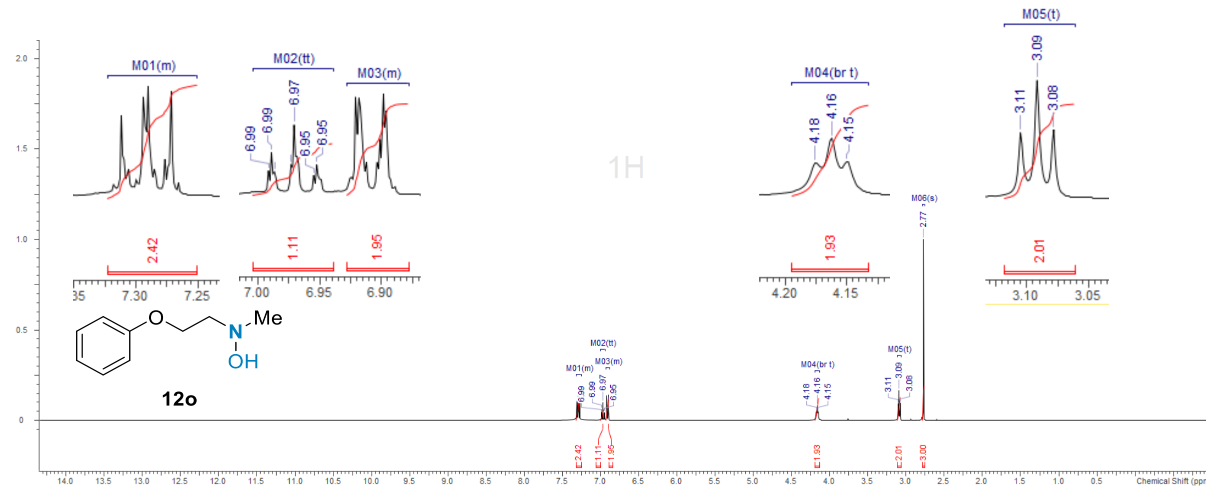

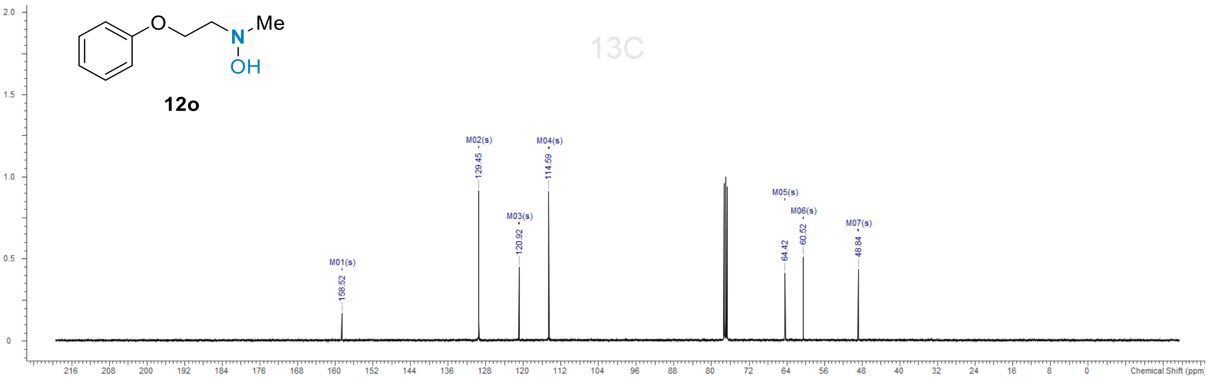

Ethyl 3-phenylbutanoate (**13m**)

Solvent: $CDCl_3$, ^1H NMR (400 MHz), ^{13}C NMR (101 MHz).

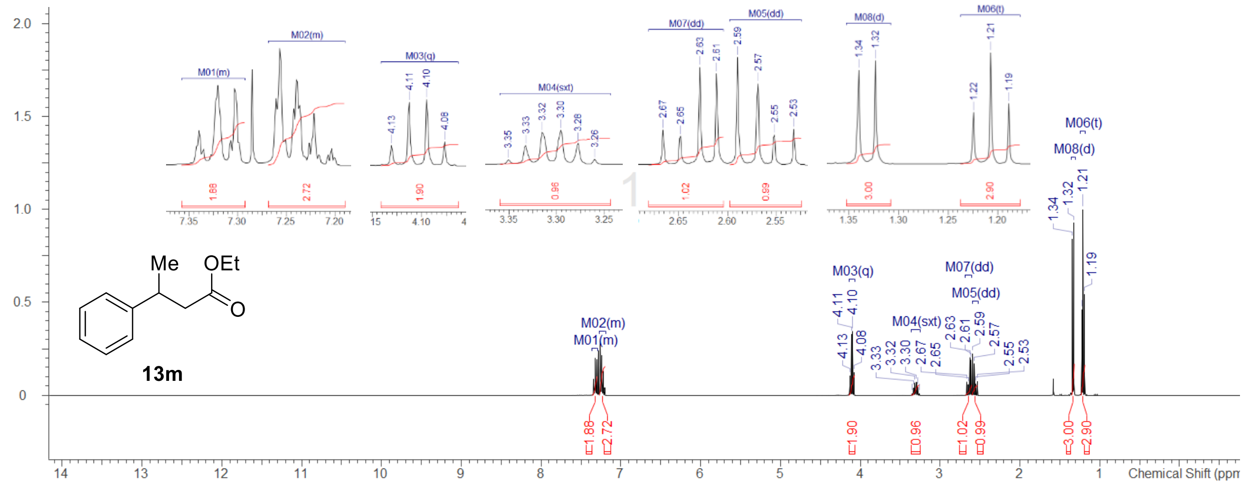

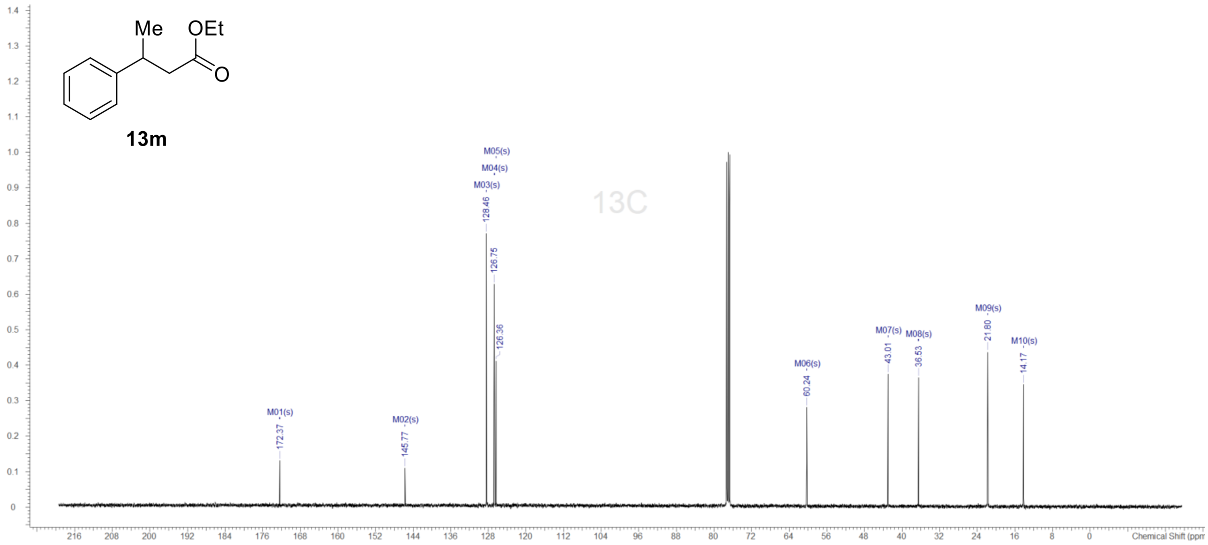

Ethyl 3,3-diphenylpropanoate (**13n**)

Solvent: $CDCl_3$, ^{1}H NMR (400 MHz), ^{13}C NMR (101 MHz).

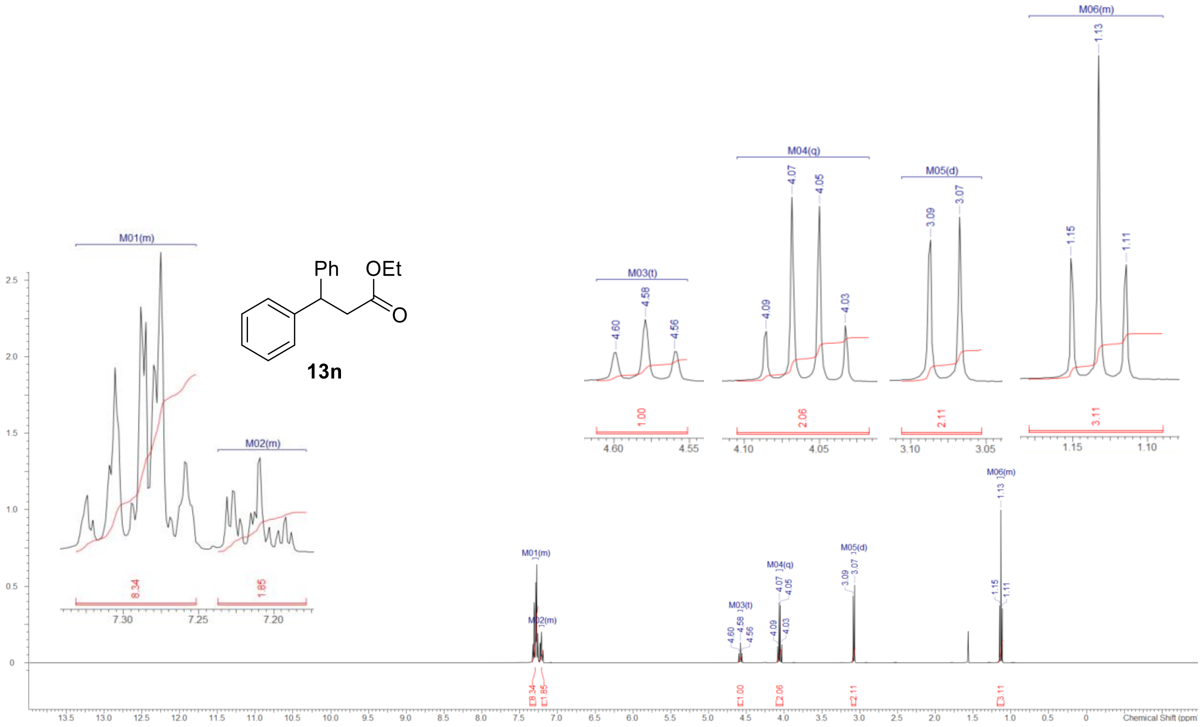

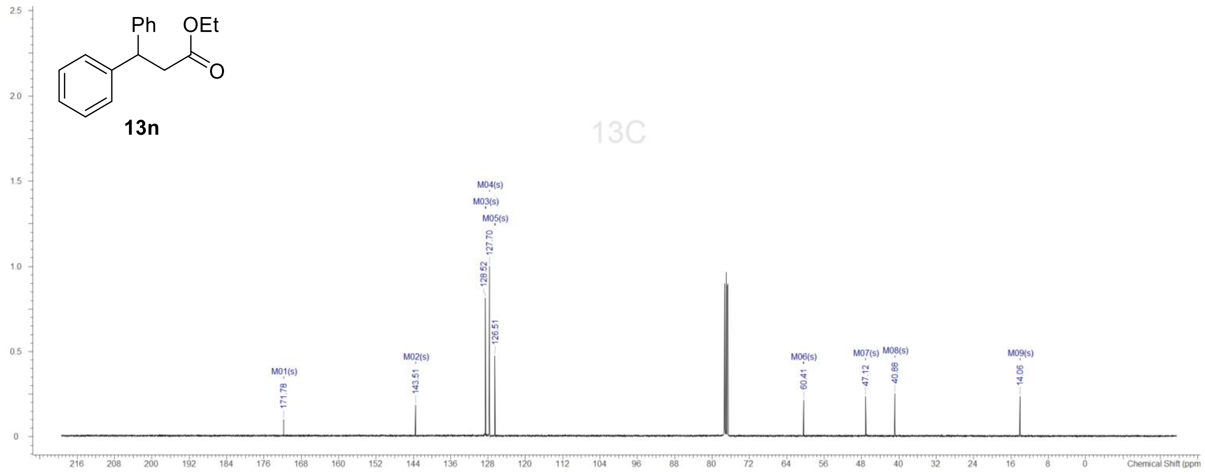

3-Phenylbutan-1-ol (**14m**)

Solvent: $CDCl_3$, ^1H NMR (400 MHz), ^{13}C NMR (101 MHz).

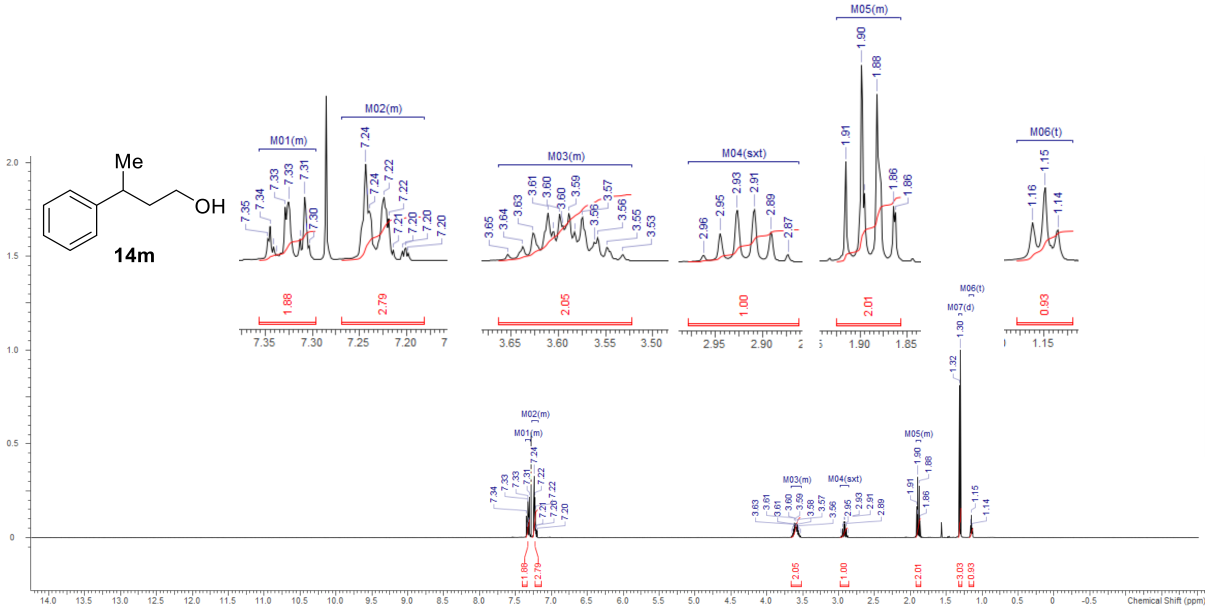

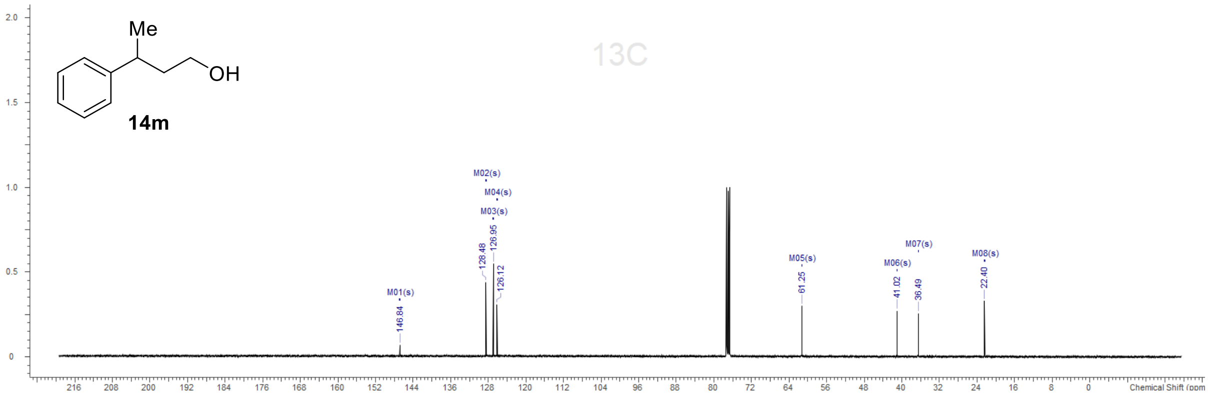

3,3-Diphenylpropan-1-ol (**21n**)

Solvent: $CDCl_3$, ^{1}H NMR (400 MHz), ^{13}C NMR (101 MHz).

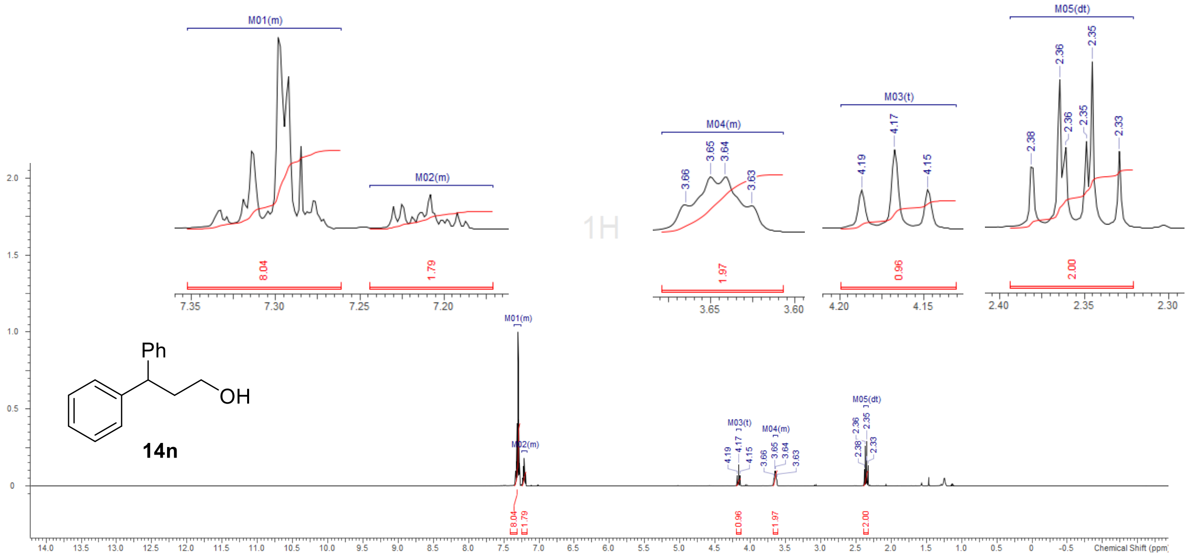

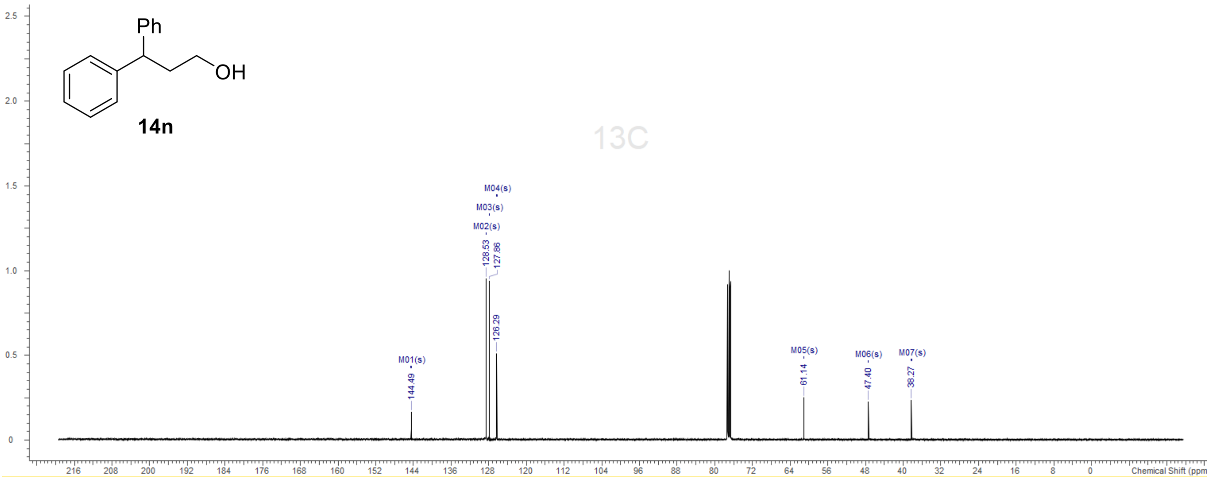

3-Phenylbutyl methanesulfonate (**15m**)

Solvent: $CDCl_3$, ^{1}H NMR (400 MHz), ^{13}C NMR (101 MHz).

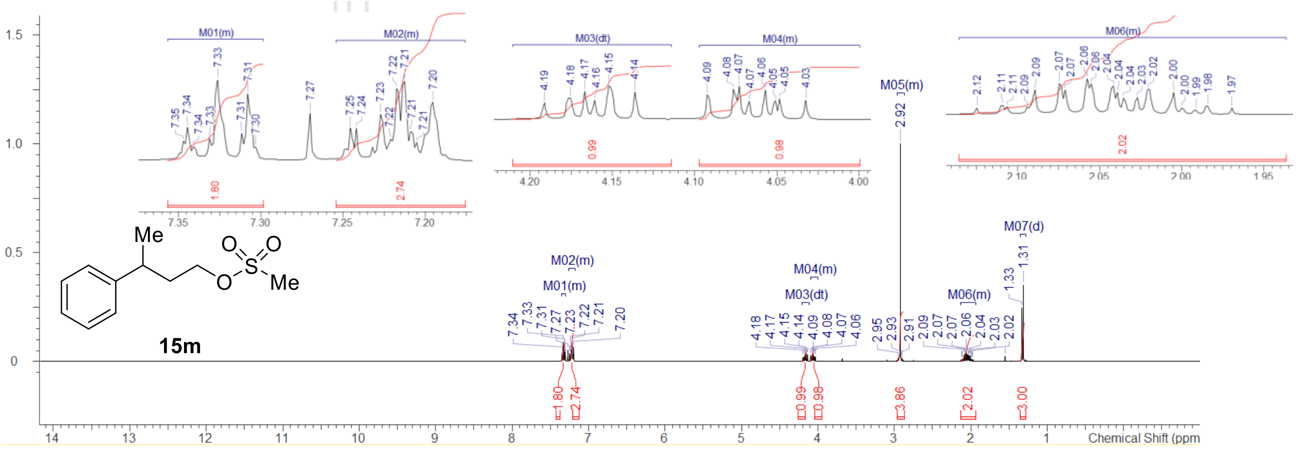

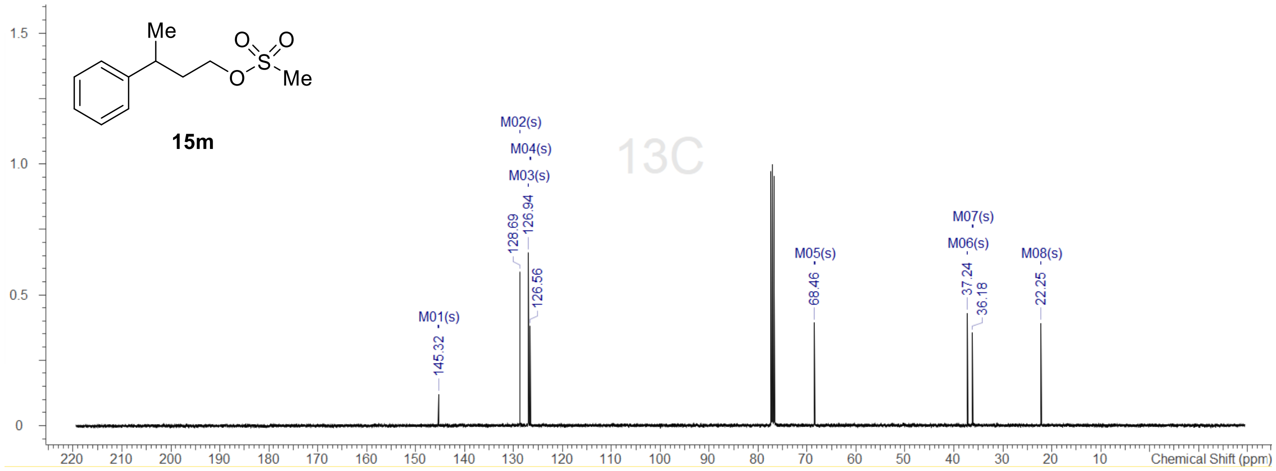

3,3-Diphenylpropyl methanesulfonate (**15n**)

Solvent: $CDCl_3$, ^1H NMR (400 MHz), ^{13}C NMR (101 MHz).

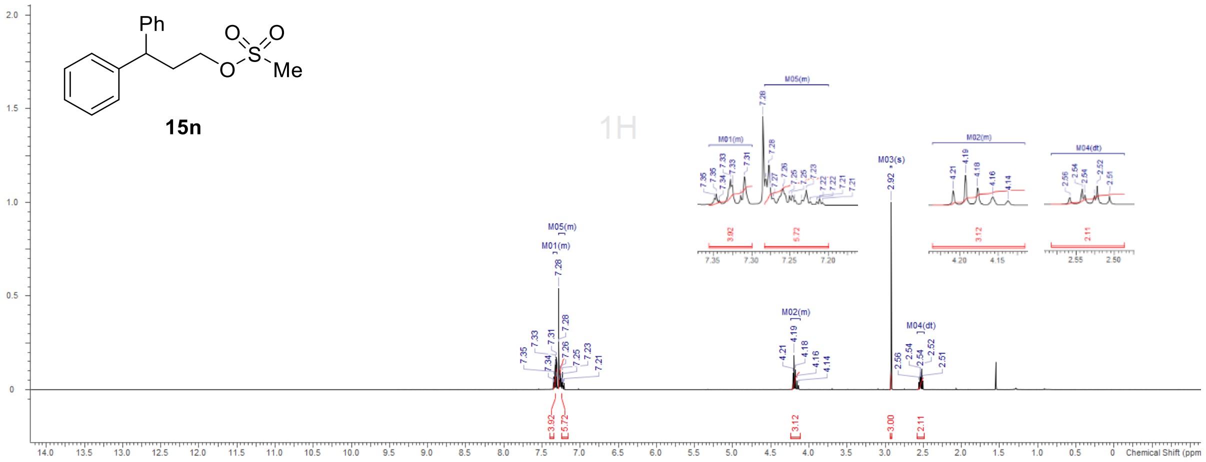

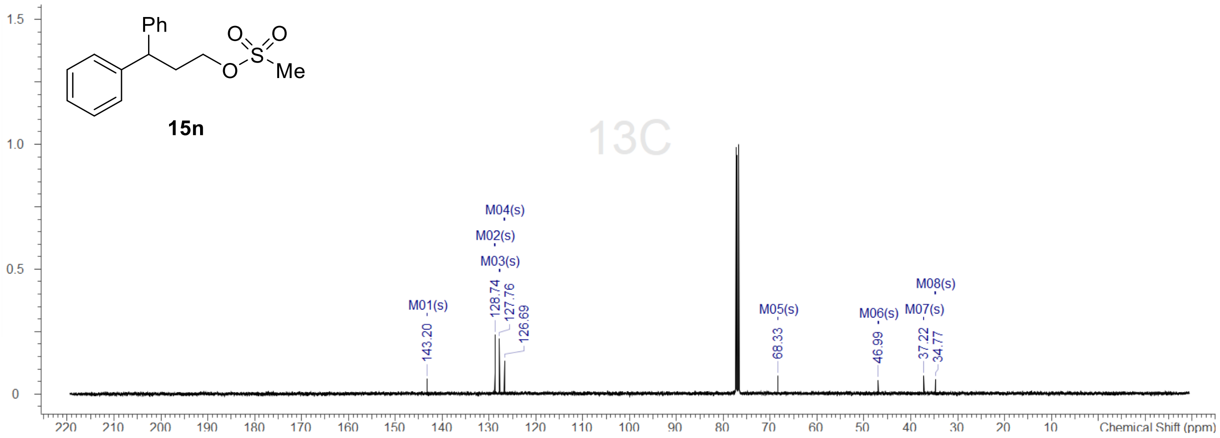

O-(2,4-Dinitrophenyl)-*N*-methyl-*N*-(3-phenylpropyl)hydroxylamine (**7a**)

Solvent: $CDCl_3$, ^{1}H NMR (400 MHz), ^{13}C NMR (101 MHz).

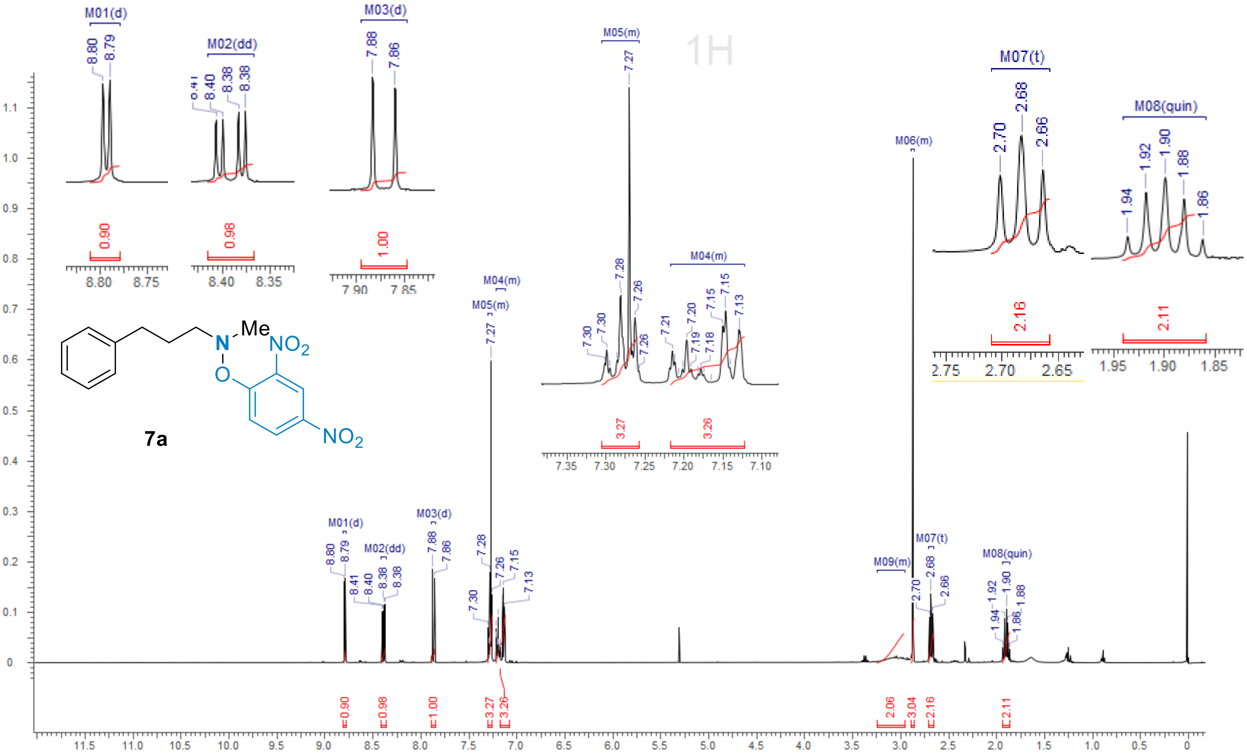

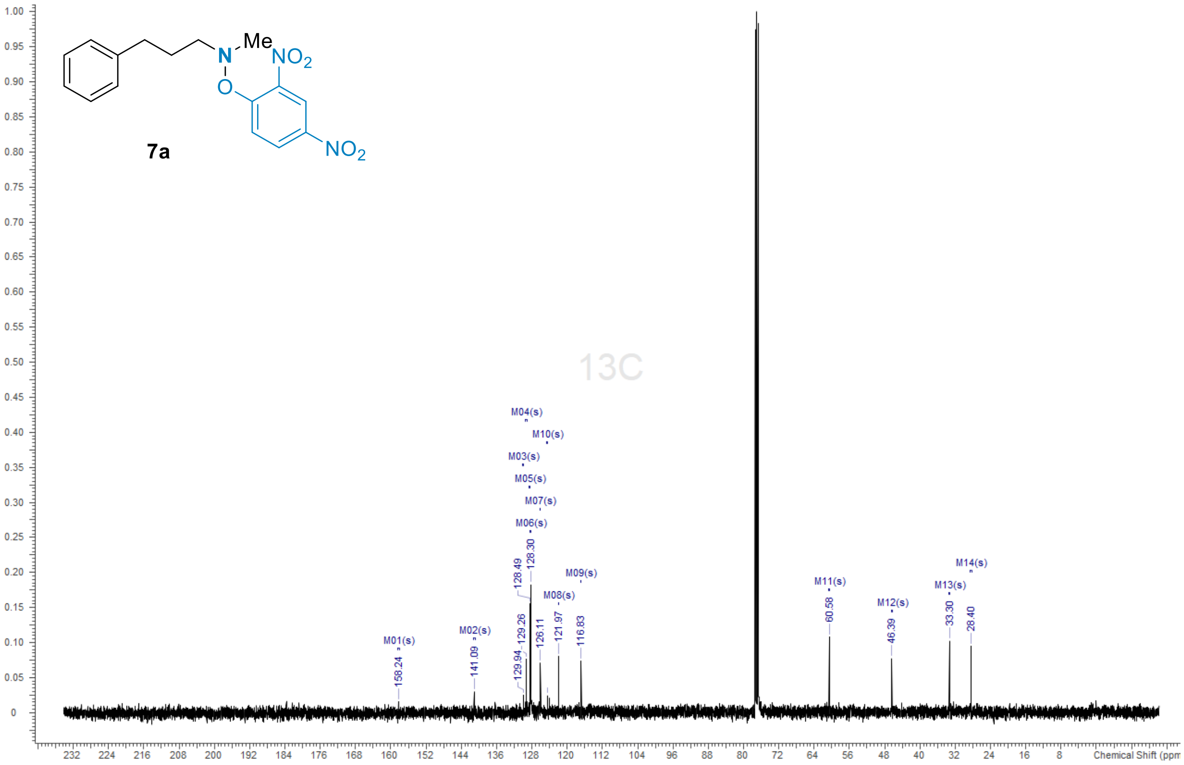

O-(2,4-Dinitrophenyl)-*N*-methyl-*N*-(3-(*p*-tolyl)propyl)hydroxylamine (**7b**)

Solvent: $CDCl_3$, ^1H NMR (400 MHz), ^{13}C NMR (101 MHz).

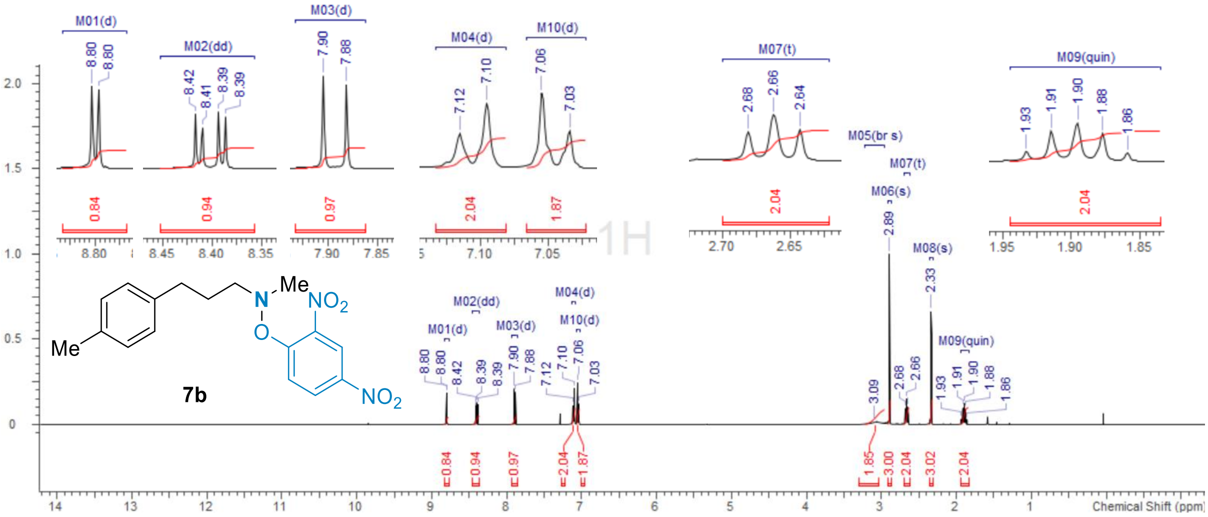

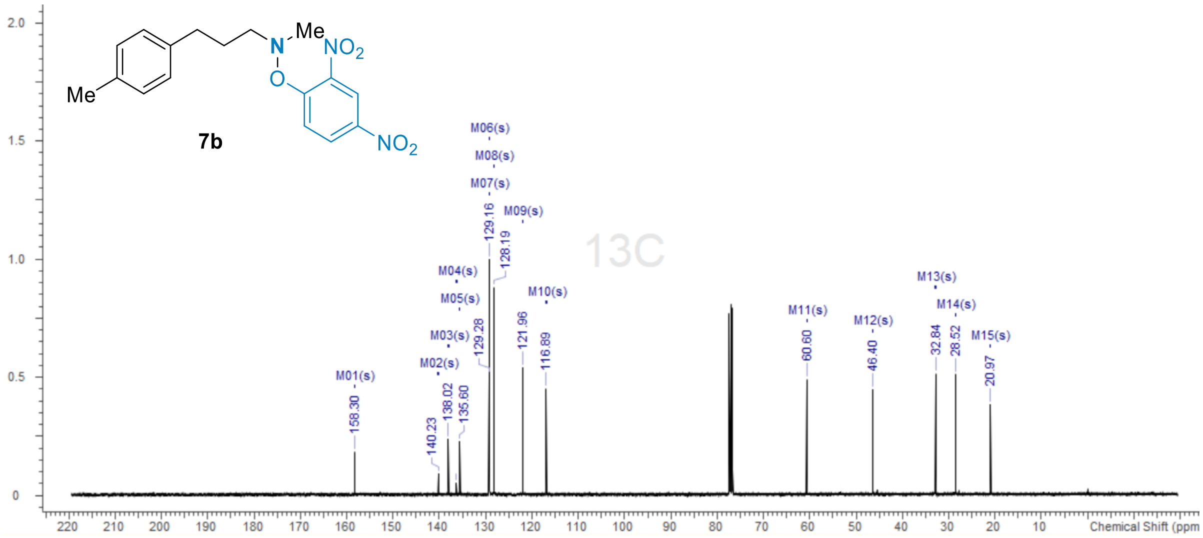

N-(3-(4-(*tert*-Butyl)phenyl)propyl)-*O*-(2,4-dinitrophenyl)-*N*-methylhydroxylamine (**7c**)

Solvent: $CDCl_3$, ^{1}H NMR (400 MHz), ^{13}C NMR (101 MHz).

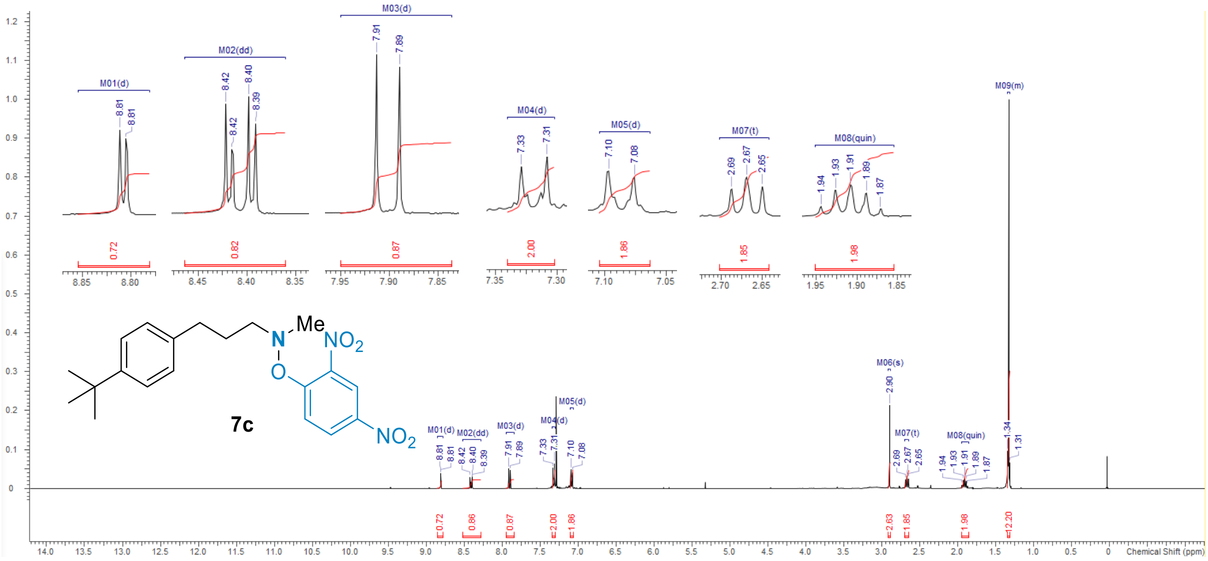

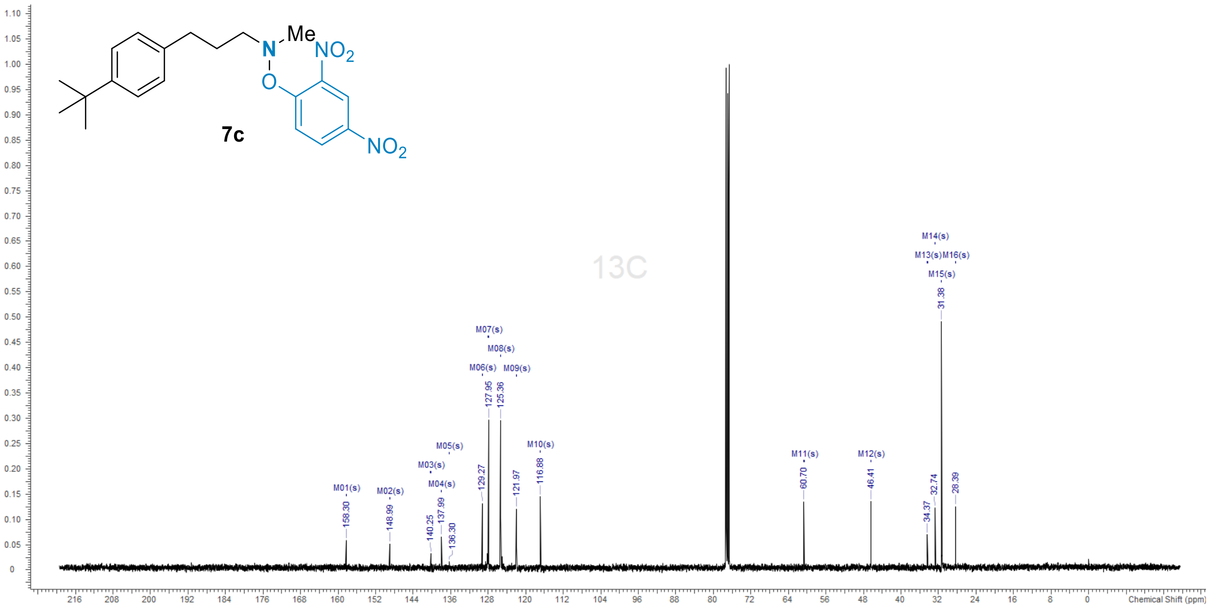

N-(3-([1,1'-Biphenyl]-4-yl)propyl)-*O*-(2,4-dinitrophenyl)-*N*-methylhydroxylamine (**7d**)

Solvent: $CDCl_3$, ^1H NMR (400 MHz), ^{13}C NMR (101 MHz).

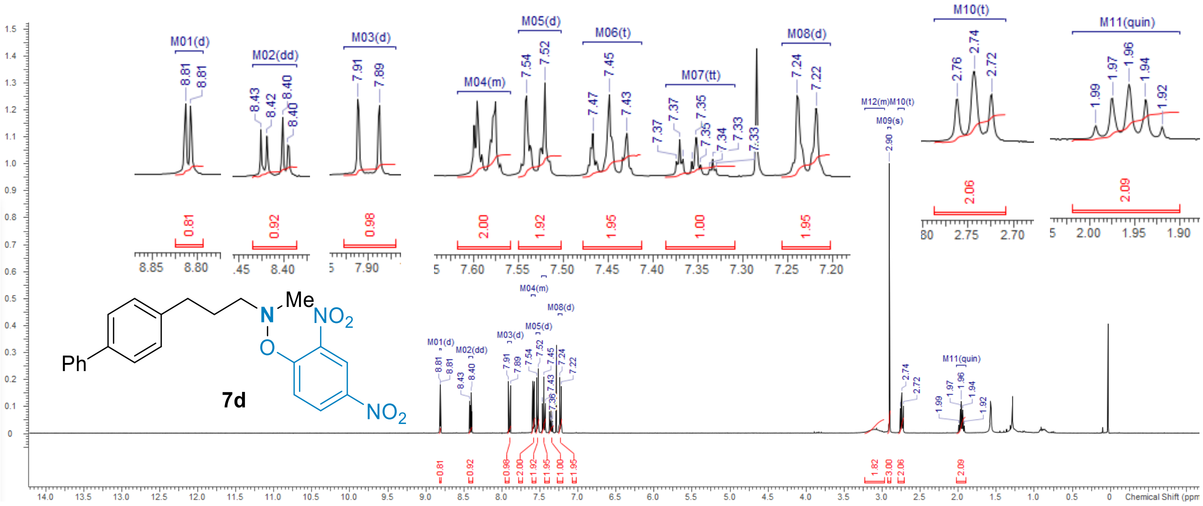

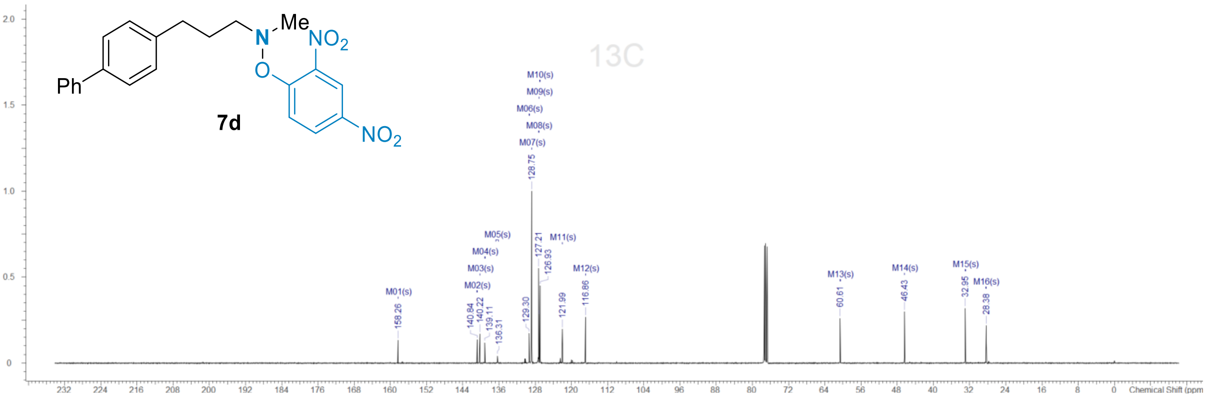

O-(2,4-Dinitrophenyl)-*N*-(3-(4-methoxyphenyl)propyl)-*N*-methylhydroxylamine (**7e**)

Solvent: $CDCl_3$, ^1H NMR (400 MHz), ^{13}C NMR (101 MHz).

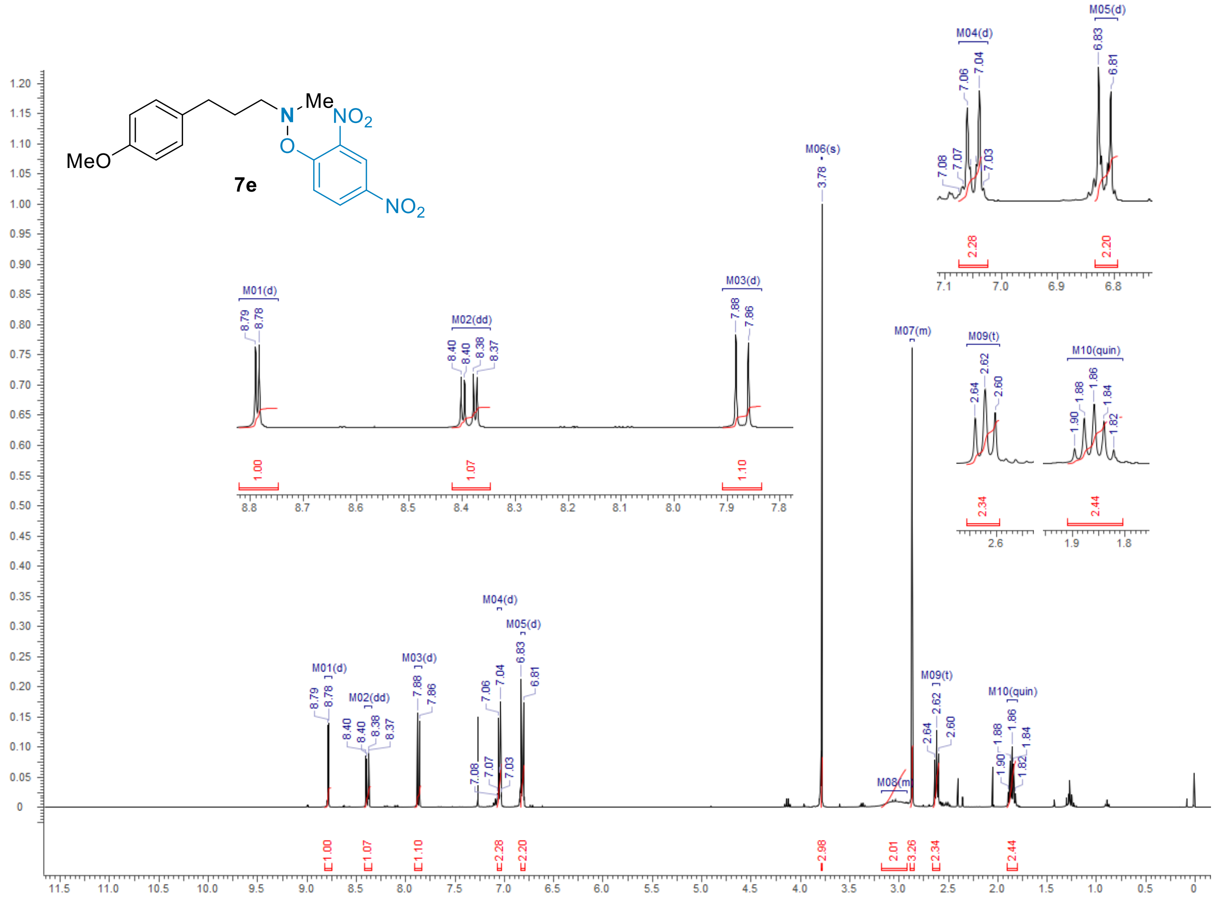

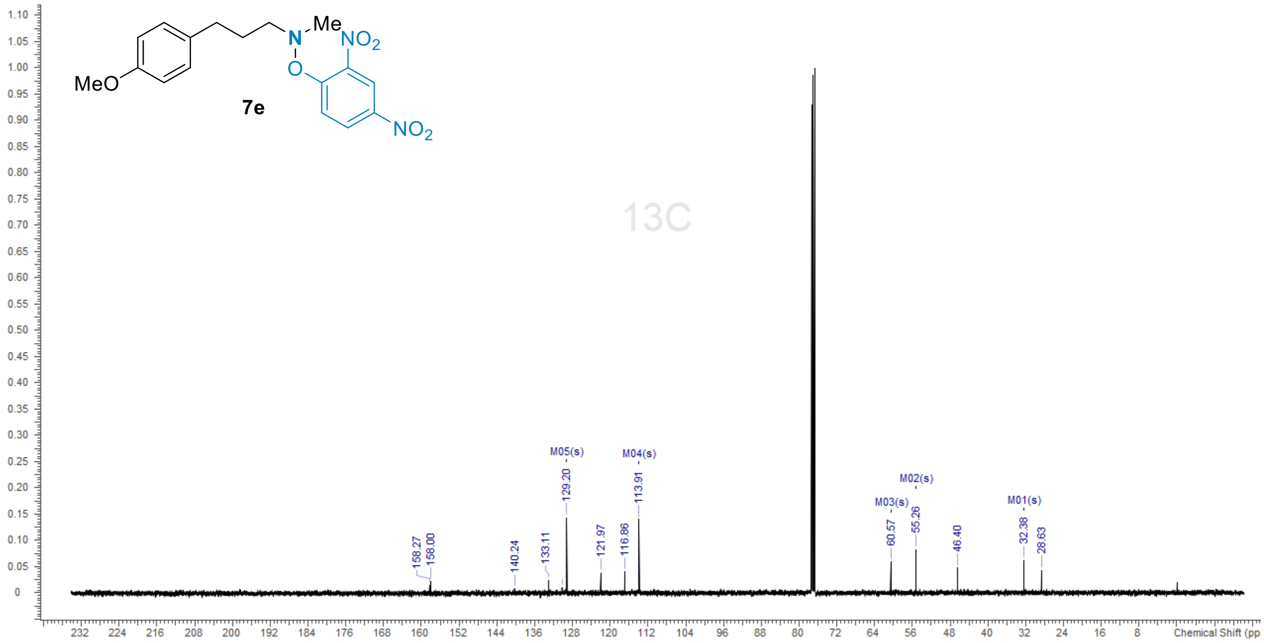

N-(3-(4-Bromophenyl)propyl)-*O*-(2,4-dinitrophenyl)-*N*-methylhydroxylamine (**7f**)

Solvent: $CDCl_3$, ^1H NMR (400 MHz), ^{13}C NMR (101 MHz).

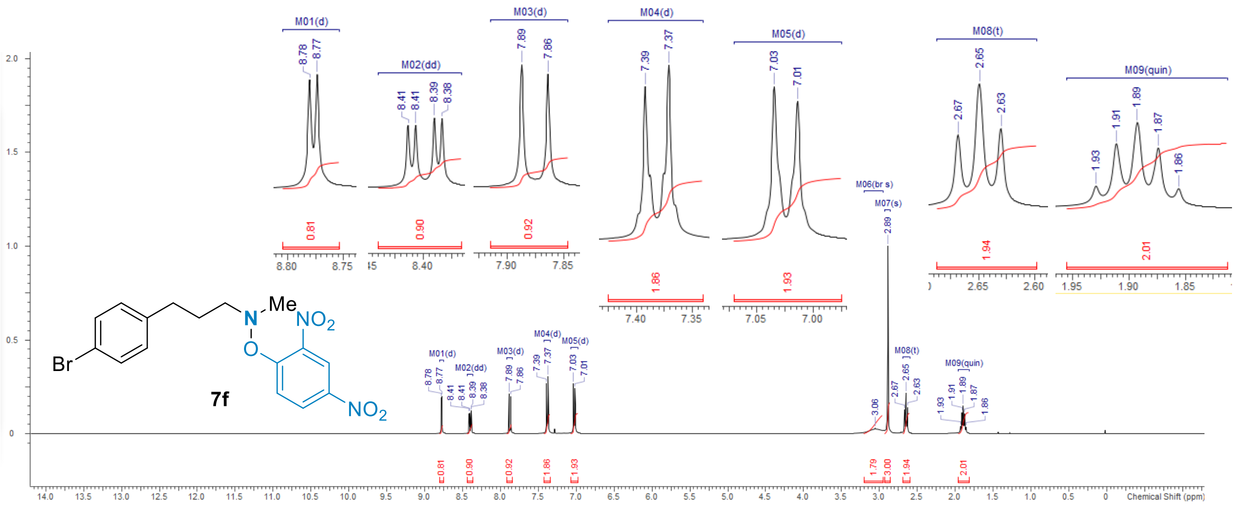

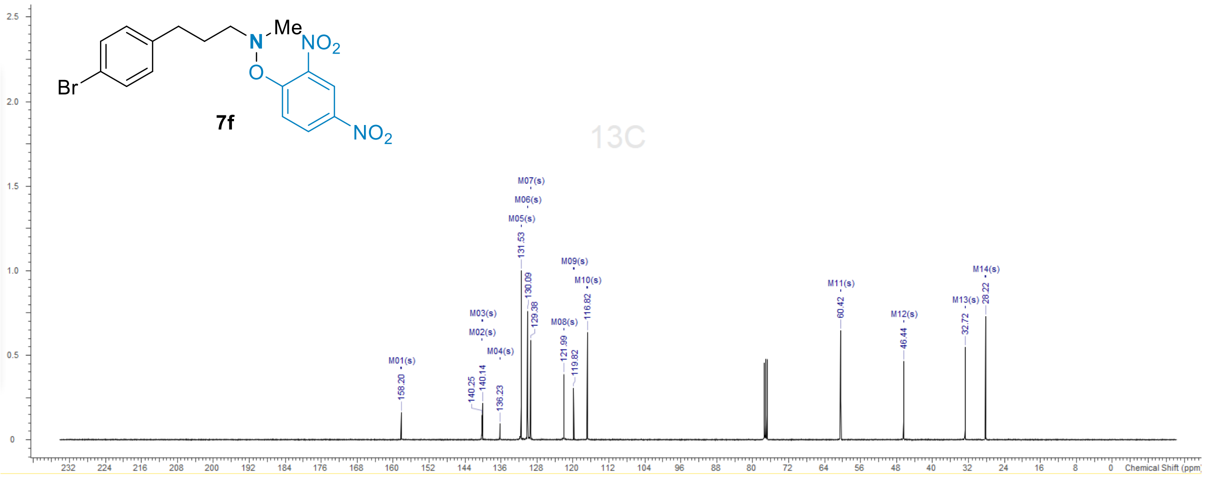

N-(3-(4-Chlorophenyl)propyl)-*O*-(2,4-dinitrophenyl)-*N*-methylhydroxylamine (**7g**)

Solvent: $CDCl_3$, ^1H NMR (400 MHz), ^{13}C NMR (101 MHz).

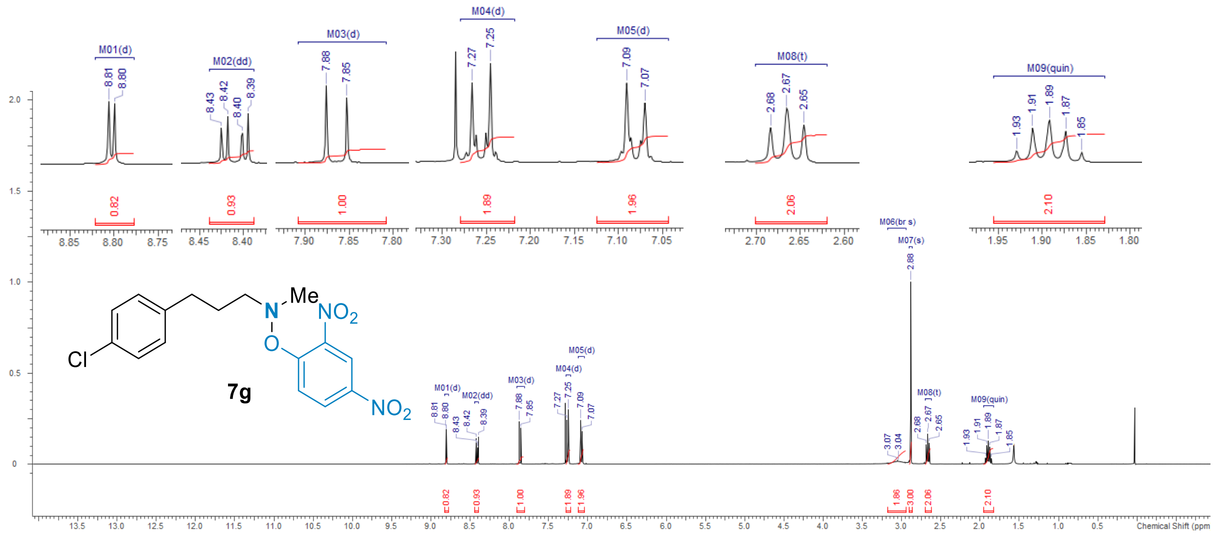

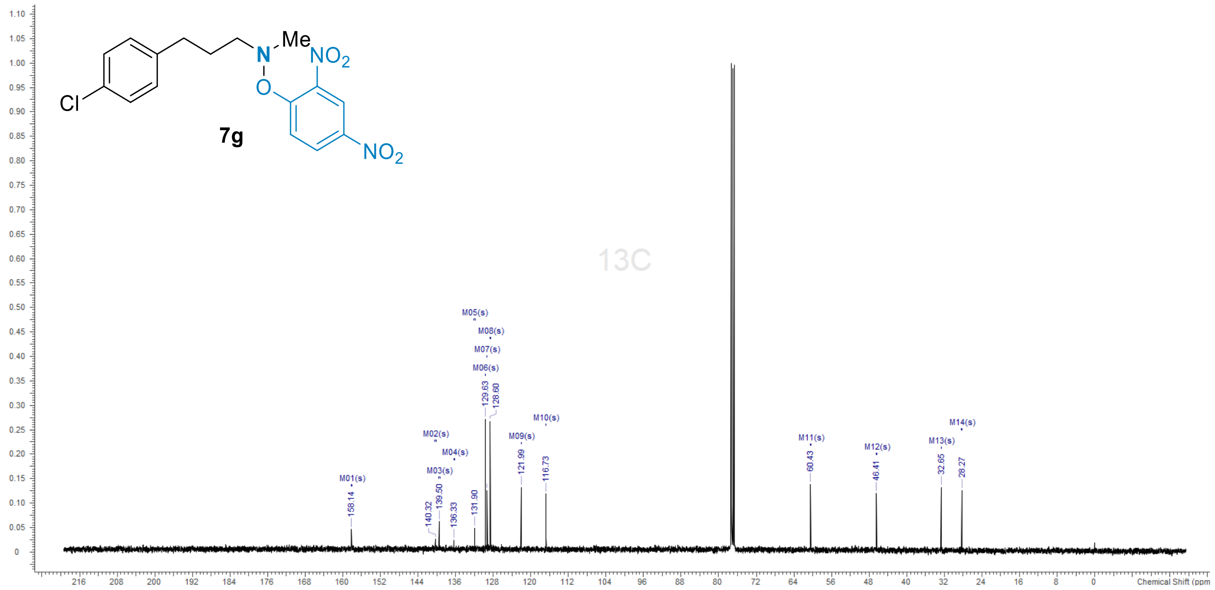

O-(2,4-Dinitrophenyl)-*N*-(3-(3-methoxyphenyl)propyl)-*N*-methylhydroxylamine (**7h**)

Solvent: $CDCl_3$, ^1H NMR (400 MHz), ^{13}C NMR (101 MHz).

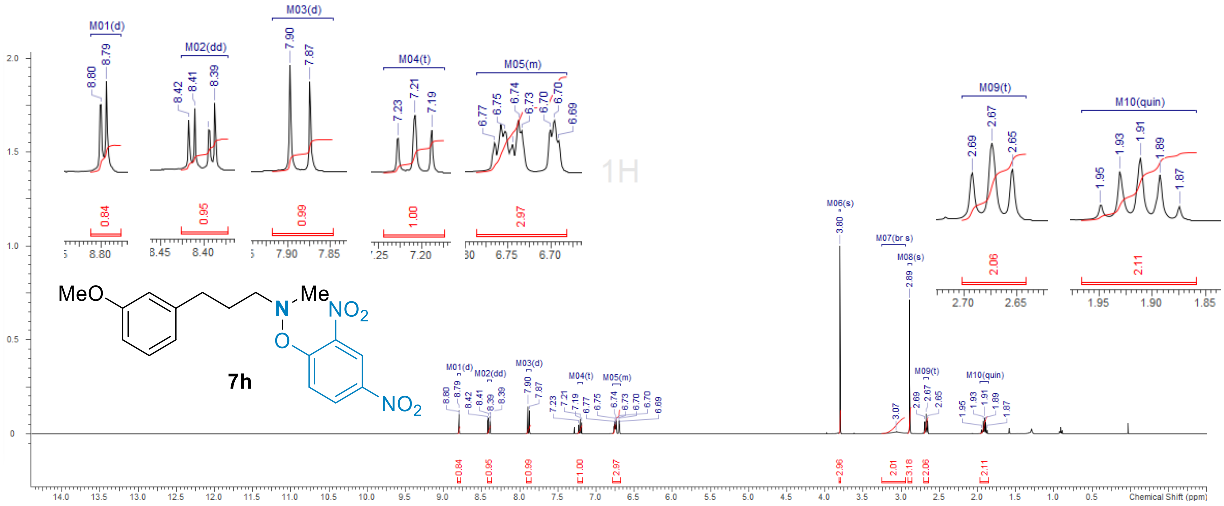

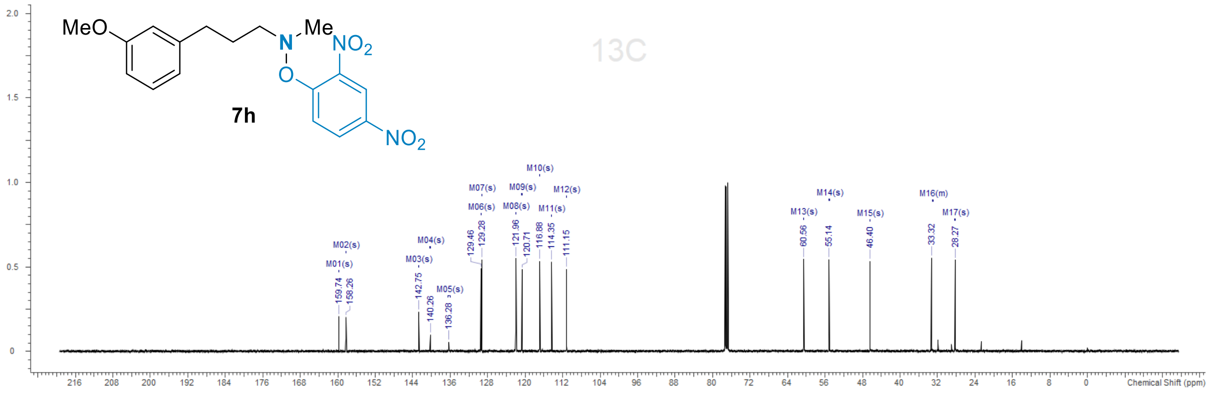

N-(3-(3-Bromophenyl)propyl)-*O*-(2,4-dinitrophenyl)-*N*-methylhydroxylamine (**7i**)

Solvent: $CDCl_3$, ^1H NMR (400 MHz), ^{13}C NMR (101 MHz).

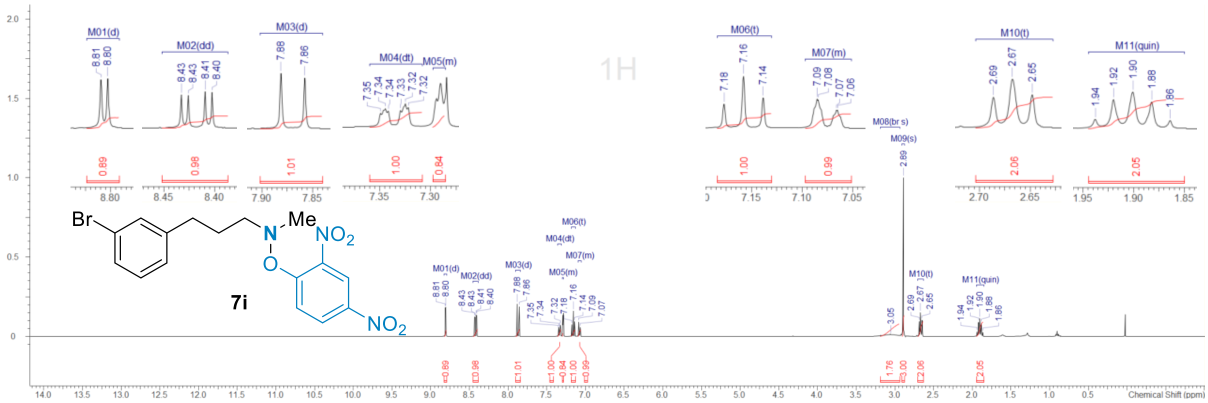

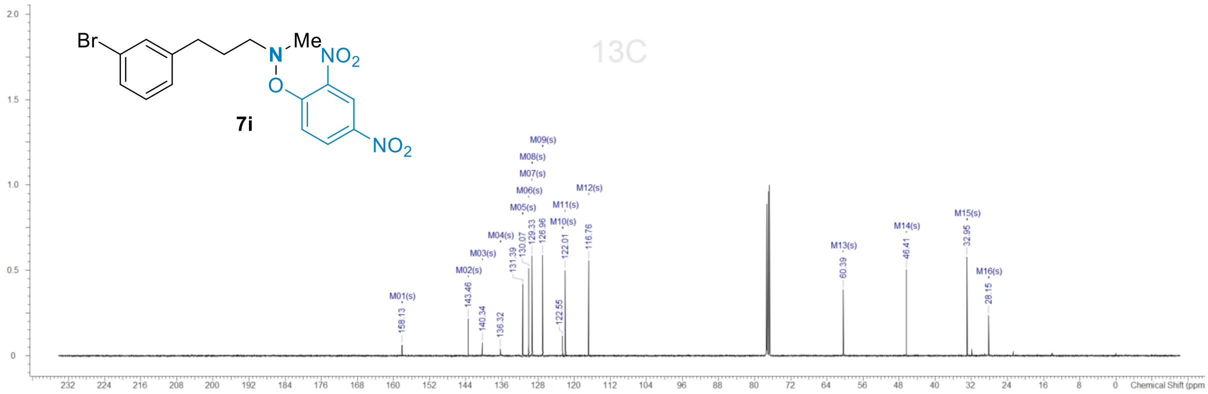

O-(2,4-Dinitrophenyl)-*N*-methyl-*N*-(3-(*p*-tolyl)propyl)hydroxylamine (**7j**)

Solvent: $CDCl_3$, ^1H NMR (400 MHz), ^{13}C NMR (101 MHz).

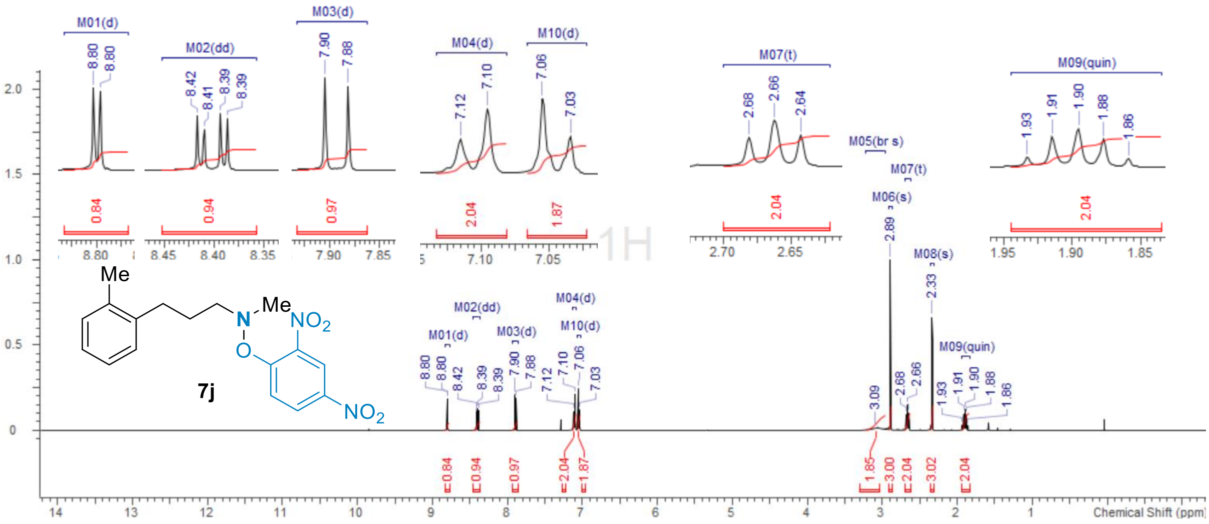

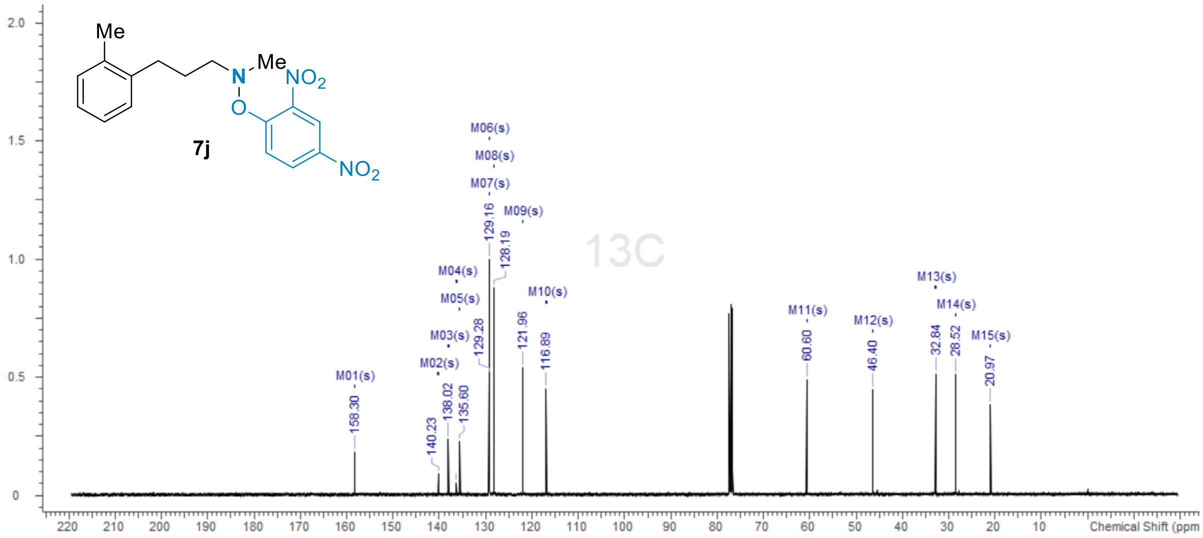

N-(3-(2-Fluorophenyl)propyl)-*O*-(2,4-dinitrophenyl)-*N*-methylhydroxylamine (**7k**)

Solvent: $CDCl_3$, ^1H NMR (400 MHz), ^{19}F NMR: (376 MHz) ^{13}C NMR (151 MHz).

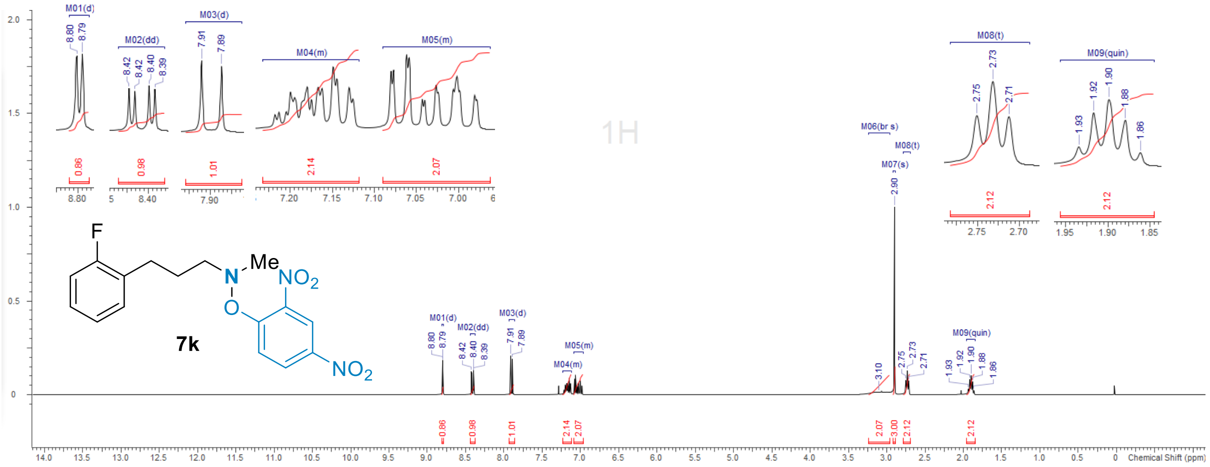

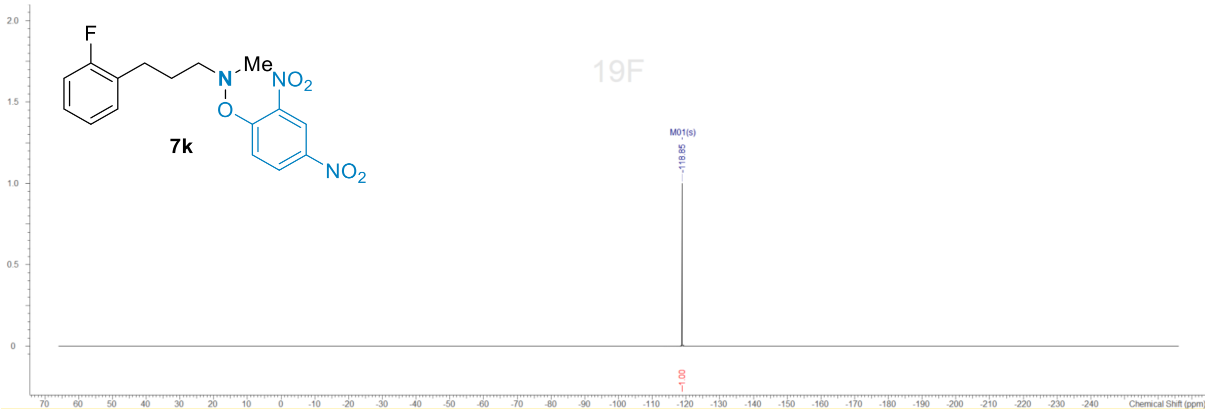

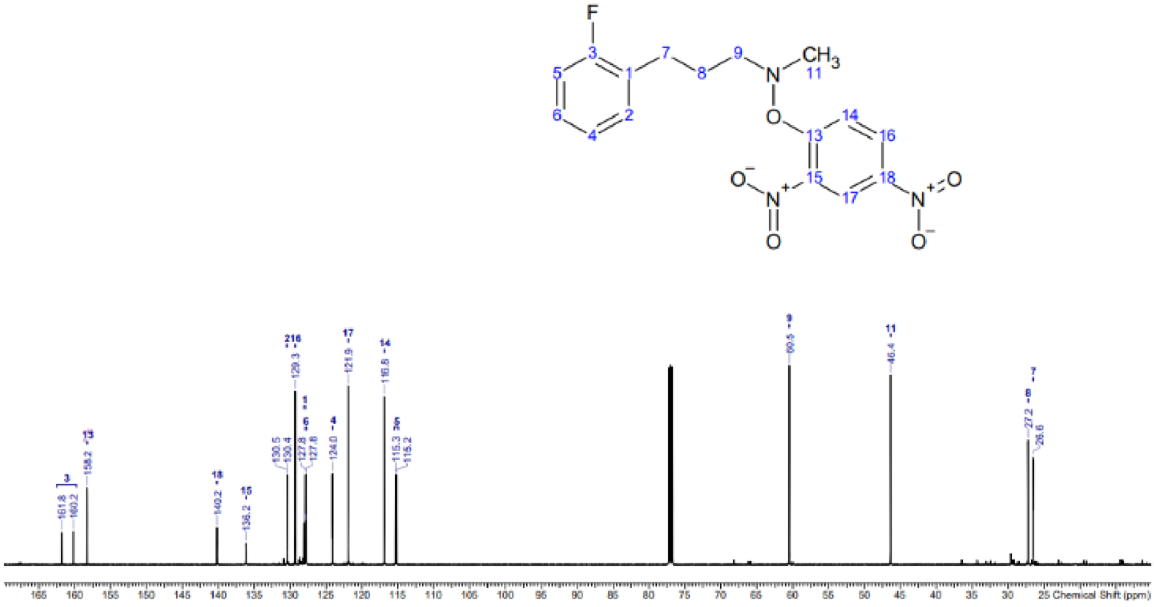

N-(3-(2-Bromophenyl)propyl)-*O*-(2,4-dinitrophenyl)-*N*-methylhydroxylamine (**7l**)

Solvent: $CDCl_3$, ^1H NMR (400 MHz), ^{13}C NMR (101 MHz).

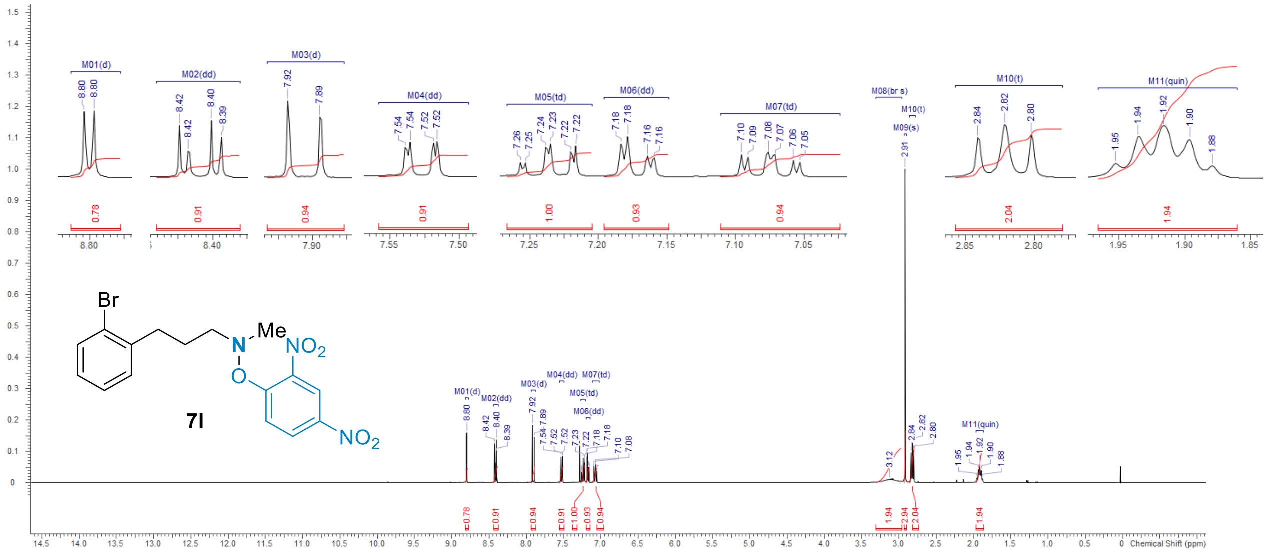

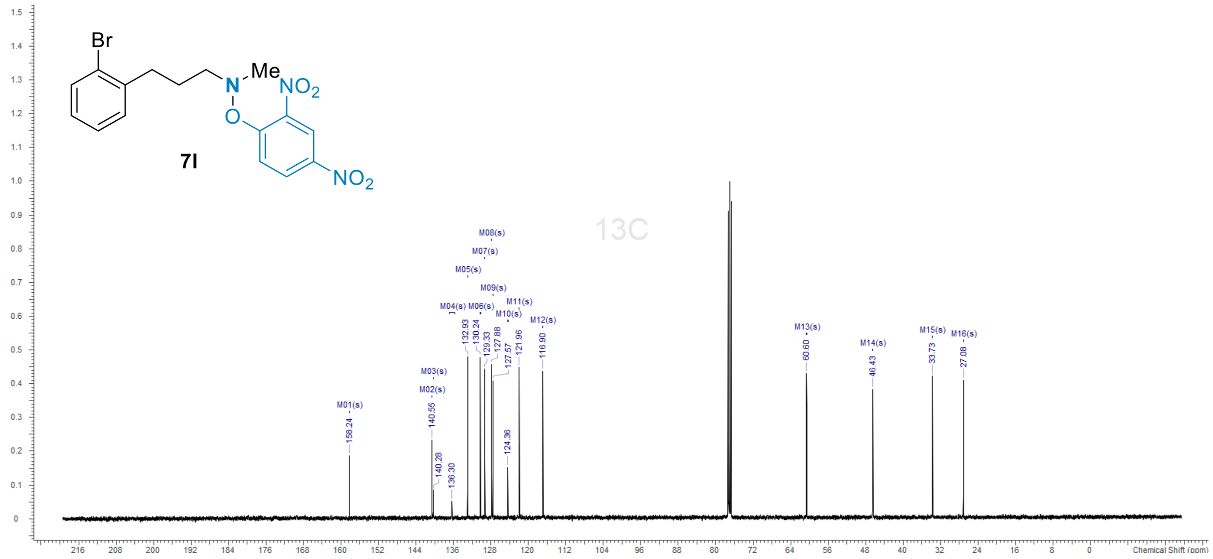

O-(2,4-Dinitrophenyl)-*N*-methyl-*N*-(3-phenylbutyl)hydroxylamine (**7m**)

Solvent: $CDCl_3$, ^1H NMR (400 MHz), ^{13}C NMR (101 MHz).

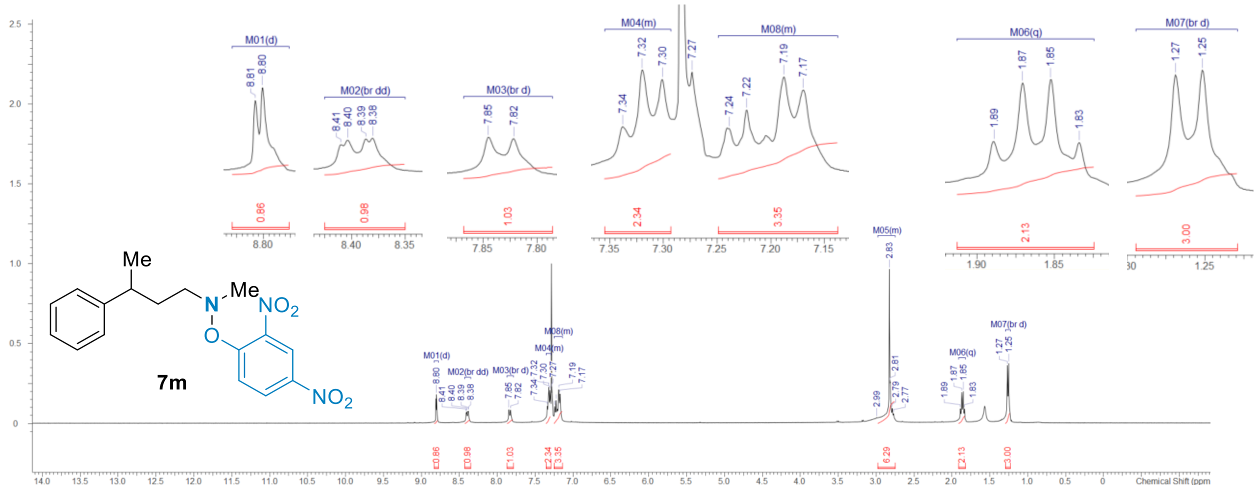

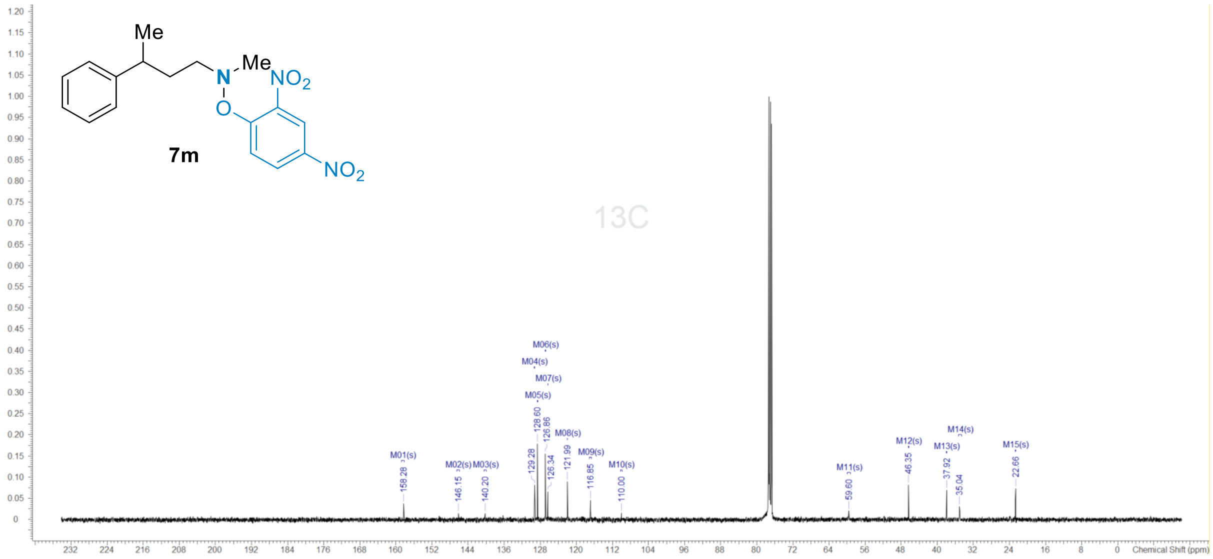

O-(2,4-Dinitrophenyl)-*N*-(3,3-diphenylpropyl)-*N*-methylhydroxylamine (**7n**)

Solvent: $CDCl_3$, ^{1}H NMR (400 MHz), ^{13}C NMR (101 MHz).

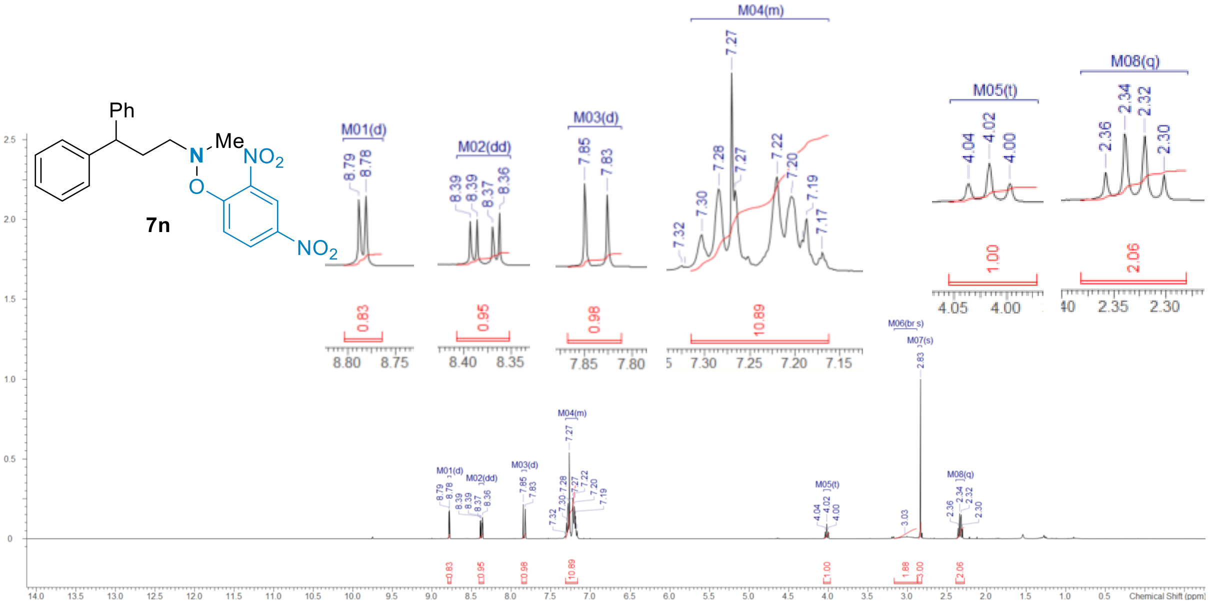

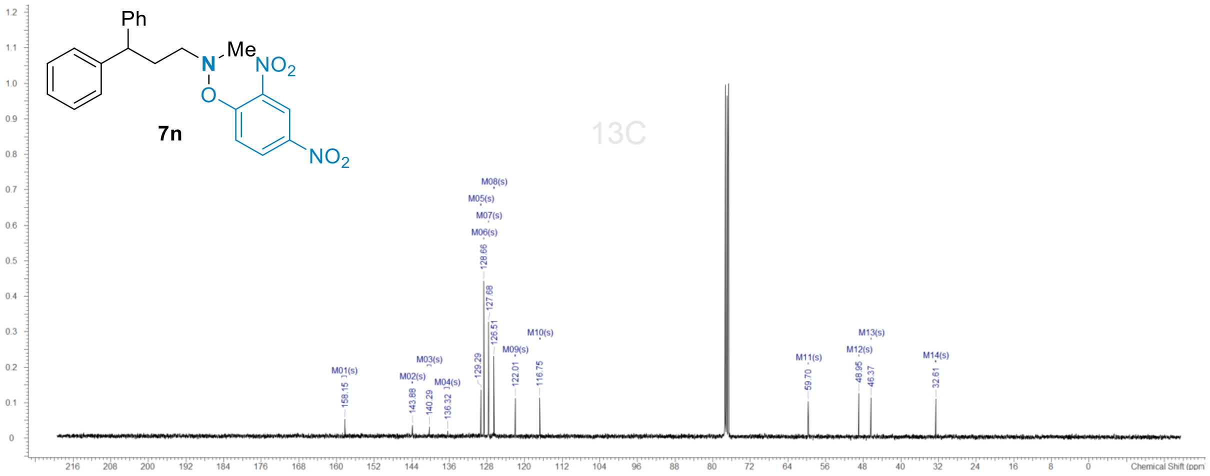

O-(2,4-Dinitrophenyl)-*N*-methyl-*N*-(2-phenoxyethyl)hydroxylamine (**7o**)

Solvent: $CDCl_3$, ^{1}H NMR (400 MHz), ^{13}C NMR (101 MHz).

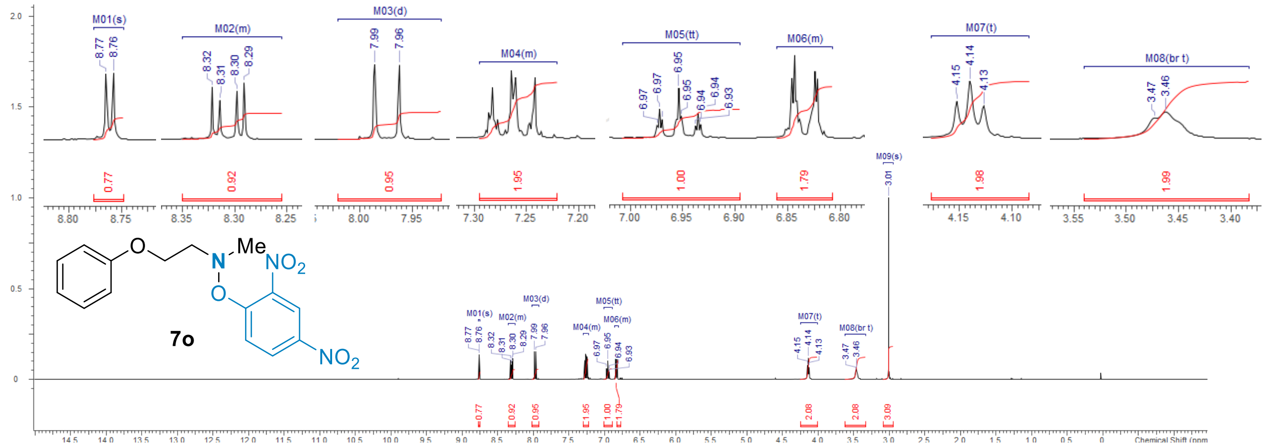

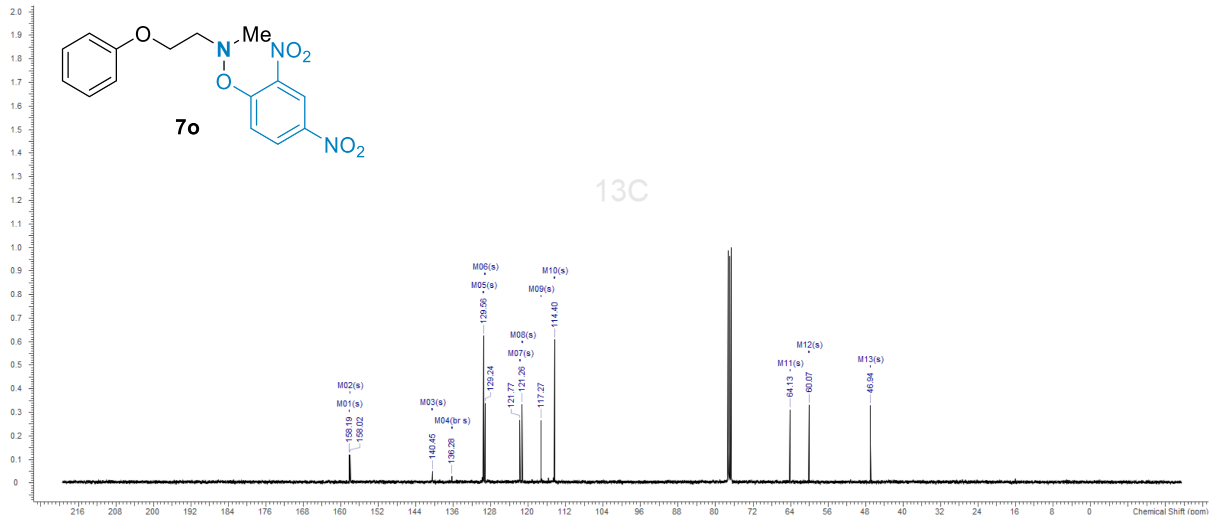

1-Methyl-1,2,3,4-tetrahydroquinoline (**8a**)

Solvent: $CDCl_3$, ^{1}H NMR (400 MHz), ^{13}C NMR (101 MHz).

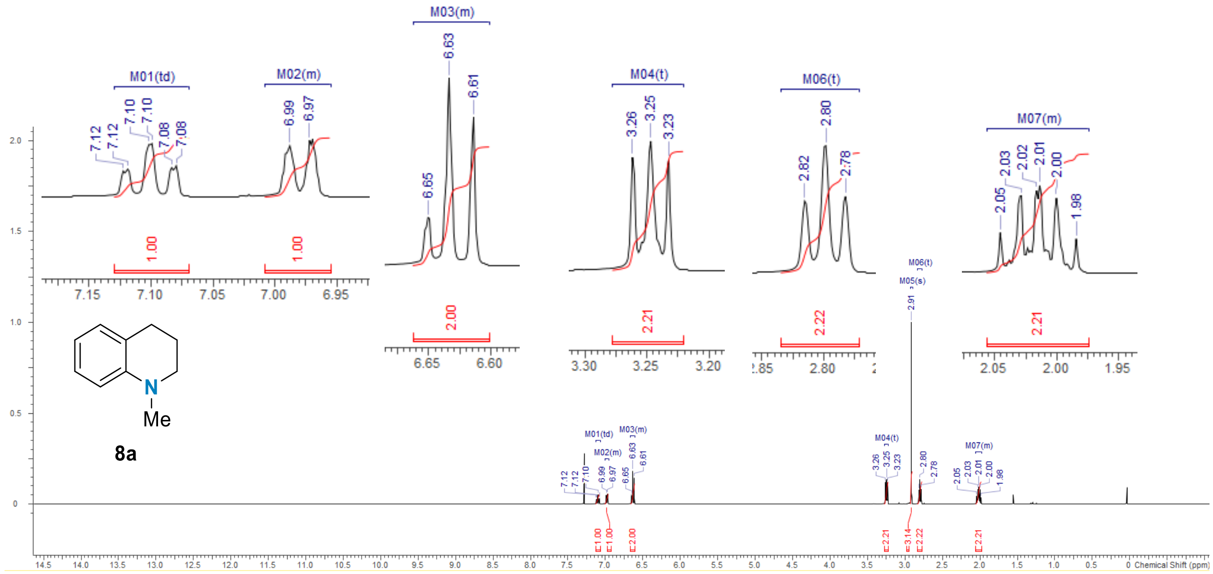

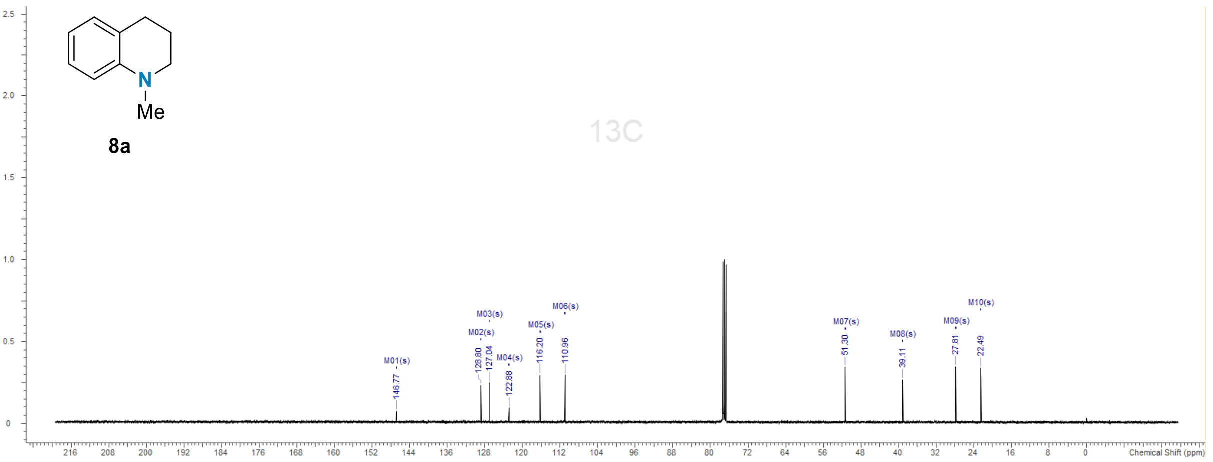

1,7-Dimethyl-1,2,3,4-tetrahydroquinoline (**8b**)

Solvent: $CDCl_3$, ^{1}H NMR (400 MHz), ^{13}C NMR (101 MHz).

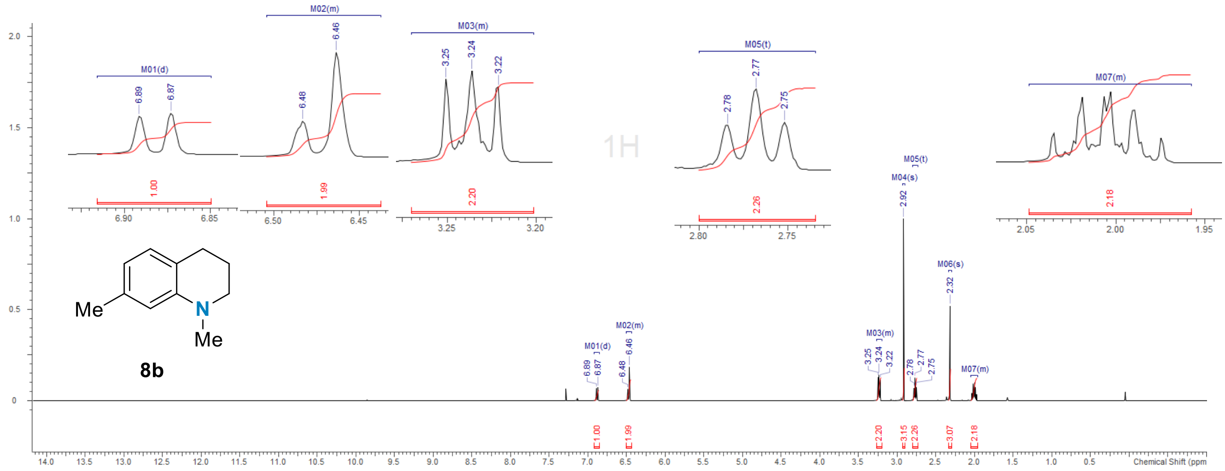

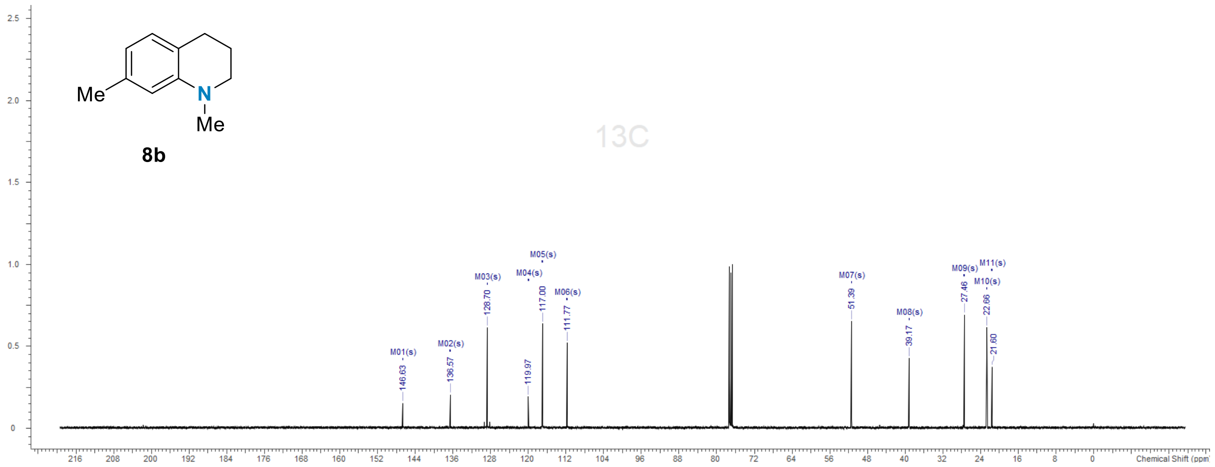

7-(*tert*-Butyl)-1-methyl-1,2,3,4-tetrahydroquinoline (**8c**)

Solvent: $CDCl_3$, ^{1}H NMR (400 MHz), ^{13}C NMR (101 MHz).

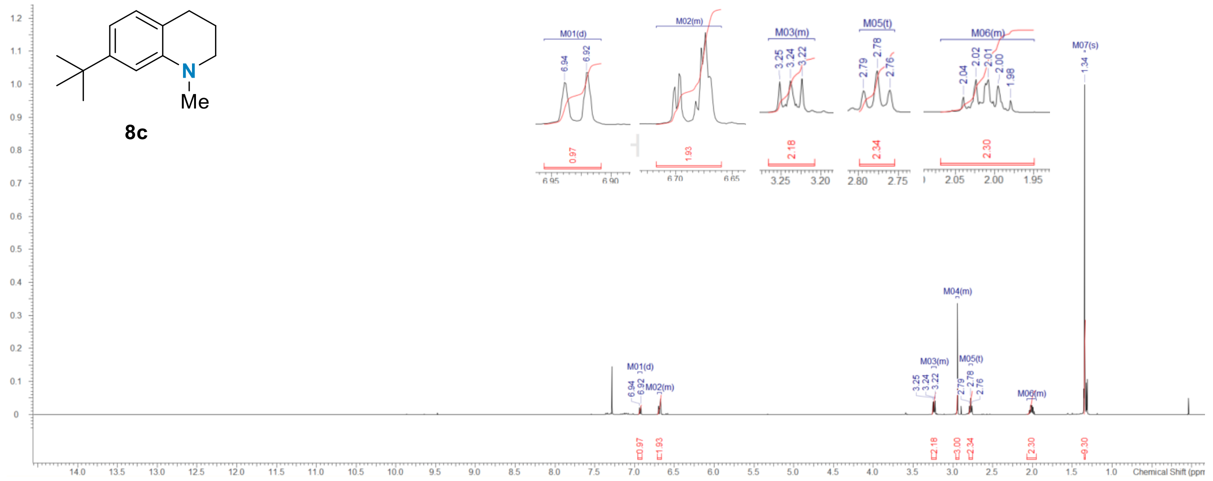

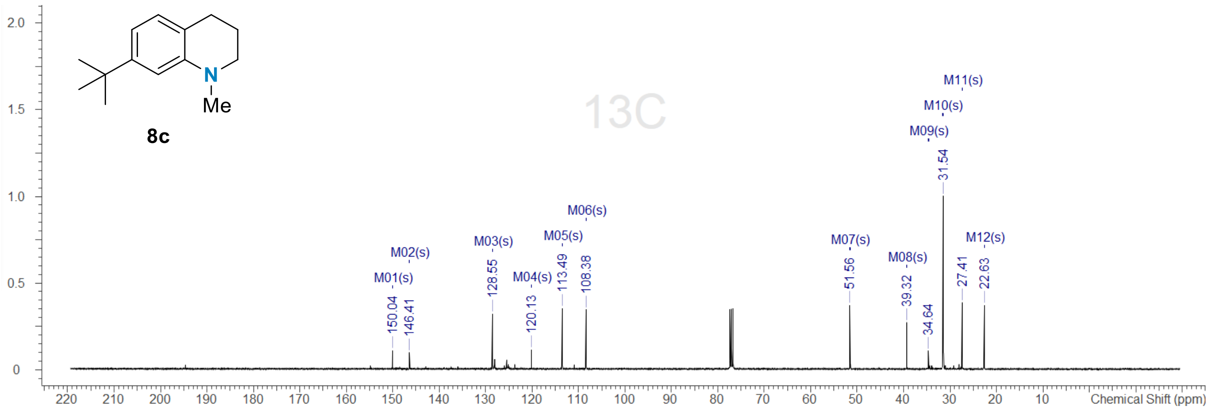

1-Methyl-7-phenyl-1,2,3,4-tetrahydroquinoline and 1-methyl-6-phenyl-1,2,3,4-tetrahydroquinoline (8d’ + 8d”)

Solvent: $CDCl_3$, ^1H NMR (600 MHz), ^{13}C NMR (151 MHz).

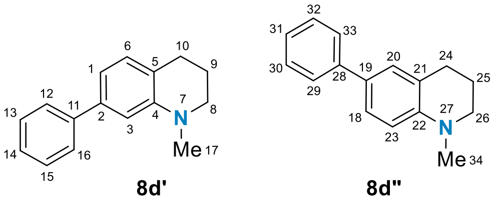

8d' **8d''**

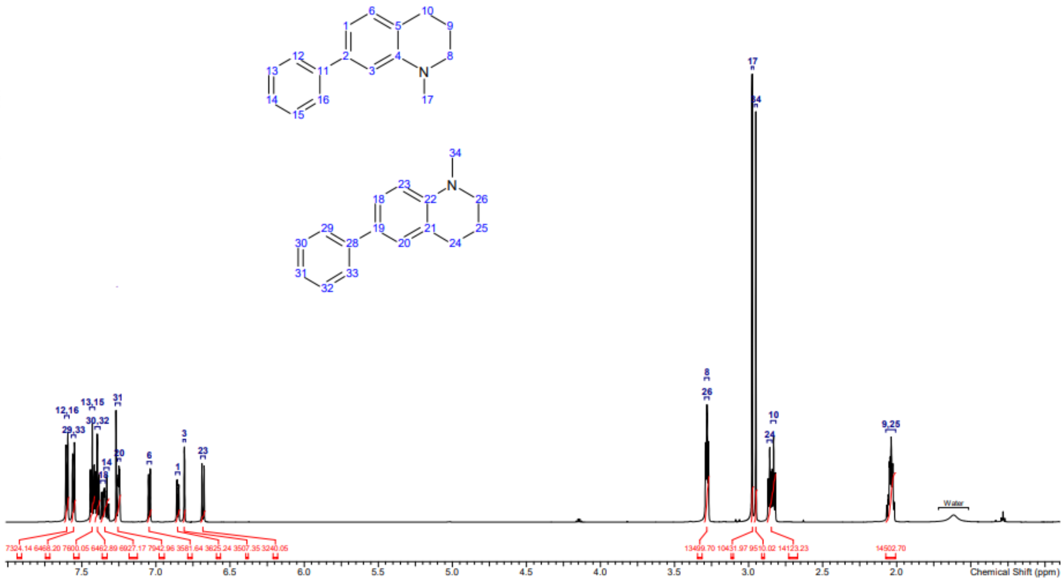

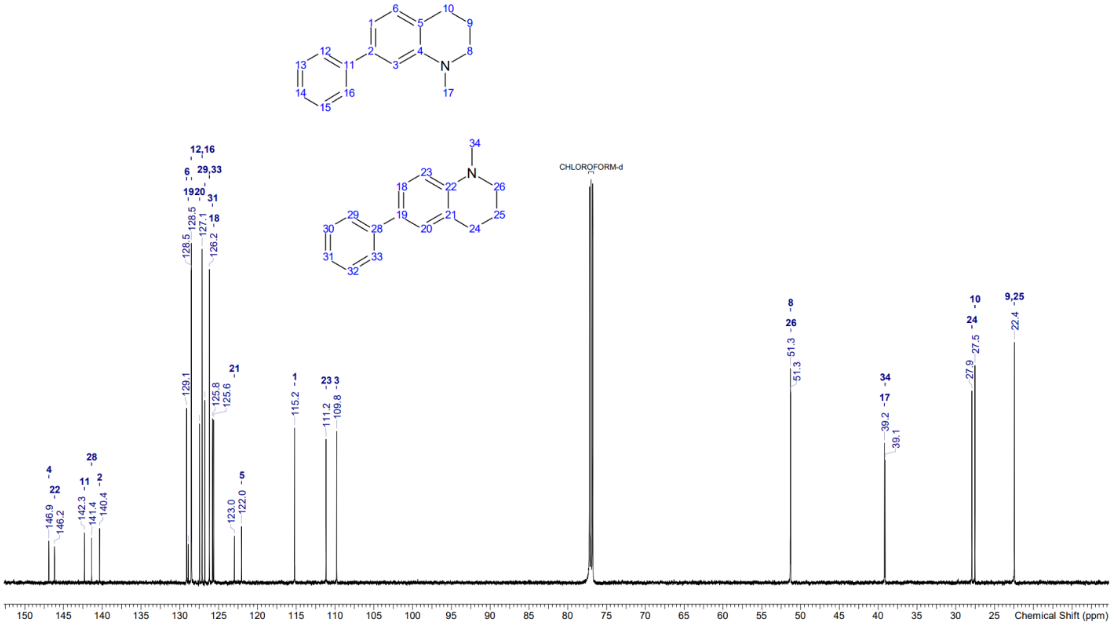

COSY

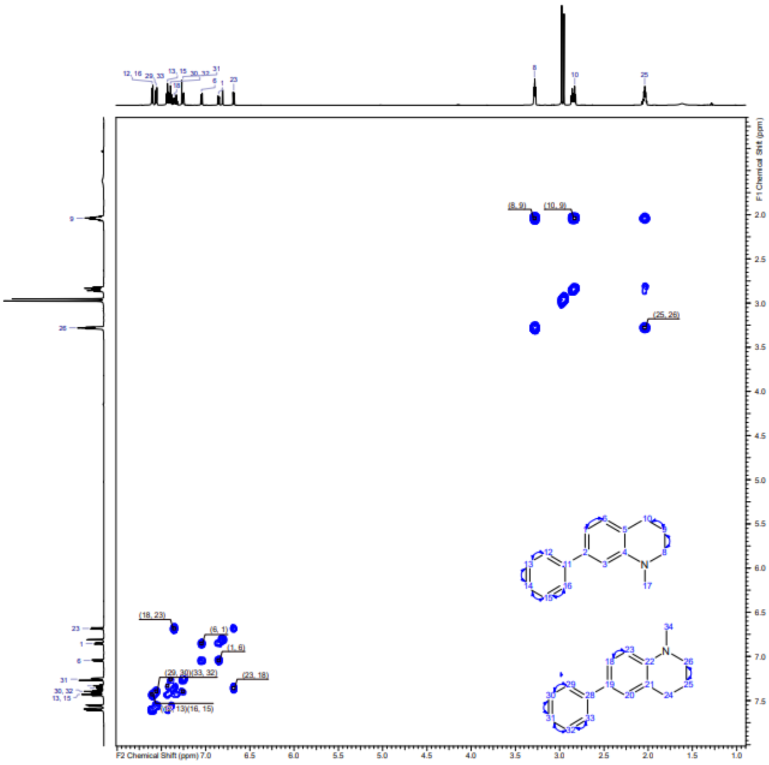

ROESY

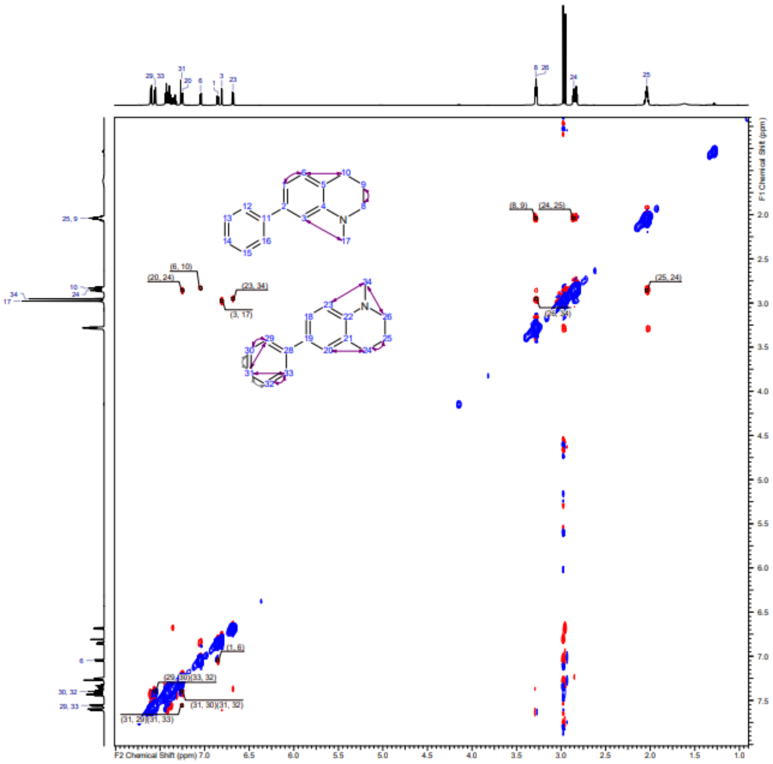

HSQC-DEPT

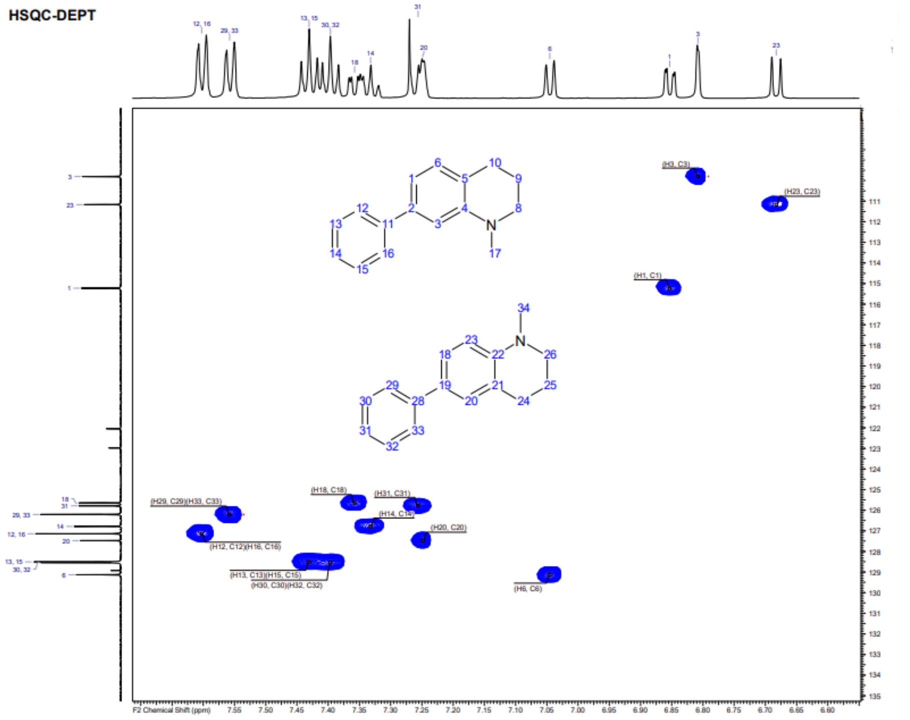

HMBC

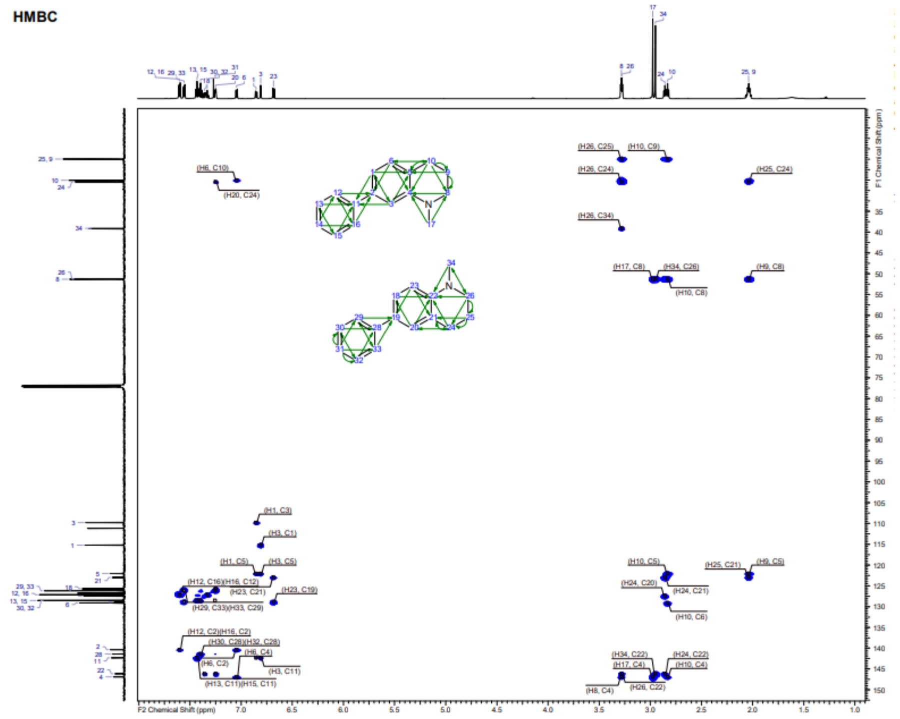

7-Methoxy-1-methyl-1,2,3,4-tetrahydroquinoline (**8e**)

Solvent: $CDCl_3$, ^{1}H NMR (400 MHz), ^{13}C NMR (101 MHz).

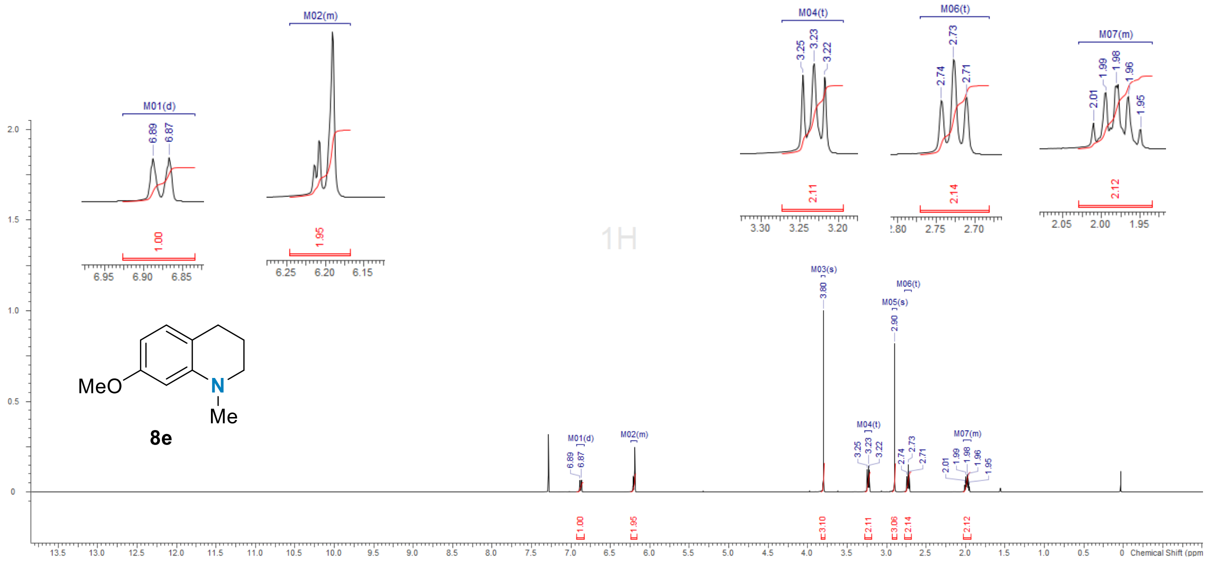

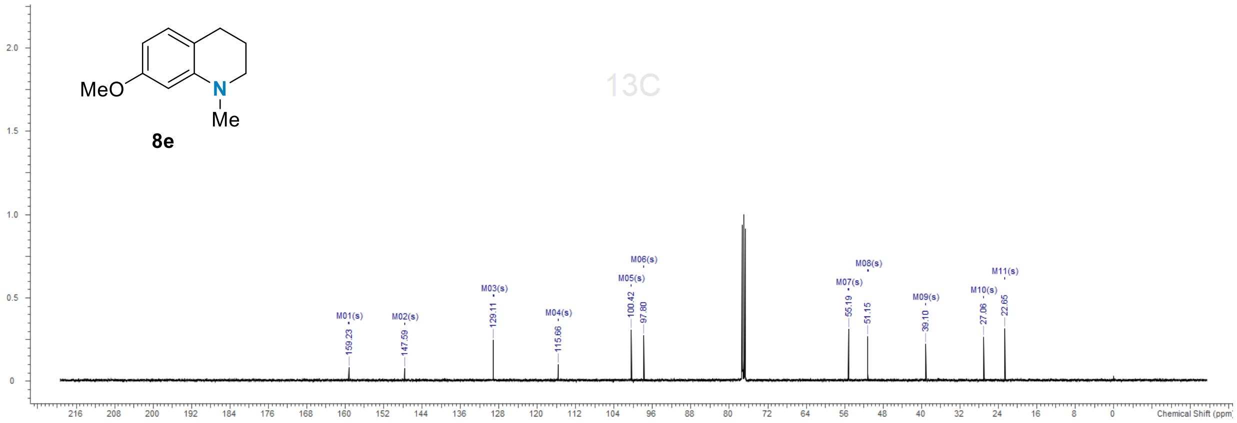

7-Bromo-1-methyl-1,2,3,4-tetrahydroquinoline (**8f**)

Solvent: $CDCl_3$, ^1H NMR (400 MHz), ^{13}C NMR (101 MHz).

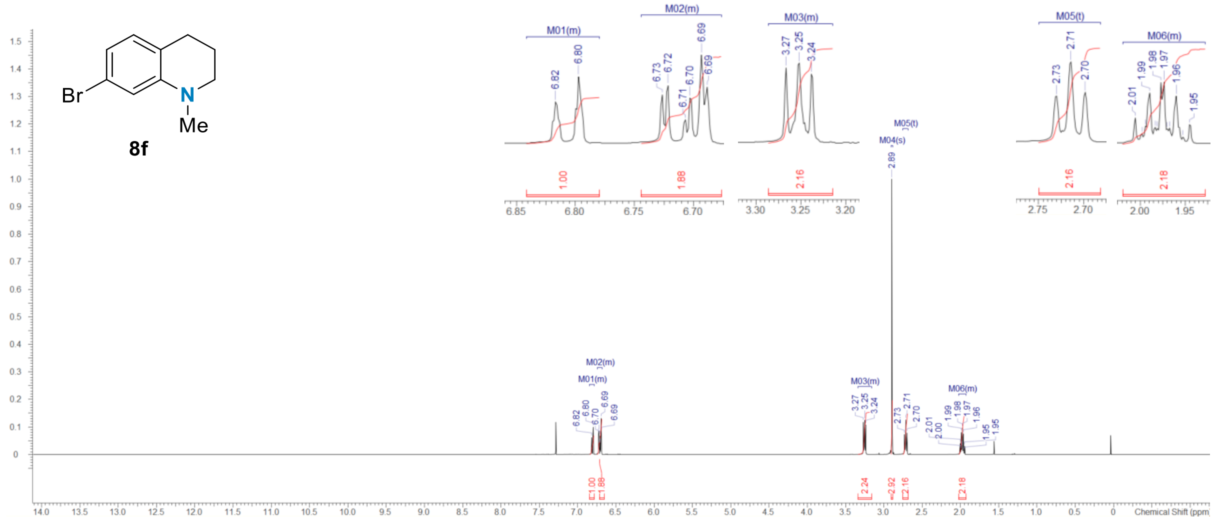

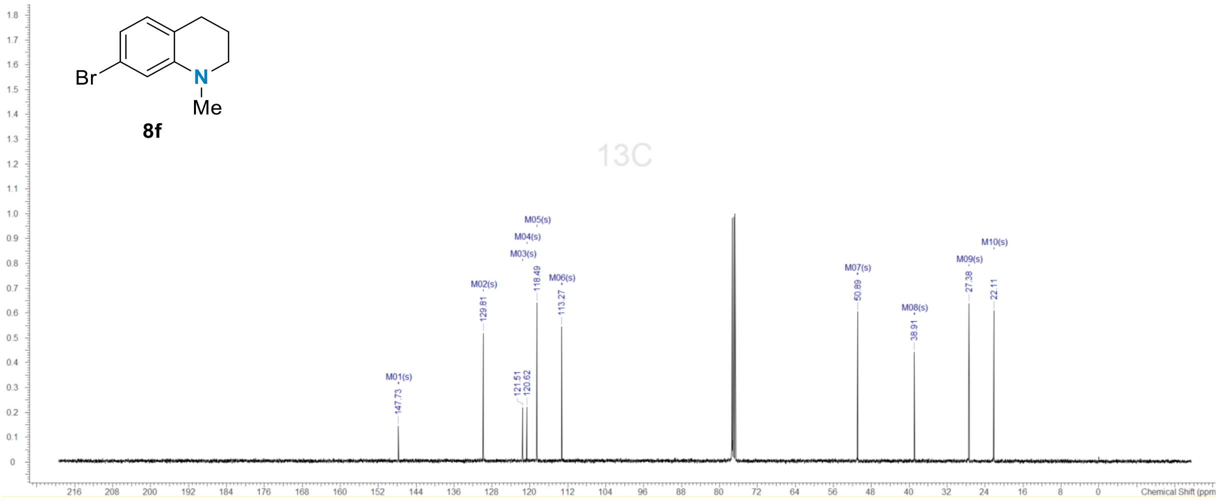

7-Chloro-1-methyl-1,2,3,4-tetrahydroquinoline (**8g**)

Solvent: $CDCl_3$, ^{1}H NMR (400 MHz), ^{13}C NMR (101 MHz).

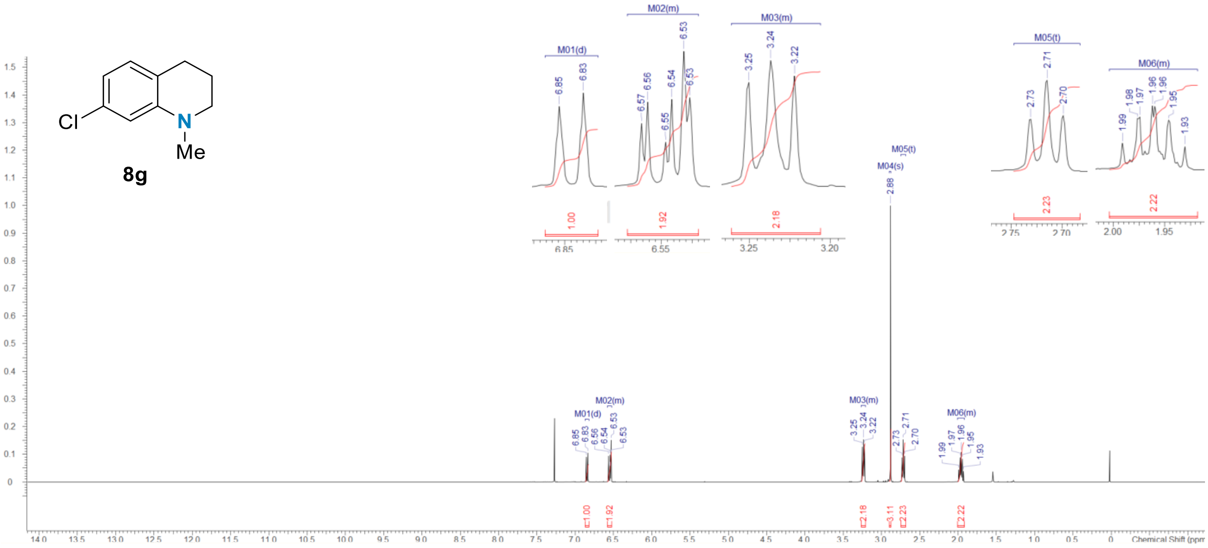

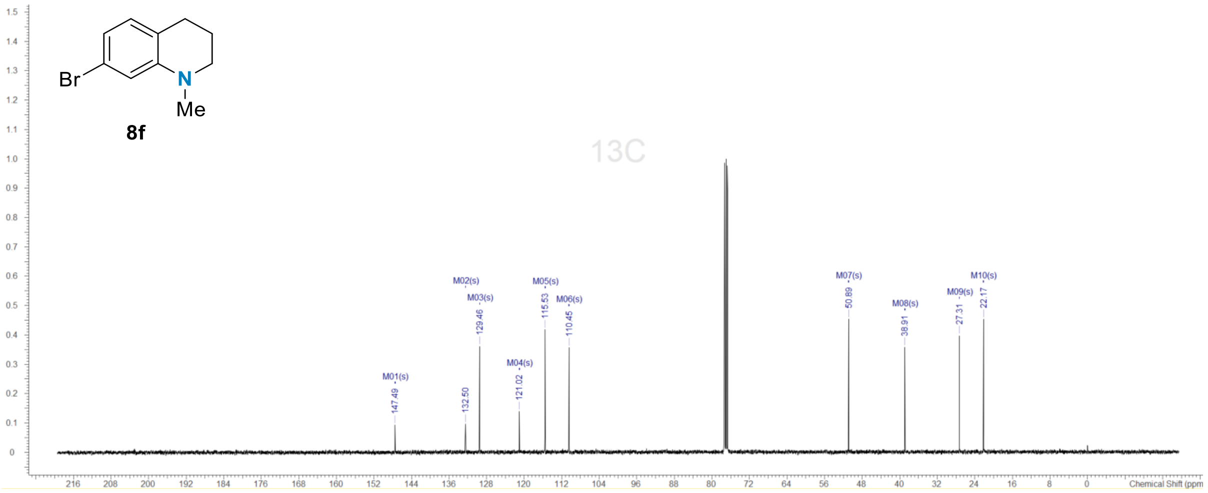

6-Methoxy-1-methyl-1,2,3,4-tetrahydroquinoline (**8h’**)

Solvent: $CDCl_3$, ^{1}H NMR (400 MHz), ^{13}C NMR (101 MHz).

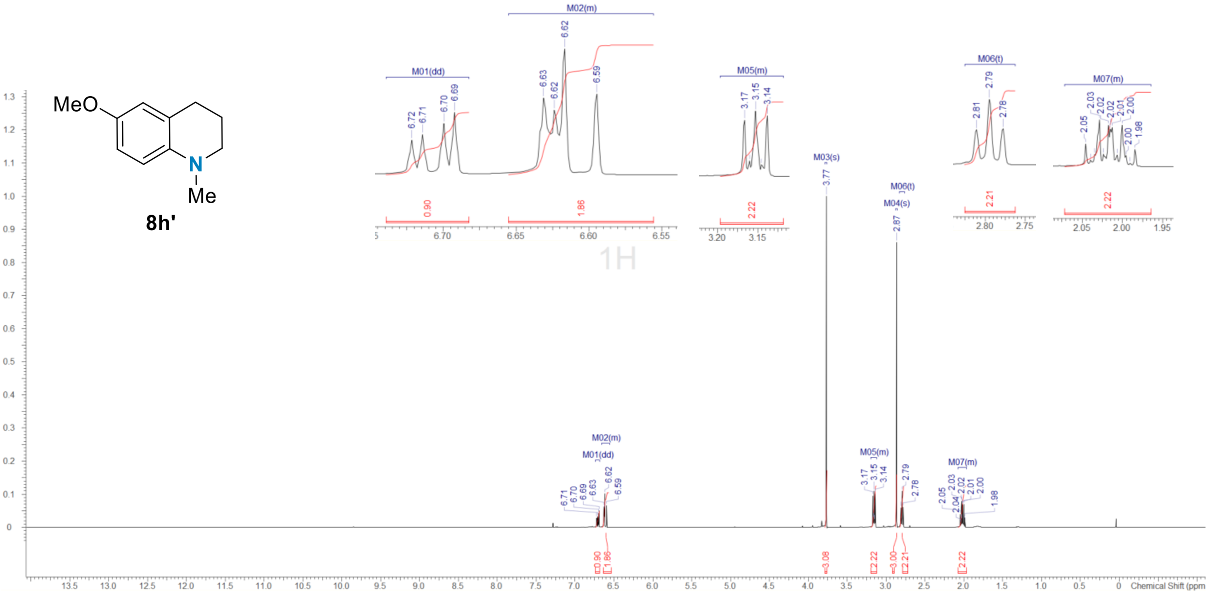

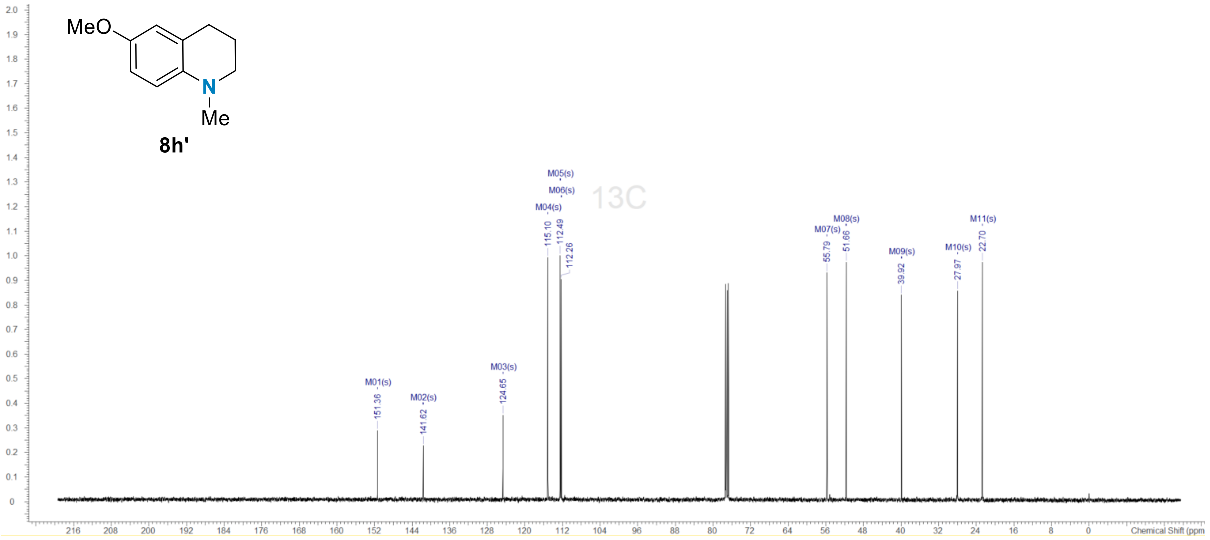

8-Methoxy-1-methyl-1,2,3,4-tetrahydroquinoline (**8h”**)

Solvent: $CDCl_3$, ^1H NMR (400 MHz), ^{13}C NMR (101 MHz).

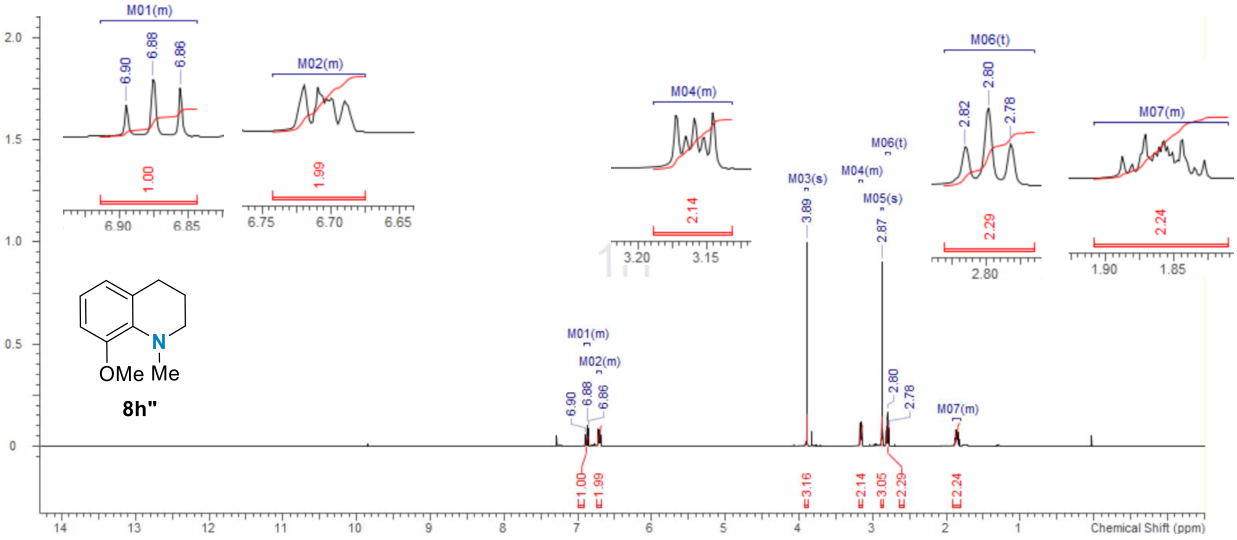

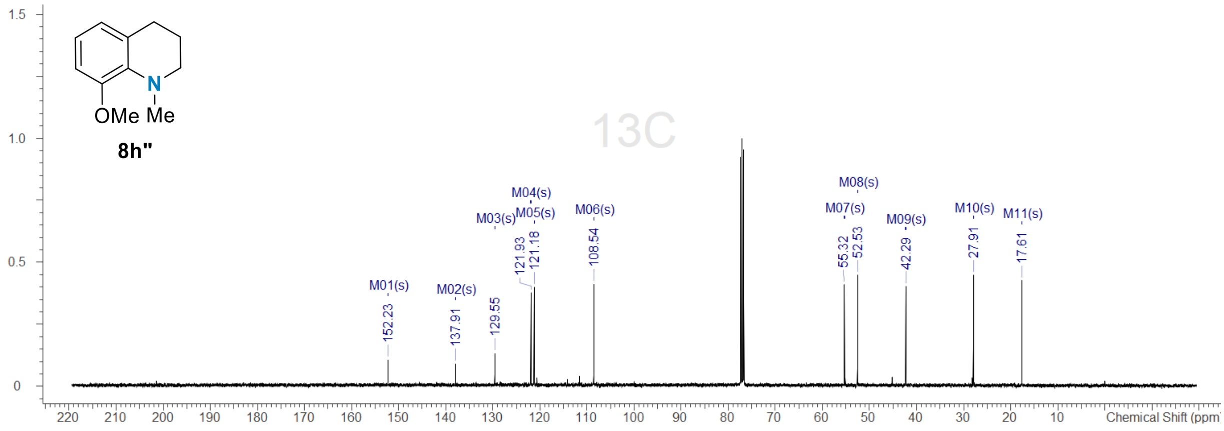

6-Bromo-1-methyl-1,2,3,4-tetrahydroquinoline (**8i**)

Solvent: $CDCl_3$, ^{1}H NMR (400 MHz), ^{13}C NMR (101 MHz).

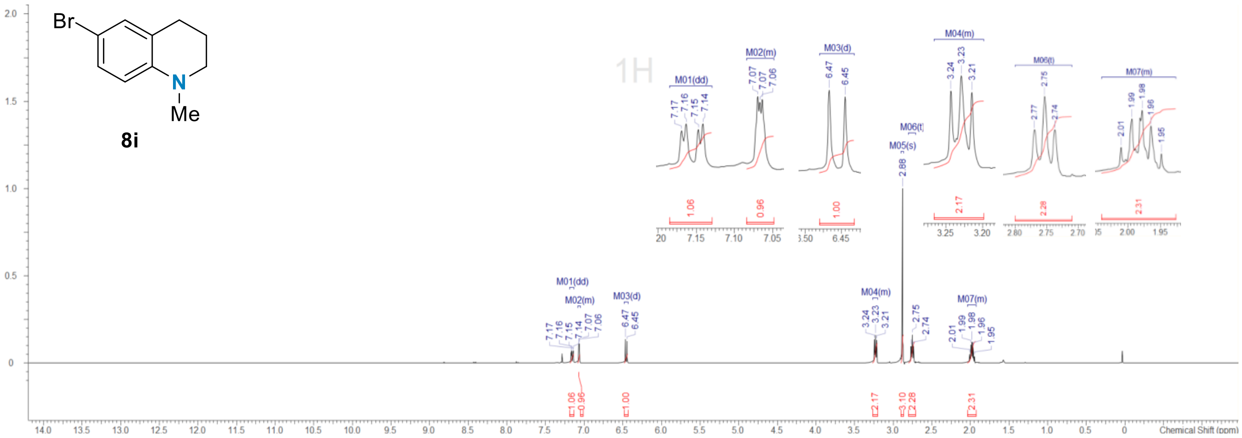

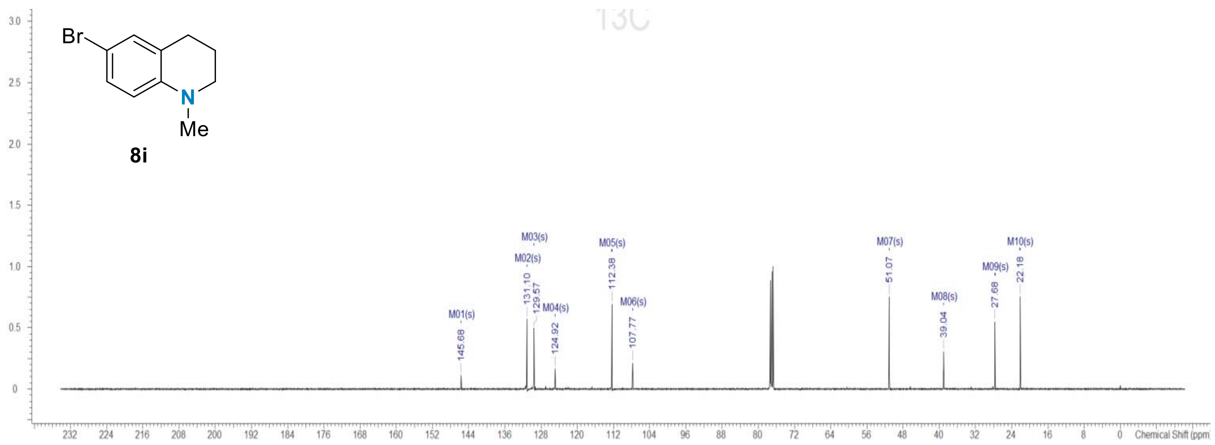

1,5-Dimethyl-1,2,3,4-tetrahydroquinoline (**8j**)

Solvent: $CDCl_3$, ^1H NMR (400 MHz), ^{13}C NMR (101 MHz).

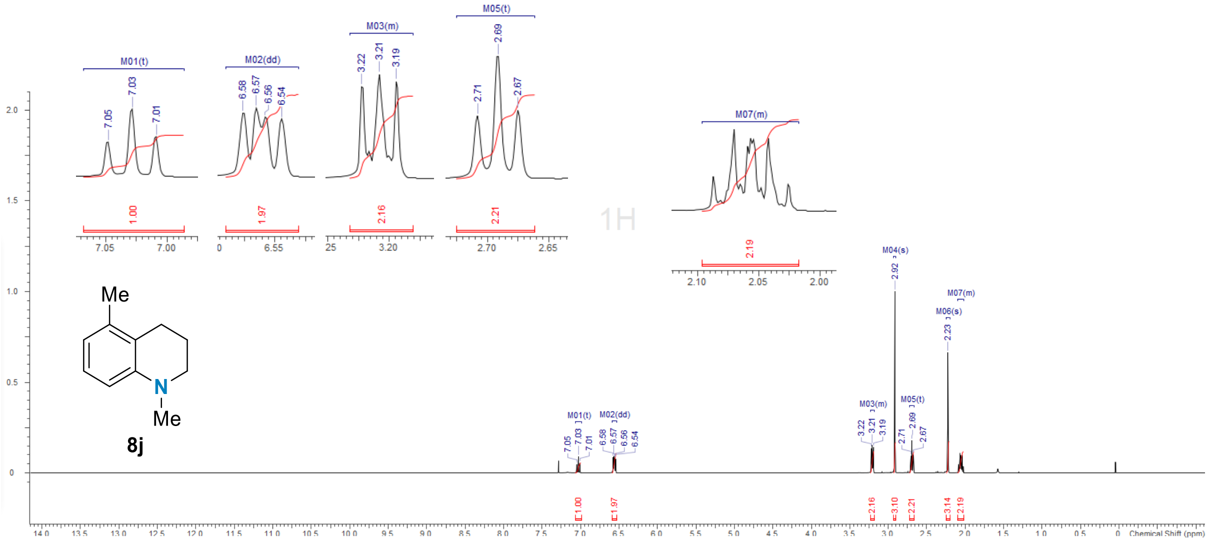

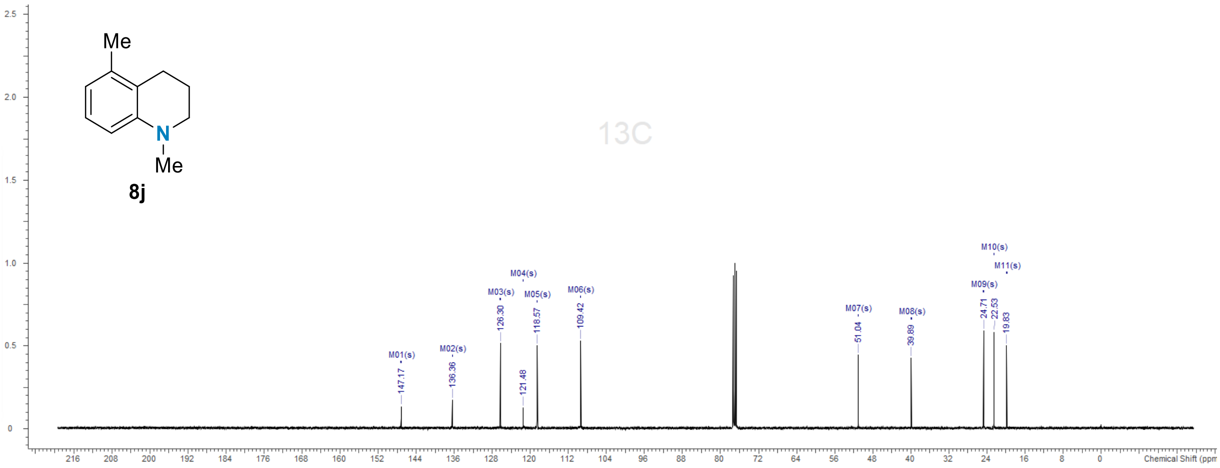

5-Fluoro-1-methyl-1,2,3,4-tetrahydroquinoline (**8k**)

Solvent: $CDCl_3$, ^1H NMR (400 MHz), ^{19}F NMR (376 MHz), ^{13}C NMR (101 MHz).

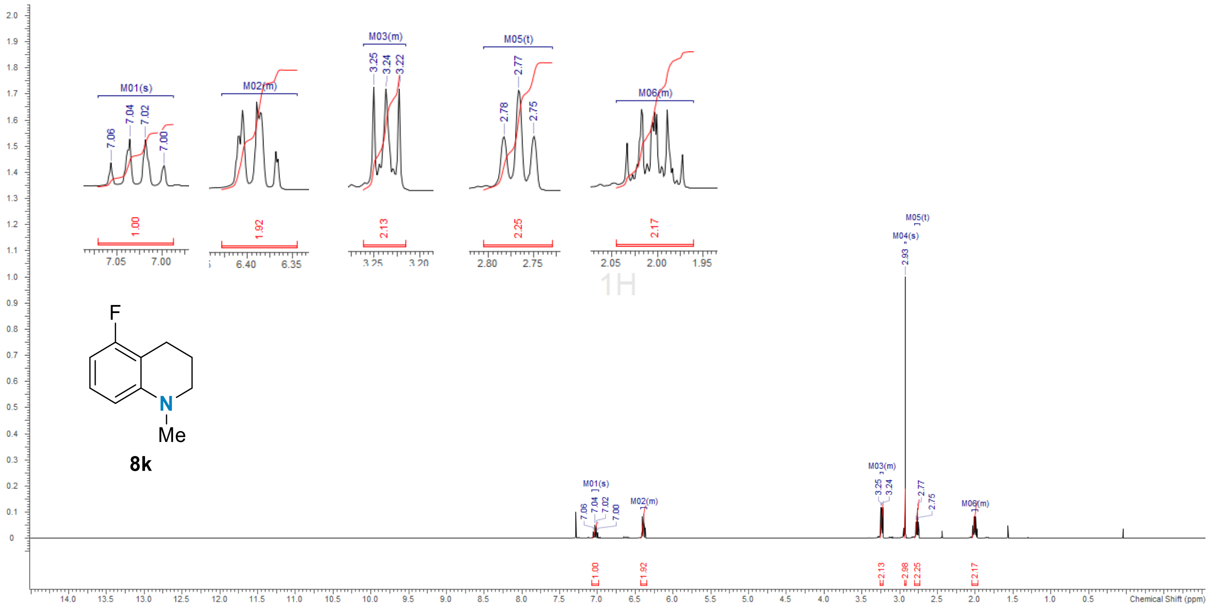

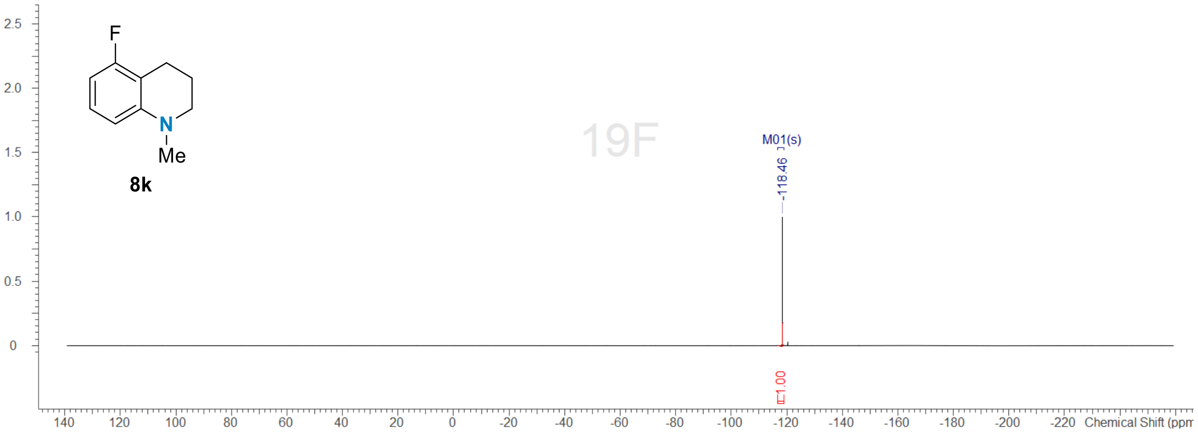

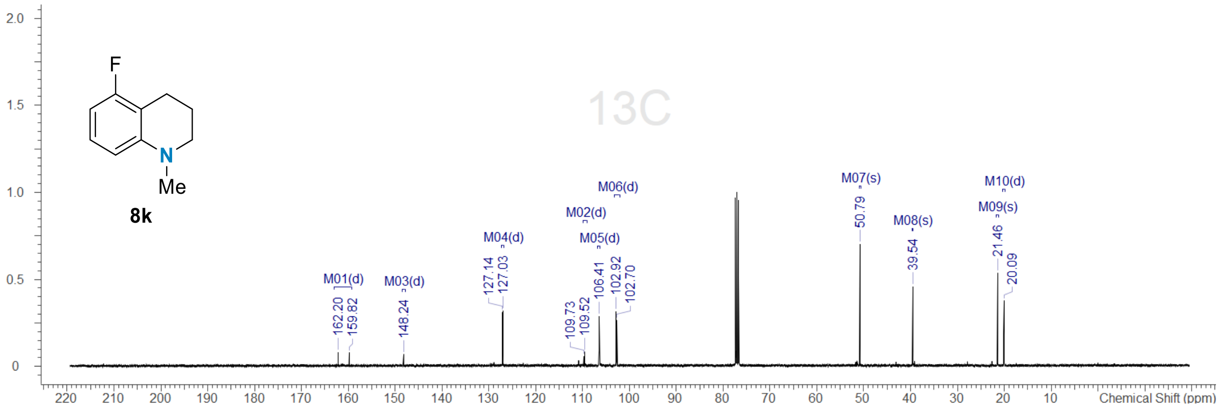

5-Bromo-1-methyl-1,2,3,4-tetrahydroquinoline (**8l**)

Solvent: $CDCl_3$, ^{1}H NMR (400 MHz), ^{13}C NMR (101 MHz).

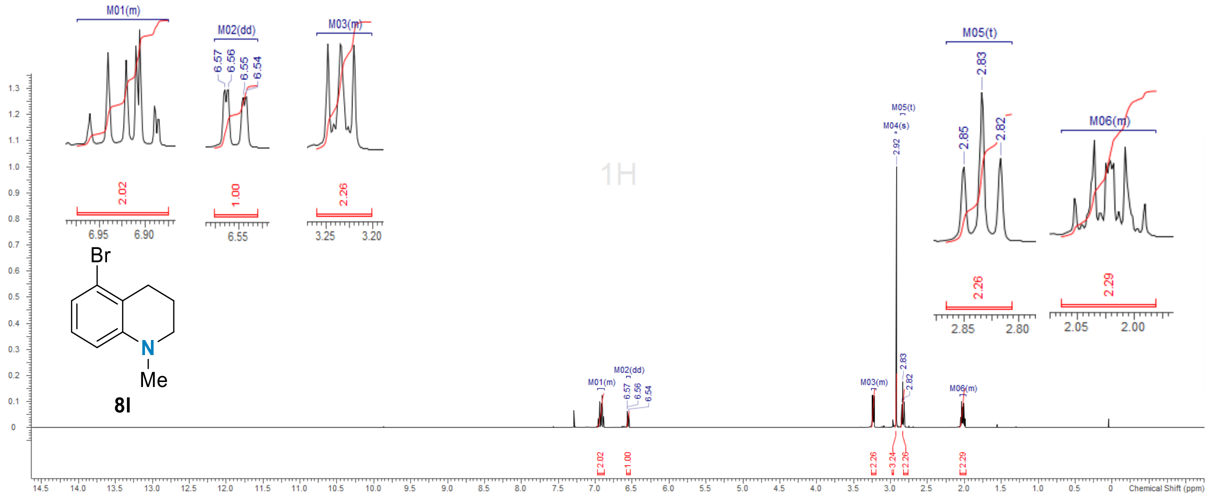

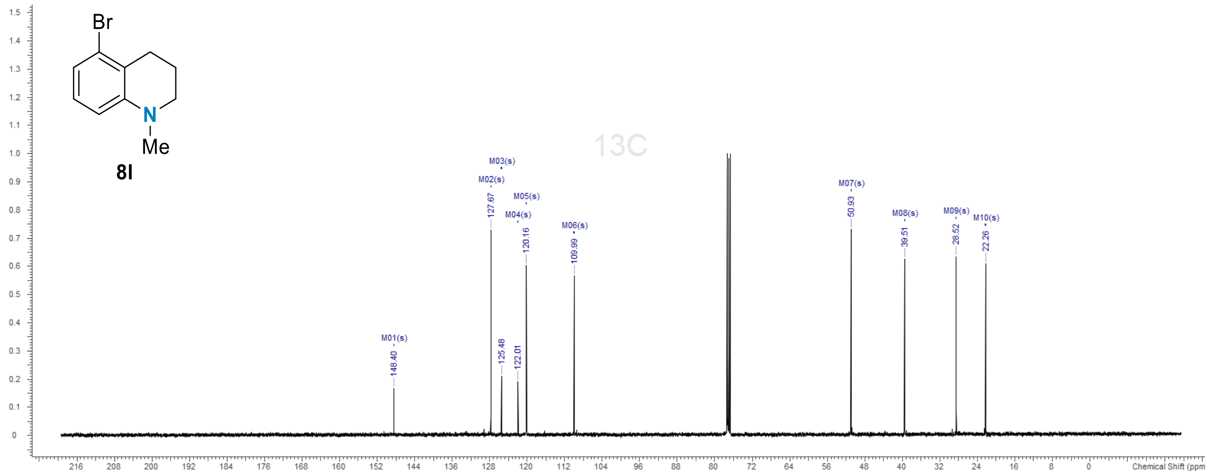

1,4-Dimethyl-3,4-dihydro-2*H*-quinoline (**8m**)

Solvent: $CDCl_3$, ^{1}H NMR (400 MHz), ^{13}C NMR (101 MHz).

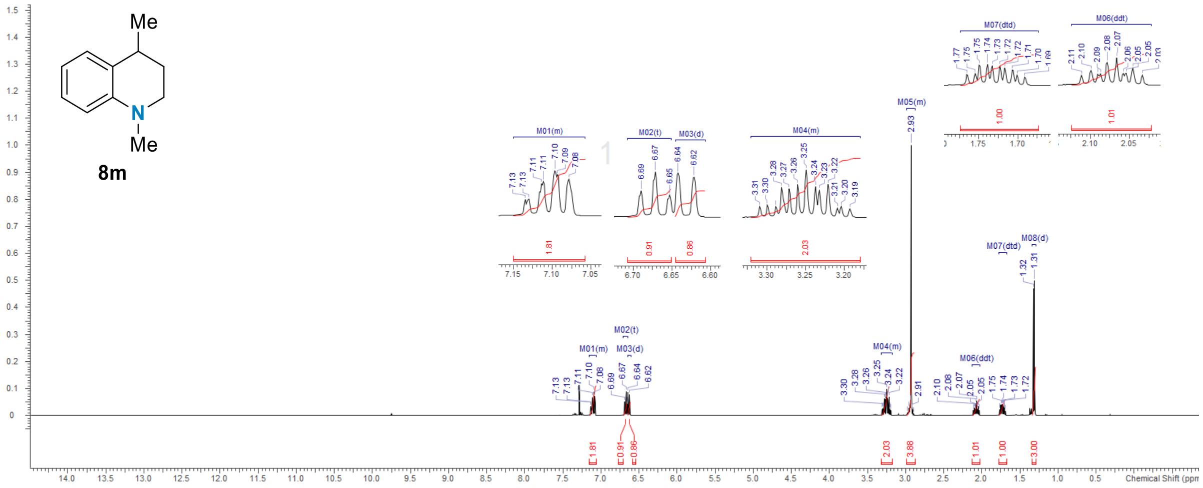

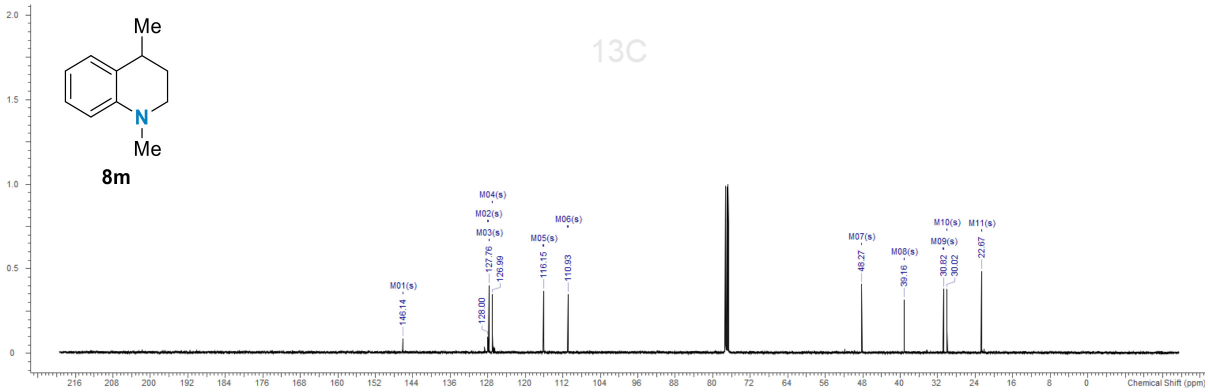

1-Methyl-4-phenyl-3,4-dihydro-2*H*-quinoline (**8n**)

Solvent: $CDCl_3$, ^1H NMR (400 MHz), ^{13}C NMR (101 MHz).

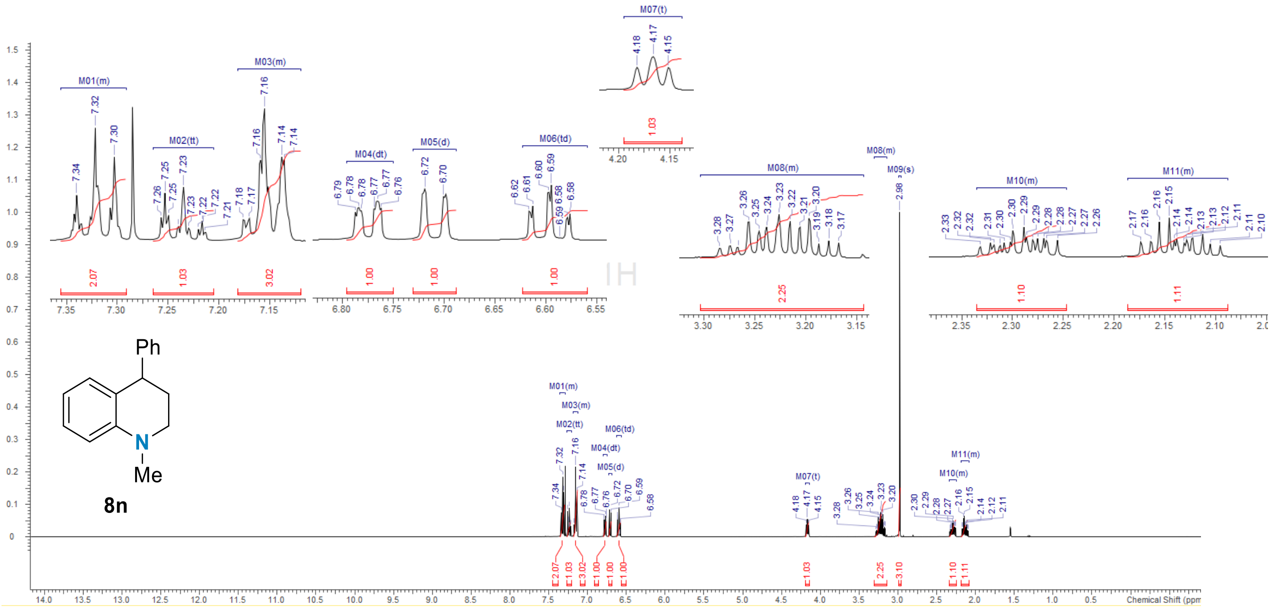

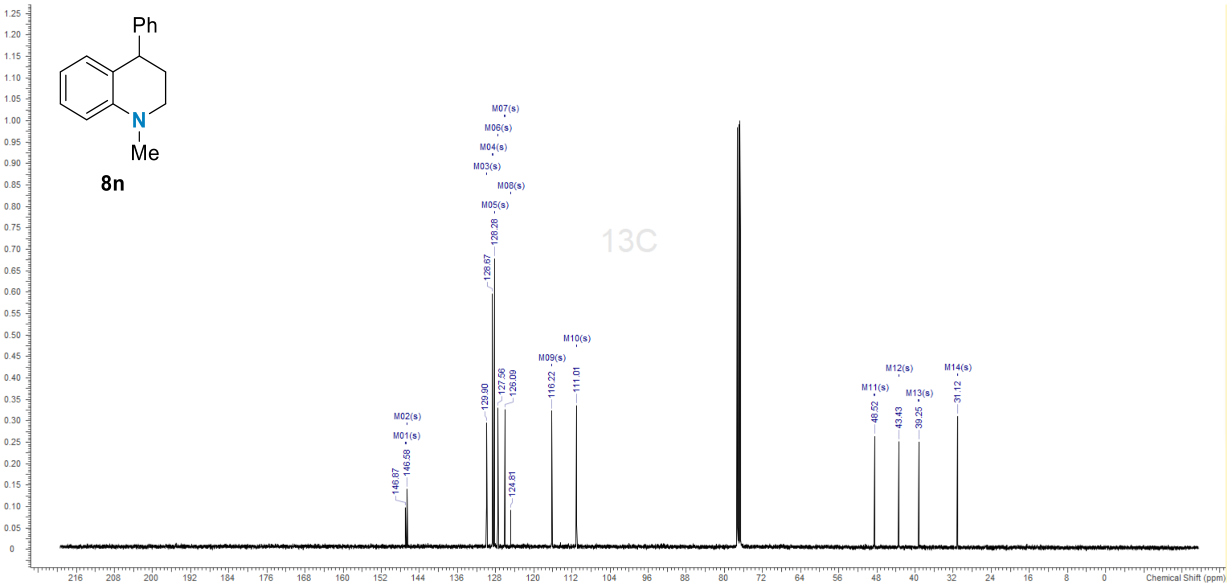

4-Methyl-3,4-dihydro-2*H*-benzo[*b*][1,4]oxazine (**8o**)

Solvent: $CDCl_3$, ^1H NMR (400 MHz), ^{13}C NMR (101 MHz).

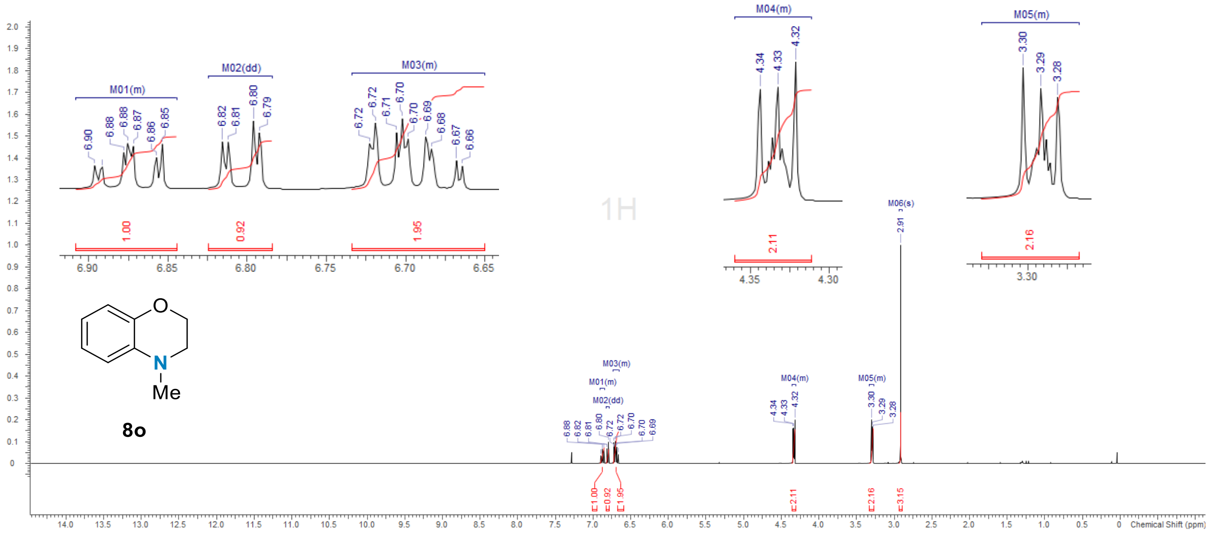

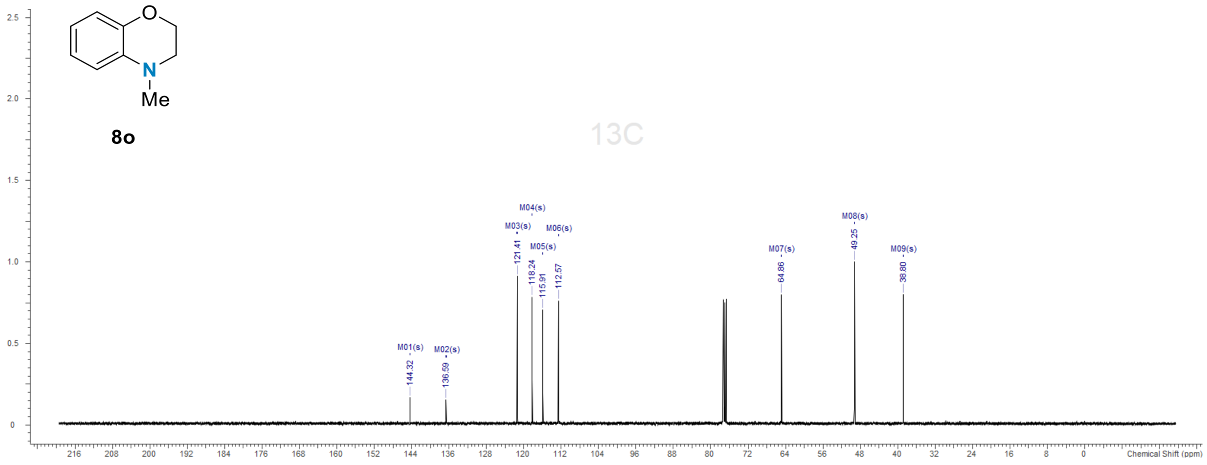

Supplement: Supplementary file 1 — ol4c00179_si_001.pdf [file ol4c00179_si_001.pdf]
